# Phylodynamics and evolutionary epidemiology of African swine fever p72-CVR genes in Eurasia and Africa

**DOI:** 10.1371/journal.pone.0192565

**Published:** 2018-02-28

**Authors:** Moh A. Alkhamis, Carmina Gallardo, Cristina Jurado, Alejandro Soler, Marisa Arias, José M. Sánchez-Vizcaíno

**Affiliations:** 1 Faculty of Public Heath, Health Sciences Centre, Kuwait University, Kuwait City, Kuwait; 2 Department of Veterinary Population Medicine, College of Veterinary Medicine, University of Minnesota, St. Paul, Minnesota, United States of America; 3 European Union Reference Laboratory for African swine fever. Centro de Investigación en Sanidad Animal (INIA-CISA), Madrid, Spain; 4 VISAVET Health Surveillance Centre and Animal Health Department, Veterinary School, Complutense University of Madrid, Madrid, Spain; University of Minnesota, UNITED STATES

## Abstract

African swine fever (ASF) is a complex infectious disease of swine that constitutes devastating impacts on animal health and the world economy. Here, we investigated the evolutionary epidemiology of ASF virus (ASFV) in Eurasia and Africa using the concatenated gene sequences of the viral protein 72 and the central variable region of isolates collected between 1960 and 2015. We used Bayesian phylodynamic models to reconstruct the evolutionary history of the virus, to identify virus population demographics and to quantify dispersal patterns between host species. Results suggest that ASFV exhibited a significantly high evolutionary rate and population growth through time since its divergence in the 18th century from East Africa, with no signs of decline till recent years. This increase corresponds to the growing pig trade activities between continents during the 19th century, and may be attributed to an evolutionary drift that resulted from either continuous circulation or maintenance of the virus within Africa and Eurasia. Furthermore, results implicate wild suids as the ancestral host species (root state posterior probability = 0.87) for ASFV in the early 1700s in Africa. Moreover, results indicate the transmission cycle between wild suids and pigs is an important cycle for ASFV spread and maintenance in pig populations, while ticks are an important natural reservoir that can facilitate ASFV spread and maintenance in wild swine populations. We illustrated the prospects of phylodynamic methods in improving risk-based surveillance, support of effective animal health policies, and epidemic preparedness in countries at high risk of ASFV incursion.

## Introduction

African swine fever (ASF) is a complex infectious disease of swine classified as a notifiable infection to the World Organisation for Animal Health. Neither vaccine nor treatment is available against this disease. Therefore, ASF control and eradication is based on rapid field recognition, isolation of suspected cases and diagnosis, followed by implementation of strict sanitary measures [1, 2]. Thus, the presence of ASF constitutes devastating impacts on animal health and the world economy due to stamping out policies and trade restriction at national and international levels [1].

ASF is caused by a complex, large, enveloped DNA virus currently classified as the only member of the family *Asfarviridae* [3]. The viral genome consists of a central conserved region and two variable ends. Therefore, ASFV DNA molecule length may range between 170 and 193 kilobase (kb) depending on isolates [4]. ASFV genotyping is usually based on partial sequence analysis of the B646L gene encoding the viral protein 72 (vp72). Thus far, 24 genotypes have been identified [5–7]. Sequence analysis of tandem repeats in the central variable region (CVR) within the B602L gene [8], the intergenic region between I73R and I329L genes [9] and the EP402R and MGF505-2R regions [10, 11] permit to distinguish between closely related ASFV isolates.

This disease was described for the first time in Kenya, in 1921 [12]. However, ASFV has not been confined to the African continent since its discovery. Several introductions from Africa into Europe have been described so far [1]. The first incursion from Angola to Lisbon took place in 1957. Three years later, in 1960, ASF reached Lisbon for the second time from there it spread to other European, Caribbean and South American countries. The disease was successfully eradicated from all these territories except from the Italian island of Sardinia where the disease remains endemic since 1978 [13]. In 2007, a new incursion of ASFV from Southeast Africa to Georgia was observed. Since then, ASF has spread northward and westward affecting the whole Caucasus region (2007) the Russian Federation (2007), Ukraine (2012), Belarus (2013), the Baltic countries (2014), Poland (2014), Moldova (2016), Czech Republic and Romania (2017) [14]. To date, ASFV has been described in more than 28 sub-Saharan countries [14, 15] as well as in previously mentioned European states.

In the current and past affected areas, different transmission models affecting domestic pigs, wild boar, wild African suids (warthogs, bush pigs, and giant forest hogs), and soft ticks of the genus *Ornithodoros* were identified [1, 16]. *Ornithodoros* ticks act as biological vectors and reservoirs of ASFV [17], able to transmit the virus even after years of absence from the viraemic hosts [18, 19]. On the South and East part of the African continent, the most complex transmission cycle involves wild African suids, domestic pigs and *O*. *moubata* complex ticks [1, 20]. On the Iberian Peninsula, a similar model was identified, where wild boar, outdoor domestic pigs, and *O*. *erraticus* ticks cohabited [17]. In addition to this, a domestic pig-tick cycle without wild suids involvement has also been reported in some African areas such as Mozambique, Malawi and the Southwest of the Iberian Peninsula [19, 21–23]. While in Eastern Europe, Sardinia and West Africa, the transmission cycle seems to implicate infected domestic pigs and/or wild boar without the involvement of ticks [20, 24].

Despite efforts made to control and eradicate the disease in Europe, ASF continues to affect domestic pigs and wild boars [24–26] which poses a threat for other pig producers and ASF-free wild populations. Since the European Union (EU) Council Decision of 1990 (EU Official Bulletin no. L 116, 1990), ASF surveillance activities have undergone substantial developments within the EU member states. Such surveillance activities have included targeted serological and virological testing of pigs and wild boar in high-risk areas, risk assessments, epidemiological investigations, and pre-movements tests [27]. Furthermore, the EU Council decision of 2002 and 2003 (Council Directive 2002/60/EC and Commission Decision 2003/422/EC) advocated for the need to assess the tick-wild boar cycle and acknowledged its importance as a key component of ASF surveillance. However, such a decision has further complicated ASF intervention measures, due to the lack of sufficient scientific data about the tick-wild boar interface [28].

Molecular surveillance of ASFV is an integral part of the disease intervention activities in Europe and Africa. Most published studies used molecular characterization of vp72 or/and CVR gene segments for genotyping, subgrouping close related isolates, [11, 29–31] and investigating the molecular epidemiology of the virus using traditional phylogenetic methods, such as neighbour-joining or maximum likelihood techniques [9, 32–34]. Furthermore, such studies draw conclusions on the evolutionary origins of isolates through examining the phylogenetic, spatial and temporal characters in an entirely separate analytical setting [33–36]. These studies ignored important uncertainties and parameters associated with the estimates of the phylogenetic relationships, spatial and temporal factors [37, 38]. Subsequently, past methodological approaches used to study the virus ignored that evolutionary and epidemiological characters of pathogens like ASF occur on approximately the same time-scale. Therefore, they must be considered in an integrated analytical setting to be properly investigated, prevent biased conclusions, and improve surveillance-related decision making [39].

In the past decade, the field of phylodynamics has become well established for investigating the evolutionary epidemiology of animal and human pathogens, [40–44] as it aims to model the joint evolutionary and epidemiological characteristics of the virus using a unified Bayesian statistical framework [45]. This approach treats evolutionary, host species, spatial, and temporal parameters as random variables and assigns them prior probability distributions to infer their corresponding posterior probability distributions [45]. This property provides a powerful molecular tool that accounts for the uncertainties in the phylogeny, viral population demographics, and spatiotemporal dispersal between geographical regions and host species, which corresponds to long-standing questions [38, 46].

To our knowledge, only Michaud *et al*. 2013 [47] advocated for the use of phylodynamic methods for molecular dating and genotyping of ASFVs. The objective of this study was to investigate the evolutionary epidemiology of ASFV in Eurasia and Africa using the vp72 and CVR combined gene sequences collected between 1960 and 2015. We used several Bayesian phylodynamic models to reconstruct the evolutionary history of the virus to identify its population demographics and other relevant evolutionary parameters. For the first time, we used discrete-trait phylodynamic models to quantify viral population demographics through time and dispersal patterns between and within continents as well as between host species. Our study illustrates the utility of Bayesian phylodynamic methods in improving ASFV molecular surveillance and decision making related to its intervention measures.

## Materials and methods

### Sequence data

**Fig 1:**
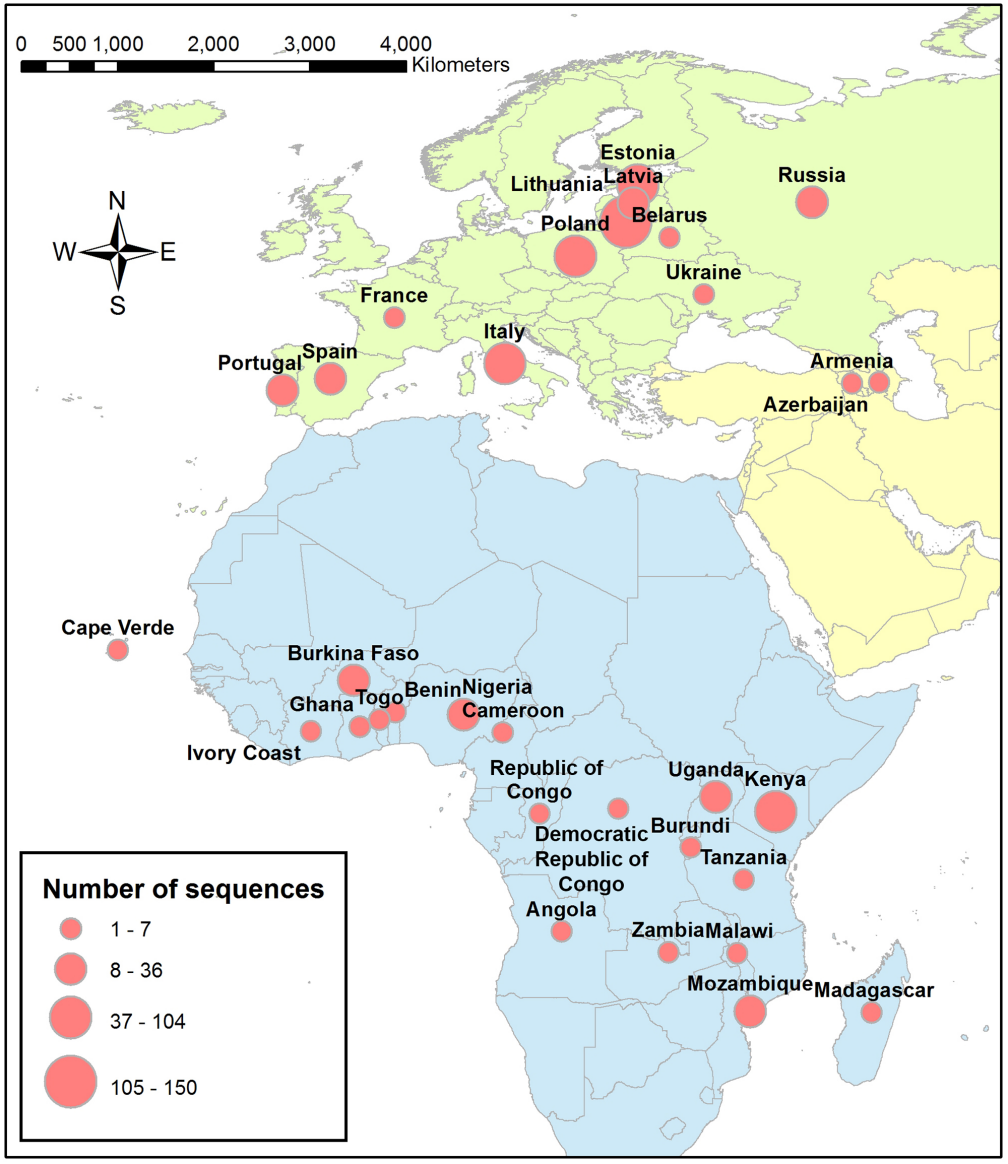
Geographical distribution of ASF sequences isolated in Eurasia and Africa between 1960 and 2015 (N = 665). Red circles indicate locations of ASFV isolates, where their CVR and vp72 gene segments were sequenced. The circles’ size is proportional to the number of isolates.

Partial sequencing of the B646L gene encoding the vp72 and tandem repeats in the CVR within the B602L gene were obtained from field isolates circulating between June 1960 and July 2015 in Eurasia and Africa. All sequences used in this study are available on request at the European Union Reference Laboratory for ASF (at http://asf-referencelab.info/asf/en/) (S1 Table). Sequences consisted of the partial sequencing of the B646L gene encoding the vp72 and tandem repeats in the CVR within the B602L gene. Sequence dataset contained information including isolate name, country of origin, collection date, and affected host. Sequencing was performed according to described procedures [48] at both national and the EU reference laboratories for ASF. The data consisted of a total of 665 sequences collected from 14 Eurasian, 8 East African and 11 West and Central African countries between June 1960 and July 2015 (these isolates had available sequences of both segments) (S2 Table and Fig 1). Date of collection for each sequence was converted into fractional years to estimate divergence times. However, 12.5% of the sequence dataset only had year-specific information; therefore, the date of collection was specified as the mid-point of the corresponding year.

AliView version 1.18 [49] was used to concatenate vp72 and CVR into one gene segment (vp72-CVR) for the subsequent analysis. MUSCLE version 3.8.3 was used to align the concatenated sequences and the resulting alignment was assessed manually by translating the reading frame into amino acids. Maximum likelihood (ML) of the phylogeny and tree topology for the sequence dataset was estimated under the GTR+Γ substitution model using RAxML version 8.0 [50], in which node support was estimated using 10 through bootstrap searches with 100 ML replicates (S1 Fig). We assessed the coefficient of concordance for the sequence data distance matrices using the ‘CADM.global’ function implemented in ‘Ape’ R statistical package [51] and rejected the null hypothesis that all matrices are incongruent (p-value < 0.05). Our preliminary phylogenetic analyses illustrated most of the sequences were collected in the recent years (S2 Fig) and 100% identical, where they clustered with no phylogenetic structure on the upper branch of the ML tree (S1 Fig). Thus, we decided to discard sequences with 100% nucleotide identity (86%) and proceeded with the subsequent analyses with the remaining sequences (n = 96; S3 Fig). This made the subsequent analyses less computationally demanding and will assure better model convergence as suggested elsewhere [52–54]. Recombination events in the selected sequences were not detected using Recombination Detection Program version 3.0 [55]. Finally, we used TempEst version 1.5.1 [56] to explore the presence of temporal structure in the sequence data and estimated a positive correlation (correlation coefficient = 0.190) between genetic divergence and sampling time. This suggested that the sequence data is suitable for the subsequent phylogenetic molecular clock analyses.

### Inferring ASFV population demographics and divergence times

Virus population demographics and time to the most recent common ancestor (TMRCA) were estimated using the relaxed-clock models implemented in BEAST version 1.8.4 [57] within a Bayesian statistical framework. Best partition scheme for the substitution models of the sequence alignment was selected using the Bayesian Information Criterion (BIC) [58] implemented in PartitionFinder version 1.1.1 [59].

Tree tips were calibrated with isolation dates of the sequences in order to estimate divergence time. Best fitting node-age tree model for the sequences data was selected through evaluating four parametric and one non-parametric coalescent priors to infer the most realistic population growth patterns of the virus through time [60]. The parametric coalescent priors included: (1) the constant population size (CP) [61]; (2) the expansion growth (EGx) [62]; (3) the exponential growth (EG) [62]; and (4) the logistic growth (LG) [62]; while the non-parametric coalescent prior was (5) the Bayesian Skygrid model, which implement a Gaussian Markov random fields (GMRF) prior to smooth the trajactories of the past population dynamics [63]. For each coalescent tree model, two branch-rate priors were further evaluated, namely, the uncorrelated lognormal (UCLN) and exponential (UCED) branch-rate priors [64]. The continuous-time Markov chain (CTMC) hyperprior [65] implemented in BEAST was used to infer the parameters of the branch-rate prior distribution. Thus, using the Bayes factor (BF) comparison approach, the fit of ten candidate relaxed-clock models were evaluated, which comprised all combinations of: (1) a single mixed-substitution model; (2) five coalescent tree priors (CP, EG, EGx, LG and GMRF); and (3) two branch-rate models (UCLN and UCED). This was achieved through estimating the marginal-likelihood of each candidate model using ‘stepping-stone’ sampling (SS) [66] and ‘path-sampling’ (PS) [67] methods implemented in BEAST. Then the resulting marginal-likelihood estimates were used to select among the corresponding relaxed-clock models using BF comparison.

Finally, posterior parameters of the phylogeny and divergence time under each candidate relaxed model were estimated using two replicate Bayesian Markov chain Monte Carlo (MCMC) simulations for 200 million cycles and sampled every 2000^th^ state. Tracer version 1.6 [68] was used to calculate the effective sample size (ESS) as an evaluation criterion (*i*.*e*. ESS > 200) for the proper convergence of each MCMC simulation of every posterior parameter. From each chain, the first 10% of the samples was discarded as burn-in. Then, the resulting marginal posterior probability density was summarised as a maximum clade credible (MCC) tree, with median node ages using TreeAnotator version 1.8.4. A Bayesian Skygrid (BSg) plot was generated using Tracer to provide temporally smoothed estimates of the changes in the effective population size trajectories of the virus between 1960 and 2015. This plot could be used as a proxy for the genetic diversity of vp72-CVR gene through time [69].

### Inferring ASFV phylogeographic history and transmission between host species

Phylodynamic histories of ASFV between host species from 1960 to 2015 were inferred using discrete-trait ancestral reconstruction phylodynamic models implemented in BEAST [48]. Selected discrete traits included a total of three host groups namely domestic pig, wild suids and ticks (S3 Table). The best fitting coalescent tree model and branch-rate prior combinations, described in the above analyses, was used for the subsequent phylodynamic models. The fit of the sequence data was further assessed for two candidate discrete-trait phylodynamic models, namely the symmetric model and the asymmetric model, which allows the non-zero rates of change between each pair of discrete states to be equal (reversible transitions) or differ (irreversible transitions), respectively. Furthermore, Bayesian stochastic search variable selection (BSSVS) [38] was used to eliminate non-significant elements of the matrix specifying the non-zero rates of change between each pair of discrete host species. Finally, the mean number of dispersal events between each pair of discrete traits was inferred using the Markov-jump approach [70]. This method uses a robust-counting MCMC approach to infer intensity of backward and forward transitions within a matrix of discrete traits [70].

Finally, Kullback-Leibler (KL) divergence statistic [71] and association index (Ai) [72] were used to validate the discrete trait prior and posterior estimates to accommodate phylogenetic uncertainty of the selected models. The KL statistic was calculated using the Razavi function [73] and Matlab version 2016a [74] to measure the departure of the posterior estimates inferred from the selected phylodynamic models from its underlying priors. The Ai statistic was calculated using Bayesian Tip-Significance Testing (BaTS) version 1.0 [72] to test the presence of structure in the evolutionary diffusion of the virus caused by the selected discrete trait.

## Results

### Demographic history of ASFV in Eurasia and Africa

**Fig 2:**
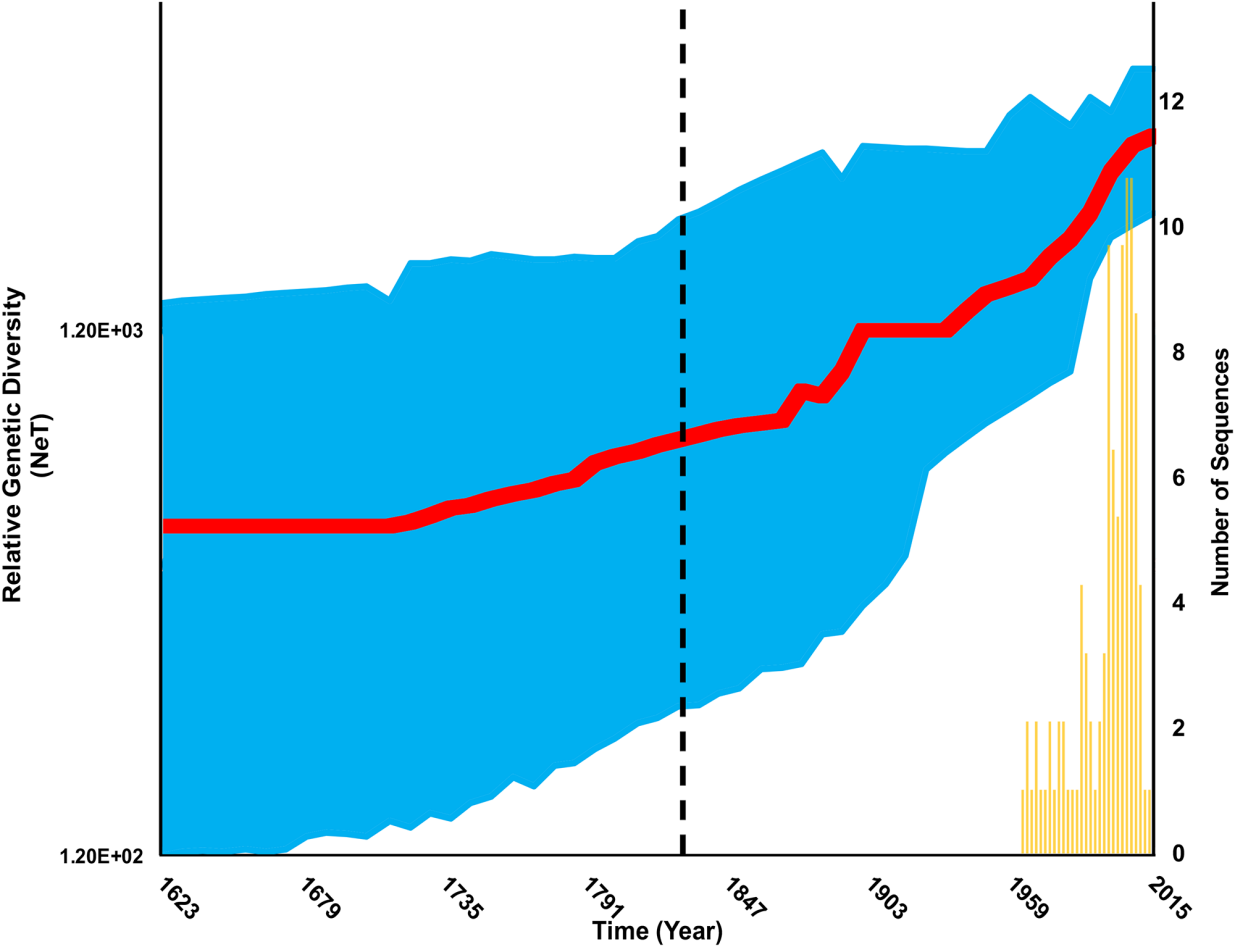
Bayesian Skygrid plot for temporal variation in the effective population size of ASF vp72-CVR genes in Eurasia and Africa between 1960 and 2015. The posterior median estimate is indicated by the red line; the blue lines correspond to the 95% HPD. Vertical dotted line represents the estimated time at which the population growth transitioned from a slow rate to a fast rate.

**Fig 3:**
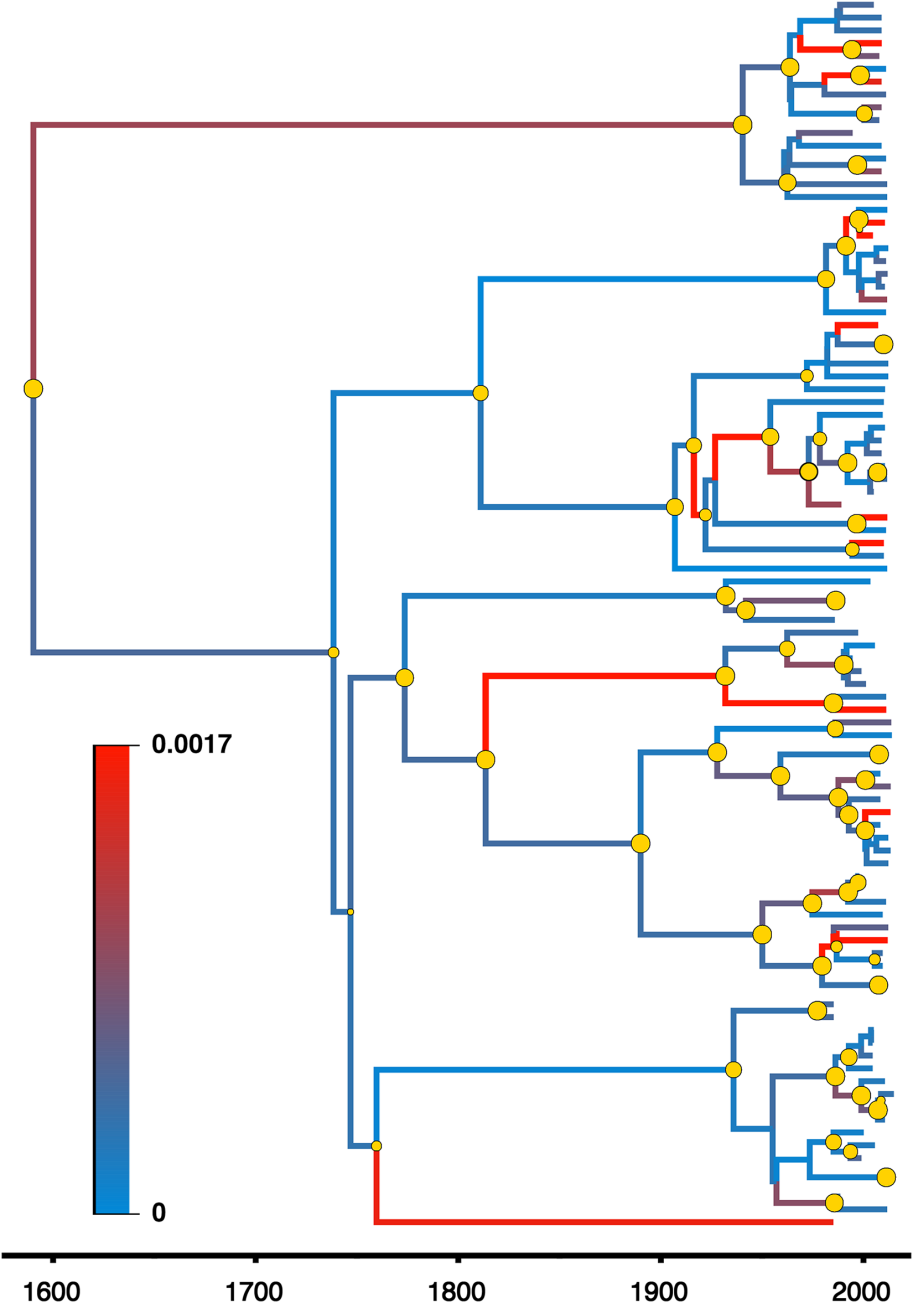
Maximum clade credibility (MCC) phylogeny of ASF vp72-CVR genes in Eurasia and Africa between 1960 and 2015 estimated under best fitting demographic model (S4 Table). The colour of the branches represents the among-branch evolutionary rate and corresponds to the colour gradient legend on the lower left. Well-supported posterior probabilities (P > 0.65) of branching events are indicated by yellow circles. The size of the yellow circles is proportional to the inferred posterior probabilities.

Vp72-CVR ASFV sequences selected by the ML analysis (S2 Fig) significantly favoured the parametric EG coalescent tree model with the UCED branch-rate prior, using the BF comparisons (BF > 15.9) of the SS and PS marginal likelihoods estimators (S4 Table, S1 File). The inferred posterior estimate of the mean nucleotide substitution rate was equal to 3.31 × 10^−4^ /site/year with a 95% highest posterior density (HPD) ranging from 8.51 × 10^−5^ to 5.89 × 10^−4^. Oldest TMRCA for vp72-CVR sequences for viruses isolated from outbreaks in Africa and Europe was approximately 243 years ago in East Africa (Table 1). While, viruses isolated from Eurasia and West Africa were younger by 87 and 68 years, respectively (Table 1). The BSg plot, generated from the BSg coalescent tree model with the UCLN branch-rate prior (S1 File), showed a slow, steady increase in the virus genetic diversity through time, followed by a distinct continuous increase after the 1800s with no sign of decline (Fig 2). The estimated exponential growth rate of the virus isolates was 0.01 year^-1^ (95% HPD: 0.001 to 0.022 year^-1^). A higher evolutionary rate was inferred after the 1800s among branches of the virus phylogeny (Fig 3).

**Table 1:** Time to the most recent common Ancestor (TMRCA) of vp72-CVR genes of ASF in Eurasia and Africa between 1960 and 2015.

Region	Mean Years	95% HPD*
Eurasia	1861	(1940, 1729)
East Africa	1774	(1937, 1566)
West Africa	1842	(1927, 1713)

*Highest posterior density.

### Phylodynamic history of ASFV between host species in Eurasia and Africa

**Fig 4:**
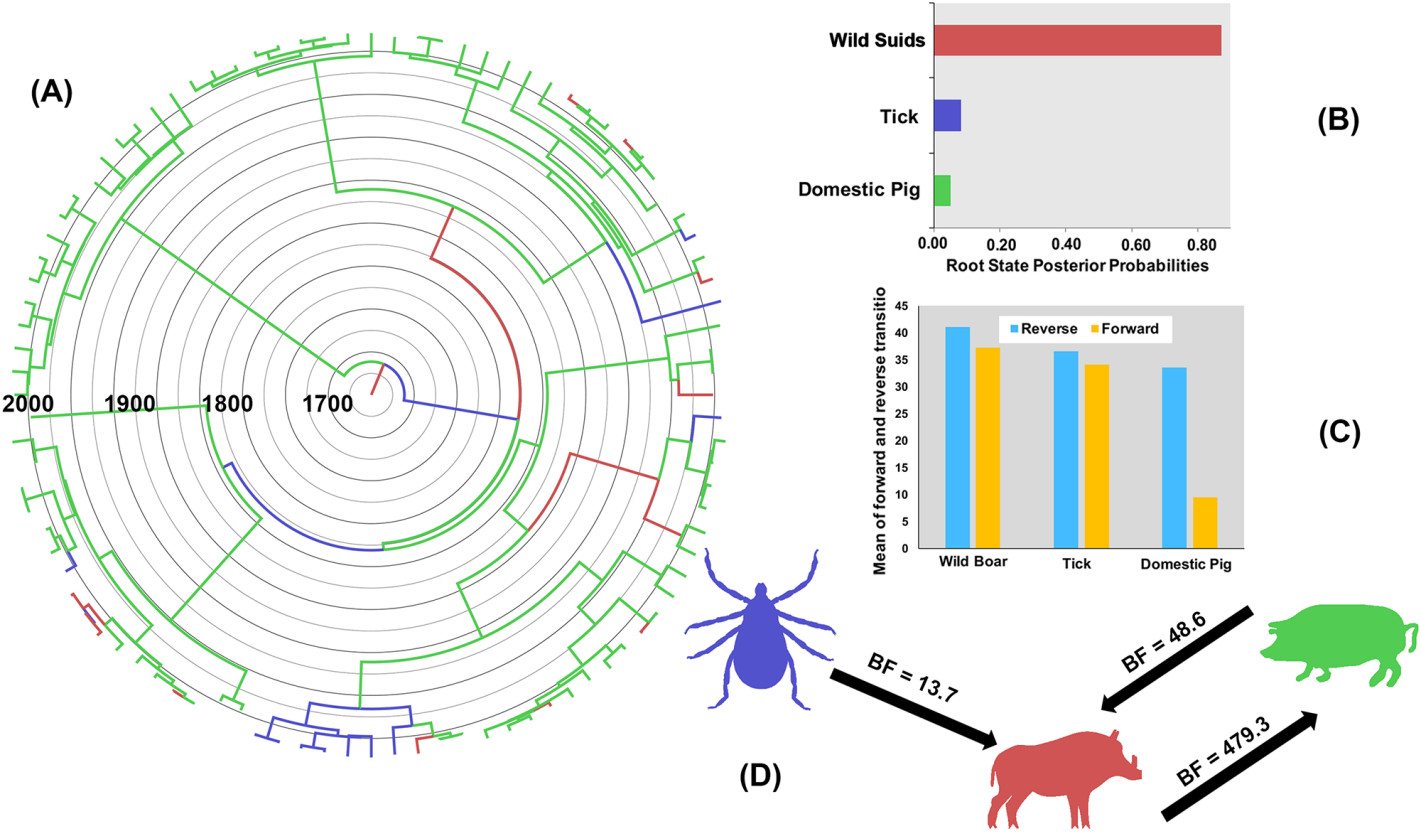
Host species Phylodynamics of ASF vp72-CVR genes in Eurasia and Africa between 1960 and 2015. A) MCC phylogeny with its branches coloured by the most probable host species state of their descendent nodes. B) represents the root location state posterior probability distributions and corresponds to the colour-coding of (A). C) mean forward and reverse transitions estimated by Markov jumps (MJ) approach between hosts (D) represents inferred transmission routes between host species, mean counts MJ of forward and reverse transitions and significant connections (BF > 13) with their directions between hosts.

BF comparisons indicated that the asymmetric model provided the best fit for the vp72-CVR sequences when using host species as a discrete state (BF > 50). This result suggests that the non-zero rates of change of the virus when it jumps between host species differ, and thus, the directionality of the transmission is significant. Wild suids were the most likely ancestral host for ASFV transmission between hosts (Fig 4A) as suggested by the substantially high root state posterior probability (RSPP = 0.87; Fig 4B). Furthermore, wild suids had the highest mean counts of relative forward and reverse transitions between host species (Forward = 41.0 *vs* Reverse = 37.2; Fig 4C), while domestic pig had the lowest mean counts of relative transitions between host species (Forward = 33.5 *vs* Reverse = 33.5; Fig 4C). Only three significant transmission routes inferred between host species (BSSVS BF > 13; Fig 4D) including two, back and forth, between wild suids and domestic pig, and one from tick to wild suids (Fig 4D). The most significant viral transmission route (BSSVS BF = 479.3) inferred from wild suids to domestic pig (Fig 4D). Finally, no significant transmission routes of ASFV inferred between tick and domestic pig (Fig 4D). KL statistic suggests a decent statistical power (KL = 0.81) of the selected host species model, which indicate that the selected discrete trait (i.e. host species) generated RSPPs that are slightly different from the underlying priors. Furthermore, the borderline statistically significant AI (p-value = 0.07) suggests that the evolutionary diffusion of ASFV between hosts is relatively structured.

## Discussion

This study provided new deeper insights into the evolutionary epidemiology of ASFV in Eurasia and Africa, regions that are important for virus emergence, maintenance, and spread. For the first time, Bayesian phylodynamic analyses of the combined vp72-CVR gene segments revealed that current infectious ASFV was not only the result of a complex evolutionary processes of the virus through time in Eurasia and Africa, but it was also the result of a transmission cycle between host species. This study also identified important viral dispersal and transmission routes between Eurasian and African host species.

Demographic reconstruction through-time of vp72-CVR gene sequences suggests a high evolutionary rate (*i*.*e*., substitution rate = 3.31 × 10^−4^ /site/year and exponential growth of 0.01 year^-1^) for ASFVs isolated from outbreaks in Eurasia and Africa between 1960 and 2015. Our estimated evolutionary rate is similar to rapidly evolving RNA viruses and relatively higher than other DNA viruses, as suggested elsewhere [47, 75]. Estimates of the divergence time (*i*.*e*., TMRCA) confirms the common notion of ASFV being native to East Africa [1, 12], where the virus first emerged in the 1700s (Table 1). Our estimates of the evolutionary rate and TMRCA inferred from vp72-CVR sequences are similar to Michaud *et al*. 2013 [47]. However, Michaud *et al*. made his inferences based on independent analyses of the three gene segments namely B646L (vp72), E183L and CP204L [47]. Inferred divergence times summarised in Table 1 further confirms that common ancestors of ASFV isolated from Eurasia and West Africa were younger than those isolated in East Africa.

The estimated genetic diversity clearly represents the inferred rapid exponential growth of ASFV through time (Fig 2). Indeed, throughout the centuries, ASFV only showed this rapid growth behaviour after its ancestors emerged from West Africa and Eurasia, which coincided with the time when major trade routes between continents started to flourish and peak in the 18th and 19th centuries. Furthermore, genetic diversity of ASFV started to peak significantly after the 1800s with no signs of decline till 2015 (Fig 2). This peaking is potentially attributed to the British colonisation of Kenya, in which the swine industry became substantially larger due to the massive importation of domestic pigs [47, 76]. This higher growth rate may suggest expanding diversity through time which corresponds to the growing pig trade activities between continents during the 19th century. This finding may also be attributed to an evolutionary drift that resulted from either continuous circulation or maintenance of the virus within Africa and Eurasia. Indeed, many of the recently isolated ASFV lineages exhibited a rapid evolutionary rate among the branches of the inferred posterior phylogeny (Fig 3).

Results of the host species phylodynamic model strongly implicate wild suids as the ancestral host species (RSPP = 0.87) for ASFV in the early 1700s in Africa (Figs 4A and 3B). The major two branches diverging from the root of the MCC tree (Fig 4A) represents two different transmission cycles for ASFV between host species. First, the Eurasian cycle between wild boar and domestic pigs that included only European isolates. The second, and the more complex African cycle between wild African suids, (known reservoirs and carriers of ASFV) ticks, and domestic pigs (Fig 4A). The later transmission cycle resulted in more significant diversification events of ASFV (*i*.*e*., larger sub-tree) in Africa and Europe than the earlier cycle (Fig 4A) [1, 24]. Also, our results indicate that the virus jumped more frequently from wild suids and ticks than from domestic pigs (Fig 4C), which suggests that both wild suids and ticks maintained an old and indefinite transmission cycle, still present in Africa, that later started infecting domestic pigs [47]. This might be explained by the ecology of some wild suids such as warthogs, that live in burrows containing infected ticks [20]. Transmission from wild African suids to domestic pigs would need to be mediated by tick bites or directly, when domestic pigs feed on carcasses of infected wild animals [20]. Fig 4D summarises the complex transmission cycle of ASFV in Eurasia and Africa since its divergence in the 1700s. As expected, the most significant transmission route is from wild suids to domestic pig (BSVSS BF = 479.3), while the opposite transmission route was substantially less significant (BSVSS BF = 48.6). These results suggest that the transmission cycle between wild suids and pigs, as well as within domestic pigs, are the most important cycles for ASFV spread and maintenance in Eurasia and Africa (Fig 4) [1, 77]. However, the inferred significant unidirectional transmission route from tick to wild suids confirms that ticks are an important natural reservoir that can facilitate ASFV spread and maintenance in wild suid populations (Fig 4D) [17, 78]. Furthermore, results confirm the notion that transmission cycle between pig and ticks is rare [47].

One important limitation of our study was that the reconstructed phylodynamic model was based on a biased subset of vp72 and CVR sequence data. Moreover, we only used 13% of the available sequence data due to the severe lack of phylogenetic structure in the remaining 87%. Hence, we were not able to model the phylogeographic history of ASFV between and within affected countries or geographical regions. Although we had tried to run our models using all sequence data, their convergence and uncertainty statistics (i.e., KL and AI) were severely poor. That said, our study is based on all available viruses of which their vp72 and CVR gene segments have been sequenced and associated with notable ASFV outbreaks in Eurasia and Africa, and therefore reflects our best understanding of ASFV evolutionary history on country and regional levels. Due to some limitations in the database (for instance, the fewer number of sequences coming from ticks and wild African suids), transmission models between hosts partially showed the complexity of ASF epidemiology. This fact might lead to certain bias in obtained result, especially in areas where wild suids, domestic pigs and tick cohabit. This situation is clearly manifested with regard to the transmission of ASFV between domestic pigs and ticks. In countries such as Malawi and Mozambique in Africa, and Spain and Portugal in Europe, the cycle tick-domestic pig has been described after finding ASFV positive ticks in pig shelters [21–23, 79, 80]. In such scenarios, the presence of ticks caused outbreaks without any apparent wild suids involvement, long persistence of ASFV (even for years) in the environment/animal facilities, [18] and re-emergence of the ASFV in areas considered to be disease-free [81, 82]. However, results obtained from this study were not able to show this transmission cycle, suggesting that there is still room for improvements when further sequences, segments or full genomes are available, as well as information related to swine farm demographics and movements within and between continents.

While Michaud *et al*. 2013 [47] endorsed the use of Bayesian phylodynamic methods for molecular dating of ASFV sequences as well as for revising its genotyping classification method, here we further recommend the utilization of these analytical methods for guiding risk-based surveillance, control, and prevention efforts. Our results provided plausible biological inferences about the evolutionary history of ASFV within geographical regions and host species in susceptible areas like Eurasia and Africa by using a vp72-CVR sequence data set and its related epidemiological information. Unfortunately, analytical methods used in this study have not been fully or even partially used by global animal health agencies for molecular surveillance of ASFV or guiding risk-based interventions. Instead, recent studies of ASFV continues to use traditional phylogenetic methods to infer the evolutionary history [9, 33, 34, 83]F without quantitatively accounting for time or other epidemiological characteristics of the virus or its host species in the inferred phylogeny. In this study, we demonstrated the prospects of our analytical approach which provided deeper insights into the evolutionary epidemiology of ASFV. The ability to specify priors for ASFV evolutionary parameters and selection of different model assumptions provides a robust tool to identify new viruses, genotyping of new viral clades, and reconstruct phylogenetic relationships between isolated strains [47].

Furthermore, molecular surveillance of ASFV evolutionary characteristics can be used to evaluate the effect of intervention measures, such as movement restriction or stamping out, on the rate of evolution and genetic diversity of the virus. Indeed, including phylodynamic methods in the set of available analytical tools will support the development of effective animal health policies. It will also aid epidemic preparedness in neighbouring ASFV free-countries, especially when the genetic diversity of ASFV continues to increase as described above.

## Conclusions

In this study, we presented a novel attempt to rigorously model the evolutionary epidemiology of ASFV in Eurasia and Africa using several variants of the Bayesian phylodynamic models. Results suggest that ASFV vp27-CVR gene sequences isolated from outbreaks in Eurasia and Africa between 1960 and 2015 exhibited a significantly high evolutionary rate since its divergence in the 18th century from East Africa with no sign of decline till 2015. Increase in the genetic diversity suggests a genetic drift and corresponds to the growing pig trade activities between continents during the 19th century. Furthermore, results implicate wild suids as the ancestral host species for ASFV in the early 1700s in Africa. Two important transmission routes were inferred between wild suids and domestic pig, while one unidirectional transmission route inferred from tick to wild suids. These results indicate the transmission cycle between wild suids and pigs is an important cycle for ASFV spread and maintenance in pig populations, while ticks are an important natural reservoir that can facilitate ASFV spread and maintenance in wild suids populations. We illustrated the prospects of phylodynamic methods in improving risk-based surveillance, support of effective animal health policies and epidemic preparedness in countries at high risk of ASFV incursion.

## Supporting information

S1 Table List of ASF virus isolate names collected in Eurasia and Africa between 1960 and 2015 per country (N = 665). (DOCX)

S2 Table Summary profile of ASF vp72-CVR gene sequences isolated in Eurasia and Africa between 1960 and 2015 per country (N = 665). (DOCX)

S3 Table Summary profile of ASF vp72-CVR gene sequences isolated in Eurasia and Africa between 1960 and 2015 (N = 665) per geographical region and host species. (DOCX)

S4 Table Bayes factors (BFs) comparisons of ASF vp72-CVR genes relaxed-clock models using stepping-stone (SS) and path-sampling (PS) methods. BFs based on SS marginal likelihood estimates are on the upper off-diagonal of this table, while BFs based on PS marginal likelihood estimates are on the lower off-diagonal of this table. Best fitting model is boldfaced.
(DOCX)

S1 Fig Maximum likelihood phylogeny of vp72-CVR sequences of ASF in Eurasia and Africa between 1960 and 2015, using the GTR+Γ substitution model. Support given at nodes based on through bootstrap search using 10 runs, with 100 ML replicates in each run, implemented in RAxML version 8. Scale bar indicate substitution rate per site for all sequence data (N = 665).
(PDF)

S2 Fig Temporal distribution of vp72-CVR sequences of ASF in Eurasia and Africa collected between 1960 and 2015 (N = 665). (TIF)

S3 Fig Maximum likelihood phylogeny of 665 vp72-CVR sequences of ASF in Europe/Asia and Africa between 1960 and 2015, using the GTR+Γ model of evolution. Support given at nodes based on through bootstrap search using 10 runs, with 100 ML replicates in each run, implemented in RAxML version 8. Scale bar indicate substitution rate per site for the selected non-100% identical sequences (n = 96).
(PDF)

S1 File XML files for the exponential, Bayesian skygrid, and host species discrete trait models used to infer the results of the current study. (ZIP)
