# Supplementary material for: Phylodynamics and evolutionary epidemiology of African swine fever p72-CVR genes in Eurasia and Africa
Source: PLoS One. 2018 Feb 28;13(2):e0192565. doi: 10.1371/journal.pone.0192565 (PMC5831051; doi:10.1371/journal.pone.0192565)
Supplement: S1 Table — (DOCX) [file pone.0192565.s001.docx]

**S1 Table. List of ASF virus isolate names and their genotypes collected in Eurasia and Africa between 1960 and 2015 per country (N = 665)**

| **ID** | **ASFV ISOLATE** | **COUNTRY** | **HOST** | **YEAR** | **P72_GB ACC Nº** | **P72**  **GENOTYPE** | **P72 REFERENCE** | **P72 SEQUENCE** | **CVR GB ACC Nº** | **CVR REFERENCE** | **CVR_SEQUENCE** |
| --- | --- | --- | --- | --- | --- | --- | --- | --- | --- | --- | --- |
| 1 | Lisbon60 | Portugal | DP | 1960 | AF301539 | I | [Bastos et al., 2003](file:///C:\Users\Cgallardo\Dropbox\Carmina%20AGOSTO%202014\SECUENCIAS%20CRL\Desktop\Carmina%20MARZO%202014\ARTICULOS\VPPA\GENOTIPADO\2003%20Bastos%20et%20al.pdf) | ATGCAGCCTACTCACCACGCAGAGATAAGCTTTCAGGATAGAGATACAGCTCTTCCAGACGCATGTTCATCTATATCGGATATTAGCCCCGTTACGTATCCGATCACATTACCTATTATTAAAAACATTTCCGTAACTGCTCATGGTATCAATCTTATCGATAAGTTTCCATCAAAGTTCTGCAGCTCTTACATACCCTTCCACTACGGAGGCAATGCAATTAAAACCCCCGATGATCCGGGTGCGATGATGATTACCTTTGCTTTGAAGCCACGGGAGGAATACCAACCCAGTGGTCATATTAACGTATCCAGAGCAAGAGAATTTTATATTAGTTGGGACACGGATTACGTGGGGTCTATCACTACGGCTGATCTTGTGGTATCGGCATCTGCTATTAACTT | DQ02833 | Duarte et al 2005 (Unpublished) | AGTGCGTATACCTGTGCAAGCACTTGTGCAGATACCAATGTAGACACCTGTGCAAGCACTTGTGCAAGCACTTGTGCAAGCACTTGTGCAAGCACAGGTGCAAGCACTTGTGCAGATACCAATGTAGACACCTGTGCAAGCACTTGTGCAGATACCAATGTAGACACCTGTGCAAGCACTTGTGCAGATACCAATGTAGACACCTGTGCAAGCACTTGTGCAGATACCAATGTAAACACTTGTGCAAGCATGTGTGCAGATACCAATGTAGACACCTGTGCAAGCACCTGTGCAAACACCTGTGCAAGCACAGAATAC |
| 2 | Ali61 | Spain | DP | 1961 | FJ154445 | I | [Gallardo et al., 2009](http://asf-referencelab.info/asf/images/files/publicaciones/Gallardo-et-al-2009a.pdf) | ATGCAGCCTACTCACCACGCAGAGATAAGCTTTCAGGATAGAGATACAGCTCTTCCAGACGCATGTTCATCTATATCGGATATTAGCCCCGTTACGTATCCGATCACATTACCTATTATTAAAAACATTTCCGTAACTGCTCATGGTATCAATCTTATCGATAAGTTTCCATCAAAGTTCTGCAGCTCTTACATACCCTTCCACTACGGAGGCAATGCAATTAAAACCCCCGATGATCCGGGTGCGATGATGATTACCTTTGCTTTGAAGCCACGGGAGGAATACCAACCCAGTGGTCATATTAACGTATCCAGAGCAAGAGAATTTTATATTAGTTGGGACACGGATTACGTGGGGTCTATCACTACGGCTGATCTTGTGGTATCGGCATCTGCTATTAACTT | AM259440 | [Nix et al 2006](http://asf-referencelab.info/asf/images/files/publicaciones/Nix_et_al_2006.pdf) | AGTGCGTATACCTGTGCAAGCACTTGTGCAGATACCAATGTAGACACCTGTGCAAGCACTTGTGCAAGCACTTGTGCAAGCACTTGTGCAAGCACAGGTGCAAGCACTTGTGCAGATACCAATGTAGACACCTGTGCAAGCACTTGTGCAGATACCAATGTAGACACCTGTGCAAGCACTTGTGCAGATACCAATGTAGACACCTGTGCAAGCACTTGTGCAGATACCAATGTAAACACTTGTGCAAGCATGTGTGCAGATACCAATGTAGACACCTGTGCAAGCACCTGTGCAAACACCTGTGCAAGCACAGAATAC |
| 3 | Co61 | Spain | DP | 1961 | FJ174346 | I | [Gallardo et al., 2009](http://asf-referencelab.info/asf/images/files/publicaciones/Gallardo-et-al-2009a.pdf) | ATGCAGCCTACTCACCACGCAGAGATAAGCTTTCAGGATAGAGATACAGCTCTTCCAGACGCATGTTCATCTATATCGGATATTAGCCCCGTTACGTATCCGATCACATTACCTATTATTAAAAACATTTCCGTAACTGCTCATGGTATCAATCTTATCGATAAGTTTCCATCAAAGTTCTGCAGCTCTTACATACCCTTCCACTACGGAGGCAATGCAATTAAAACCCCCGATGATCCGGGTGCGATGATGATTACCTTTGCTTTGAAGCCACGGGAGGAATACCAACCCAGTGGTCATATTAACGTATCCAGAGCAAGAGAATTTTATATTAGTTGGGACACGGATTACGTGGGGTCTATCACTACGGCTGATCTTGTGGTATCGGCATCTGCTATTAACTT | AM259431 | [Nix et al 2006](http://asf-referencelab.info/asf/images/files/publicaciones/Nix_et_al_2006.pdf) | AGTGCGTATACTTGTGCAAGCACTTGTGCAAGCACTTGTGCAAGCACTTGTGCAGATACCAATGTAGACACCTGTGCAAGCACTTGTGCAAGCACTTGTGCAAGCACTTGTGCAAGCACTTGTGCAAGCACTTGTGCAAGCACTTGTGCAAGCACTTGTGCAAGCACAGGTGCAAGCACTTGTGCAGATACCAATGTAGACACCTGTGCAAGCACTTGTGCAGATACCAATGTAGACACCTGTGCAAGCACTTGTGCAGATACCAATGTAGACACCTGTGCAAGCACTTGTGCAGATACCAATGTAAACACTTGTGCAAGCATGTGTGCAAACACCTGTGCAAGCACAGAATAC |
| 4 | M61 | Spain | DP | 1961 | FJ174345 | I | [Gallardo et al., 2009](http://asf-referencelab.info/asf/images/files/publicaciones/Gallardo-et-al-2009a.pdf) | ATGCAGCCTACTCACCACGCAGAGATAAGCTTTCAGGATAGAGATACAGCTCTTCCAGACGCATGTTCATCTATATCGGATATTAGCCCCGTTACGTATCCGATCACATTACCTATTATTAAAAACATTTCCGTAACTGCTCATGGTATCAATCTTATCGATAAGTTTCCATCAAAGTTCTGCAGCTCTTACATACCCTTCCACTACGGAGGCAATGCAATTAAAACCCCCGATGATCCGGGTGCGATGATGATTACCTTTGCTTTGAAGCCACGGGAGGAATACCAACCCAGTGGTCATATTAACGTATCCAGAGCAAGAGAATTTTATATTAGTTGGGACACGGATTACGTGGGGTCTATCACTACGGCTGATCTTGTGGTATCGGCATCTGCTATTAACTT | AM259429 | [Nix et al 2006](http://asf-referencelab.info/asf/images/files/publicaciones/Nix_et_al_2006.pdf) | AGTGCGTATACCTGTGCAAGCACTTGTGCAGATACCAATGTAGACACCTGTGCAAGCACTTGTGCAAGCACTTGTGCAAGCACTTGTGCAAGCACTTGTGCAAGCACTTGTGCAAGCACTTGTGCAGACACCTGTGCAAGCACTTGTGCAGATACCAATGTAGACACCTGTGCAAGCACTTGTGCAGATACCAATGTAGACACCTGTGCAAGCACTTGTGCAGATACCAATGTAGACACCTGTGCAAGCACTTGTGCAGATACCAATGTAAACACTTGTGCAAGCATGTGTGCAAACACCTGTGCAAGCACAGAATAC |
| 5 | Co62 | Spain | DP | 1962 | FJ174347 | I | [Gallardo et al., 2009](http://asf-referencelab.info/asf/images/files/publicaciones/Gallardo-et-al-2009a.pdf) | ATGCAGCCTACTCACCACGCAGAGATAAGCTTTCAGGATAGAGATACAGCTCTTCCAGACGCATGTTCATCTATATCGGATATTAGCCCCGTTACGTATCCGATCACATTACCTATTATTAAAAACATTTCCGTAACTGCTCATGGTATCAATCTTATCGATAAGTTTCCATCAAAGTTCTGCAGCTCTTACATACCCTTCCACTACGGAGGCAATGCAATTAAAACCCCCGATGATCCGGGTGCGATGATGATTACCTTTGCTTTGAAGCCACGGGAGGAATACCAACCCAGTGGTCATATTAACGTATCCAGAGCAAGAGAATTTTATATTAGTTGGGACACGGATTACGTGGGGTCTATCACTACGGCTGATCTTGTGGTATCGGCATCTGCTATTAACTT | AM259430 | [Nix et al 2006](http://asf-referencelab.info/asf/images/files/publicaciones/Nix_et_al_2006.pdf) | AGTGCGTATACCTGTGCAAGCACTTGTGCAGATACCAATGTAGACACCTGTGCAAGCACTTGTGCAAGCACTTGTGCAAGCACTTGTGCAAGCACTTGTGCAAGCACTTGTGCAAGCACTTGTGCAGACACCTGTGCAAGCACTTGTGCAGATACCAATGTAGACACCTGTGCAAGCACTTGTGCAGATACCAATGTAGACACCTGTGCAAGCACTTGTGCAGATACCAATGTAGACACCTGTGCAAGCACTTGTGCAGATACCAATGTAAACACTTGTGCAAGCATGTGTGCAAACACCTGTGCAAGCACAGAATAC |
| 6 | Tengani | Malawi | Wild pig | 1962 | AF301541 | V | [Bastos et al., 2003](file:///C:\Users\Cgallardo\Dropbox\Carmina%20AGOSTO%202014\SECUENCIAS%20CRL\Desktop\Carmina%20MARZO%202014\ARTICULOS\VPPA\GENOTIPADO\2003%20Bastos%20et%20al.pdf) | ATGCAGCCTACTCACCACGCAGAGATAAGCTTTCAGGATAGAGATACAGCTCTTCCAGACGCATGTTCATCTATATCTGATATTAGCCCCGTCACGTATCCGATTACATTACCTATTATTAAAAACATTTCCGTAACTGCTCATGGTATCAATCTTATCGATAAATTTCCATCAAAGTTCTGCAGCTCTTACATACCTTTCCACTACGGAGGCAATGCGATTAAAACCCCCGATGATCCGGGTGCGATGATGATTACCTTTGCCTTGAAGCCACGGGAGGAATACCAACCCAGTGGTCATATTAACGTATCTAGAGCAAGAGAATTTTATATTAGTTGGGATACGGATTACGTGGGGTCTATCACTACGGCTGATCTTGTGGTATCAGCATCTGCTATTAACTT | AY538727 | [Bastos et al., 2004](file:///C:\Users\Cgallardo\Dropbox\Carmina%20AGOSTO%202014\SECUENCIAS%20CRL\Desktop\Carmina%20MARZO%202014\ARTICULOS\VPPA\GENOTIPADO\2004%20Bastos%20et%20al.pdf) | AGTGCGTATACTTGTGCAAGCACTTGTGCAGATACCAATGTAGACACTTGTGCAGACACTTGTGCAGACACTAATGAAGACACTTGTACAAGCACAGAATATAC |
| 7 | Por63 | Portugal | DP | 1963 |  | I | [Nix et al 2006](http://asf-referencelab.info/asf/images/files/publicaciones/Gallardo-et-al-2009a.pdf) | ATGCAGCCTACTCACCACGCAGAGATAAGCTTTCAGGATAGAGATACAGCTCTTCCAGACGCATGTTCATCTATATCGGATATTAGCCCCGTTACGTATCCGATCACATTACCTATTATTAAAAACATTTCCGTAACTGCTCATGGTATCAATCTTATCGATAAGTTTCCATCAAAGTTCTGCAGCTCTTACATACCCTTCCACTACGGAGGCAATGCAATTAAAACCCCCGATGATCCGGGTGCGATGATGATTACCTTTGCTTTGAAGCCACGGGAGGAATACCAACCCAGTGGTCATATTAACGTATCCAGAGCAAGAGAATTTTATATTAGTTGGGACACGGATTACGTGGGGTCTATCACTACGGCTGATCTTGTGGTATCGGCATCTGCTATTAACTT | AM259388 | [Nix et al 2006](http://asf-referencelab.info/asf/images/files/publicaciones/Nix_et_al_2006.pdf) | AGTGCGTATACCTGTGCAAGCACTTGTGCAGATACCAATGTAGACACCTGTGCAAGCACTTGTGCAAGCACTTGTGCAAGCACTTGTGCAAGCACTTGTGCAAGCACTTGTGCAAGCACTTGTGCAAGCACAGGTGCAAGCACTTGTGCAGATACCAATGTAGACACCTGTGCAAGCACTTGTGCAGATACCAATGTAGACACCTGTGCAAGCACTTGTGCAGATACCAATGTAGACACCTGTGCAAGCACTTGTGCAGATACCAATGTAAACACTTGTGCAAGCATGTGTGCAGATACCAATGTAGACACCTGTGCAAGCACCTGTGCAAACACCTGTGCAAGCACAGAATAC |
| 8 | Fr64 | Francia | DP | 1964 | FJ174374 | I | [Gallardo et al., 2009](http://asf-referencelab.info/asf/images/files/publicaciones/Gallardo-et-al-2009a.pdf) | ATGCAGCCTACTCACCACGCAGAGATAAGCTTTCAGGATAGAGATACAGCTCTTCCAGACGCATGTTCATCTATATCGGATATTAGCCCCGTTACGTATCCGATCACATTACCTATTATTAAAAACATTTCCGTAACTGCTCATGGTATCAATCTTATCGATAAGTTTCCATCAAAGTTCTGCAGCTCTTACATACCCTTCCACTACGGAGGCAATGCAATTAAAACCCCCGATGATCCGGGTGCGATGATGATTACCTTTGCTTTGAAGCCACGGGAGGAATACCAACCCAGTGGTCATATTAACGTATCCAGAGCAAGAGAATTTTATATTAGTTGGGACACGGATTACGTGGGGTCTATCACTACGGCTGATCTTGTGGTATCGGCATCTGCTATTAACTT | AM259432 | [Nix et al 2006](http://asf-referencelab.info/asf/images/files/publicaciones/Nix_et_al_2006.pdf) | AGTGCGTATACCTGTGCAAGCACTTGTGCAGATACCAATGTAGACACCTGTGCAAGCACTTGTGCAAGCACTTGTGCAAGCACTTGTGCAAGCACAGGTGCAAGCACTTGTGCAGATACCAATGTAGACACCTGTGCAAGCACTTGTGCAGATACCAATGTAGACACCTGTGCAAGCACTTGTGCAGATACCAATGTAGACACCTGTGCAAGCACTTGTGCAGATACCAATGTAAACACTTGTGCAAGCATGTGTGCAGATACCAATGTAGACACCTGTGCAAGCACCTGTGCAAACACCTGTGCAAGCACAGAATAC |
| 9 | MOZ/1960 | Mozambique | DP | 1964 | AF270708 | V | [Bastos et al., 2004](file:///C:\Users\Cgallardo\Dropbox\Carmina%20AGOSTO%202014\SECUENCIAS%20CRL\Desktop\Carmina%20MARZO%202014\ARTICULOS\VPPA\GENOTIPADO\2004%20Bastos%20et%20al.pdf) | ATGCAGCCTACTCACCACGCAGAGATAAGCTTTCAGGATAGAGATACAGCTCTTCCAGACGCATGTTCATCTATATCTGATATTAGCCCCGTCACGTATCCGATTACATTACCTATTATTAAAAACATTTCCGTAACTGCTCATGGTATCAATCTTATCGATAAATTTCCATCAAAGTTCTGCAGCTCTTACATACCTTTCCACTACGGAGGCAATGCGATTAAAACCCCCGATGATCCGGGTGCAATGATGATTACCTTTGCCTTGAAGCCACGGGAGGAATACCAACCCAGTGGTCATATTAACGTATCTAGAGCAAGAGAATTTTATATTAGTTGGGATACGGATTACGTGGGGTCTATCACTACGGCTGATCTTGTGGTATCGGCATCTGCTATTAACTT | AY274465 | [Heath,L.E (Unpublished OVI](http://asf-referencelab.info/asf/images/files/publicaciones/Nix_et_al_2006.pdf) | AGTGCGTATACTTGTGCAAGCACTTGTGCAGATACCAATGTAGACACTTGTGCAAGCACTTGTGCAAGCACTTGTGCAAGCACTTGTGCAAGCACTTGTACAAGCACTTGTGCAGATACCAATGTAGACACTTGTGCAGATACCAATGTAGACACTTGTGCAGACACTTGTGCAAGCACTTGTGCAGATACCAATGTAGACACTTGTGCAGACACTTGTGCAAGCACTTGTGCAGACACTAATGAAGACACTTGTGCAAGCACTTGTACAAGCACAGAATAC |
| 10 | Moz64 | Mozambique | DP | 1964 | FJ174376 | V | [Gallardo et al., 2009](http://asf-referencelab.info/asf/images/files/publicaciones/Gallardo-et-al-2009a.pdf) | ATGCAGCCTACTCACCACGCAGAGATAAGCTTTCAGGATAGAGATACAGCTCTTCCAGACGCATGTTCATCTATATCTGATATTAGCCCCGTCACGTATCCGATTACATTACCTATTATTAAAAACATTTCCGTAACTGCTCATGGTATCAATCTTATCGATAAATTTCCATCAAAGTTCTGCAGCTCTTACATACCTTTCCACTACGGAGGCAATGCGATTAAAACCCCCGATGATCCGGGTGCAATGATGATTACCTTTGCCTTGAAGCCACGGGAGGAATACCAACCCAGTGGTCATATTAACGTATCTAGAGCAAGAGAATTTTATATTAGTTGGGATACGGATTACGTGGGGTCTATCACTACGGCTGATCTTGTGGTATCGGCATCTGCTATTAACTT | AM259465 | [Nix et al 2006](http://asf-referencelab.info/asf/images/files/publicaciones/Nix_et_al_2006.pdf) | AGTGCGTATACCTGTGCAAGCACTTGTGCAGATACCAATGTAGACACTTGTGCAAGCACTTGTGCAAGCACTTGTGCAAGCACTTGTACAAGCACTTGTGCAGATACCAATGTAGACACTTGTGCAGATACCAATGTAGACACTTGTGCAGACACTTGTGCAAGCACTTGTGCAGATACCAATGTAGACACTTGTGCAGACACTTGTGCAAGCACTTGTGCAGACACTAATGAAGACACTTGTGCAAGCACTTGTACAAGCACAGAATAC |
| 11 | Kat67 | Democratic Republic Congo | DP | 1967 | FJ174377 | I | [Gallardo et al., 2009](http://asf-referencelab.info/asf/images/files/publicaciones/Gallardo-et-al-2009a.pdf) | ATGCAGCCTACTCACCACGCAGAGATAAGCTTTCAGGATAGAGATACAGCTCTTCCAGACGCATGTTCATCTATATCGGATATTAGCCCCGTTACGTATCCGATCACATTACCTATTATTAAAAACATTTCCGTAACTGCTCATGGTATCAATCTTATCGATAAGTTTCCATCAAAGTTCTGCAGCTCTTACATACCCTTCCACTACGGAGGCAATGCAATTAAAACCCCCGATGATCCGGGTGCGATGATGATTACCTTTGCTTTGAAGCCACGGGAGGAATACCAACCCAGTGGTCATATTAACGTATCCAGAGCAAGAGAATTTTATATTAGTTGGGACACGGATTACGTGGGGTCTATCACTACGGCTGATCTTGTGGTATCGGCATCTGCTATTAACTT | AM259461 | [Nix et al 2006](http://asf-referencelab.info/asf/images/files/publicaciones/Nix_et_al_2006.pdf) | AGTGCGTATACCTGTGCAAGCACTTGTGCAAGCACTTGTGCAAGCACTTGTGCAAGCACTTGTGCAAGCACTTGTGCAAGCACTTGTGCAAGCACTTGTGCAAGCACTTGTGCAGATACCAATGTAGACACCTGTGCAAGCACTTGTGCAGATACCAATGTAAACACTTGTGCAAGCATGTGTGCAGATACCAATGTAGACACCTGTGCAAGCACCTGTGCAAGCACTTGTGCAAGCACTTGTGCAAGCACTTGTGCAAGCACTTGTGCAAGCACTTGTGCAAGCACAGAATAC |
| 12 | Co68 | Spain | DP | 1968 | FJ238538 | I | [Gallardo et al., 2009](http://asf-referencelab.info/asf/images/files/publicaciones/Gallardo-et-al-2009a.pdf) | ATGCAGCCTACTCACCACGCAGAGATAAGCTTTCAGGATAGAGATACAGCTCTTCCAGACGCATGTTCATCTATATCGGATATTAGCCCCGTTACGTATCCGATCACATTACCTATTATTAAAAACATTTCCGTAACTGCTCATGGTATCAATCTTATCGATAAGTTTCCATCAAAGTTCTGCAGCTCTTACATACCCTTCCACTACGGAGGCAATGCAATTAAAACCCCCGATGATCCGGGTGCGATGATGATTACCTTTGCTTTGAAGCCACGGGAGGAATACCAACCCAGTGGTCATATTAACGTATCCAGAGCAAGAGAATTTTATATTAGTTGGGACACGGATTACGTGGGGTCTATCACTACGGCTGATCTTGTGGTATCGGCATCTGCTATTAACTT | AM259451 | [Nix et al 2006](http://asf-referencelab.info/asf/images/files/publicaciones/Nix_et_al_2006.pdf) | AGTGCGTATACCTGTGCAAGCACTTGTGCAGATACCAATGTAGACACCTGTGCAAGCACTTGTGCAAGCACTTGTGCAAGCACTTGTGCAAGCACTTGTGCAAGCACAGGTGCAAGCACTTGTGCAGATACCAATGTAGACACCTGTGCAAGCACTTGTGCAGATACCAATGTAGACACCTGTGCAAGCACTTGTGCAGATACCAATGTAGACACCTGTGCAAGCACTTGTGCAGATACCAATGTAAACACTTGTGCAAGCATGTGTGCAGATACCAATGTAGACACCTGTGCAAGCACCTGTGCAAACACCTGTGCAAGCACAGAATAC |
| 13 | NH/P68 | Portugal | DP | 1968 | DQ028313. | I | [Duarte et al 2005 (Unpublished)](http://asf-referencelab.info/asf/images/files/publicaciones/Gallardo-et-al-2009a.pdf) | ATGCAGCCTACTCACCACGCAGAGATAAGCTTTCAGGATAGAGATACAGCTCTTCCAGACGCATGTTCATCTATATCGGATATTAGCCCCGTTACGTATCCGATCACATTACCTATTATTAAAAACATTTCCGTAACTGCTCATGGTATCAATCTTATCGATAAGTTTCCATCAAAGTTCTGCAGCTCTTACATACCCTTCCACTACGGAGGCAATGCAATTAAAACCCCCGATGATCCGGGTGCGATGATGATTACCTTTGCTTTGAAGCCACGGGAGGAATACCAACCCAGTGGTCATATTAACGTATCCAGAGCAAGAGAATTTTATATTAGTTGGGACACGGATTACGTGGGGTCTATCACTACGGCTGATCTTGTGGTATCGGCATCTGCTATTAACTT | DQ028330 | [Duarte et al 2005 (Unpublished)](http://asf-referencelab.info/asf/images/files/publicaciones/Nix_et_al_2006.pdf) | AGTGCGTATACCTGTGCAAGCACTTGTGCAGATACCAATGTAGACACTTGTGCAAGCACTTGTGCAAGCACTTGTGCAAGCACTTGTGCAAGCACTTGTGCAAGCACTTGTGCAAGCACTTGTGCAAGCACTTGTGCAAGCACCTGTGCAAGCACTTGTGCAAGCACTTGTGCAAGCACTTGTGCAAGCACTTGTGCAAGCACAGGTGCAAGCACTTGTGCAGATACCAATGTAGACACCTGTGCAAGCACTTGTGCAGATACCAATGTAGACACCTGTGCAAGCACTTGTGCAGATACCAATGTAGACACCTGTGCAAGCACTTGTGCAGATACCAATGTAAACACTTGTGCAAGCATGTGTGCAGATACCAATGTAGACACCTGTGCAAGCACCTGTGCAAACACCTGTGCAAGCACAGAATAC |
| 14 | 646 | Spain | DP | 1969 | FJ174351 | I | [Gallardo et al., 2009](http://asf-referencelab.info/asf/images/files/publicaciones/Gallardo-et-al-2009a.pdf) | ATGCAGCCTACTCACCACGCAGAGATAAGCTTTCAGGATAGAGATACAGCTCTTCCAGACGCATGTTCATCTATATCGGATATTAGCCCCGTTACGTATCCGATCACATTACCTATTATTAAAAACATTTCCGTAACTGCTCATGGTATCAATCTTATCGATAAGTTTCCATCAAAGTTCTGCAGCTCTTACATACCCTTCCACTACGGAGGCAATGCAATTAAAACCCCCGATGATCCGGGTGCGATGATGATTACCTTTGCTTTGAAGCCACGGGAGGAATACCAACCCAGTGGTCATATTAACGTATCCAGAGCAAGAGAATTTTATATTAGTTGGGACACGGATTACGTGGGGTCTATCACTACGGCTGATCTTGTGGTATCGGCATCTGCTATTAACTT | AM259444 | [Nix et al 2006](http://asf-referencelab.info/asf/images/files/publicaciones/Nix_et_al_2006.pdf) | AGTGCGTATACCTGTGCAAGCACTTGTGCAGATACCAATGTAGACACCTGTGCAAGCACTTGTGCAAGCACTTGTGCAAGCACTTGTGCAAGCACAGGTGCAAGCACTTGTGCAGATACCAATGTAGACACCTGTGCAAGCACTTGTGCAGATACCAATGTAGACACCTGTGCAAGCACTTGTGCAGATACCAATGTAGACACCTGTGCAAGCACTTGTGCAGATACCAATGTAAACACTTGTGCAAGCATGTGTGCAGATACCAATGTAGACACCTGTGCAAGCACCTGTGCAAACACCTGTGCAAGCACAGAATAC |
| 15 | Av71 | Spain | DP | 1971 | FJ174349 | I | [Gallardo et al., 2009](http://asf-referencelab.info/asf/images/files/publicaciones/Gallardo-et-al-2009a.pdf) | ATGCAGCCTACTCACCACGCAGAGATAAGCTTTCAGGATAGAGATACAGCTCTTCCAGACGCATGTTCATCTATATCGGATATTAGCCCCGTTACGTATCCGATCACATTACCTATTATTAAAAACATTTCCGTAACTGCTCATGGTATCAATCTTATCGATAAGTTTCCATCAAAGTTCTGCAGCTCTTACATACCCTTCCACTACGGAGGCAATGCAATTAAAACCCCCGATGATCCGGGTGCGATGATGATTACCTTTGCTTTGAAGCCACGGGAGGAATACCAACCCAGTGGTCATATTAACGTATCCAGAGCAAGAGAATTTTATATTAGTTGGGACACGGATTACGTGGGGTCTATCACTACGGCTGATCTTGTGGTATCGGCATCTGCTATTAACTT | AM25944 | [Nix et al 2006](http://asf-referencelab.info/asf/images/files/publicaciones/Nix_et_al_2006.pdf) | AGTGCGTATACCTGTGCAAGCACTTGTGCAGATACCAATGTAGACACCTGTGCAAGCACTTGTGCAAGCACTTGTGCAAGCACTTGTGCAAGCACAGGTGCAAGCACTTGTGCAGATACCAATGTAGACACCTGTGCAAGCACTTGTGCAGATACCAATGTAGACACCTGTGCAAGCACTTGTGCAGATACCAATGTAGACACCTGTGCAAGCACTTGTGCAGATACCAATGTAAACACTTGTGCAAGCATGTGTGCAGATACCAATGTAGACACCTGTGCAAGCACCTGTGCAAACACCTGTGCAAGCACAGAATAC |
| 16 | Ang72 | Angola | DP | 1972 | FJ174378 | I | [Gallardo et al., 2009](http://asf-referencelab.info/asf/images/files/publicaciones/Gallardo-et-al-2009a.pdf) | ATGCAGCCTACTCACCACGCAGAGATAAGCTTTCAGGATAGAGATACAGCTCTTCCAGACGCATGTTCATCTATATCGGATATTAGCCCCGTTACGTATCCGATCACATTACCTATTATTAAAAACATTTCCGTAACTGCTCATGGTATCAATCTTATCGATAAGTTTCCATCAAAGTTCTGCAGCTCTTACATACCCTTCCACTACGGAGGCAATGCAATTAAAACCCCCGATGATCCGGGTGCGATGATGATTACCTTTGCTTTGAAGCCACGGGAGGAATACCAACCCAGTGGTCATATTAACGTATCCAGAGCAAGAGAATTTTATATTAGTTGGGACACGGATTACGTGGGGTCTATCACTACGGCTGATCTTGTGGTATCGGCATCTGCTATTAACTT | AM259410 | [Nix et al 2006](http://asf-referencelab.info/asf/images/files/publicaciones/Nix_et_al_2006.pdf) | AGTGCGTATACCTGTGCAAGCACTTGTGCAAGCACTTGTGCAGATACCAATGTAGACACCTGTGCAAGCACTTGTGCAGATACCAATGTAAACACTTGTGCAAGCATGTGTGCAGATACCAATGTAGACACCTGTGCAAGCACCTGTGCAAGCACTTGTGCAAGCACTTGTGCAAGCACAGAATAC |
| 17 | B74 | Spain | DP | 1974 | FJ174350 | I | [Gallardo et al., 2009](http://asf-referencelab.info/asf/images/files/publicaciones/Gallardo-et-al-2009a.pdf) | ATGCAGCCTACTCACCACGCAGAGATAAGCTTTCAGGATAGAGATACAGCTCTTCCAGACGCATGTTCATCTATATCGGATATTAGCCCCGTTACGTATCCGATCACATTACCTATTATTAAAAACATTTCCGTAACTGCTCATGGTATCAATCTTATCGATAAGTTTCCATCAAAGTTCTGCAGCTCTTACATACCCTTCCACTACGGAGGCAATGCAATTAAAACCCCCGATGATCCGGGTGCGATGATGATTACCTTTGCTTTGAAGCCACGGGAGGAATACCAACCCAGTGGTCATATTAACGTATCCAGAGCAAGAGAATTTTATATTAGTTGGGACACGGATTACGTGGGGTCTATCACTACGGCTGATCTTGTGGTATCGGCATCTGCTATTAACTT | AM259442 | [Nix et al 2006](http://asf-referencelab.info/asf/images/files/publicaciones/Nix_et_al_2006.pdf) | AGTGCGTATACCTGTGCAAGCACTTGTGCAGATACCAATGTAGACACCTGTGCAAGCACTTGTGCAAGCACTTGTGCAAGCACTTGTGCAAGCACAGGTGCAAGCACTTGTGCAGATACCAATGTAGACACCTGTGCAAGCACTTGTGCAGATACCAATGTAGACACCTGTGCAAGCACTTGTGCAGATACCAATGTAGACACCTGTGCAAGCACTTGTGCAGATACCAATGTAAACACTTGTGCAAGCATGTGTGCAGATACCAATGTAGACACCTGTGCAAGCACCTGTGCAAACACCTGTGCAAGCACAGAATAC |
| 18 | E75 | Spain | DP | 1975 | AY578693 | I | [De Villiers et al 2010](http://asf-referencelab.info/asf/images/files/publicaciones/Gallardo-et-al-2009a.pdf) | ATGCAGCCTACTCACCACGCAGAGATAAGCTTTCAGGATAGAGATACAGCTCTTCCAGACGCATGTTCATCTATATCGGATATTAGCCCCGTTACGTATCCGATCACATTACCTATTATTAAAAACATTTCCGTAACTGCTCATGGTATCAATCTTATCGATAAGTTTCCATCAAAGTTCTGCAGCTCTTACATACCCTTCCACTACGGAGGCAATGCAATTAAAACCCCCGATGATCCGGGTGCGATGATGATTACCTTTGCTTTGAAGCCACGGGAGGAATACCAACCCAGTGGTCATATTAACGTATCCAGAGCAAGAGAATTTTATATTAGTTGGGACACGGATTACGTGGGGTCTATCACTACGGCTGATCTTGTGGTATCGGCATCTGCTATTAACTT | AM259450 | [Nix et al 2006](http://asf-referencelab.info/asf/images/files/publicaciones/Nix_et_al_2006.pdf) | AGTGCGTATACCTGTGCAAGCACTTGTGCAGATACCAATGTAGACACCTGTGCAAGCACTTGTGCAAGCACTTGTGCAAGCACTTGTGCAAGCACAGGTGCAAGCACTTGTGCAGATACCAATGTAGACACCTGTGCAAGCACTTGTGCAGATACCAATGTAGACACCTGTGCAAGCACTTGTGCAGATACCAATGTAGACACCTGTGCAAGCACTTGTGCAGATACCAATGTAAACACTTGTGCAAGCATGTGTGCAGATACCAATGTAGACACCTGTGCAAGCACCTGTGCAAACACCTGTGCAAGCACAGAATAC |
| 19 | 56/Ca/78 | Italy | DP | 1978 | FR686761 | I | [Giammarioli et al 2011](http://asf-referencelab.info/asf/images/files/publicaciones/Nix_et_al_2006.pdf) | ATGCAGCCTACTCACCACGCAGAGATAAGCTTTCAGGATAGAGATACAGCTCTTCCAGACGCATGTTCATCTATATCGGATATTAGCCCCGTTACGTATCCGATCACATTACCTATTATTAAAAACATTTCCGTAACTGCTCATGGTATCAATCTTATCGATAAGTTTCCATCAAAGTTCTGCAGCTCTTACATACCCTTCCACTACGGAGGCAATGCAATTAAAACCCCCGATGATCCGGGTGCGATGATGATTACCTTTGCTTTGAAGCCACGGGAGGAATACCAACCCAGTGGTCATATTAACGTATCCAGAGCAAGAGAATTTTATATTAGTTGGGACACGGATTACGTGGGGTCTATCACTACGGCTGATCTTGTGGTATCGGCATCTGCTATTAACTT | FR686765 | [Giammarioli et al 2011](http://asf-referencelab.info/asf/images/files/publicaciones/Nix_et_al_2006.pdf) | AGTGCGTATACCTGTGCAAGCACTTGTGCAGATACCAATGTAGACACCTGTGCAAGCACTTGTGCAAGCACTTGTGCAAGCACTTGTGCAAGCACAGGTGCAAGCACTTGTGCAGATACCAATGTAGACACCTGTGCAAGCACTTGTGCAGATACCAATGTAGACACCTGTGCAAGCACTTGTGCAGATACCAATGTAGACACCTGTGCAAGCACTTGTGCAGATACCAATGTAAACACTTGTGCAAGCATGTGTGCAGATACCAATGTAGACACCTGTGCAAGCACCTGTGCAAACACCTGTGCAAGCACAGAATAC |
| 20 | Ca78 | Italy | DP | 1978 | FJ174357 | I | [Gallardo et al., 2009](http://asf-referencelab.info/asf/images/files/publicaciones/Gallardo-et-al-2009a.pdf) | ATGCAGCCTACTCACCACGCAGAGATAAGCTTTCAGGATAGAGATACAGCTCTTCCAGACGCATGTTCATCTATATCGGATATTAGCCCCGTTACGTATCCGATCACATTACCTATTATTAAAAACATTTCCGTAACTGCTCATGGTATCAATCTTATCGATAAGTTTCCATCAAAGTTCTGCAGCTCTTACATACCCTTCCACTACGGAGGCAATGCAATTAAAACCCCCGATGATCCGGGTGCGATGATGATTACCTTTGCTTTGAAGCCACGGGAGGAATACCAACCCAGTGGTCATATTAACGTATCCAGAGCAAGAGAATTTTATATTAGTTGGGACACGGATTACGTGGGGTCTATCACTACGGCTGATCTTGTGGTATCGGCATCTGCTATTAACTT | AM259433 | [Nix et al 2006](http://asf-referencelab.info/asf/images/files/publicaciones/Nix_et_al_2006.pdf) | AGTGCGTATACCTGTGCAAGCACTTGTGCAGATACCAATGTAGACACCTGTGCAAGCACTTGTGCAAGCACTTGTGCAAGCACTTGTGCAAGCACAGGTGCAAGCACTTGTGCAGATACCAATGTAGACACCTGTGCAAGCACTTGTGCAGATACCAATGTAGACACCTGTGCAAGCACTTGTGCAGATACCAATGTAGACACCTGTGCAAGCACTTGTGCAGATACCAATGTAAACACTTGTGCAAGCATGTGTGCAGATACCAATGTAGACACCTGTGCAAGCACCTGTGCAAACACCTGTGCAAGCACAGAATAC |
| 21 | MAL/1978 | Malawi | DP | 1978 | AF270707 | VIII | [Bastos et al., 2003](http://asf-referencelab.info/asf/images/files/publicaciones/Gallardo-et-al-2009a.pdf) | ATGCAGCCTACTCACCACGCAGAAATAAGCTTTCAGGATAGAGATACAGCTCTTCCAGACGCATGTTCATCTATATCTGATATTAACCCCGTTACTTATCCGATCACATTACCTATTATTAAAAACATTTCCGTAACTGCTCATGGTATCAATCTTATCGATAAGTTTCCATCAAAGTTCTGCAGCTCTTACATACCCTTCCACTATGGAGGCAATTCGATTAAAACCCCTGATGATCCGGGCGCGATGATGATTACCTTCGCTTTGAAGCCACGGGAGGAATACCAACCCAGTGGTCATATTAACGTATCCAGAGCAAGAGAATTTTATATTAGTTGGGACACGGATTATGTGGGATCTATTACCACGGCTGATCTTGTGGTATCAGCATCTGCTATTAACTT | AY274466 | [Bastos et al., 2004](http://asf-referencelab.info/asf/images/files/publicaciones/Nix_et_al_2006.pdf) | AGTGCGTATACCTGTGCAAGCACCAATGCAGACACAAGTGCAAGCACCAATGCAGACACAAGTGCAAGCACCAATGCAAGCATAAATGCAGATACCAATGTAGATACTTGTGCAAGCACCAATGCAAGCACAAATGTAGACACCAATGCAAGCATAAATGCAAGCACCAATGCAAGCACAAATGTAGACACCAATGCAAGCACCAATGCAGACATCAATGCAAACACCAATGCAGACATCAATGCAAACATCAATGCAAACACAGAATAC |
| 22 | 57/Ca/79 | Italy | DP | 1979 | FR686762 | I | [Giammarioli et al 2011](http://asf-referencelab.info/asf/images/files/publicaciones/Gallardo-et-al-2009a.pdf) | ATGCAGCCTACTCACCACGCAGAGATAAGCTTTCAGGATAGAGATACAGCTCTTCCAGACGCATGTTCATCTATATCGGATATTAGCCCCGTTACGTATCCGATCACATTACCTATTATTAAAAACATTTCCGTAACTGCTCATGGTATCAATCTTATCGATAAGTTTCCATCAAAGTTCTGCAGCTCTTACATACCCTTCCACTACGGAGGCAATGCAATTAAAACCCCCGATGATCCGGGTGCGATGATGATTACCTTTGCTTTGAAGCCACGGGAGGAATACCAACCCAGTGGTCATATTAACGTATCCAGAGCAAGAGAATTTTATATTAGTTGGGACACGGATTACGTGGGGTCTATCACTACGGCTGATCTTGTGGTATCGGCATCTGCTATTAACTT | FR686766 | [Giammarioli et al 2011](http://asf-referencelab.info/asf/images/files/publicaciones/Nix_et_al_2006.pdf) | AGTGCGTATACCTGTGCAAGCACTTGTGCAGATACCAATGTAGACACCTGTGCAAGCACTTGTGCAAGCACTTGTGCAAGCACTTGTGCAAGCACAGGTGCAAGCACTTGTGCAGATACCAATGTAGACACCTGTGCAAGCACTTGTGCAGATACCAATGTAGACACCTGTGCAAGCACTTGTGCAGATACCAATGTAGACACCTGTGCAAGCACTTGTGCAGATACCAATGTAAACACTTGTGCAAGCATGTGTGCAGATACCAATGTAGACACCTGTGCAAGCACCTGTGCAAACACCTGTGCAAGCACAGAATAC |
| 23 | Nu81 | Italy | DP | 1981 | FJ174358 | I | [Gallardo et al., 2009](http://asf-referencelab.info/asf/images/files/publicaciones/Gallardo-et-al-2009a.pdf) | ATGCAGCCTACTCACCACGCAGAGATAAGCTTTCAGGATAGAGATACAGCTCTTCCAGACGCATGTTCATCTATATCGGATATTAGCCCCGTTACGTATCCGATCACATTACCTATTATTAAAAACATTTCCGTAACTGCTCATGGTATCAATCTTATCGATAAGTTTCCATCAAAGTTCTGCAGCTCTTACATACCCTTCCACTACGGAGGCAATGCAATTAAAACCCCCGATGATCCGGGTGCGATGATGATTACCTTTGCTTTGAAGCCACGGGAGGAATACCAACCCAGTGGTCATATTAACGTATCCAGAGCAAGAGAATTTTATATTAGTTGGGACACGGATTACGTGGGGTCTATCACTACGGCTGATCTTGTGGTATCGGCATCTGCTATTAACTT | AM259435 | [Nix et al 2006](http://asf-referencelab.info/asf/images/files/publicaciones/Nix_et_al_2006.pdf) | AGTGCGTATACCTGTGCAAGCACTTGTGCAGATACCAATGTAGACACCTGTGCAAGCACTTGTGCAAGCACTTGTGCAAGCACTTGTGCAAGCACAGGTGCAAGCACTTGTGCAGATACCAATGTAGACACCTGTGCAAGCACTTGTGCAGATACCAATGTAGACACCTGTGCAAGCACTTGTGCAGATACCAATGTAGACACCTGTGCAAGCACTTGTGCAGATACCAATGTAAACACTTGTGCAAGCATGTGTGCAGATACCAATGTAGACACCTGTGCAAGCACCTGTGCAAACACCTGTGCAAGCACAGAATAC |
| 24 | SS81 | Italy | DP | 1981 | FJ174359 | I | [Gallardo et al., 2009](http://asf-referencelab.info/asf/images/files/publicaciones/Gallardo-et-al-2009a.pdf) | ATGCAGCCTACTCACCACGCAGAGATAAGCTTTCAGGATAGAGATACAGCTCTTCCAGACGCATGTTCATCTATATCGGATATTAGCCCCGTTACGTATCCGATCACATTACCTATTATTAAAAACATTTCCGTAACTGCTCATGGTATCAATCTTATCGATAAGTTTCCATCAAAGTTCTGCAGCTCTTACATACCCTTCCACTACGGAGGCAATGCAATTAAAACCCCCGATGATCCGGGTGCGATGATGATTACCTTTGCTTTGAAGCCACGGGAGGAATACCAACCCAGTGGTCATATTAACGTATCCAGAGCAAGAGAATTTTATATTAGTTGGGACACGGATTACGTGGGGTCTATCACTACGGCTGATCTTGTGGTATCGGCATCTGCTATTAACTT | AM259436 | [Nix et al 2006](http://asf-referencelab.info/asf/images/files/publicaciones/Nix_et_al_2006.pdf) | AGTGCGTATACCTGTGCAAGCACTTGTGCAGATACCAATGTAGACACCTGTGCAAGCACTTGTGCAAGCACTTGTGCAAGCACTTGTGCAAGCACAGGTGCAAGCACTTGTGCAGATACCAATGTAGACACCTGTGCAAGCACTTGTGCAGATACCAATGTAGACACCTGTGCAAGCACTTGTGCAGATACCAATGTAGACACCTGTGCAAGCACTTGTGCAGATACCAATGTAAACACTTGTGCAAGCATGTGTGCAGATACCAATGTAGACACCTGTGCAAGCACCTGTGCAAACACCTGTGCAAGCACAGAATAC |
| 25 | Mu82 | Spain | DP | 1982 | FJ174352 | I | [Gallardo et al., 2009](http://asf-referencelab.info/asf/images/files/publicaciones/Gallardo-et-al-2009a.pdf) | ATGCAGCCTACTCACCACGCAGAGATAAGCTTTCAGGATAGAGATACAGCTCTTCCAGACGCATGTTCATCTATATCGGATATTAGCCCCGTTACGTATCCGATCACATTACCTATTATTAAAAACATTTCCGTAACTGCTCATGGTATCAATCTTATCGATAAGTTTCCATCAAAGTTCTGCAGCTCTTACATACCCTTCCACTACGGAGGCAATGCAATTAAAACCCCCGATGATCCGGGTGCGATGATGATTACCTTTGCTTTGAAGCCACGGGAGGAATACCAACCCAGTGGTCATATTAACGTATCCAGAGCAAGAGAATTTTATATTAGTTGGGACACGGATTACGTGGGGTCTATCACTACGGCTGATCTTGTGGTATCGGCATCTGCTATTAACTT | AM259448 | [Nix et al 2006](http://asf-referencelab.info/asf/images/files/publicaciones/Nix_et_al_2006.pdf) | AGTGCGTATACCTGTGCAAGCACTTGTGCAGATACCAATGTAGACACCTGTGCAAGCACTTGTGCAAGCACTTGTGCAAGCACTTGTGCAAGCACAGGTGCAAGCACTTGTGCAGATACCAATGTAGACACCTGTGCAAGCACTTGTGCAGATACCAATGTAGACACCTGTGCAAGCACTTGTGCAGATACCAATGTAGACACCTGTGCAAGCACTTGTGCAGATACCAATGTAAACACTTGTGCAAGCATGTGTGCAGATACCAATGTAGACACCTGTGCAAGCACCTGTGCAAACACCTGTGCAAGCACAGAATAC |
| 26 | MwLil 20/1 | Malawi | Tick | 1983 | AY261361 | VIII | [Complete genome](http://www.ncbi.nlm.nih.gov/nuccore/AY261361.1) | ATGCAGCCTACTCACCACGCAGAAATAAGCTTTCAGGATAGAGATACAGCTCTTCCAGACGCATGTTCATCTATATCTGATATTAACCCCGTTACTTATCCGATCACATTACCTATTATTAAAAACATTTCCGTAACTGCTCATGGTATCAATCTTATCGATAAGTTTCCATCAAAGTTCTGCAGCTCTTACATACCCTTCCACTATGGAGGCAATTCGATTAAAACCCCTGATGATCCGGGCGCGATGATGATTACCTTCGCTTTGAAGCCACGGGAGGAATACCAACCCAGTGGTCATATTAACGTATCCAGAGCAAGAGAATTTTATATTAGTTGGGACACGGATTATGTGGGATCTATTACCACGGCTGATCTTGTGGTATCAGCATCTGCTATTAACTT | AM259427 | [Nix et al 2006](http://asf-referencelab.info/asf/images/files/publicaciones/Nix_et_al_2006.pdf) | AGTGCGTATACCTGTGCAAGCACCAATGCAGACACAAGTGCAAGCACCAATGCAGACACAAGTGCAAGCACCAATGCAAGCATAAATGCAGATACCAATGTAGATACTTGTGCAAGCACCAATGCAAGCACAAATGTAGACACCAATGCAAGCATAAATGCAAGCACCAATGCAAGCACAAATGTAGACACCAATGCAAGCACCAATGCAAGCATAAATGCAGATACCAATGTAGATACTTGTGCAAGCACCAATGCAAGCACAAATGTAGACACCAATGCAAGCATAAATGCAAGCACCAATGCAAGCACAAATGTAGACACCAATGCAGACATCAATGCAAACACCAATGCAGACATCAATGCAAACATCAATGCAAACACAGAATAC |
| 27 | BUR/1/84 | Burundi | DP | 1984 | AF449463 | X | [Bastos et al., 2003](http://asf-referencelab.info/asf/images/files/publicaciones/Gallardo-et-al-2009a.pdf) | ATGCAGCCTACCCACCACGCAGAGGTAAGCTTTCAGGATAGAGATACAGCTCTTCCAGATGCATGTTCATCCATATCAGATATTTCCCCCATTACTTATCCGATCACGTTACCTATTATTAAAAACATTTCCGTCACTGCTCATGGTATCAATCTTATCGATAAATTTCCATCAAAGTTCTGCAGCTCTTACATACCCTTTCACTACGGAGGCAATTCGATTAAAACCCCCGACGATCCGGGCGCGATGATGATTACCTTTGCTTTGAAACCACGGGAGGAATACCAACCCAGTGGTCATATTAACGTATCCAGAGCAAGAGAGTTTTATATTAGCTGGGACACAGATTATGTGGGGTCTATCACCACGGCCGATCTTGTGGTATCGGCATCCGCTATTAACTT | AM259422 | [Nix et al 2006](http://asf-referencelab.info/asf/images/files/publicaciones/Nix_et_al_2006.pdf) | AGTGCGTATACCTGTGCAAGCACTTGTGCAAGCACTTGTGCAAGCACCTGTGCAGACACCAATGTAGACACTTGTGCAAGCACTTGTGCAAGCACTTGTGCAAGCACTTGTGCAAGCACTTGTGCAAGCACTTGTGCAAGCACTTGTGCAAGCACTTGTGCAAGCACTTGTGCAAGCACTTGTGCAAGCACTTGTGCAGACACCTGTGCAAGCACAGAATAC |
| 28 | BUR/2/84 | Burundi | DP | 1984 | AF449464 | X | [Bastos et al., 2003](http://asf-referencelab.info/asf/images/files/publicaciones/Gallardo-et-al-2009a.pdf) | ATGCAGCCTACCCACCACGCAGAGGTAAGCTTTCAGGATAGAGATACAGCTCTTCCAGATGCATGTTCATCCATATCAGATATTTCCCCCATTACTTATCCGATCACGTTACCTATTATTAAAAACATTTCCGTCACTGCTCATGGTATCAATCTTATCGATAAATTTCCATCAAAGTTCTGCAGCTCTTACATACCCTTTCACTACGGAGGCAATTCGATTAAAACCCCCGACGATCCGGGCGCGATGATGATTACCTTTGCTTTGAAACCACGGGAGGAATACCAACCCAGTGGTCATATTAACGTATCCAGAGCAAGAGAGTTTTATATTAGCTGGGACACAGATTATGTGGGGTCTATCACCACGGCCGATCTTGTGGTATCGGCATCCGCTATTAACTT | AM259423 | [Nix et al 2006](http://asf-referencelab.info/asf/images/files/publicaciones/Nix_et_al_2006.pdf) | AGTGCGTATACCTGTGCAAGCACTTGTGCAAGCACTTGTGCAAGCACCTGTGCAGACACCAATGTAGACACTTGTGCAAGCACTTGTGCAAGCACTTGTGCAAGCACTTGTGCAAGCACTTGTGCAAGCACTTGTGCAAGCACTTGTGCAAGCACTTGTGCAAGCACTTGTGCAAGCACTTGTGCAAGCACTTGTGCAGACACCTGTGCAAGCACAGAATAC |
| 29 | Mon84 | Portugal | DP | 1984 |  | I | [Nix et al 2006](http://asf-referencelab.info/asf/images/files/publicaciones/Gallardo-et-al-2009a.pdf) | ATGCAGCCTACTCACCACGCAGAGATAAGCTTTCAGGATAGAGATACAGCTCTTCCAGACGCATGTTCATCTATATCGGATATTAGCCCCGTTACGTATCCGATCACATTACCTATTATTAAAAACATTTCCGTAACTGCTCATGGTATCAATCTTATCGATAAGTTTCCATCAAAGTTCTGCAGCTCTTACATACCCTTCCACTACGGAGGCAATGCAATTAAAACCCCCGATGATCCGGGTGCGATGATGATTACCTTTGCTTTGAAGCCACGGGAGGAATACCAACCCAGTGGTCATATTAACGTATCCAGAGCAAGAGAATTTTATATTAGTTGGGACACGGATTACGTGGGGTCTATCACTACGGCTGATCTTGTGGTATCGGCATCTGCTATTAACTT | AM259389 | [Nix et al 2006](http://asf-referencelab.info/asf/images/files/publicaciones/Nix_et_al_2006.pdf) | AGTGCGTATACTTGTGCAAGCACTTGTGCAGATACCAATGTAGACACCTGTGCAAGCACTTGTGCAAGCACTTGTGCAAGCACTTGTGCAAGCACAGGTGCAAGCACTTGTGCAGATACCAATGTAGACACCTGTGCAAGCACTTGTGCAGATACCAATGTAGACACCTGTGCAAGCACTTGTGCAGATACCAATGTAGACACCTGTGCAAGCACTTGTGCAGATACCAATGTAAACACTTGTGCAAGCATGTGTGCAGATACCAATGTAGACACCTGTGCAAGCACCTGTGCAAACACCTGTGCAAGCACAGAATAC |
| 30 | Ori84 | Italy | DP | 1984 | FJ174360 | I | [Gallardo et al., 2009](http://asf-referencelab.info/asf/images/files/publicaciones/Gallardo-et-al-2009a.pdf) | ATGCAGCCTACTCACCACGCAGAGATAAGCTTTCAGGATAGAGATACAGCTCTTCCAGACGCATGTTCATCTATATCGGATATTAGCCCCGTTACGTATCCGATCACATTACCTATTATTAAAAACATTTCCGTAACTGCTCATGGTATCAATCTTATCGATAAGTTTCCATCAAAGTTCTGCAGCTCTTACATACCCTTCCACTACGGAGGCAATGCAATTAAAACCCCCGATGATCCGGGTGCGATGATGATTACCTTTGCTTTGAAGCCACGGGAGGAATACCAACCCAGTGGTCATATTAACGTATCCAGAGCAAGAGAATTTTATATTAGTTGGGACACGGATTACGTGGGGTCTATCACTACGGCTGATCTTGTGGTATCGGCATCTGCTATTAACTT | AM259437 | [Nix et al 2006](http://asf-referencelab.info/asf/images/files/publicaciones/Nix_et_al_2006.pdf) | AGTGCGTATACCTGTGCAAGCACTTGTGCAGATACCAATGTAGACACCTGTGCAAGCACTTGTGCAAGCACTTGTGCAAGCACTTGTGCAAGCACAGGTGCAAGCACTTGTGCAGATACCAATGTAGACACCTGTGCAAGCACTTGTGCAGATACCAATGTAGACACCTGTGCAAGCACTTGTGCAGATACCAATGTAGACACCTGTGCAAGCACTTGTGCAGATACCAATGTAAACACTTGTGCAAGCATGTGTGCAGATACCAATGTAGACACCTGTGCAAGCACCTGTGCAAACACCTGTGCAAGCACAGAATAC |
| 31 | ZOM/2/84 | Malawi | DP | 1984 | AF449471 | VIII | [Bastos et al., 2003](http://asf-referencelab.info/asf/images/files/publicaciones/Gallardo-et-al-2009a.pdf) | ATGCAGCCTACTCACCACGCAGAAATAAGCTTTCAGGATAGAGATACAGCTCTTCCAGACGCATGTTCATCTATATCTGATATTAACCCCGTTACTTATCCGATCACATTACCTATTATTAAAAACATTTCCGTAACTGCTCATGGTATCAATCTTATCGATAAGTTTCCATCAAAGTTCTGCAGCTCTTACATACCCTTCCACTATGGAGGCAATTCGATTAAAACCCCTGATGATCCGGGCGCGATGATGATTACCTTCGCTTTGAAGCCACGGGAGGAATACCAACCCAGTGGTCATATTAACGTATCCAGAGCAAGAGAATTTTATATTAGTTGGGACACGGATTATGTGGGATCTATTACCACGGCTGATCTTGTGGTATCAGCATCTGCTATTAACTT | AY538731 | [Bastos et al., 2004](http://asf-referencelab.info/asf/images/files/publicaciones/Nix_et_al_2006.pdf) | AGTGCGTATACCTGTGCAAGCACCAATGCAGACACAAGTGCAAGCACCAATGCAGACACAAGTGCAAGCACCAATGCAAGCATAAATGCAGATACCAATGTAGATACTTGTGCAAGCACCAATGCAAGCACAAATGTAGACACCAATGCAAGCATAAATGCAAGCACCAATGCAAGCACAAATGTAGACACCAATGCAAGCACCAATGCAGACATCAATGCAAACACCAATGCAGACATCAATGCAAACATCAATGCAAACACAGAATAC |
| 32 | Ori85 | Italy | DP | 1985 | FJ174361 | I | [Gallardo et al., 2009](http://asf-referencelab.info/asf/images/files/publicaciones/Gallardo-et-al-2009a.pdf) | ATGCAGCCTACTCACCACGCAGAGATAAGCTTTCAGGATAGAGATACAGCTCTTCCAGACGCATGTTCATCTATATCGGATATTAGCCCCGTTACGTATCCGATCACATTACCTATTATTAAAAACATTTCCGTAACTGCTCATGGTATCAATCTTATCGATAAGTTTCCATCAAAGTTCTGCAGCTCTTACATACCCTTCCACTACGGAGGCAATGCAATTAAAACCCCCGATGATCCGGGTGCGATGATGATTACCTTTGCTTTGAAGCCACGGGAGGAATACCAACCCAGTGGTCATATTAACGTATCCAGAGCAAGAGAATTTTATATTAGTTGGGACACGGATTACGTGGGGTCTATCACTACGGCTGATCTTGTGGTATCGGCATCTGCTATTAACTT | AM259397 | [Nix et al 2006](http://asf-referencelab.info/asf/images/files/publicaciones/Nix_et_al_2006.pdf) | AGTGCGTATACCTGTGCAAGCACTTGTGCAGATACCAATGTAGACACCTGTGCAAGCACTTGTGCAAGCACTTGTGCAAGCACTTGTGCAAGCACAGGTGCAAGCACTTGTGCAGATACCAATGTAGACACCTGTGCAAGCACTTGTGCAGATACCAATGTAGACACCTGTGCAAGCACTTGTGCAGATACCAATGTAGACACCTGTGCAAGCACTTGTGCAGATACCAATGTAAACACTTGTGCAAGCATGTGTGCAGATACCAATGTAGACACCTGTGCAAGCACCTGTGCAAACACCTGTGCAAGCACAGAATAC |
| 33 | Coi86 | Portugal | DP | 1986 |  | I | [Nix et al 2006](http://asf-referencelab.info/asf/images/files/publicaciones/Gallardo-et-al-2009a.pdf) | ATGCAGCCTACTCACCACGCAGAGATAAGCTTTCAGGATAGAGATACAGCTCTTCCAGACGCATGTTCATCTATATCGGATATTAGCCCCGTTACGTATCCGATCACATTACCTATTATTAAAAACATTTCCGTAACTGCTCATGGTATCAATCTTATCGATAAGTTTCCATCAAAGTTCTGCAGCTCTTACATACCCTTCCACTACGGAGGCAATGCAATTAAAACCCCCGATGATCCGGGTGCGATGATGATTACCTTTGCTTTGAAGCCACGGGAGGAATACCAACCCAGTGGTCATATTAACGTATCCAGAGCAAGAGAATTTTATATTAGTTGGGACACGGATTACGTGGGGTCTATCACTACGGCTGATCTTGTGGTATCGGCATCTGCTATTAACTT | AM259392 | [Nix et al 2006](http://asf-referencelab.info/asf/images/files/publicaciones/Nix_et_al_2006.pdf) | AGTGCGTATACTTGTGCAAGCACTTGTGCAGATACCAATGTAGACACCTGTGCAAGCACTTGTGCAAGCACTTGTGCAAGCACTTGTGCAAGCACAGGTGCAAGCACTTGTGCAGATACCAATGTAGACACCTGTGCAAGCACTTGTGCAGATACCAATGTAGACACCTGTGCAAGCACTTGTGCAGATACCAATGTAGACACCTGTGCAAGCACTTGTGCAGATACCAATGTAAACACTTGTGCAAGCATGTGTGCAGATACCAATGTAGACACCTGTGCAAGCACCTGTGCAAACACCTGTGCAAGCACAGAATACCCCGATTTAGCAGAT |
| 34 | Dedzaa | Malawi | DP | 1986 | AF449479 | VIII | [Bastos et al., 2003](http://asf-referencelab.info/asf/images/files/publicaciones/Gallardo-et-al-2009a.pdf) | ATGCAGCCTACTCACCACGCAGAAATAAGCTTTCAGGATAGAGATACAGCTCTTCCAGACGCATGTTCATCTATATCTGATATTAACCCCGTTACTTATCCGATCACATTACCTATTATTAAAAACATTTCCGTAACTGCTCATGGTATCAATCTTATCGATAAGTTTCCATCAAAGTTCTGCAGCTCTTACATACCCTTCCACTATGGAGGCAATTCGATTAAAACCCCTGATGATCCGGGCGCGATGATGATTACCTTCGCTTTGAAGCCACGGGAGGAATACCAACCCAGTGGTCATATTAACGTATCCAGAGCAAGAGAATTTTATATTAGTTGGGACACGGATTATGTGGGATCTATTACCACGGCTGATCTTGTGGTATCAGCATCTGCTATTAACTT | AY538729 | [Bastos et al., 2004](http://asf-referencelab.info/asf/images/files/publicaciones/Nix_et_al_2006.pdf) | AGTGCGTATACCTGTGCAAGCACCAATGCAGACACAAGTGCAAGCACCAATGTAGACACCAATGCAAGCATAAATGCAAGCACCAATGCAAGCACAAATGTAGACACCAATGCAAGCACCAATGCAGACATCAATGCAAACACCAATGCAGACATCAATGCAAACATCAATGCAAACACAGAATAC |
| 35 | MAFRA86 | Portugal | DP | 1986 | DQ028312 | I | [Duarte et al 2005 (Unpublished)](http://asf-referencelab.info/asf/images/files/publicaciones/Gallardo-et-al-2009a.pdf) | ATGCAGCCTACTCACCACGCAGAGATAAGCTTTCAGGATAGAGATACAGCTCTTCCAGACGCATGTTCATCTATATCGGATATTAGCCCCGTTACGTATCCGATCACATTACCTATTATTAAAAACATTTCCGTAACTGCTCATGGTATCAATCTTATCGATAAGTTTCCATCAAAGTTCTGCAGCTCTTACATACCCTTCCACTACGGAGGCAATGCAATTAAAACCCCCGATGATCCGGGTGCGATGATGATTACCTTTGCTTTGAAGCCACGGGAGGAATACCAACCCAGTGGTCATATTAACGTATCCAGAGCAAGAGAATTTTATATTAGTTGGGACACGGATTACGTGGGGTCTATCACTACGGCTGATCTTGTGGTATCGGCATCTGCTATTAACTT | DQ028328 | [Duarte et al 2005 (Unpublished)](http://asf-referencelab.info/asf/images/files/publicaciones/Nix_et_al_2006.pdf) | AGTGCGTATACCTGTGCAAGCACTTGTGCAGATACCAATGTAGACACCTGTGCAAGCACTTGTGCAAGCACTTGTGCAAGCACTTGTGCAAGCACAGGTGCAAGCACTTGTGCAGATACCAATGTAAACACTTGTGCAAGCATGTGTGCAGATACCAATGTAGACACCTGTGCAAGCACTTGTGCAGATACCAATGTAGACACCTGTGCAAGCACTTGTGCAGATACCAATGTAAACACTTGTGCAAGCACTTGTGCAGATACCAATGTAGACACCTGTGCAAGCACCTGTGCAAACACCTGTGCAAGCACAGAATAC |
| 36 | Por86 | Portugal | DP | 1986 |  | I | [Nix et al 2006](http://asf-referencelab.info/asf/images/files/publicaciones/Gallardo-et-al-2009a.pdf) | ATGCAGCCTACTCACCACGCAGAGATAAGCTTTCAGGATAGAGATACAGCTCTTCCAGACGCATGTTCATCTATATCGGATATTAGCCCCGTTACGTATCCGATCACATTACCTATTATTAAAAACATTTCCGTAACTGCTCATGGTATCAATCTTATCGATAAGTTTCCATCAAAGTTCTGCAGCTCTTACATACCCTTCCACTACGGAGGCAATGCAATTAAAACCCCCGATGATCCGGGTGCGATGATGATTACCTTTGCTTTGAAGCCACGGGAGGAATACCAACCCAGTGGTCATATTAACGTATCCAGAGCAAGAGAATTTTATATTAGTTGGGACACGGATTACGTGGGGTCTATCACTACGGCTGATCTTGTGGTATCGGCATCTGCTATTAACTT | AM259391 | [Nix et al 2006](http://asf-referencelab.info/asf/images/files/publicaciones/Nix_et_al_2006.pdf) | AGTGCGTATACTTGTGCAAGCACTTGTGCAGATACCAATGTAGACACCTGTGCAAGCACTTGTGCAAGCACTTGTGCAAGCACTTGTGCAAGCACAGGTGCAAGCACTTGTGCAGATACCAATGTAGACACCTGTGCAAGCACTTGTGCAGATACCAATGTAGACACCTGTGCAAGCACTTGTGCAGATACCAATGTAGACACCTGTGCAAGCACTTGTGCAGATACCAATGTAAACACTTGTGCAAGCATGTGTGCAGATACCAATGTAGACACCTGTGCAAGCACCTGTGCAAACACCTGTGCAAGCACAGAATAC |
| 37 | San 86 | Portugal | DP | 1986 |  | I | [Nix et al 2006](file:///C:\Users\Cgallardo\Dropbox\Carmina%20AGOSTO%202014\SECUENCIAS%20CRL\Desktop\Carmina%20MARZO%202014\ARTICULOS\VPPA\GENOTIPADO\2003%20Bastos%20et%20al.pdf) | ATGCAGCCTACTCACCACGCAGAGATAAGCTTTCAGGATAGAGATACAGCTCTTCCAGACGCATGTTCATCTATATCGGATATTAGCCCCGTTACGTATCCGATCACATTACCTATTATTAAAAACATTTCCGTAACTGCTCATGGTATCAATCTTATCGATAAGTTTCCATCAAAGTTCTGCAGCTCTTACATACCCTTCCACTACGGAGGCAATGCAATTAAAACCCCCGATGATCCGGGTGCGATGATGATTACCTTTGCTTTGAAGCCACGGGAGGAATACCAACCCAGTGGTCATATTAACGTATCCAGAGCAAGAGAATTTTATATTAGTTGGGACACGGATTACGTGGGGTCTATCACTACGGCTGATCTTGTGGTATCGGCATCTGCTATTAACTT | AM259393 | [Nix et al 2006](http://asf-referencelab.info/asf/images/files/publicaciones/Nix_et_al_2006.pdf) | AGTGCGTATACTTGTGCAAGCACTTGTGCAGATACCAATGTAGACACCTGTGCAAGCACTTGTGCAAGCACTTGTGCAAGCACTTGTGCAAGCACAGGTGCAAGCACTTGTGCAGATACCAATGTAGACACCTGTGCAAGCACTTGTGCAGATACCAATGTAGACACCTGTGCAAGCACTTGTGCAGATACCAATGTAGACACCTGTGCAAGCACTTGTGCAGATACCAATGTAAACACTTGTGCAAGCATGTGTGCAGATACCAATGTAGACACCTGTGCAAGCACCTGTGCAAACACCTGTGCAAGCACAGAATAC |
| 38 | Vis86 | Portugal | DP | 1986 |  | I | [Nix et al 2006](file:///C:\Users\Cgallardo\Dropbox\Carmina%20AGOSTO%202014\SECUENCIAS%20CRL\Desktop\Carmina%20MARZO%202014\ARTICULOS\VPPA\GENOTIPADO\2003%20Bastos%20et%20al.pdf) | ATGCAGCCTACTCACCACGCAGAGATAAGCTTTCAGGATAGAGATACAGCTCTTCCAGACGCATGTTCATCTATATCGGATATTAGCCCCGTTACGTATCCGATCACATTACCTATTATTAAAAACATTTCCGTAACTGCTCATGGTATCAATCTTATCGATAAGTTTCCATCAAAGTTCTGCAGCTCTTACATACCCTTCCACTACGGAGGCAATGCAATTAAAACCCCCGATGATCCGGGTGCGATGATGATTACCTTTGCTTTGAAGCCACGGGAGGAATACCAACCCAGTGGTCATATTAACGTATCCAGAGCAAGAGAATTTTATATTAGTTGGGACACGGATTACGTGGGGTCTATCACTACGGCTGATCTTGTGGTATCGGCATCTGCTATTAACTT | AM259390 | [Nix et al 2006](http://asf-referencelab.info/asf/images/files/publicaciones/Nix_et_al_2006.pdf) | AGTGCGTATACTTGTGCAAGCACTTGTGCAGATACCAATGTAGACACCTGTGCAAGCACTTGTGCAAGCACTTGTGCAAGCACTTGTGCAAGCACAGGTGCAAGCACTTGTGCAGATACCAATGTAGACACCTGTGCAAGCACTTGTGCAGATACCAATGTAGACACCTGTGCAAGCACTTGTGCAGATACCAATGTAGACACCTGTGCAAGCACTTGTGCAGATACCAATGTAAACACTTGTGCAAGCATGTGTGCAGATACCAATGTAGACACCTGTGCAAGCACCTGTGCAAACACCTGTGCAAGCACAGAATAC |
| 39 | Coimbra87 | Portugal | DP | 1987 | DQ028310 | I | [Duarte et al 2005 (Unpublished)](http://asf-referencelab.info/asf/images/files/publicaciones/Gallardo-et-al-2009a.pdf) | ATGCAGCCTACTCACCACGCAGAGATAAGCTTTCAGGATAGAGATACAGCTCTTCCAGACGCATGTTCATCTATATCGGATATTAGCCCCGTTACGTATCCGATCACATTACCTATTATTAAAAACATTTCCGTAACTGCTCATGGTATCAATCTTATCGATAAGTTTCCATCAAAGTTCTGCAGCTCTTACATACCCTTCCACTACGGAGGCAATGCAATTAAAACCCCCGATGATCCGGGTGCGATGATGATTACCTTTGCTTTGAAGCCACGGGAGGAATACCAACCCAGTGGTCATATTAACGTATCCAGAGCAAGAGAATTTTATATTAGTTGGGACACGGATTACGTGGGGTCTATCACTACGGCTGATCTTGTGGTATCGGCATCTGCTATTAACTT | DQ028329 | [Duarte et al 2005 (Unpublished)](http://asf-referencelab.info/asf/images/files/publicaciones/Nix_et_al_2006.pdf) | AGTGCGTATACCTGTGCAAGCACTTGTGCAGATACCAATGTAGACACCTGTGCAAGCACTTGTGCAAGCACTTGTGCAAGCACTTGTGCAAGCACAGGTGCAAGCACTTGTGCAGATACCAATGTAGACACCTGTGCAAGCACTTGTGCAGATACCAATGTAGACACCTGTGCAAGCACTTGTGCAGATACCAATGTAGACACCTGTGCAAGCACTTGTGCAGATACCAATGTAAACACTTGTGCAAGCATGTGTGCAGATACCAATGTAGACACCTGTGCAAGCACCTGTGCAAACACCTGTGCAAGCACAGAATAC |
| 40 | KAL/88/1 | Zambia | DP | 1988 | AF449468 | VIII | [Bastos et al., 2003](http://asf-referencelab.info/asf/images/files/publicaciones/Gallardo-et-al-2009a.pdf) | ATGCAGCCTACTCACCACGCAGAAATAAGCTTTCAGGATAGAGATACAGCTCTTCCAGACGCATGTTCATCTATATCTGATATTAACCCCGTTACTTATCCGATCACATTACCTATTATTAAAAACATTTCCGTAACTGCTCATGGTATCAATCTTATCGATAAGTTTCCATCAAAGTTCTGCAGCTCTTACATACCCTTCCACTATGGAGGCAATTCGATTAAAACCCCTGATGATCCGGGCGCGATGATGATTACCTTCGCTTTGAAGCCACGGGAGGAATACCAACCCAGTGGTCATATTAACGTATCCAGAGCAAGAGAATTTTATATTAGTTGGGACACGGATTATGTGGGATCTATTACCACGGCTGATCTTGTGGTATCAGCATCTGCTATTAACTT | AY538732 | [Bastos et al., 2004](http://asf-referencelab.info/asf/images/files/publicaciones/Gianmaroli_et_al_2011.pdf) | AGTGCGTATACCTGTGCAAGCACCAATGCAGACACAAGTGCAAGCACCAATGCAGACACAAGTGCAAGCACCAATGCAGACACAAGTGCAAGCACCAATGCAAGCATAAATGCAGATACCAATGTAGATACTTGTGCAAGCACCAATGCAAGCACAAATGTAGACACCAATGCAAGCATAAATGCAAGCACCAATGCAAGCACAAATGTAGACACCAATGCAAGCACCAATGCAGACATCAATGCAAACACCAATGCAGACATCAATGCAAACATCAATGCAAACACAGAATAC |
| 41 | OURT88/3 | Portugal | Tick | 1988 | AM712240 | I | [Chapman et al., 2008](http://vir.sgmjournals.org/content/89/2/397.long) | ATGCAGCCTACTCACCACGCAGAGATAAGCTTTCAGGATAGAGATACAGCTCTTCCAGACGCATGTTCATCTATATCGGATATTAGCCCCGTTACGTATCCGATCACATTACCTATTATTAAAAACATTTCCGTAACTGCTCATGGTATCAATCTTATCGATAAGTTTCCATCAAAGTTCTGCAGCTCTTACATACCCTTCCACTACGGAGGCAATGCAATTAAAACCCCCGATGATCCGGGTGCGATGATGATTACCTTTGCTTTGAAGCCACGGGAGGAATACCAACCCAGTGGTCATATTAACGTATCCAGAGCAAGAGAATTTTATATTAGTTGGGACACGGATTACGTGGGGTCTATCACTACGGCTGATCTTGTGGTATCGGCATCTGCTATTAACTT | AM712240 | [Chapman et al., 2008](http://vir.sgmjournals.org/content/89/2/397.long) | AGTGCGTATACTTGTGCAAGCACTTGTGCAGATACCAATGTAGACACCTGTGCAAGCACTTGTGCAAGCACTTGTGCAAGCACTTGTGCAAGCACTTGTGCAAGCACTTGTGCAAGCACTTGTGCAAGCACTTGTGCAAGCACTTGTGCAAGCACTTGTGCAAGCACTTGTGCAAGCACTTGTGCAAGCACTTGTGCAAGCACAGGTGCAAGCACAGGTGCAAGCACTTGTGCAGATACCAATGTAGACACCTGTGCAAGCACTTGTGCAGATACCAATGTAGACACCTGTGCAAGCACTTGTGCAAGCACTTGTGCAAGCACTTGTGCAAGCACTTGTGCAAGCACAGGTGCAAGCACTTGTGCAGATACCAATGTAGACACCTGTGCAAGCACTTGTGCAGATACCAATGTAGACACCTGTGCAAGCACTTGTGCAGATACCAATGTAGACACCTGTGCAAGCACTTGTGCAGATACCAATGTAAACACTTGTGCAAGCATGTGTGCAGATACCAATGTAGACACCTGTGCAAGCACCTGTGCAAACACCTGTGCAAGCACAGAATAC |
| 42 | Sa88 | Spain | DP | 1988 | FJ174353 | I | [Gallardo et al., 2009](http://asf-referencelab.info/asf/images/files/publicaciones/Gianmaroli_et_al_2011.pdf) | ATGCAGCCTACTCACCACGCAGAGATAAGCTTTCAGGATAGAGATACAGCTCTTCCAGACGCATGTTCATCTATATCGGATATTAGCCCCGTTACGTATCCGATCACATTACCTATTATTAAAAACATTTCCGTAACTGCTCATGGTATCAATCTTATCGATAAGTTTCCATCAAAGTTCTGCAGCTCTTACATACCCTTCCACTACGGAGGCAATGCAATTAAAACCCCCGATGATCCGGGTGCGATGATGATTACCTTTGCTTTGAAGCCACGGGAGGAATACCAACCCAGTGGTCATATTAACGTATCCAGAGCAAGAGAATTTTATATTAGTTGGGACACGGATTACGTGGGGTCTATCACTACGGCTGATCTTGTGGTATCGGCATCTGCTATTAACTT | AM259447 | [Nix et al 2006](http://asf-referencelab.info/asf/images/files/publicaciones/Gianmaroli_et_al_2011.pdf) | AGTGCGTATACCTGTGCAAGCACTTGTGCAGATACCAATGTAGACACCTGTGCAAGCACTTGTGCAAGCACTTGTGCAAGCACTTGTGCAAGCACAGGTGCAAGCACTTGTGCAGATACCAATGTAGACACCTGTGCAAGCACTTGTGCAGATACCAATGTAGACACCTGTGCAAGCACTTGTGCAGATACCAATGTAGACACCTGTGCAAGCACTTGTGCAGATACCAATGTAAACACTTGTGCAAGCATGTGTGCAGATACCAATGTAGACACCTGTGCAAGCACCTGTGCAAACACCTGTGCAAGCACAGAATAC |
| 43 | Se88 | Spain | DP | 1988 | FJ174354 | I | [Gallardo et al., 2009](http://asf-referencelab.info/asf/images/files/publicaciones/Gianmaroli_et_al_2011.pdf) | ATGCAGCCTACTCACCACGCAGAGATAAGCTTTCAGGATAGAGATACAGCTCTTCCAGACGCATGTTCATCTATATCGGATATTAGCCCCGTTACGTATCCGATCACATTACCTATTATTAAAAACATTTCCGTAACTGCTCATGGTATCAATCTTATCGATAAGTTTCCATCAAAGTTCTGCAGCTCTTACATACCCTTCCACTACGGAGGCAATGCAATTAAAACCCCCGATGATCCGGGTGCGATGATGATTACCTTTGCTTTGAAGCCACGGGAGGAATACCAACCCAGTGGTCATATTAACGTATCCAGAGCAAGAGAATTTTATATTAGTTGGGACACGGATTACGTGGGGTCTATCACTACGGCTGATCTTGTGGTATCGGCATCTGCTATTAACTT | AM259446 | [Nix et al 2006](http://asf-referencelab.info/asf/images/files/publicaciones/Gianmaroli_et_al_2011.pdf) | AGTGCGTATACCTGTGCAAGCACTTGTGCAGATACCAATGTAGACACCTGTGCAAGCACTTGTGCAAGCACTTGTGCAAGCACTTGTGCAAGCACAGGTGCAAGCACTTGTGCAGATACCAATGTAGACACCTGTGCAAGCACTTGTGCAGATACCAATGTAGACACCTGTGCAAGCACTTGTGCAGATACCAATGTAGACACCTGTGCAAGCACTTGTGCAGATACCAATGTAAACACTTGTGCAAGCATGTGTGCAGATACCAATGTAGACACCTGTGCAAGCACCTGTGCAAACACCTGTGCAAGCACAGAATAC |
| 44 | Ss88 | Italy | DP | 1988 | FJ174362 | I | [Gallardo et al., 2009](http://asf-referencelab.info/asf/images/files/publicaciones/Gianmaroli_et_al_2011.pdf) | ATGCAGCCTACTCACCACGCAGAGATAAGCTTTCAGGATAGAGATACAGCTCTTCCAGACGCATGTTCATCTATATCGGATATTAGCCCCGTTACGTATCCGATCACATTACCTATTATTAAAAACATTTCCGTAACTGCTCATGGTATCAATCTTATCGATAAGTTTCCATCAAAGTTCTGCAGCTCTTACATACCCTTCCACTACGGAGGCAATGCAATTAAAACCCCCGATGATCCGGGTGCGATGATGATTACCTTTGCTTTGAAGCCACGGGAGGAATACCAACCCAGTGGTCATATTAACGTATCCAGAGCAAGAGAATTTTATATTAGTTGGGACACGGATTACGTGGGGTCTATCACTACGGCTGATCTTGTGGTATCGGCATCTGCTATTAACTT | AM259438 | [Nix et al 2006](http://asf-referencelab.info/asf/images/files/publicaciones/Gianmaroli_et_al_2011.pdf) | AGTGCGTATACTTGTGCAAGCACTTGTGCAGATACCAATGTAGACACCTGTGCAAGCACTTGTGCAAGCACTTGTGCAAGCACTTGTGCAAGCACAGGTGCAAGCACTTGTGCAGATACCAATGTAGACACCTGTGCAAGCACTTGTGCAGATACCAATGTAGACACCTGTGCAAGCACTTGTGCAGATACCAATGTAGACACCTGTGCAAGCACTTGTGCAGATACCAATGTAAACACTTGTGCAAGCATGTGTGCAGATACCAATGTAGACACCTGTGCAAGCACCTGTGCAAACACCTGTGCAAGCACAGAATAC |
| 45 | JON/89/13 | Zambia | DP | 1989 | AF449469 | VIII | [Bastos et al., 2003](http://asf-referencelab.info/asf/images/files/publicaciones/Gianmaroli_et_al_2011.pdf) | ATGCAGCCTACTCACCACGCAGAAATAAGCTTTCAGGATAGAGATACAGCTCTTCCAGACGCATGTTCATCTATATCTGATATTAACCCCGTTACTTATCCGATCACATTACCTATTATTAAAAACATTTCCGTAACTGCTCATGGTATCAATCTTATCGATAAGTTTCCATCAAAGTTCTGCAGCTCTTACATACCCTTCCACTATGGAGGCAATTCGATTAAAACCCCTGATGATCCGGGCGCGATGATGATTACCTTCGCTTTGAAGCCACGGGAGGAATACCAACCCAGTGGTCATATTAACGTATCCAGAGCAAGAGAATTTTATATTAGTTGGGACACGGATTATGTGGGATCTATTACCACGGCTGATCTTGTGGTATCAGCATCTGCTATTAACTT | AY538728 | [Bastos et al., 2004](http://asf-referencelab.info/asf/images/files/publicaciones/Gianmaroli_et_al_2011.pdf) | AGTGCGTATACCTGTGCAAGCACCAATGCAGACACAAGTGCAAGCACCAATGCAGACACAAGTGCAAGCACCAATGCAAGCATAAATGCAGATACCAATGTAGATACTTGTGCAAGCACCAATGCAAGCACAAATGTAGACACCAATGCAAGCATAAATGCAAGCACCAATGCAAGCACAAATGTAGACACCAATGCAAGCACCAATGCAGACATCAATGCAAACACCAATGCAGACATCAATGCAAACATCAATGCAAACACAGAATAC |
| 46 | BUR/90/1 | Burundi | DP | 1990 | AF449472 | X | [Bastos et al., 2003](http://asf-referencelab.info/asf/images/files/publicaciones/Gianmaroli_et_al_2011.pdf) | ATGCAGCCTACCCACCACGCAGAGGTAAGCTTTCAGGATAGAGATACAGCTCTTCCAGATGCATGTTCATCCATATCAGATATTTCCCCCATTACTTATCCGATCACGTTACCTATTATTAAAAACATTTCCGTCACTGCTCATGGTATCAATCTTATCGATAAATTTCCATCAAAGTTCTGCAGCTCTTACATACCCTTTCACTACGGAGGCAATTCGATTAAAACCCCCGACGATCCGGGCGCGATGATGATTACCTTTGCTTTGAAACCACGGGAGGAATACCAACCCAGTGGTCATATTAACGTATCCAGAGCAAGAGAGTTTTATATTAGCTGGGACACAGATTATGTGGGGTCTATCACCACGGCCGATCTTGTGGTATCGGCATCCGCTATTAACTT | AM259424 | [Nix et al 2006](http://asf-referencelab.info/asf/images/files/publicaciones/Gianmaroli_et_al_2011.pdf) | AGTGCGTATACCTGTGCAAGCACTTGTGCAAGCACTTGTGCAAGCACCTGTGCAGACACCAATGTAGACACTTGTGCAAGCACTTGTGCAAGCACTTGTGCAAGCACTTGTGCAAGCACTTGTGCAAGCACTTGTGCAAGCACTTGTGCAAGCACTTGTGCAAGCACTTGTGCAAGCACTTGTGCAAGCACTTGTGCAGACACCTGTGCAAGCACAGAATAC |
| 47 | Hu90 | Spain | DP | 1990 | FJ174355 | I | [Gallardo et al., 2009](http://asf-referencelab.info/asf/images/files/publicaciones/Gianmaroli_et_al_2011.pdf) | ATGCAGCCTACTCACCACGCAGAGATAAGCTTTCAGGATAGAGATACAGCTCTTCCAGACGCATGTTCATCTATATCGGATATTAGCCCCGTTACGTATCCGATCACATTACCTATTATTAAAAACATTTCCGTAACTGCTCATGGTATCAATCTTATCGATAAGTTTCCATCAAAGTTCTGCAGCTCTTACATACCCTTCCACTACGGAGGCAATGCAATTAAAACCCCCGATGATCCGGGTGCGATGATGATTACCTTTGCTTTGAAGCCACGGGAGGAATACCAACCCAGTGGTCATATTAACGTATCCAGAGCAAGAGAATTTTATATTAGTTGGGACACGGATTACGTGGGGTCTATCACTACGGCTGATCTTGTGGTATCGGCATCTGCTATTAACTT | AM259445 | [Nix et al 2006](http://asf-referencelab.info/asf/images/files/publicaciones/Gianmaroli_et_al_2011.pdf) | AGTGCGTATACCTGTGCAAGCACTTGTGCAGATACCAATGTAGACACCTGTGCAAGCACTTGTGCAAGCACTTGTGCAAGCACTTGTGCAAGCACAGGTGCAAGCACTTGTGCAGATACCAATGTAGACACCTGTGCAAGCACTTGTGCAGATACCAATGTAGACACCTGTGCAAGCACTTGTGCAGATACCAATGTAGACACCTGTGCAAGCACTTGTGCAGATACCAATGTAAACACTTGTGCAAGCATGTGTGCAGATACCAATGTAGACACCTGTGCAAGCACCTGTGCAAACACCTGTGCAAGCACAGAATAC |
| 48 | NDA/1/90 | Malawi | DP | 1990 | AF449473 | VIII | [Bastos et al., 2003](http://asf-referencelab.info/asf/images/files/publicaciones/Gallardo-et-al-2009a.pdf) | ATGCAGCCTACTCACCACGCAGAAATAAGCTTTCAGGATAGAGATACAGCTCTTCCAGACGCATGTTCATCTATATCTGATATTATCCCCGTTACTTATCCGATCACATTACCTATTATTAAAAACATTTCCGTAACTGCTCATGGTATCAATCTTATCGATAAGTTTCCATCAAAGTTCTGCAGCTCTTACATACCCTTCCACTATGGAGGCAATTCGATTAAAACCCCTGATGATCCGGGCGCGATGATGATTACCTTCGCTTTGAAGCCACGGGAGGAATACCAACCCAGTGGTCATATTAACGTATCCAGAGCAAGAGAATTTTATATTAGTTGGGACACGGATTATGTGGGATCTATTACCACGGCTGATCTTGTGGTATCAGCATCTGCTATTAACTT | AY538730 | [Bastos et al., 2004](http://wwwnc.cdc.gov/eid/article/20/9/14-0554_article) | AGTGCGTATACCTGTGCAAGCACCAATGCAGACACAAGTGCAAGCACCAATGCAGACACAAGTGCAAGCACCAATGCAAGCATAAATGCAGATACCAATGTAGATACTTGTGCAAGCACCAATGCAAGCACAAATGTAGACACCAATGCAAGCATAAATGCAAGCACCAATGCAAGCACAAATGTAGACACCAATGCAAGCACCAATGCAGACATCAATGCAAACACCAATGCAGACATCAATGCAAACATCAATGCAAACACAGAATACA |
| 49 | Nu90/1 | Italy | DP | 1990 | AF302813 | I | [Bastos et al., 2003](http://wwwnc.cdc.gov/eid/article/20/9/14-0554_article) | ATGCAGCCTACTCACCACGCAGAGATAAGCTTTCAGGATAGAGATACAGCTCTTCCAGACGCATGTTCATCTATATCGGATATTAGCCCCGTTACGTATCCGATCACATTACCTATTATTAAAAACATTTCCGTAACTGCTCATGGTATCAATCTTATCGATAAGTTTCCATCAAAGTTCTGCAGCTCTTACATACCCTTCCACTACGGAGGCAATGCAATTAAAACCCCCGATGATCCGGGTGCGATGATGATTACCTTTGCTTTGAAGCCACGGGAGGAATACCAACCCAGTGGTCATATTAACGTATCCAGAGCAAGAGAATTTTATATTAGTTGGGACACGGATTACGTGGGGTCTATCACTACGGCTGATCTTGTGGTATCGGCATCTGCTATTAACTT | AM259453 | [Nix et al 2006](http://wwwnc.cdc.gov/eid/article/20/9/14-0554_article) | AGTGCGTATACTTGTGCAAGCACTTGTGCAGATACCAATGTAGACACCTGTGCAAGCACTTGTGCAAGCACTTGTGCAAGCACTTGTGCAAGCACAGGTGCAAGCACTTGTGCAGATACCAATGTAGACACCTGTGCAAGCACTTGTGCAGATACCAATGTAGACACCTGTGCAAGCACTTGTGCAGATACCAATGTAGACACCTGTGCAAGCACTTGTGCAGATACCAATGTAAACACTTGTGCAAGCATGTGTGCAGATACCAATGTAGACACCTGTGCAAGCACCTGTGCAAACACCTGTGCAAGCACAGAATAC |
| 50 | Ori90 | Italy | DP | 1990 | FJ174363 | I | [Gallardo et al., 2009](http://wwwnc.cdc.gov/eid/article/20/9/14-0554_article) | ATGCAGCCTACTCACCACGCAGAGATAAGCTTTCAGGATAGAGATACAGCTCTTCCAGACGCATGTTCATCTATATCGGATATTAGCCCCGTTACGTATCCGATCACATTACCTATTATTAAAAACATTTCCGTAACTGCTCATGGTATCAATCTTATCGATAAGTTTCCATCAAAGTTCTGCAGCTCTTACATACCCTTCCACTACGGAGGCAATGCAATTAAAACCCCCGATGATCCGGGTGCGATGATGATTACCTTTGCTTTGAAGCCACGGGAGGAATACCAACCCAGTGGTCATATTAACGTATCCAGAGCAAGAGAATTTTATATTAGTTGGGACACGGATTACGTGGGGTCTATCACTACGGCTGATCTTGTGGTATCGGCATCTGCTATTAACTT | AM259406 | [Nix et al 2006](http://wwwnc.cdc.gov/eid/article/20/9/14-0554_article) | AGTGCGTATACTTGTGCAAGCACTTGTGCAGATACCAATGTAGACACCTGTGCAAGCACTTGTGCAAGCACTTGTGCAAGCACTTGTGCAAGCACAGGTGCAAGCACTTGTGCAGATACCAATGTAGACACCTGTGCAAGCACTTGTGCAGATACCAATGTAGACACCTGTGCAAGCACTTGTGCAGATACCAATGTAGACACCTGTGCAAGCACTTGTGCAGATACCAATGTAAACACTTGTGCAAGCATGTGTGCAGATACCAATGTAGACACCTGTGCAAGCACCTGTGCAAACACCTGTGCAAGCACAGAATAC |
| 51 | PORTALEGRE90 | Portugal | DP | 1990 | DQ028314 | I | [Duarte et al 2005 (Unpublished)](http://wwwnc.cdc.gov/eid/article/20/9/14-0554_article) | ATGCAGCCTACTCACCACGCAGAGATAAGCTTTCAGGATAGAGATACAGCTCTTCCAGACGCATGTTCATCTATATCGGATATTAGCCCCGTTACGTATCCGATCACATTACCTATTATTAAAAACATTTCCGTAACTGCTCATGGTATCAATCTTATCGATAAGTTTCCATCAAAGTTCTGCAGCTCTTACATACCCTTCCACTACGGAGGCAATGCAATTAAAACCCCCGATGATCCGGGTGCGATGATGATTACCTTTGCTTTGAAGCCACGGGAGGAATACCAACCCAGTGGTCATATTAACGTATCCAGAGCAAGAGAATTTTATATTAGTTGGGACACGGATTACGTGGGGTCTATCACTACGGCTGATCTTGTGGTATCGGCATCTGCTATTAACTT | DQ028332 | [Duarte et al 2005 (Unpublished)](http://wwwnc.cdc.gov/eid/article/20/9/14-0554_article) | AGTGCGTATACCTGTGCAAGCACTTGTGCAGATACCAATGTAGACACCTGTGCAAGCACTTGTGCAAGCACTTGTGCAAGCACTTGTGCAAGCACAGGTGCAAGCACTTGTGCAGATACCAATGTAGACACCTGTGCAAGCACTTGTGCAGATACCAATGTAGACACCTGTGCAAGCACTTGTGCAGATACCAATGTAGACACCTGTGCAAGCACTTGTGCAGATACCAATGTAAACACTTGTGCAAGCATGTGTGCAGATACCAATGTAGACACCTGTGCAAGCACCTGTGCAAACACCTGTGCAAGCACAGAATAC |
| 52 | Nu91/3 | Italy | DP | 1991 | FJ174364 | I | [Gallardo et al., 2009](http://asf-referencelab.info/asf/images/files/publicaciones/Gallardo-et-al-2009a.pdf) | ATGCAGCCTACTCACCACGCAGAGATAAGCTTTCAGGATAGAGATACAGCTCTTCCAGACGCATGTTCATCTATATCGGATATTAGCCCCGTTACGTATCCGATCACATTACCTATTATTAAAAACATTTCCGTAACTGCTCATGGTATCAATCTTATCGATAAGTTTCCATCAAAGTTCTGCAGCTCTTACATACCCTTCCACTACGGAGGCAATGCAATTAAAACCCCCGATGATCCGGGTGCGATGATGATTACCTTTGCTTTGAAGCCACGGGAGGAATACCAACCCAGTGGTCATATTAACGTATCCAGAGCAAGAGAATTTTATATTAGTTGGGACACGGATTACGTGGGGTCTATCACTACGGCTGATCTTGTGGTATCGGCATCTGCTATTAACTT | AM259455 | [Nix et al 2006](http://asf-referencelab.info/asf/images/files/publicaciones/Nix_et_al_2006.pdf) | AGTGCGTATACTTGTGCAAGCACTTGTGCAGATACCAATGTAGACACCTGTGCAAGCACTTGTGCAAGCACTTGTGCAAGCACTTGTGCAAGCACAGGTGCAAGCACTTGTGCAGATACCAATGTAGACACCTGTGCAAGCACTTGTGCAGATACCAATGTAGACACCTGTGCAAGCACTTGTGCAGATACCAATGTAGACACCTGTGCAAGCACTTGTGCAGATACCAATGTAAACACTTGTGCAAGCATGTGTGCAGATACCAATGTAGACACCTGTGCAAGCACCTGTGCAAACACCTGTGCAAGCACAGAATAC |
| 53 | Nu91/5 | Italy | DP | 1991 | FJ174365 | I | [Gallardo et al., 2009](http://asf-referencelab.info/asf/images/files/publicaciones/Gallardo-et-al-2009a.pdf) | ATGCAGCCTACTCACCACGCAGAGATAAGCTTTCAGGATAGAGATACAGCTCTTCCAGACGCATGTTCATCTATATCGGATATTAGCCCCGTTACGTATCCGATCACATTACCTATTATTAAAAACATTTCCGTAACTGCTCATGGTATCAATCTTATCGATAAGTTTCCATCAAAGTTCTGCAGCTCTTACATACCCTTCCACTACGGAGGCAATGCAATTAAAACCCCCGATGATCCGGGTGCGATGATGATTACCTTTGCTTTGAAGCCACGGGAGGAATACCAACCCAGTGGTCATATTAACGTATCCAGAGCAAGAGAATTTTATATTAGTTGGGACACGGATTACGTGGGGTCTATCACTACGGCTGATCTTGTGGTATCGGCATCTGCTATTAACTT | AM259456 | [Nix et al 2006](http://asf-referencelab.info/asf/images/files/publicaciones/Nix_et_al_2006.pdf) | AGTGCGTATACTTGTGCAAGCACTTGTGCAGATACCAATGTAGACACCTGTGCAAGCACTTGTGCAAGCACTTGTGCAAGCACTTGTGCAAGCACAGGTGCAAGCACTTGTGCAGATACCAATGTAGACACCTGTGCAAGCACTTGTGCAGATACCAATGTAGACACCTGTGCAAGCACTTGTGCAGATACCAATGTAGACACCTGTGCAAGCACTTGTGCAGATACCAATGTAAACACTTGTGCAAGCATGTGTGCAGATACCAATGTAGACACCTGTGCAAGCACCTGTGCAAACACCTGTGCAAGCACAGAATAC |
| 54 | Almodovar 99/E2 | Portugal | Tick | 1993 | DQ028308 | I | [Duarte et al 2005 (Unpublished)](http://asf-referencelab.info/asf/images/files/publicaciones/Gallardo-et-al-2009a.pdf) | ATGCAGCCTACTCACCACGCAGAGATAAGCTTTCAGGATAGAGATACAGCTCTTCCAGACGCATGTTCATCTATATCGGATATTAGCCCCGTTACGTATCCGATCACATTACCTATTATTAAAAACATTTCCGTAACTGCTCATGGTATCAATCTTATCGATAAGTTTCCATCAAAGTTCTGCAGCTCTTACATACCCTTCCACTACGGAGGCAATGCAATTAAAACCCCCGATGATCCGGGTGCGATGATGATTACCTTTGCTTTGAAGCCACGGGAGGAATACCAACCCAGTGGTCATATTAACGTATCCAGAGCAAGAGAATTTTATATTAGTTGGGACACGGATTACGTGGGGTCTATCACTACGGCTGATCTTGTGGTATCGGCATCTGCTATTAACTT | DQ028324 | [Duarte et al 2005 (Unpublished)](http://asf-referencelab.info/asf/images/files/publicaciones/Nix_et_al_2006.pdf) | AGTGCGTATACCTGTGCAAGCACTTGTGCAGATACCAATGTAGACACCTGTGCAAGCACTTGTGCAAGCACTTGTGCAAGCACTTGTGCAAGCACAGGTGCAAGCACTTGTGCAGATACCAATGTAGACACCTGTGCAAGCACTTGTGCAGATACCAATGTAGACACCTGTGCAAGCACTTGTGCAGATACCAATGTAGACACCTGTGCAAGCACTTGTGCAGATACCAATGTAAACACTTGTGCAAGCATGTGTGCAGATACCAATGTAGACACCTGTGCAAGCACCTGTGCAAACACCTGTGCAAGCACAGAATAC |
| 55 | Almodovar99 | Portugal | Tick | 1993 | DQ028306 | I | [Duarte et al 2005 (Unpublished)](http://asf-referencelab.info/asf/images/files/publicaciones/Gallardo-et-al-2009a.pdf) | ATGCAGCCTACTCACCACGCAGAGATAAGCTTTCAGGATAGAGATACAGCTCTTCCAGACGCATGTTCATCTATATCGGATATTAGCCCCGTTACGTATCCGATCACATTACCTATTATTAAAAACATTTCCGTAACTGCTCATGGTATCAATCTTATCGATAAGTTTCCATCAAAGTTCTGCAGCTCTTACATACCCTTCCACTACGGAGGCAATGCAATTAAAACCCCCGATGATCCGGGTGCGATGATGATTACCTTTGCTTTGAAGCCACGGGAGGAATACCAACCCAGTGGTCATATTAACGTATCCAGAGCAAGAGAATTTTATATTAGTTGGGACACGGATTACGTGGGGTCTATCACTACGGCTGATCTTGTGGTATCGGCATCTGCTATTAACTT | DQ028326 | [Duarte et al 2005 (Unpublished)](http://asf-referencelab.info/asf/images/files/publicaciones/Nix_et_al_2006.pdf) | AGTGCGTATACCTGTGCAAGCACTTGTGCAGATACCAATGTAGACACCTGTGCAAGCACTTGTGCAAGCACTTGTGCAAGCACTTGTGCAAGCACAGGTGCAAGCACTTGTGCAGATACCAATGTAGACACCTGTGCAAGCACTTGTGCAGATACCAATGTAGACACCTGTGCAAGCACTTGTGCAGATACCAATGTAGACACCTGTGCAAGCACTTGTGCAGATACCAATGTAAACACTTGTGCAAGCATGTGTGCAGATACCAATGTAGACACCTGTGCAAGCACCTGTGCAAACACCTGTGCAAGCACAGAATAC |
| 56 | Almodovar99/NE1 | Portugal | DP | 1993 | DQ028309 | I | [Duarte et al 2005 (Unpublished)](http://asf-referencelab.info/asf/images/files/publicaciones/Gallardo-et-al-2009a.pdf) | ATGCAGCCTACTCACCACGCAGAGATAAGCTTTCAGGATAGAGATACAGCTCTTCCAGACGCATGTTCATCTATATCGGATATTAGCCCCGTTACGTATCCGATCACATTACCTATTATTAAAAACATTTCCGTAACTGCTCATGGTATCAATCTTATCGATAAGTTTCCATCAAAGTTCTGCAGCTCTTACATACCCTTCCACTACGGAGGCAATGCAATTAAAACCCCCGATGATCCGGGTGCGATGATGATTACCTTTGCTTTGAAGCCACGGGAGGAATACCAACCCAGTGGTCATATTAACGTATCCAGAGCAAGAGAATTTTATATTAGTTGGGACACGGATTACGTGGGGTCTATCACTACGGCTGATCTTGTGGTATCGGCATCTGCTATTAACTT | DQ028325 | [Duarte et al 2005 (Unpublished)](http://asf-referencelab.info/asf/images/files/publicaciones/Nix_et_al_2006.pdf) | AGTGCGTATACCTGTGCAAGCACTTGTGCAGATACCAATGTAGACACCTGTGCAAGCACTTGTGCAAGCACTTGTGCAAGCACTTGTGCAAGCACAGGTGCAAGCACTTGTGCAGATACCAATGTAGACACCTGTGCAAGCACTTGTGCAGATACCAATGTAGACACCTGTGCAAGCACTTGTGCAGATACCAATGTAGACACCTGTGCAAGCACTTGTGCAGATACCAATGTAAACACTTGTGCAAGCATGTGTGCAGATACCAATGTAGACACCTGTGCAAGCACCTGTGCAAACACCTGTGCAAGCACAGAATAC |
| 57 | Barrancos93 | Portugal | DP | 1993 | DQ028307 | I | [Duarte et al 2005 (Unpublished)](file:///C:\Users\Cgallardo\Dropbox\Carmina%20AGOSTO%202014\SECUENCIAS%20CRL\Desktop\Carmina%20MARZO%202014\ARTICULOS\VPPA\GENOTIPADO\2003%20Bastos%20et%20al.pdf) | ATGCAGCCTACTCACCACGCAGAGATAAGCTTTCAGGATAGAGATACAGCTCTTCCAGACGCATGTTCATCTATATCGGATATTAGCCCCGTTACGTATCCGATCACATTACCTATTATTAAAAACATTTCCGTAACTGCTCATGGTATCAATCTTATCGATAAGTTTCCATCAAAGTTCTGCAGCTCTTACATACCCTTCCACTACGGAGGCAATGCAATTAAAACCCCCGATGATCCGGGTGCGATGATGATTACCTTTGCTTTGAAGCCACGGGAGGAATACCAACCCAGTGGTCATATTAACGTATCCAGAGCAAGAGAATTTTATATTAGTTGGGACACGGATTACGTGGGGTCTATCACTACGGCTGATCTTGTGGTATCGGCATCTGCTATTAACTT | DQ028327 | [Duarte et al 2005 (Unpublished)](http://asf-referencelab.info/asf/images/files/publicaciones/Nix_et_al_2006.pdf) | AGTGCGTATACCTGTGCAAGCACTTGTGCAGATACCAATGTAGACACCTGTGCAAGCACTTGTGCAAGCACTTGTGCAAGCACTTGTGCAAGCACAGGTGCAAGCACTTGTGCAGATACCAATGTAGACACCTGTGCAAGCACTTGTGCAGATACCAATGTAGACACCTGTGCAAGCACTTGTGCAGATACCAATGTAGACACCTGTGCAAGCACTTGTGCAGATACCAATGTAAACACTTGTGCAAGCATGTGTGCAGATACCAATGTAGACACCTGTGCAAGCACCTGTGCAAACACCTGTGCAAGCACAGAATAC |
| 58 | Hu94 | Spain | DP | 1994 | FJ174356 | I | [Gallardo et al., 2009](file:///C:\Users\Cgallardo\Dropbox\Carmina%20AGOSTO%202014\SECUENCIAS%20CRL\Desktop\Carmina%20MARZO%202014\ARTICULOS\VPPA\GENOTIPADO\2003%20Bastos%20et%20al.pdf) | ATGCAGCCTACTCACCACGCAGAGATAAGCTTTCAGGATAGAGATACAGCTCTTCCAGACGCATGTTCATCTATATCGGATATTAGCCCCGTTACGTATCCGATCACATTACCTATTATTAAAAACATTTCCGTAACTGCTCATGGTATCAATCTTATCGATAAGTTTCCATCAAAGTTCTGCAGCTCTTACATACCCTTCCACTACGGAGGCAATGCAATTAAAACCCCCGATGATCCGGGTGCGATGATGATTACCTTTGCTTTGAAGCCACGGGAGGAATACCAACCCAGTGGTCATATTAACGTATCCAGAGCAAGAGAATTTTATATTAGTTGGGACACGGATTACGTGGGGTCTATCACTACGGCTGATCTTGTGGTATCGGCATCTGCTATTAACTT | AM259443 | [Nix et al 2006](http://asf-referencelab.info/asf/images/files/publicaciones/Nix_et_al_2006.pdf) | AGTGCGTATACCTGTGCAAGCACTTGTGCAGATACCAATGTAGACACCTGTGCAAGCACTTGTGCAAGCACTTGTGCAAGCACTTGTGCAAGCACAGGTGCAAGCACTTGTGCAGATACCAATGTAGACACCTGTGCAAGCACTTGTGCAGATACCAATGTAGACACCTGTGCAAGCACTTGTGCAGATACCAATGTAGACACCTGTGCAAGCACTTGTGCAGATACCAATGTAAACACTTGTGCAAGCATGTGTGCAGATACCAATGTAGACACCTGTGCAAGCACCTGTGCAAACACCTGTGCAAGCACAGAATAC |
| 59 | MOZ/94/1 | Mozambique | DP | 1994 | AF270711 | VI | [Bastos et al., 2003](http://asf-referencelab.info/asf/images/files/publicaciones/Gallardo-et-al-2009a.pdf) | ATGCAGCCTACTCACCACGCAGAGATAAGCTTTCAGGATAGAGATACAGCTCTTCCAGACGCATGTTCATCTATATCTGATATTAGCCCCGTCACGTATCCGATCACATTACCTATTATTAAAAACATTTCTGTAACTGCTCATGGTATCAATCTTATCGATAAATTTCCATCAAAGTTCTGCAGCTCTTACATACCTTTCCACTACGGAGGCAATGCGATTAAAACCCCCGACGATCCGGGTGCGATGATGATTACCTTTGCCTTGAAGCCACGGGAGGAATACCAACCCAGTGGTCATATTAACGTATCTAGAGCAAGAGAATTTTATATTAGTTGGGATACGGATTACGTGGGGTCTATCACTACGGCTGATCTTGTGGTATCGGCATCTGCTATTAACTT | AY274468 | [Bastos et al., 2004](http://asf-referencelab.info/asf/images/files/publicaciones/Nix_et_al_2006.pdf) | AGTGCGTATACTTGTGCAAGCACTTGTGCAAGCACTTGTGCAGACACTTGTGCAAGCACTTGTGCAGATACCAATGTAGACACTTGTGCAAGCACTTGTGCAGACACTTGTGCAAGCACTTGTGCAGATACCAATGTAGACACTTGTGCAGACACTTGTGCAAGCACTTGTGCAGACACTAATGAAGACACTTGTGCAAGCACAGAATAC |
| 60 | MOZ/94/8 | Mozambique | DP | 1994 | AF270712 | VI | [Bastos et al., 2004](http://asf-referencelab.info/asf/images/files/publicaciones/Gallardo-et-al-2009a.pdf) | ATGCAGCCTACTCACCACGCAGAGATAAGCTTTCAGGATAGAGATACAGCTCTTCCAGACGCATGTTCATCTATATCTGATATTAGCCCCGTCACGTATCCGATCACATTACCTATTATTAAAAACATTTCTGTAACTGCTCATGGTATCAATCTTATCGATAAATTTCCATCAAAGTTCTGCAGCTCTTACATACCTTTCCACTACGGAGGCAATGCGATTAAAACCCCCGACGATCCGGGTGCGATGATGATTACCTTTGCCTTGAAGCCACGGGAGGAATACCAACCCAGTGGTCATATTAACGTATCTAGAGCAAGAGAATTTTATATTAGTTGGGATACGGATTACGTGGGGTCTATCACTACGGCTGATCTTGTGGTATCGGCATCTGCTATTAACTT | AY274469 | [Bastos et al., 2004](file:///C:\Users\Cgallardo\Dropbox\Carmina%20AGOSTO%202014\SECUENCIAS%20CRL\Desktop\Carmina%20MARZO%202014\ARTICULOS\VPPA\GENOTIPADO\2004%20Bastos%20et%20al.pdf) | AGTGCGTATACTTGTGCAAGCACTTGTGCAAGCACTTGTGCAGACACTTGTGCAAGCACTTGTGCAGATACCAATGTAGACACTTGTGCAAGCACTTGTGCAGACACTTGTGCAAGCACTTGTGCAGATACCAATGTAGACACTTGTGCAGACACTTGTGCAAGCACTTGTGCAGACACTAATGAAGACACTTGTGCAAGCACAGAATAC |
| 61 | SPEC265 | Mozambique | DP | 1994 | AF270710 | VI | [Bastos et al., 2003](http://asf-referencelab.info/asf/images/files/publicaciones/Gallardo-et-al-2009a.pdf) | ATGCAGCCTACTCACCACGCAGAGATAAGCTTTCAGGATAGAGATACAGCTCTTCCAGACGCATGTTCATCTATATCTGATATTAGCCCCGTCACGTATCCGATCACATTACCTATTATTAAAAACATTTCTGTAACTGCTCATGGTATCAATCTTATCGATAAATTTCCATCAAAGTTCTGCAGCTCTTACATACCTTTCCACTACGGAGGCAATGCGATTAAAACCCCCGACGATCCGGGTGCGATGATGATTACCTTTGCCTTGAAGCCACGGGAGGAATACCAACCCAGTGGTCATATTAACGTATCTAGAGCAAGAGAATTTTATATTAGTTGGGATACGGATTACGTGGGGTCTATCACTACGGCTGATCTTGTGGTATCGGCATCTGCTATTAACTT | AY274467 | [Bastos et al., 2004](http://asf-referencelab.info/asf/images/files/publicaciones/Nix_et_al_2006.pdf) | AGTGCGTATACTTGTGCAAGCACTTGTGCAAGCACTTGTGCAGACACTTGTGCAAGCACTTGTGCAGATACCAATGTAGACACTTGTGCAAGCACTTGTGCAGACACTTGTGCAAGCACTTGTGCAGATACCAATGTAGACACTTGTGCAGACACTTGTGCAAGCACTTGTGCAGACACTAATGAAGACACTTGTGCAAGCACAGAATAC |
| 62 | Nu95/1 | Italy | DP | 1995 | FJ174368 | I | [Gallardo et al., 2009](http://asf-referencelab.info/asf/images/files/publicaciones/Gallardo-et-al-2009a.pdf) | ATGCAGCCTACTCACCACGCAGAGATAAGCTTTCAGGATAGAGATACAGCTCTTCCAGACGCATGTTCATCTATATCGGATATTAGCCCCGTTACGTATCCGATCACATTACCTATTATTAAAAACATTTCCGTAACTGCTCATGGTATCAATCTTATCGATAAGTTTCCATCAAAGTTCTGCAGCTCTTACATACCCTTCCACTACGGAGGCAATGCAATTAAAACCCCCGATGATCCGGGTGCGATGATGATTACCTTTGCTTTGAAGCCACGGGAGGAATACCAACCCAGTGGTCATATTAACGTATCCAGAGCAAGAGAATTTTATATTAGTTGGGACACGGATTACGTGGGGTCTATCACTACGGCTGATCTTGTGGTATCGGCATCTGCTATTAACTT | AM259457 | [Nix et al 2006](http://asf-referencelab.info/asf/images/files/publicaciones/Nix_et_al_2006.pdf) | AGTGCGTATACTTGTGCAAGCACTTGTGCAGATACCAATGTAGACACCTGTGCAAGCACTTGTGCAAGCACTTGTGCAAGCACTTGTGCAAGCACAGGTGCAAGCACTTGTGCAGATACCAATGTAGACACCTGTGCAAGCACTTGTGCAGATACCAATGTAGACACCTGTGCAAGCACTTGTGCAGATACCAATGTAGACACCTGTGCAAGCACTTGTGCAGATACCAATGTAAACACTTGTGCAAGCATGTGTGCAGATACCAATGTAGACACCTGTGCAAGCACCTGTGCAAACACCTGTGCAAGCACAGAATAC |
| 63 | IC96/596 | Cote d Ivoire | DP | 1996 |  | I | This study (gallardo@inia.es) | ATGCAGCCTACTCACCACGCAGAGATAAGCTTTCAGGATAGAGATACAGCTCTTCCAGACGCATGTTCATCTATATCGGATATTAGCCCCGTTACGTATCCGATCACATTACCTATTATTAAAAACATTTCCGTAACTGCTCATGGTATCAATCTTATCGATAAGTTTCCATCAAAGTTCTGCAGCTCTTACATACCCTTCCACTACGGAGGCAATGCAATTAAAACCCCCGATGATCCGGGTGCGATGATGATTACCTTTGCTTTGAAGCCACGGGAGGAATACCAACCCAGTGGTCATATTAACGTATCCAGAGCAAGAGAATTTTATATTAGTTGGGACACGGATTACGTGGGGTCTATCACTACGGCTGATCTTGTGGTATCGGCATCTGCTATTAACTT |  | [Unpublished CISA (gallardo@inia.es)](http://asf-referencelab.info/asf/images/files/publicaciones/Nix_et_al_2006.pdf) | AGTGCGTATACTTGTGCAAGCACTTGTGCAAGCACTTGTGCAGATACCAATGTAGACACCTGTGCAAGCACTTGTGCAGATACCAATGTAAACACTTGTGCAAGCATGTGTGCAGATACCAATGTAGACACCTGTGCAAGCACCTGTGCAAGCACTTGTGCAAGCACTTGTGCAAGCACAGAATAC |
| 64 | IC96/696 | Cote d Ivoire | DP | 1996 |  | I | [This study (gallardo@inia.es)](http://asf-referencelab.info/asf/images/files/publicaciones/Gallardo-et-al-2009a.pdf) | ATGCAGCCTACTCACCACGCAGAGATAAGCTTTCAGGATAGAGATACAGCTCTTCCAGACGCATGTTCATCTATATCGGATATTAGCCCCGTTACGTATCCGATCACATTACCTATTATTAAAAACATTTCCGTAACTGCTCATGGTATCAATCTTATCGATAAGTTTCCATCAAAGTTCTGCAGCTCTTACATACCCTTCCACTACGGAGGCAATGCAATTAAAACCCCCGATGATCCGGGTGCGATGATGATTACCTTTGCTTTGAAGCCACGGGAGGAATACCAACCCAGTGGTCATATTAACGTATCCAGAGCAAGAGAATTTTATATTAGTTGGGACACGGATTACGTGGGGTCTATCACTACGGCTGATCTTGTGGTATCGGCATCTGCTATTAACTT |  | [This study (gallardo@inia.es)](http://asf-referencelab.info/asf/images/files/publicaciones/Nix_et_al_2006.pdf) | AGTGCGTATACTTGTGCAAGCACTTGTGCAAGCACTTGTGCAGATACCAATGTAGACACCTGTGCAAGCACTTGTGCAGATACCAATGTAAACACTTGTGCAAGCATGTGTGCAGATACCAATGTAGACACCTGTGCAAGCACCTGTGCAAGCACTTGTGCAAGCACTTGTGCAAGCACAGAATAC |
| 65 | IC/1/96 | Cote d Ivoire | DP | 1996 | AF302814 | I | [Bastos et al., 2003](http://asf-referencelab.info/asf/images/files/publicaciones/Gallardo-et-al-2009a.pdf) | ATGCAGCCTACTCACCACGCAGAGATAAGCTTTCAGGATAGAGATACAGCTCTTCCAGACGCATGTTCATCTATATCGGATATTAGCCCCGTTACGTATCCGATCACATTACCTATTATTAAAAACATTTCCGTAACTGCTCATGGTATCAATCTTATCGATAAGTTTCCATCAAAGTTCTGCAGCTCTTACATACCCTTCCACTACGGAGGCAATGCAATTAAAACCCCCGATGATCCGGGTGCGATGATGATTACCTTTGCTTTGAAGCCACGGGAGGAATACCAACCCAGTGGTCATATTAACGTATCCAGAGCAAGAGAATTTTATATTAGTTGGGACACGGATTACGTGGGGTCTATCACTACGGCTGATCTTGTGGTATCGGCATCTGCTATTAACTT | AF513036 | [Heath,L.E (Unpublished OVI](http://asf-referencelab.info/asf/images/files/publicaciones/Nix_et_al_2006.pdf) | AGTGCGTATACTTGTGCAAGCACTTGTGCAGATACCAATGTAGACACCTGTGCAAGCACTTGTGCAAGCACTTGTGCAAGCACTTGTGCAAGCACAGGTGCAAGCACTTGTGCAGATACCAATGTAGACACCTGTGCAAGCACTTGTGCAAGCACTTGTGCAAGCACTTGTGCAAGCACTTGTGCAAGCACAGGTGCAAGCACTTGTGCAGATACCAATGTAGACACCTGTGCAAGCACTTGTGCAAGCACTTGTGCAAGCACTTGTGCAAGCACTTGTGCAAGCACAGGTGCAAGCACTTGTGCAGATACCAATGTAGACACCTGTGCAAGCACTTGTGCAAGCACTTGTGCAAGCACTTGTGCAAGCACAGGTGCAAGCACTTGTGCAGATACCAATGTAGACACCTGTGCAAGCACCTGTGCAAACACCTGTGCAAGCACAGAATAC |
| 66 | IC/3/96 | Cote d Ivoire | DP | 1996 | AF504882 | I | [Bastos et al., 2003](http://asf-referencelab.info/asf/images/files/publicaciones/Gallardo-et-al-2009a.pdf) | ATGCAGCCTACTCACCACGCAGAGATAAGCTTTCAGGATAGAGATACAGCTCTTCCAGACGCATGTTCATGTATATCGGATATTAGCCCCGTTACGTATCCGATCACATTACCTATTATTAAAAACATTTCCGTAACTGCTCATGGTATCAATCTTATCGATAAGTTTCCATCAAAGTTCTGCAGCTCTTACATACCCTTCCACTACGGAGGCAATGCAATTAAAACCCCCGATGATCCGGGTGCGATGATGATTACCTTTGCTTTGAAGCCACGGGAGGAATACCAACCCAGTGGTCATATTAACGTATCCAGAGCAAGAGAATTTTATATTAGTTGGGACACGGATTACGTGGGGTCTATCACTACGGCTGATCTTGTGGTATCGGCATCTGCTATTAACTT | AF513053 | [Heath,L.E (Unpublished OVI](http://asf-referencelab.info/asf/images/files/publicaciones/Nix_et_al_2006.pdf) | AGTGCGTATACTTGTGCAAGCACTTGTGCAAGCACTTGTGCAGATACCAATGTAGACACCTGTGCAAGCACTTGTGCAGATACCAATGTAAACACTTGTGCAAGCATGTGTGCAGATACCAATGTAGACACCTGTGCAAGCACCTGTGCAAGCACTTGTGCAAGCACTTGTGCAAGCACAGAATAC |
| 67 | IC96 | Cote d Ivoire | DP | 1996 | FJ174379 | I | [Gallardo et al., 2009](http://asf-referencelab.info/asf/images/files/publicaciones/Gallardo-et-al-2009a.pdf) | ATGCAGCCTACTCACCACGCAGAGATAAGCTTTCAGGATAGAGATACAGCTCTTCCAGACGCATGTTCATCTATATCGGATATTAGCCCCGTTACGTATCCGATCACATTACCTATTATTAAAAACATTTCCGTAACTGCTCATGGTATCAATCTTATCGATAAGTTTCCATCAAAGTTCTGCAGCTCTTACATACCCTTCCACTACGGAGGCAATGCAATTAAAACCCCCGATGATCCGGGTGCGATGATGATTACCTTTGCTTTGAAGCCACGGGAGGAATACCAACCCAGTGGTCATATTAACGTATCCAGAGCAAGAGAATTTTATATTAGTTGGGACACGGATTACGTGGGGTCTATCACTACGGCTGATCTTGTGGTATCGGCATCTGCTATTAACTT |  | [This study (gallardo@inia.es)](http://asf-referencelab.info/asf/images/files/publicaciones/Nix_et_al_2006.pdf) | AGTGCGTATACTTGTGCAAGCACTTGTGCAAGCACTTGTGCAGATACCAATGTAGACACCTGTGCAAGCACTTGTGCAGATACCAATGTAAACACTTGTGCAAGCATGTGTGCAGATACCAATGTAGACACCTGTGCAAGCACCTGTGCAAGCACTTGTGCAAGCACTTGTGCAAGCACAGAATAC |
| 68 | Nu96 | Italy | DP | 1996 | FJ174369 | I | [Gallardo et al., 2009](http://asf-referencelab.info/asf/images/files/publicaciones/Gallardo-et-al-2009a.pdf) | ATGCAGCCTACTCACCACGCAGAGATAAGCTTTCAGGATAGAGATACAGCTCTTCCAGACGCATGTTCATCTATATCGGATATTAGCCCCGTTACGTATCCGATCACATTACCTATTATTAAAAACATTTCCGTAACTGCTCATGGTATCAATCTTATCGATAAGTTTCCATCAAAGTTCTGCAGCTCTTACATACCCTTCCACTACGGAGGCAATGCAATTAAAACCCCCGATGATCCGGGTGCGATGATGATTACCTTTGCTTTGAAGCCACGGGAGGAATACCAACCCAGTGGTCATATTAACGTATCCAGAGCAAGAGAATTTTATATTAGTTGGGACACGGATTACGTGGGGTCTATCACTACGGCTGATCTTGTGGTATCGGCATCTGCTATTAACTT | AM259458 | [Nix et al 2006](http://asf-referencelab.info/asf/images/files/publicaciones/Nix_et_al_2006.pdf) | AGTGCGTATACTTGTGCAAGCACTTGTGCAGATACCAATGTAGACACCTGTGCAAGCACTTGTGCAAGCACTTGTGCAAGCACTTGTGCAAGCACAGGTGCAAGCACTTGTGCAGATACCAATGTAGACACCTGTGCAAGCACTTGTGCAGATACCAATGTAGACACCTGTGCAAGCACTTGTGCAGATACCAATGTAGACACCTGTGCAAGCACTTGTGCAGATACCAATGTAAACACTTGTGCAAGCATGTGTGCAGATACCAATGTAGACACCTGTGCAAGCACCTGTGCAAACACCTGTGCAAGCACAGAATAC |
| 69 | IC96/796 | Cote d Ivoire | DP | 1996 |  | I | [This study (gallardo@inia.es)](http://asf-referencelab.info/asf/images/files/publicaciones/Gallardo-et-al-2009a.pdf) | ATGCAGCCTACTCACCACGCAGAGATAAGCTTTCAGGATAGAGATACAGCTCTTCCAGACGCATGTTCATCTATATCGGATATTAGCCCCGTTACGTATCCGATCACATTACCTATTATTAAAAACATTTCCGTAACTGCTCATGGTATCAATCTTATCGATAAGTTTCCATCAAAGTTCTGCAGCTCTTACATACCCTTCCACTACGGAGGCAATGCAATTAAAACCCCCGATGATCCGGGTGCGATGATGATTACCTTTGCTTTGAAGCCACGGGAGGAATACCAACCCAGTGGTCATATTAACGTATCCAGAGCAAGAGAATTTTATATTAGTTGGGACACGGATTACGTGGGGTCTATCACTACGGCTGATCTTGTGGTATCGGCATCTGCTATTAACTT |  | [This study (gallardo@inia.es)](http://asf-referencelab.info/asf/images/files/publicaciones/Nix_et_al_2006.pdf) | AGTGCGTATACTTGTGCAAGCACTTGTGCAAGCACTTGTGCAGATACCAATGTAGACACCTGTGCAAGCACTTGTGCAGATACCAATGTAAACACTTGTGCAAGCATGTGTGCAGATACCAATGTAGACACCTGTGCAAGCACCTGTGCAAGCACTTGTGCAAGCACTTGTGCAAGCACAGAATAC |
| 70 | 1/Nu/97 | Italy | D DP P | 1997 | FR668398. | I | [Giammarioli et al 2011](http://asf-referencelab.info/asf/images/files/publicaciones/Gianmaroli_et_al_2011.pdf) | ATGCAGCCTACTCACCACGCAGAGATAAGCTTTCAGGATAGAGATACAGCTCTTCCAGACGCATGTTCATCTATATCGGATATTAGCCCCGTTACGTATCCGATCACATTACCTATTATTAAAAACATTTCCGTAACTGCTCATGGTATCAATCTTATCGATAAGTTTCCATCAAAGTTCTGCAGCTCTTACATACCCTTCCACTACGGAGGCAATGCAATTAAAACCCCCGATGATCCGGGTGCGATGATGATTACCTTTGCTTTGAAGCCACGGGAGGAATACCAACCCAGTGGTCATATTAACGTATCCAGAGCAAGAGAATTTTATATTAGTTGGGACACGGATTACGTGGGGTCTATCACTACGGCTGATCTTGTGGTATCGGCATCTGCTATTAACTT | FR681789.2 | [Giammarioli et al 2011](http://asf-referencelab.info/asf/images/files/publicaciones/Nix_et_al_2006.pdf) | AGTGCGTATACCTGTGCAAGCACTTGTGCAGATACCAATGTAGACACCTGTGCAAGCACTTGTGCAAGCACTTGTGCAAGCACTTGTGCAAGCATGTGTGCAGATACCAATGTAGACACCTGTGCAAGCACCTGTGCAAACACCTGTGCAAGCACAGAATACACCGATTTAACAGATCCTGAGCGC |
| 71 | 2/Og/97 | Italy | DP | 1997 | FR668399 | I | [Giammarioli et al 2011](http://asf-referencelab.info/asf/images/files/publicaciones/Gianmaroli_et_al_2011.pdf) | ATGCAGCCTACTCACCACGCAGAGATAAGCTTTCAGGATAGAGATACAGCTCTTCCAGACGCATGTTCATCTATATCGGATATTAGCCCCGTTACGTATCCGATCACATTACCTATTATTAAAAACATTTCCGTAACTGCTCATGGTATCAATCTTATCGATAAGTTTCCATCAAAGTTCTGCAGCTCTTACATACCCTTCCACTACGGAGGCAATGCAATTAAAACCCCCGATGATCCGGGTGCGATGATGATTACCTTTGCTTTGAAGCCACGGGAGGAATACCAACCCAGTGGTCATATTAACGTATCCAGAGCAAGAGAATTTTATATTAGTTGGGACACGGATTACGTGGGGTCTATCACTACGGCTGATCTTGTGGTATCGGCATCTGCTATTAACTT | FR681790 | [Giammarioli et al 2011](http://asf-referencelab.info/asf/images/files/publicaciones/Nix_et_al_2006.pdf) | AGTGCGTATACCTGTGCAAGCACTTGTGCAGATACCAATGTAGACACCTGTGCAAGCACTTGTGCAAGCACTTGTGCAAGCACTTGTGCAAGCATGTGTGCAGATACCAATGTAGACACCTGGGCAAGCACCTGTGCAAACACCTGTGCAAGCACAGAATAC |
| 72 | Ca97 | Italy | DP | 1997 | FJ174371 | I | [Gallardo et al., 2009](http://asf-referencelab.info/asf/images/files/publicaciones/Gallardo-et-al-2009a.pdf) | ATGCAGCCTACTCACCACGCAGAGATAAGCTTTCAGGATAGAGATACAGCTCTTCCAGACGCATGTTCATCTATATCGGATATTAGCCCCGTTACGTATCCGATCACATTACCTATTATTAAAAACATTTCCGTAACTGCTCATGGTATCAATCTTATCGATAAGTTTCCATCAAAGTTCTGCAGCTCTTACATACCCTTCCACTACGGAGGCAATGCAATTAAAACCCCCGATGATCCGGGTGCGATGATGATTACCTTTGCTTTGAAGCCACGGGAGGAATACCAACCCAGTGGTCATATTAACGTATCCAGAGCAAGAGAATTTTATATTAGTTGGGACACGGATTACGTGGGGTCTATCACTACGGCTGATCTTGTGGTATCGGCATCTGCTATTAACTT | AM259460 | [Nix et al 2006](http://asf-referencelab.info/asf/images/files/publicaciones/Nix_et_al_2006.pdf) | AGTGCGTATACTTGTGCAAGCACTTGTGCAGATACCAATGTAGACACCTGTGCAAGCACTTGTGCAAGCACTTGTGCAAGCACTTGTGCAAGCACAGGTGCAAGCACTTGTGCAGATACCAATGTAGACACCTGTGCAAGCACTTGTGCAGATACCAATGTAGACACCTGTGCAAGCACTTGTGCAGATACCAATGTAGACACCTGTGCAAGCACTTGTGCAGATACCAATGTAAACACTTGTGCAAGCATGTGTGCAGATACCAATGTAGACACCTGTGCAAGCACCTGTGCAAACACCTGTGCAAGCACAGAATAC |
| 73 | CV97 | Cape Verde | DP | 1997 | FJ174380 | I | [Gallardo et al., 2009](http://asf-referencelab.info/asf/images/files/publicaciones/Gallardo-et-al-2009a.pdf) | ATGCAGCCTACTCACCACGCAGAGATAAGCTTTCAGGATAGAGATACAGCTCTTCCAGACGCATGTTCATCTATATCGGATATTAGCCCCGTTACGTATCCGATCACATTACCTATTATTAAAAACATTTCCGTAACTGCTCATGGTATCAATCTTATCGATAAGTTTCCATCAAAGTTCTGCAGCTCTTACATACCCTTCCACTACGGAGGCAATGCAATTAAAACCCCCGATGATCCGGGTGCGATGATGATTACCTTTGCTTTGAAGCCACGGGAGGAATACCAACCCAGTGGTCATATTAACGTATCCAGAGCAAGAGAATTTTATATTAGTTGGGACACGGATTACGTGGGGTCTATCACTACGGCTGATCTTGTGGTATCGGCATCTGCTATTAACTT | AM259462 | [Nix et al 2006](http://asf-referencelab.info/asf/images/files/publicaciones/Nix_et_al_2006.pdf) | AGTGCGTATACCTGTGCAAGCACTTGTGCAAGCACTTGTGCAGATACCAATGTAGACACCTGTGCAAGCACTTGTGCAGATACCAATGTAGACACCTGTGCAAGCACTTGTGCAGATACCAATGTAAACACTTGTGCAAGCATGTGTGCAGATACCAATGTAGACACCTGTGCAAGCACCTGTGCAAGCACTTGTGCAAGCACTTGTGCAAGCACAGAATAC |
| 74 | Nu97 | Italy | DP | 1997 | FJ174370 | I | [Gallardo et al., 2009](http://asf-referencelab.info/asf/images/files/publicaciones/Gallardo-et-al-2009a.pdf) | ATGCAGCCTACTCACCACGCAGAGATAAGCTTTCAGGATAGAGATACAGCTCTTCCAGACGCATGTTCATCTATATCGGATATTAGCCCCGTTACGTATCCGATCACATTACCTATTATTAAAAACATTTCCGTAACTGCTCATGGTATCAATCTTATCGATAAGTTTCCATCAAAGTTCTGCAGCTCTTACATACCCTTCCACTACGGAGGCAATGCAATTAAAACCCCCGATGATCCGGGTGCGATGATGATTACCTTTGCTTTGAAGCCACGGGAGGAATACCAACCCAGTGGTCATATTAACGTATCCAGAGCAAGAGAATTTTATATTAGTTGGGACACGGATTACGTGGGGTCTATCACTACGGCTGATCTTGTGGTATCGGCATCTGCTATTAACTT | AM259459 | [Nix et al 2006](http://asf-referencelab.info/asf/images/files/publicaciones/Nix_et_al_2006.pdf) | AGTGCGTATACTTGTGCAAGCACTTGTGCAGATACCAATGTAGACACCTGTGCAAGCACTTGTGCAAGCACTTGTGCAAGCACTTGTGCAAGCACAGGTGCAAGCACTTGTGCAGATACCAATGTAGACACCTGTGCAAGCACTTGTGCAGATACCAATGTAGACACCTGTGCAAGCACTTGTGCAGATACCAATGTAGACACCTGTGCAAGCACTTGTGCAGATACCAATGTAAACACTTGTGCAAGCATGTGTGCAGATACCAATGTAGACACCTGTGCAAGCACCTGTGCAAACACCTGTGCAAGCACAGAATAC |
| 75 | 3/Og/98 | Italy | DP | 1998 | FR668400 | I | [Giammarioli et al 2011](http://asf-referencelab.info/asf/images/files/publicaciones/Gallardo-et-al-2009a.pdf) | ATGCAGCCTACTCACCACGCAGAGATAAGCTTTCAGGATAGAGATACAGCTCTTCCAGACGCATGTTCATCTATATCGGATATTAGCCCCGTTACGTATCCGATCACATTACCTATTATTAAAAACATTTCCGTAACTGCTCATGGTATCAATCTTATCGATAAGTTTCCATCAAAGTTCTGCAGCTCTTACATACCCTTCCACTACGGAGGCAATGCAATTAAAACCCCCGATGATCCGGGTGCGATGATGATTACCTTTGCTTTGAAGCCACGGGAGGAATACCAACCCAGTGGTCATATTAACGTATCCAGAGCAAGAGAATTTTATATTAGTTGGGACACGGATTACGTGGGGTCTATCACTACGGCTGATCTTGTGGTATCGGCATCTGCTATTAACTT | FR681791 | [Giammarioli et al 2011](http://asf-referencelab.info/asf/images/files/publicaciones/Nix_et_al_2006.pdf) | AGTGCGTATACCTGTGCAAGCACTTGTGCAGATACCAATGTAGACACCTGTGCAAGCACTTGTGCAAGCACTTGTGCAAGCACTTGTGCAAGCATGTGTGCAGATACCAATGTAGACACCTGTGCAAGCACCTGTGCAAACACCTGTGCAAGCACAGAATAC |
| 76 | CV98 | Cape Verde | DP | 1998 | FJ174381 | I | [Gallardo et al., 2009](http://asf-referencelab.info/asf/images/files/publicaciones/Gallardo-et-al-2009a.pdf) | ATGCAGCCTACTCACCACGCAGAGATAAGCTTTCAGGATAGAGATACAGCTCTTCCAGACGCATGTTCATCTATATCGGATATTAGCCCCGTTACGTATCCGATCACATTACCTATTATTAAAAACATTTCCGTAACTGCTCATGGTATCAATCTTATCGATAAGTTTCCATCAAAGTTCTGCAGCTCTTACATACCCTTCCACTACGGAGGCAATGCAATTAAAACCCCCGATGATCCGGGTGCGATGATGATTACCTTTGCTTTGAAGCCACGGGAGGAATACCAACCCAGTGGTCATATTAACGTATCCAGAGCAAGAGAATTTTATATTAGTTGGGACACGGATTACGTGGGGTCTATCACTACGGCTGATCTTGTGGTATCGGCATCTGCTATTAACTT | AM259463 | [Nix et al 2006](http://asf-referencelab.info/asf/images/files/publicaciones/Gallardo_et_al_2011b_Uganda.pdf) | AGTGCGTATACCTGTGCAAGCACTTGTGCAAGCACTTGTGCAGATACCAATGTAGACACCTGTGCAAGCACTTGTGCAGATACCAATGTAGACACCTGTGCAAGCACTTGTGCAGATACCAATGTAAACACTTGTGCAAGCATGTGTGCAGATACCAATGTAGACACCTGTGCAAGCACCTGTGCAAGCACTTGTGCAAGCACTTGTGCAAGCACAGAATAC |
| 77 | MAD/1/98 | Madagascar | DP | 1998 | AF270706 | II | [[Bastos et al., 200](http://asf-referencelab.info/asf/images/files/publicaciones/Gallardo-et-al-2009a.pdf)3](http://asf-referencelab.info/asf/files/2003-Bastos.pdf) | ATGCAGCCCACTCACCACGCAGAGATAAGCTTTCAGGATAGAGATACAGCTCTTCCAGACGCATGTTCATCTATATCTGATATTAGCCCCGTTACGTATCCGATCACATTACCTATTATTAAAAACATTTCCGTAACTGCTCATGGTATCAATCTTATCGATAAATTTCCATCAAAGTTCTGCAGCTCTTACATACCCTTCCACTACGGAGGCAATGCGATTAAAACCCCCGATGATCCGGGTGCGATGATGATTACCTTTGCTTTGAAGCCACGGGAGGAATACCAACCCAGTGGTCATATTAACGTATCCAGAGCAAGAGAATTTTATATTAGTTGGGACACGGATTACGTGGGGTCTATCACTACGGCTGATCTTGTGGTATCGGCATCTGCTATTAACTT | AY274471 | [[[Bastos et al., 200](http://asf-referencelab.info/asf/images/files/publicaciones/Gallardo-et-al-2009a.pdf)3](http://asf-referencelab.info/asf/files/2003-Bastos.pdf)](http://asf-referencelab.info/asf/images/files/publicaciones/Gallardo-et-al-2009a.pdf) | AGTGCGTATACTTGTGCAGATACCAATGTAGACACTTGTGCAAGCATGTGTGCAGATACCAATGTAGACACCTGTGCAAGCATGTGTGCAGATACCAATGTAGATACCTGTGCAAGCACTTGTACAAGCACAGAATAC |
| 78 | MOZ/60-98 | Mozambique | DP | 1998 | AY274455 | II | [Bastos et al., 2004](http://asf-referencelab.info/asf/images/files/publicaciones/Gallardo-et-al-2009a.pdf) | ATGCAGCCCACTCACCACGCAGAGATAAGCTTTCAGGATAGAGATACAGCTCTTCCAGACGCATGTTCATCTATATCTGATATTAGCCCCGTTACGTATCCGATCACATTACCTATTATTAAAAACATTTCCGTAACTGCTCATGGTATCAATCTTATCGATAAATTTCCATCAAAGTTCTGCAGCTCTTACATACCCTTCCACTACGGAGGCAATGCGATTAAAACCCCCGATGATCCGGGTGCGATGATGATTACCTTTGCTTTGAAGCCACGGGAGGAATACCAACCCAGTGGTCATATTAACGTATCCAGAGCAAGAGAATTTTATATTAGTTGGGACACGGATTACGTGGGGTCTATCACTACGGCTGATCTTGTGGTATCGGCATCTGCTATTAACTT | AY274472 | [Bastos et al., 2004](http://asf-referencelab.info/asf/images/files/publicaciones/Gallardo-et-al-2009a.pdf) | AGTGCGTATACTTGTGCAGATACCAATGTAGACACTTGTGCAAGCATGTGTGCAGATACCAATGTAGACACCTGTGCAAGCATGTGTGCAGATACCAATGTAGATACCTGTGCAAGCACTTGTACAAGCACAGAATAC |
| 79 | MOZ/61-98 | Mozambique | DP | 1998 | AY274456 | II | [Bastos et al., 2004](http://asf-referencelab.info/asf/images/files/publicaciones/Gallardo-et-al-2009a.pdf) | ATGCAGCCCACTCACCACGCAGAGATAAGCTTTCAGGATAGAGATACAGCTCTTCCAGACGCATGTTCATCTATATCTGATATTAGCCCCGTTACGTATCCGATCACATTACCTATTATTAAAAACATTTCCGTAACTGCTCATGGTATCAATCTTATCGATAAATTTCCATCAAAGTTCTGCAGCTCTTACATACCCTTCCACTACGGAGGCAATGCGATTAAAACCCCCGATGATCCGGGTGCGATGATGATTACCTTTGCTTTGAAGCCACGGGAGGAATACCAACCCAGTGGTCATATTAACGTATCCAGAGCAAGAGAATTTTATATTAGTTGGGACACGGATTACGTGGGGTCTATCACTACGGCTGATCTTGTGGTATCGGCATCTGCTATTAACTT | AY274473 | [Bastos et al., 2004](http://asf-referencelab.info/asf/images/files/publicaciones/Gallardo-et-al-2009a.pdf) | AGTGCGTATACTTGTGCAGATACCAATGTAGACACTTGTGCAAGCATGTGTGCAGATACCAATGTAGACACCTGTGCAAGCATGTGTGCAGATACCAATGTAGATACCTGTGCAAGCACTTGTACAAGCACAGAATAC |
| 80 | MOZ/62-98 | Mozambique | DP | 1998 | AY274457 | VIII | [Bastos et al., 2004](http://asf-referencelab.info/asf/images/files/publicaciones/Gallardo-et-al-2009a.pdf) | ATGCAGCCTACTCACCACGCAGAAATAAGCTTTCAGGATAGAGATACAGCTCTTCCAGACGCATGTTCATCTATATCTGATATTAACCCCGTTACTTATCCGATCACATTACCTATTATTAAAAACATTTCCGTAACTGCTCATGGTATCAATCTTATCGATAAGTTTCCATCAAAGTTCTGCAGCTCTTACATACCCTTCCACTATGGAGGCAATTCGATTAAAACCCCTGATGATCCGGGCGCGATGATGATTACCTTCGCTTTGAAGCCACGGGAGGAATACCAACCCAGTGGTCATATTAACGTATCCAGAGCAAGAGAATTTTATATTAGTTGGGACACGGATTATGTGGGATCTATTACCACGGCTGATCTTGTGGTATCAGCATCTGCTATTAACTT | AY274464 | [Bastos et al., 2004](http://asf-referencelab.info/asf/images/files/publicaciones/Gallardo_et_al_2011b_Uganda.pdf) | AGTGCGTATACCTGTGCAAGCACCAATGCAGACACAAGTGCAAGCACCAATGCAGACACAAGTGCAAGCACCAATGCAGACACAAGTGCAAGCACCAATGCAAGCATAAATGCAGATACCAATGTAGATACTTGTGCAAGCACCAATGCAAGCACAAATGTAGACACCAATGCAAGCATAAATGCAAGCACCAATGCAAGCACAAATGTAGACACCAATGCAAGCACCAATGCAGACATCAATGCAAACACCAATGCAGACATCAATGCAAACATCAATGCAAACACAGAATAC |
| 81 | MOZ/63-98 | Mozambique | DP | 1998 | AY274458 | II | [Bastos et al., 2004](http://asf-referencelab.info/asf/images/files/publicaciones/Gallardo-et-al-2009a.pdf) | ATGCAGCCCACTCACCACGCAGAGATAAGCTTTCAGGATAGAGATACAGCTCTTCCAGACGCATGTTCATCTATATCTGATATTAGCCCCGTTACGTATCCGATCACATTACCTATTATTAAAAACATTTCCGTAACTGCTCATGGTATCAATCTTATCGATAAATTTCCATCAAAGTTCTGCAGCTCTTACATACCCTTCCACTACGGAGGCAATGCGATTAAAACCCCCGATGATCCGGGTGCGATGATGATTACCTTTGCTTTGAAGCCACGGGAGGAATACCAACCCAGTGGTCATATTAACGTATCCAGAGCAAGAGAATTTTATATTAGTTGGGACACGGATTACGTGGGGTCTATCACTACGGCTGATCTTGTGGTATCGGCATCTGCTATTAACTT | AY274474 | [Bastos et al., 2004](http://asf-referencelab.info/asf/images/files/publicaciones/Gallardo-et-al-2009a.pdf) | AGTGCGTATACTTGTGCAGATACCAATGTAGACACTTGTGCAAGCATGTGTGCAGATACCAATGTAGACACCTGTGCAAGCATGTGTGCAGATACCAATGTAGATACCTGTGCAAGCACTTGTACAAGCACAGAATAC |
| 82 | MOZ/70-98 | Mozambique | DP | 1998 | AY274459 | II | [Bastos et al., 2004](http://asf-referencelab.info/asf/images/files/publicaciones/Gallardo-et-al-2009a.pdf) | ATGCAGCCCACTCACCACGCAGAGATAAGCTTTCAGGATAGAGATACAGCTCTTCCAGACGCATGTTCATCTATATCTGATATTAGCCCCGTTACGTATCCGATCACATTACCTATTATTAAAAACATTTCCGTAACTGCTCATGGTATCAATCTTATCGATAAATTTCCATCAAAGTTCTGCAGCTCTTACATACCCTTCCACTACGGAGGCAATGCGATTAAAACCCCCGATGATCCGGGTGCGATGATGATTACCTTTGCTTTGAAGCCACGGGAGGAATACCAACCCAGTGGTCATATTAACGTATCCAGAGCAAGAGAATTTTATATTAGTTGGGACACGGATTACGTGGGGTCTATCACTACGGCTGATCTTGTGGTATCGGCATCTGCTATTAACTT | AY274475 | [Bastos et al., 2004](http://asf-referencelab.info/asf/images/files/publicaciones/Gallardo-et-al-2009a.pdf) | AGTGCGTATACTTGTGCAGATACCAATGTAGACACTTGTGCAAGCATGTGTGCAGATACCAATGTAGACACCTGTGCAAGCATGTGTGCAGATACCAATGTAGATACCTGTGCAAGCACTTGTACAAGCACAGAATAC |
| 83 | MOZ/A-98 | Mozambique | DP | 1998 | AY274452 | VIII | [Bastos et al., 2004](http://asf-referencelab.info/asf/images/files/publicaciones/Gallardo-et-al-2009a.pdf) | ATGCAGCCTACTCACCACGCAGAAATAAGCTTTCAGGATAGAGATACAGCTCTTCCAGACGCATGTTCATCTATATCTGATATTAACCCCGTTACTTATCCGATCACATTACCTATTATTAAAAACATTTCCGTAACTGCTCATGGTATCAATCTTATCGATAAGTTTCCATCAAAGTTCTGCAGCTCTTACATACCCTTCCACTATGGAGGCAATTCGATTAAAACCCCTGATGATCCGGGCGCGATGATGATTACCTTCGCTTTGAAGCCACGGGAGGAATACCAACCCAGTGGTCATATTAACGTATCCAGAGCAAGAGAATTTTATATTAGTTGGGACACGGATTATGTGGGATCTATTACCACGGCTGATCTTGTGGTATCAGCATCTGCTATTAACTT | AY274461 | [Bastos et al., 2004](http://asf-referencelab.info/asf/images/files/publicaciones/Gallardo_et_al_2011b_Uganda.pdf) | AGTGCGTATACCTGTGCAAGCACCAATGCAGACACAAGTGCAAGCACCAATGCAGACACAAGTGCAAGCACCAATGCAGACACAAGTGCAAGCACCAATGCAAGCATAAATGCAGATACCAATGTAGATACTTGTGCAAGCACCAATGCAAGCACAAATGTAGACACCAATGCAAGCATAAATGCAAGCACCAATGCAAGCACAAATGTAGACACCAATGCAAGCACCAATGCAGACATCAATGCAAACACCAATGCAGACATCAATGCAAACATCAATGCAAACACAGAATAC |
| 84 | MOZ/B-98 | Mozambique | DP | 1998 | AY274453 | VIII | [Bastos et al., 2004](http://asf-referencelab.info/asf/images/files/publicaciones/Gallardo-et-al-2009a.pdf) | ATGCAGCCTACTCACCACGCAGAAATAAGCTTTCAGGATAGAGATACAGCTCTTCCAGACGCATGTTCATCTATATCTGATATTAACCCCGTTACTTATCCGATCACATTACCTATTATTAAAAACATTTCCGTAACTGCTCATGGTATCAATCTTATCGATAAGTTTCCATCAAAGTTCTGCAGCTCTTACATACCCTTCCACTATGGAGGCAATTCGATTAAAACCCCTGATGATCCGGGCGCGATGATGATTACCTTCGCTTTGAAGCCACGGGAGGAATACCAACCCAGTGGTCATATTAACGTATCCAGAGCAAGAGAATTTTATATTAGTTGGGACACGGATTATGTGGGATCTATTACCACGGCTGATCTTGTGGTATCAGCATCTGCTATTAACTT | AY274462 | [Bastos et al., 2004](http://asf-referencelab.info/asf/images/files/publicaciones/Gallardo_et_al_2011b_Uganda.pdf) | AGTGCGTATACCTGTGCAAGCACCAATGCAGACACAAGTGCAAGCACCAATGCAGACACAAGTGCAAGCACCAATGCAGACACAAGTGCAAGCACCAATGCAAGCATAAATGCAGATACCAATGTAGATACTTGTGCAAGCACCAATGCAAGCACAAATGTAGACACCAATGCAAGCATAAATGCAAGCACCAATGCAAGCACAAATGTAGACACCAATGCAAGCACCAATGCAGACATCAATGCAAACACCAATGCAGACATCAATGCAAACATCAATGCAAACACAGAATAC |
| 85 | MOZ/C-98 | Mozambique | DP | 1998 | AY274454 | VIII | [Bastos et al., 2004](http://asf-referencelab.info/asf/images/files/publicaciones/Gallardo-et-al-2009a.pdf) | ATGCAGCCTACTCACCACGCAGAAATAAGCTTTCAGGATAGAGATACAGCTCTTCCAGACGCATGTTCATCTATATCTGATATTAACCCCGTTACTTATCCGATCACATTACCTATTATTAAAAACATTTCCGTAACTGCTCATGGTATCAATCTTATCGATAAGTTTCCATCAAAGTTCTGCAGCTCTTACATACCCTTCCACTATGGAGGCAATTCGATTAAAACCCCTGATGATCCGGGCGCGATGATGATTACCTTCGCTTTGAAGCCACGGGAGGAATACCAACCCAGTGGTCATATTAACGTATCCAGAGCAAGAGAATTTTATATTAGTTGGGACACGGATTATGTGGGATCTATTACCACGGCTGATCTTGTGGTATCAGCATCTGCTATTAACTT | AY274463 | [Bastos et al., 2004](http://asf-referencelab.info/asf/images/files/publicaciones/Gallardo_et_al_2011b_Uganda.pdf) | AGTGCGTATACCTGTGCAAGCACCAATGCAGACACAAGTGCAAGCACCAATGCAGACACAAGTGCAAGCACCAATGCAGACACAAGTGCAAGCACCAATGCAAGCATAAATGCAGATACCAATGTAGATACTTGTGCAAGCACCAATGCAAGCACAAATGTAGACACCAATGCAAGCATAAATGCAAGCACCAATGCAAGCACAAATGTAGACACCAATGCAAGCACCAATGCAGACATCAATGCAAACACCAATGCAGACATCAATGCAAACATCAATGCAAACACAGAATAC |
| 86 | Nu98/3 | Italy | DP | 1998 | FJ174372 | I | [Gallardo et al., 2009](http://asf-referencelab.info/asf/images/files/publicaciones/Gallardo-et-al-2009a.pdf) | ATGCAGCCTACTCACCACGCAGAGATAAGCTTTCAGGATAGAGATACAGCTCTTCCAGACGCATGTTCATCTATATCGGATATTAGCCCCGTTACGTATCCGATCACATTACCTATTATTAAAAACATTTCCGTAACTGCTCATGGTATCAATCTTATCGATAAGTTTCCATCAAAGTTCTGCAGCTCTTACATACCCTTCCACTACGGAGGCAATGCAATTAAAACCCCCGATGATCCGGGTGCGATGATGATTACCTTTGCTTTGAAGCCACGGGAGGAATACCAACCCAGTGGTCATATTAACGTATCCAGAGCAAGAGAATTTTATATTAGTTGGGACACGGATTACGTGGGGTCTATCACTACGGCTGATCTTGTGGTATCGGCATCTGCTATTAACTT | AM25945 | [Nix et al 2006](http://asf-referencelab.info/asf/images/files/publicaciones/Gallardo_et_al_2011b_Uganda.pdf) | AGTGCGTATACTTGTGCAAGCACTTGTGCAGATACCAATGTAGACACCTGTGCAAGCACTTGTGCAAGCACTTGTGCAAGCACTTGTGCAAGCACAGGTGCAAGCACTTGTGCAGATACCAATGTAGACACCTGTGCAAGCACTTGTGCAGATACCAATGTAGACACCTGTGCAAGCACTTGTGCAGATACCAATGTAGACACCTGTGCAAGCACTTGTGCAGATACCAATGTAAACACTTGTGCAAGCATGTGTGCAGATACCAATGTAGACACCTGTGCAAGCACCTGTGCAAACACCTGTGCAAGCACAGAATAC |
| 87 | Nig01 | Nigeria | DP | 2001 | FJ174382 | I | [Gallardo et al., 2009](http://asf-referencelab.info/asf/images/files/publicaciones/Gallardo-et-al-2009a.pdf) | ATGCAGCCTACTCACCACGCAGAGATAAGCTTTCAGGATAGAGATACAGCTCTTCCAGACGCATGTTCATCTATATCGGATATTAGCCCCGTTACGTATCCGATCACATTACCTATTATTAAAAACATTTCCGTAACTGCTCATGGTATCAATCTTATCGATAAGTTTCCATCAAAGTTCTGCAGCTCTTACATACCCTTCCACTACGGAGGCAATGCAATTAAAACCCCCGATGATCCGGGTGCGATGATGATTACCTTTGCTTTGAAGCCACGGGAGGAATACCAACCCAGTGGTCATATTAACGTATCCAGAGCAAGAGAATTTTATATTAGTTGGGACACGGATTACGTGGGGTCTATCACTACGGCTGATCTTGTGGTATCGGCATCTGCTATTAACTT | AM25946 | [Nix et al 2006](http://asf-referencelab.info/asf/images/files/publicaciones/Gallardo_et_al_2011b_Uganda.pdf) | AGTGCGTATACCTGTGCAAGCACTTGTGCAAGCACTTGTGCAAGCACAGGTGCAAGCACTTGTGCAGATACCAATGTAGACACCTGTGCAAGCACTTGTGCAAGCACTTGTGCAAGCACTTGTGCAAGCACTTGTGCAAGCACTTGTGCAAGCACAGGTGCAAGCACTTGTGCAGATACCAATGTAGACACCTGTGCAAGCACTTGTGCAAGCACTTGTGCAAGCACTTGTGCAAGCACTTGTGCAAGCACTTGTGCAAGCACAGGTGCAAGCACTTGTGCAGATACCAATGTAGACACCTGTGCAAGCACTTGTGCAAGCACTTGTGCAAGCACTTGTGCAAGCACAGGTGCAAGCACTTGTGCAGATACCAATGTAGACCACCCCTGTGCAAGCACAGAATAC |
| 88 | 4/Ol/02 | Italy | DP | 2002 | FR668401 | I | Giammaroli et al 2011 | ATGCAGCCTACTCACCACGCAGAGATAAGCTTTCAGGATAGAGATACAGCTCTTCCAGACGCATGTTCATCTATATCGGATATTAGCCCCGTTACGTATCCGATCACATTACCTATTATTAAAAACATTTCCGTAACTGCTCATGGTATCAATCTTATCGATAAGTTTCCATCAAAGTTCTGCAGCTCTTACATACCCTTCCACTACGGAGGCAATGCAATTAAAACCCCCGATGATCCGGGTGCGATGATGATTACCTTTGCTTTGAAGCCACGGGAGGAATACCAACCCAGTGGTCATATTAACGTATCCAGAGCAAGAGAATTTTATATTAGTTGGGACACGGATTACGTGGGGTCTATCACTACGGCTGATCTTGTGGTATCGGCATCTGCTATTAACTT | FR686538 | Giammaroli et al 2011 | AGTGCGTATACCTGTGCGAGCACTTGTGCAGATACCAATGTAGACACCTGTGCCAGCACTTGTGCAAGCACTTGTGCAAGCACTTGTGCAAGCATGTGTGCAGATACCAATGTAGACACCTGCGCAAGCACCTGTGCAAACACCTGTGCAAGCACAGAATAC |
| 89 | 5/Ca/02 | Italy | DP | 2002 | FR668402 | I | Giammaroli et al 2011 | ATGCAGCCTACTCACCACGCAGAGATAAGCTTTCAGGATAGAGATACAGCTCTTCCAGACGCATGTTCATCTATATCGGATATTAGCCCCGTTACGTATCCGATCACATTACCTATTATTAAAAACATTTCCGTAACTGCTCATGGTATCAATCTTATCGATAAGTTTCCATCAAAGTTCTGCAGCTCTTACATACCCTTCCACTACGGAGGCAATGCAATTAAAACCCCCGATGATCCGGGTGCGATGATGATTACCTTTGCTTTGAAGCCACGGGAGGAATACCAACCCAGTGGTCATATTAACGTATCCAGAGCAAGAGAATTTTATATTAGTTGGGACACGGATTACGTGGGGTCTATCACTACGGCTGATCTTGTGGTATCGGCATCTGCTATTAACTT | FR686539 | Giammaroli et al 2011 | AGTGCGTATACCTGTGCGAGCACTTGTGCAGATACCAATGTAGACACCTGTGCAAGCACTTGTGCAAGCACTTGTGCAAGCACTTGTGCAAGCATGTGTGCAGATACCAATGTAGACACCTGCGCAAGCACCTGTGCAAACACCTGTGCAAGCACAGAATAC |
| 90 | Ug03H.1 | Uganda | DP | 2003 | FJ154428 | IX | [Gallardo et al., 2009](http://asf-referencelab.info/asf/images/files/publicaciones/Gallardo-et-al-2009a.pdf) | ATGCAGCCTACCCACCACGCAGAGGTAAGCTTTCAGGATAGAGATACAGCTCTTCCAGATGCATGTTCATCCATATCTGATATTACCCCCATTACTTATCCGATCACATTACCTATTATTAAAAACATTTCCGTTACTGCTCACGGTATCAATCTTATCGATAAATTTCCATCAAAGTTCTGCAGCTCTTACATACCCTTCCACTACGGAGGCAATTCGATTAAAACCCCCGACGATCCGGGCGCGATGATGATTACCTTTGCTTTGAAACCACGGGAGGAATACCAACCCAGCGGTCATATTAACGTATCCAGAGCAAGAGAATTTTATATTAGCTGGGACACAGATTATGTGGGGTCTATCACCACGGCTGATCTTGTGGTATCGGCATCCGCTATTAACTT | FJ174339 | [Gallardo et al., 2009](http://asf-referencelab.info/asf/images/files/publicaciones/Gallardo_et_al_2011b_Uganda.pdf) | AGTGCGTATACCTGTGCAAGCACTTGTGCAAGCACTTGTGCAAGCACTTGTGCAGACACCAATGTAGACACTTGTGCAAGCACTTGTGCAGACATTTGTGCAGACACCAATGTAGACACTTGTGCAAGCACTTGTGCAGATACTTGTGCAGACACTTGTGTAAGCACTTGTGTAAGCACTTGTGCAGATACTTGTGCAGACACCAATGTAGACACTTGTGCAAGCACTTGTGCAGACACCAATGTAGACACTTGTGTAAGCACTTGTGCAGACACCTGTGCAAGCACAGAATAC |
| 91 | Ug03H.2 | Uganda | DP | 2003 | FJ154429 | IX | [Gallardo et al., 2009](http://asf-referencelab.info/asf/images/files/publicaciones/Gallardo-et-al-2009a.pdf) | ATGCAGCCTACCCACCACGCAGAGGTAAGCTTTCAGGATAGAGATACAGCTCTTCCAGATGCATGTTCATCCATATCTGATATTACCCCCATTACTTATCCGATCACATTACCTATTATTAAAAACATTTCCGTTACTGCTCACGGTATCAATCTTATCGATAAATTTCCATCAAAGTTCTGCAGCTCTTACATACCCTTCCACTACGGAGGCAATTCGATTAAAACCCCCGACGATCCGGGCGCGATGATGATTACCTTTGCTTTGAAACCACGGGAGGAATACCAACCCAGCGGTCATATTAACGTATCCAGAGCAAGAGAATTTTATATTAGCTGGGACACAGATTATGTGGGGTCTATCACCACGGCTGATCTTGTGGTATCGGCATCCGCTATTAACTT | FJ174340 | [Gallardo et al., 2009](http://asf-referencelab.info/asf/images/files/publicaciones/Gallardo_et_al_2011b_Uganda.pdf) | AGTGCGTATACCTGTGCAAGCACTTGTGCAAGCACTTGTGCAAGCACTTGTGCAGACACCAATGTAGACACTTGTGCAAGCACTTGTGCAGACATTTGTGCAGACACCAATGTAGACACTTGTGCAAGCACTTGTGCAGATACTTGTGCAGACACTTGTGTAAGCACTTGTGTAAGCACTTGTGCAGATACTTGTGCAGACACCAATGTAGACACTTGTGCAAGCACTTGTGCAGACACCAATGTAGACACTTGTGTAAGCACTTGTGCAGACACCTGTGCAAGCACAGAATAC |
| 92 | Ug03H.3 | Uganda | DP | 2003 | FJ154430 | IX | [Gallardo et al., 2009](http://asf-referencelab.info/asf/images/files/publicaciones/Gallardo-et-al-2009a.pdf) | ATGCAGCCTACCCACCACGCAGAGGTAAGCTTTCAGGATAGAGATACAGCTCTTCCAGATGCATGTTCATCCATATCTGATATTACCCCCATTACTTATCCGATCACATTACCTATTATTAAAAACATTTCCGTTACTGCTCACGGTATCAATCTTATCGATAAATTTCCATCAAAGTTCTGCAGCTCTTACATACCCTTCCACTACGGAGGCAATTCGATTAAAACCCCCGACGATCCGGGCGCGATGATGATTACCTTTGCTTTGAAACCACGGGAGGAATACCAACCCAGCGGTCATATTAACGTATCCAGAGCAAGAGAATTTTATATTAGCTGGGACACAGATTATGTGGGGTCTATCACCACGGCTGATCTTGTGGTATCGGCATCCGCTATTAACTT | FJ174341 | [Gallardo et al., 2009](http://asf-referencelab.info/asf/images/files/publicaciones/Gallardo_et_al_2011b_Uganda.pdf) | AGTGCGTATACCTGTGCAAGCACTTGTGCAAGCACTTGTGCAAGCACTTGTGCAGACACCAATGTAGACACTTGTGCAAGCACTTGTGCAGACATTTGTGCAGACACCAATGTAGACACTTGTGCAAGCACTTGTGCAGATACTTGTGCAGACACTTGTGTAAGCACTTGTGTAAGCACTTGTGCAGATACTTGTGCAGACACCAATGTAGACACTTGTGCAAGCACTTGTGCAGACACCAATGTAGACACTTGTGTAAGCACTTGTGCAGACACCTGTGCAAGCACAGAATAC |
| 93 | Ug03P.4 | Uganda | DP | 2003 | FJ154431 | IX | [Gallardo et al., 2009](http://asf-referencelab.info/asf/images/files/publicaciones/Gallardo-et-al-2009a.pdf) | ATGCAGCCTACCCACCACGCAGAGGTAAGCTTTCAGGATAGAGATACAGCTCTTCCAGATGCATGTTCATCCATATCTGATATTACCCCCATTACTTATCCGATCACATTACCTATTATTAAAAACATTTCCGTTACTGCTCACGGTATCAATCTTATCGATAAATTTCCATCAAAGTTCTGCAGCTCTTACATACCCTTCCACTACGGAGGCAATTCGATTAAAACCCCCGACGATCCGGGCGCGATGATGATTACCTTTGCTTTGAAACCACGGGAGGAATACCAACCCAGCGGTCATATTAACGTATCCAGAGCAAGAGAATTTTATATTAGCTGGGACACAGATTATGTGGGGTCTATCACCACGGCTGATCTTGTGGTATCGGCATCCGCTATTAACTT | FJ174342 | [Gallardo et al., 2009](http://asf-referencelab.info/asf/images/files/publicaciones/Gallardo_et_al_2011b_Uganda.pdf) | AGTGCGTATACCTGTGCAAGCACTTGTGCAAGCACTTGTGCAAGCACTTGTGCAGACACCAATGTAGACACTTGTGCAAGCACTTGTGCAGACATTTGTGCAGACACCAATGTAGACACTTGTGCAAGCACTTGTGCAGATACTTGTGCAGACACTTGTGTAAGCACTTGTGTAAGCACTTGTGCAGATACTTGTGCAGACACCAATGTAGACACTTGTGCAAGCACTTGTGCAGACACCAATGTAGACACTTGTGTAAGCACTTGTGCAGACACCTGTGCAAGCACAGAATAC |
| 94 | Ug03P.5 | Uganda | DP | 2003 | FJ154432 | IX | [Gallardo et al., 2009](http://asf-referencelab.info/asf/images/files/publicaciones/Gallardo-et-al-2009a.pdf) | ATGCAGCCTACCCACCACGCAGAGGTAAGCTTTCAGGATAGAGATACAGCTCTTCCAGATGCATGTTCATCCATATCTGATATTACCCCCATTACTTATCCGATCACATTACCTATTATTAAAAACATTTCCGTTACTGCTCACGGTATCAATCTTATCGATAAATTTCCATCAAAGTTCTGCAGCTCTTACATACCCTTCCACTACGGAGGCAATTCGATTAAAACCCCCGACGATCCGGGCGCGATGATGATTACCTTTGCTTTGAAACCACGGGAGGAATACCAACCCAGCGGTCATATTAACGTATCCAGAGCAAGAGAATTTTATATTAGCTGGGACACAGATTATGTGGGGTCTATCACCACGGCTGATCTTGTGGTATCGGCATCCGCTATTAACTT | FJ174343 | [Gallardo et al., 2009](http://asf-referencelab.info/asf/images/files/publicaciones/Gallardo_et_al_2011b_Uganda.pdf) | AGTGCGTATACCTGTGCAAGCACTTGTGCAAGCACTTGTGCAAGCACTTGTGCAGACACCAATGTAGACACTTGTGCAAGCACTTGTGCAGACATTTGTGCAGACACCAATGTAGACACTTGTGCAAGCACTTGTGCAGATACTTGTGCAGACACTTGTGTAAGCACTTGTGTAAGCACTTGTGCAGATACTTGTGCAGACACCAATGTAGACACTTGTGCAAGCACTTGTGCAGACACCAATGTAGACACTTGTGTAAGCACTTGTGCAGACACCTGTGCAAGCACAGAATAC |
| 95 | Ug03P.6 | Uganda | DP | 2003 | FJ154433 | IX | [Gallardo et al., 2009](http://asf-referencelab.info/asf/images/files/publicaciones/Gallardo-et-al-2009a.pdf) | ATGCAGCCTACCCACCACGCAGAGGTAAGCTTTCAGGATAGAGATACAGCTCTTCCAGATGCATGTTCATCCATATCTGATATTACCCCCATTACTTATCCGATCACATTACCTATTATTAAAAACATTTCCGTTACTGCTCACGGTATCAATCTTATCGATAAATTTCCATCAAAGTTCTGCAGCTCTTACATACCCTTCCACTACGGAGGCAATTCGATTAAAACCCCCGACGATCCGGGCGCGATGATGATTACCTTTGCTTTGAAACCACGGGAGGAATACCAACCCAGCGGTCATATTAACGTATCCAGAGCAAGAGAATTTTATATTAGCTGGGACACAGATTATGTGGGGTCTATCACCACGGCTGATCTTGTGGTATCGGCATCCGCTATTAACTT | FJ174344 | [Gallardo et al., 2009](http://asf-referencelab.info/asf/images/files/publicaciones/Gallardo_et_al_2011b_Uganda.pdf) | AGTGCGTATACCTGTGCAAGCACTTGTGCAAGCACTTGTGCAAGCACTTGTGCAGACACCAATGTAGACACTTGTGCAAGCACTTGTGCAGACATTTGTGCAGACACCAATGTAGACACTTGTGCAAGCACTTGTGCAGATACTTGTGCAGACACTTGTGTAAGCACTTGTGTAAGCACTTGTGCAGATACTTGTGCAGACACCAATGTAGACACTTGTGCAAGCACTTGTGCAGACACCAATGTAGACACTTGTGTAAGCACTTGTGCAGACACCTGTGCAAGCACAGAATAC |
| 96 | 11/Og/04 | Italy | DP | 2004 | FR668403 | I | [Giammarioli et al 2011](http://asf-referencelab.info/asf/images/files/publicaciones/Gianmaroli_et_al_2011.pdf) | ATGCAGCCTACTCACCACGCAGAGATAAGCTTTCAGGATAGAGATACAGCTCTTCCAGACGCATGTTCATCTATATCGGATATTAGCCCCGTTACGTATCCGATCACATTACCTATTATTAAAAACATTTCCGTAACTGCTCATGGTATCAATCTTATCGATAAGTTTCCATCAAAGTTCTGCAGCTCTTACATACCCTTCCACTACGGAGGCAATGCAATTAAAACCCCCGATGATCCGGGTGCGATGATGATTACCTTTGCTTTGAAGCCACGGGAGGAATACCAACCCAGTGGTCATATTAACGTATCCAGAGCAAGAGAATTTTATATTAGTTGGGACACGGATTACGTGGGGTCTATCACTACGGCTGATCTTGTGGTATCGGCATCTGCTATTAACTT | FR681792 | [Giammarioli et al 2011](http://asf-referencelab.info/asf/images/files/publicaciones/Gianmaroli_et_al_2011.pdf) | AGTGCGTATACCTGTGCAAGCACTTGTGCAGATACCAATGTAGACACCTGTGCAAGCACTTGTGCAAGCACTTGTGCAAGCACTTGTGCAAGCATGTGTGCAGATACCAATGTAGACACCTGTGCAAGCACCTGTGCAAACACCTGTGCAAGCACAGAATAC |
| 97 | 13/Nu/04 | Italy | DP | 2004 | FR668404 | I | [Giammarioli et al 2011](http://asf-referencelab.info/asf/images/files/publicaciones/Gianmaroli_et_al_2011.pdf) | ATGCAGCCTACTCACCACGCAGAGATAAGCTTTCAGGATAGAGATACAGCTCTTCCAGACGCATGTTCATCTATATCGGATATTAGCCCCGTTACGTATCCGATCACATTACCTATTATTAAAAACATTTCCGTAACTGCTCATGGTATCAATCTTATCGATAAGTTTCCATCAAAGTTCTGCAGCTCTTACATACCCTTCCACTACGGAGGCAATGCAATTAAAACCCCCGATGATCCGGGTGCGATGATGATTACCTTTGCTTTGAAGCCACGGGAGGAATACCAACCCAGTGGTCATATTAACGTATCCAGAGCAAGAGAATTTTATATTAGTTGGGACACGGATTACGTGGGGTCTATCACTACGGCTGATCTTGTGGTATCGGCATCTGCTATTAACTT | FR681793 | [Giammarioli et al 2011](http://asf-referencelab.info/asf/images/files/publicaciones/Gianmaroli_et_al_2011.pdf) | ATGCCTGTGCAAGCACTTGTGCAGATACCAATGTAGACACCTGTGCAAGCACTTGTGCAAGCACTTGTGCAAGCACTTGTGCAAGCATGTGTGCAGATACCAATGTAGACACCTGTGCAAGCACCTGTGCAAACACCTGTGCAAGCACAGAATACACCGATTTAC |
| 98 | 16/Og/04 | Italy | DP | 2004 | FR682502 | I | [Giammarioli et al 2011](http://asf-referencelab.info/asf/images/files/publicaciones/Gianmaroli_et_al_2011.pdf) | ATGCAGCCTACTCACCACGCAGAGATAAGCTTTCAGGATAGAGATACAGCTCTTCCAGACGCATGTTCATCTATATCGGATATTAGCCCCGTTACGTATCCGATCACATTACCTATTATTAAAAACATTTCCGTAACTGCTCATGGTATCAATCTTATCGATAAGTTTCCATCAAAGTTCTGCAGCTCTTACATACCCTTCCACTACGGAGGCAATGCAATTAAAACCCCCGATGATCCGGGTGCGATGATGATTACCTTTGCTTTGAAGCCACGGGAGGAATACCAACCCAGTGGTCATATTAACGTATCCAGAGCAAGAGAATTTTATATTAGTTGGGACACGGATTACGTGGGGTCTATCACTACGGCTGATCTTGTGGTATCGGCATCTGCTATTAACTT | FR681794 | [Giammarioli et al 2011](http://asf-referencelab.info/asf/images/files/publicaciones/Gallardo_et_al_2011b_Uganda.pdf) | AGTGCGTATACCTGTGCAAGCACTTGTGCAGATACCAATGTAGACACCTGTGCAAGCACTTGTGCAAGCACTTGTGCCAGCACTTGTGCCAGCATGTGTGCAGATACCAATGTAGACACCTGTGCAAGCACCTGTGCAAACACCTGTGCAAGCACAGAATAC |
| 99 | 18/Nu/04 | Italy | DP | 2004 | FR677326 | I | [Giammarioli et al 2011](http://asf-referencelab.info/asf/images/files/publicaciones/Gianmaroli_et_al_2011.pdf) | ATGCAGCCTACTCACCACGCAGAGATAAGCTTTCAGGATAGAGATACAGCTCTTCCAGACGCATGTTCATCTATATCGGATATTAGCCCCGTTACGTATCCGATCACATTACCTATTATTAAAAACATTTCCGTAACTGCTCATGGTATCAATCTTATCGATAAGTTTCCATCAAAGTTCTGCAGCTCTTACATACCCTTCCACTACGGAGGCAATGCAATTAAAACCCCCGATGATCCGGGTGCGATGATGATTACCTTTGCTTTGAAGCCACGGGAGGAATACCAACCCAGTGGTCATATTAACGTATCCAGAGCAAGAGAATTTTATATTAGTTGGGACACGGATTACGTGGGGTCTATCACTACGGCTGATCTTGTGGTATCGGCATCTGCTATTAACTT | FR681795 | [Giammarioli et al 2011](http://asf-referencelab.info/asf/images/files/publicaciones/Gallardo_et_al_2011b_Uganda.pdf) | AGTGCGTATACCTGTGCGAGCACTTGTGCAGATACCAATGTAGACACCTGTGCAAGCACTTGTGCAAGCACTTGTGCAAGCACTTGTGCAAGCATGTGTGCAGATACCAATGTAGACACCTGTGCAAGCACCTGTGCAAACACCTGTGCAAGCACAGAATAC |
| 100 | 22/Nu/04 | Italy | DP | 2004 | FR668405 | I | [Giammarioli et al 2011](http://asf-referencelab.info/asf/images/files/publicaciones/Gallardo-et-al-2009a.pdf) | ATGCAGCCTACTCACCACGCAGAGATAAGCTTTCAGGATAGAGATACAGCTCTTCCAGACGCATGTTCATCTATATCGGATATTAGCCCCGTTACGTATCCGATCACATTACCTATTATTAAAAACATTTCCGTAACTGCTCATGGTATCAATCTTATCGATAAGTTTCCATCAAAGTTCTGCAGCTCTTACATACCCTTCCACTACGGAGGCAATGCAATTAAAACCCCCGATGATCCGGGTGCGATGATGATTACCTTTGCTTTGAAGCCACGGGAGGAATACCAACCCAGTGGTCATATTAACGTATCCAGAGCAAGAGAATTTTATATTAGTTGGGACACGGATTACGTGGGGTCTATCACTACGGCTGATCTTGTGGTATCGGCATCTGCTATTAACTT | FR681796 | [Giammarioli et al 2011](http://asf-referencelab.info/asf/images/files/publicaciones/Gallardo_et_al_2011b_Uganda.pdf) | AGTGCGTATACCTGTGCAAGCACTTGTGCAGATACCAATGTAGACACCTGTGCAAGCACTTGTGCAAGCACTTGTGCAAGCACTTGTGCAAGCATGTGTGCAGATACCAATGTAGACACCTGTGCAAGCACCTGTGCAAACACCTGTGCAAGCACAGAATAC |
| 101 | 23/Or/04 | Italy | DP | 2004 | FR668406 | I | [Giammarioli et al 2011](http://asf-referencelab.info/asf/images/files/publicaciones/Gallardo-et-al-2009a.pdf) | ATGCAGCCCACTCACCACGCAGAGATAAGCTTTCAGGAAAGAGATACAGCTCTTCCAGACGCATGTTCATCCATATCGGATATTAGCCCCGTTACGTATCCGATCACATTACCTATTATTAAAAACATTTTCGTAACTGCTCATGGTATCAATCTTATCGATACGTTTCCATCAAAGTTCTGCAGCTCTTACATACACTTCCACTACGGAGGCAATGCAATTAAAACCCCCGATGATCCGGGTGCGATGATGATTACCTTTGCTTTGAAGCCACGGGAGGAATACCAACCCAGTGGTCATATTAACGTATCCAGAGCAAGAGAATTTTATATTAGTTGGGACACGGATTACGTGGGGTCTATCACTACGGCTGATCTTGTGGTATCGGCATCTGCTATTAACTT | FR681797 | [Giammarioli et al 2011](http://asf-referencelab.info/asf/images/files/publicaciones/Gallardo_et_al_2011b_Uganda.pdf) | AGTGCGTATACCTGTGCAAGCACTTGTGCAGATACCAATGTAGACACCTGTGCAAGCACTTGTGCAAGCACTTGTGCAAGCACTTGTGCAAGCATGTGTGCAGATACCAATGTAGACACCTGTGCAAGCACCTGTGCAAACACCTGTGCAAGCACAGAATAC |
| 102 | 24/Or/04 | Italy | DP | 2004 | FR668407 | I | [Giammarioli et al 2011](http://asf-referencelab.info/asf/images/files/publicaciones/Gallardo-et-al-2009a.pdf) | ATGCAGCCCACTCACCACGCAGAGATAAGCTTTCAGGATAGAGATACAGCTCTTCCAGACGCATGTTCATCTATATCGGATATTAGCCCCGTTACGTATCCGATCACATTACCTATTATTAAAAACATCTCCGTAACTGCTCATGGTATCAATCTTATCGATAAGTTTCCATCAAAGTTCTGCAGCTCTTACATACCCTTCCACTACGGAGGCAATGCAATTAAAACCCCCGATGATCCGGGTGCGATGATGATTACCTTTGCTTTGAAGCCACGGGAGGAATACCAACCCAGTGGTCATATTAACGTATCCAGAGCAAGAGAATTTTATATTAGTTGGGACACGGATTACGTGGGGTCTATCACTACGGCTGATCTTGTGGTATCGGCATCTGCTATTAACTT | FR681798 | [Giammarioli et al 2011](http://asf-referencelab.info/asf/images/files/publicaciones/Gallardo_et_al_2011b_Uganda.pdf) | AGTGCGTATACCTGTGCAAGCACTTGTGCAGATACCAATGTAGACACCTGTGCAAGCACTTGTGCAAGCACTTGTGCAAGCACTTGTGCAAGCATGTGTGCAGATACCAATGTAGACACCTGTGCAAGCACCTGTGCAAACACCTGTGCAAGCACAGAATAC |
| 103 | 25/NU/04 | Italy | DP | 2004 | FR668408 | I | [Giammarioli et al 2011](http://asf-referencelab.info/asf/images/files/publicaciones/Gallardo-et-al-2009a.pdf) | ATGCAGCCTACTCACCACGCAGAGATAAGCTTTCAGGATAGAGATACAGCTCTTCCAGACGCATGTTCATCTATATCGGATATTAGCCCCGTTACGTATCCGATCACATTACCTATTATTAAAAACATTTCCGTAACTGCTCATGGTATCAATCTTATCGATAAGTTTCCATCAAAGTTCTGCAGCTCTTACATACCCTTCCACTACGGAGGCAATGCAATTAAAACCCCCGATGATCCGGGTGCGATGATGATTACCTTTGCTTTGAAGCCACGGGAGGAATACCAACCCAGTGGTCATATTAACGTATCCAGAGCAAGAGAATTTTATATTAGTTGGGACACGGATTACGTGGGGTCTATCACTACGGCTGATCTTGTGGTATCGGCATCTGCTATTAACTT | FR686540 | [Giammarioli et al 2011](http://asf-referencelab.info/asf/images/files/publicaciones/Gallardo_et_al_2011b_Uganda.pdf) | AGTGCGTATACCTGTGCAAGCACTTGTGCAGATACCAATGTAGACACCTGTGCAAGCACTTGTGCAAGCACTTGTGCAAGCACTTGTGCAAGCATGTGTGCAGATACCAATGTAGACACCTGTGCAAGCACCTGTGCAAACACCTGTGCAAGCACAGAATAC |
| 104 | 26/Ss/04 | Italy | DP | 2004 | FR668409 | I | [Giammarioli et al 2011](http://asf-referencelab.info/asf/images/files/publicaciones/Gallardo-et-al-2009a.pdf) | ATGCAGCCTACTCACCACGCAGAGATAAGCTTTCAGGATAGAGATACAGCTCTTCCAGACGCATGTTCATCTATATCGGATATTAGCCCCGTTACGTATCCGATCACATTACCTATTATTAAAAACATTTCCGTAACTGCTCATGGTATCAATCTTATCGATAAGTTTCCATCAAAGTTCTGCAGCTCTTACATACCCTTCCACTACGGAGGCAATGCAATTAAAACCCCCGATGATCCGGGTGCGATGATGATTACCTTTGCTTTGAAGCCACGGGAGGAATACCAACCCAGTGGTCATATTAACGTATCCAGAGCAAGAGAATTTTATATTAGTTGGGACACGGATTACGTGGGGTCTATCACTACGGCTGATCTTGTGGTATCGGCATCTGCTATTAACTT | FR686541 | [Giammarioli et al 2011](http://asf-referencelab.info/asf/images/files/publicaciones/Gallardo_et_al_2011b_Uganda.pdf) | AGTGCGTATACCTGTGCAAGCACTTGTGCAGATACCAATGTAGACACCTGTGCAAGCACTTGTGCAAGCACTTGTGCAAGCACTTGTGCAAGCATGTGTGCAGATACCAATGTAGACACCTGTGCAAGCACCTGTGCAAACACCTGTGCAAGCACAGAATAC |
| 105 | 30/Ol/04 | Italy | DP | 2004 | FR668410 | I | [Giammarioli et al 2011](http://asf-referencelab.info/asf/images/files/publicaciones/Gallardo-et-al-2009a.pdf) | ATGCAGCCTACTCACCACGCAGAGATAAGCTTTCAGGATAGAGATACAGCTCTTCCAGACGCATGTTCATCTATATCGGATATTAGCCCCGTTACGTATCCGATCACATTACCTATTATTAAAAACATTTCCGTAACTGCTCATGGTATCAATCTTATCGATAAGTTTCCATCAAAGTTCTGCAGCTCTTACATACCCTTCCACTACGGAGGCAATGCAATTAAAACCCCCGATGATCCGGGTGCGATGATGATTACCTTTGCTTTGAAGCCACGGGAGGAATACCAACCCAGTGGTCATATTAACGTATCCAGAGCAAGAGAATTTTATATTAGTTGGGACACGGATTACGTGGGGTCTATCACTACGGCTGATCTTGTGGTATCGGCATCTGCTATTAACTT | FR686542 | [Giammarioli et al 2011](http://asf-referencelab.info/asf/images/files/publicaciones/Gallardo_et_al_2011b_Uganda.pdf) | AGTGCGTATACCTGTGCAAGCACTTGTGCAGATACCAATGTAGACACCTGTGCAAGCACTTGTGCAAGCACTTGTGCAAGCACTTGTGCAAGCATGTGTGCAGATACCAATGTAGACACCTGTGCAAGCACCTGTGCAAACACCTGTGCAAGCACAGAATAC |
| 106 | Ca04.1 | Italy | DP | 2004 | FR668270 | I | [Giammarioli et al 2011](http://asf-referencelab.info/asf/images/files/publicaciones/Gianmaroli_et_al_2011.pdf) | ATGCAGCCTACTCACCACGCAGAGATAAGCTTTCAGGATAGAGATACAGCTCTTCCAGACGCATGTTCATCTATATCGGATATTAGCCCCGTTACGTATCCGATCACATTACCTATTATTAAAAACATTTCCGTAACTGCTCATGGTATCAATCTTATCGATAAGTTTCCATCAAAGTTCTGCAGCTCTTACATACCCTTCCACTACGGAGGCAATGCAATTAAAACCCCCGATGATCCGGGTGCGATGATGATTACCTTTGCTTTGAAGCCACGGGAGGAATACCAACCCAGTGGTCATATTAACGTATCCAGAGCAAGAGAATTTTATATTAGTTGGGACACGGATTACGTGGGGTCTATCACTACGGCTGATCTTGTGGTATCGGCATCTGCTATTAACTT | FR668272 | [Giammarioli et al 2011](http://asf-referencelab.info/asf/images/files/publicaciones/Gallardo_et_al_2011b_Uganda.pdf) | AGTGCGTATACCTGTGCAAGCACTTGTGCAGATACCAATGTAGACACCTGTGCAAGCACTTGTGCAAGCACTTGTGCAAGCACTTGTGCAAGCATGTGTGCAGATACCAATGTAGACACCTGTGCAAGCACCTGTGCAAACACCTGTGCAAGCACAGAATAC |
| 107 | Nu04.3 | Italy | DP | 2004 | FR668262 | I | [Giammarioli et al 2011](http://asf-referencelab.info/asf/images/files/publicaciones/Gallardo-et-al-2009a.pdf) | ATGCAGCCTACTCACCACGCAGAGATAAGCTTTCAGGATAGAGATACAGCTCTTCCAGACGCATGTTCATCTATATCGGATATTAGCCCCGTTACGTATCCGATCACATTACCTATTATTAAAAACATTTCCGTAACTGCTCATGGTATCAATCTTATCGATAAGTTTCCATCAAAGTTCTGCAGCTCTTACATACCCTTCCACTACGGAGGCAATGCAATTAAAACCCCCGATGATCCGGGTGCGATGATGATTACCTTTGCTTTGAAGCCACGGGAGGAATACCAACCCAGTGGTCATATTAACGTATCCAGAGCAAGAGAATTTTATATTAGTTGGGACACGGATTACGTGGGGTCTATCACTACGGCTGATCTTGTGGTATCGGCATCTGCTATTAACTT | FR668255 | [Giammarioli et al 2011](http://asf-referencelab.info/asf/images/files/publicaciones/Gallardo_et_al_2011b_Uganda.pdf) | AGTGCGTATACCTGTGCAAGCACTTGTGCAGATACCAATGTAGACACCTGTGCAAGCACTTGTGCAAGCACTTGTGCAAGCACTTGTGCAAGCATGTGTGCAGATACCAATGTAGACACCTGTGCAAGCACCTGTGCAAACACCTGTGCAAGCACAGAATAC |
| 108 | Nu04.4 | Italy | DP | 2004 | FR668263 | I | [Giammarioli et al 2011](http://asf-referencelab.info/asf/images/files/publicaciones/Gallardo-et-al-2009a.pdf) | ATGCAGCCTACTCACCACGCAGAGATAAGCTTTCAGGATAGAGATACAGCTCTTCCAGACGCATGTTCATCTATATCGGATATTAGCCCCGTTACGTATCCGATCACATTACCTATTATTAAAAACATTTCCGTAACTGCTCATGGTATCAATCTTATCGATAAGTTTCCATCAAAGTTCTGCAGCTCTTACATACCCTTCCACTACGGAGGCAATGCAATTAAAACCCCCGATGATCCGGGTGCGATGATGATTACCTTTGCTTTGAAGCCACGGGAGGAATACCAACCCAGTGGTCATATTAACGTATCCAGAGCAAGAGAATTTTATATTAGTTGGGACACGGATTACGTGGGGTCTATCACTACGGCTGATCTTGTGGTATCGGCATCTGCTATTAACTT | FR668256 | [Giammarioli et al 2011](http://asf-referencelab.info/asf/images/files/publicaciones/Gallardo_et_al_2011b_Uganda.pdf) | AGTGCGTATACCTGTGCAAGCACTTGTGCAGATACCAATGTAGACACCTGTGCAAGCACTTGTGCAAGCACTTGTGCAAGCACTTGTGCAAGCATGTGTGCAGATACCAATGTAGACACCTGTGCAAGCACCTGTGCAAACACCTGTGCAAGCACAGAATAC |
| 109 | Nu04.6a | Italy | DP | 2004 | FR668264 | I | [Giammarioli et al 2011](http://asf-referencelab.info/asf/images/files/publicaciones/Gallardo-et-al-2009a.pdf) | ATGCAGCCTACTCACCACGCAGAGATAAGCTTTCAGGATAGAGATACAGCTCTTCCAGACGCATGTTCATCTATATCGGATATTAGCCCCGTTACGTATCCGATCACATTACCTATTATTAAAAACATTTCCGTAACTGCTCATGGTATCAATCTTATCGATAAGTTTCCATCAAAGTTCTGCAGCTCTTACATACCCTTCCACTACGGAGGCAATGCAATTAAAACCCCCGATGATCCGGGTGCGATGATGATTACCTTTGCTTTGAAGCCACGGGAGGAATACCAACCCAGTGGTCATATTAACGTATCCAGAGCAAGAGAATTTTATATTAGTTGGGACACGGATTACGTGGGGTCTATCACTACGGCTGATCTTGTGGTATCGGCATCTGCTATTAACTT | FR668257 | [Giammarioli et al 2011](http://asf-referencelab.info/asf/images/files/publicaciones/Gallardo_et_al_2011b_Uganda.pdf) | AGTGCGTATACCTGTGCAAGCACTTGTGCAGATACCAATGTAGACACCTGTGCAAGCACTTGTGCAAGCACTTGTGCAAGCACTTGTGCAAGCATGTGTGCAGATACCAATGTAGACACCTGTGCAAGCACCTGTGCAAACACCTGTGCAAGCACAGAATAC |
| 110 | Nu04.6b | Italy | DP | 2004 | FR668265 | I | [Giammarioli et al 2011](http://asf-referencelab.info/asf/images/files/publicaciones/Gallardo-et-al-2009a.pdf) | ATGCAGCCTACTCACCACGCAGAGATAAGCTTTCAGGATAGAGATACAGCTCTTCCAGACGCATGTTCATCTATATCGGATATTAGCCCCGTTACGTATCCGATCACATTACCTATTATTAAAAACATTTCCGTAACTGCTCATGGTATCAATCTTATCGATAAGTTTCCATCAAAGTTCTGCAGCTCTTACATACCCTTCCACTACGGAGGCAATGCAATTAAAACCCCCGATGATCCGGGTGCGATGATGATTACCTTTGCTTTGAAGCCACGGGAGGAATACCAACCCAGTGGTCATATTAACGTATCCAGAGCAAGAGAATTTTATATTAGTTGGGACACGGATTACGTGGGGTCTATCACTACGGCTGATCTTGTGGTATCGGCATCTGCTATTAACTT | FR668258 | [Giammarioli et al 2011](http://asf-referencelab.info/asf/images/files/publicaciones/Gallardo_et_al_2011b_Uganda.pdf) | AGTGCGTATACCTGTGCAAGCACTTGTGCAGATACCAATGTAGACACCTGTGCAAGCACTTGTGCAAGCACTTGTGCAAGCACTTGTGCAAGCATGTGTGCAGATACCAATGTAGACACCTGTGCAAGCACCTGTGCAAACACCTGTGCAAGCACAGAATAC |
| 111 | Nu04WB | Italy | Wild pig | 2004 | FR668266 | I | [Giammarioli et al 2011](http://asf-referencelab.info/asf/images/files/publicaciones/Gallardo-et-al-2009a.pdf) | ATGCAGCCTACTCACCACGCAGAGATAAGCTTTCAGGATAGAGATACAGCTCTTCCAGACGCATGTTCATCTATATCGGATATTAGCCCCGTTACGTATCCGATCACATTACCTATTATTAAAAACATTTCCGTAACTGCTCATGGTATCAATCTTATCGATAAGTTTCCATCAAAGTTCTGCAGCTCTTACATACCCTTCCACTACGGAGGCAATGCAATTAAAACCCCCGATGATCCGGGTGCGATGATGATTACCTTTGCTTTGAAGCCACGGGAGGAATACCAACCCAGTGGTCATATTAACGTATCCAGAGCAAGAGAATTTTATATTAGTTGGGACACGGATTACGTGGGGTCTATCACTACGGCTGATCTTGTGGTATCGGCATCTGCTATTAACTT | FR676962 | [Giammarioli et al 2011](http://asf-referencelab.info/asf/images/files/publicaciones/Gallardo_et_al_2011b_Uganda.pdf) | AGTGCGTATACCTGTGCAAGCACTTGTGCAGATACCAATGTAGACACCTGTGCAAGCACTTGTGCAAGCACTTGTGCAAGCACTTGTGCAAGCATGTGTGCAGATACCAATGTAGACACCTGTGCAAGCACCTGTGCAAACACCTGTGCAAGCACAGAATAC |
| 112 | Ss04.10 | Italy | DP | 2004 | FR668266 | I | [Giammarioli et al 2011](http://asf-referencelab.info/asf/images/files/publicaciones/Gallardo-et-al-2009a.pdf) | ATGCAGCCTACTCACCACGCAGAGATAAGCTTTCAGGATAGAGATACAGCTCTTCCAGACGCATGTTCATCTATATCGGATATTAGCCCCGTTACGTATCCGATCACATTACCTATTATTAAAAACATTTCCGTAACTGCTCATGGTATCAATCTTATCGATAAGTTTCCATCAAAGTTCTGCAGCTCTTACATACCCTTCCACTACGGAGGCAATGCAATTAAAACCCCCGATGATCCGGGTGCGATGATGATTACCTTTGCTTTGAAGCCACGGGAGGAATACCAACCCAGTGGTCATATTAACGTATCCAGAGCAAGAGAATTTTATATTAGTTGGGACACGGATTACGTGGGGTCTATCACTACGGCTGATCTTGTGGTATCGGCATCTGCTATTAACTT | FR668259 | [Giammarioli et al 2011](http://asf-referencelab.info/asf/images/files/publicaciones/Nix_et_al_2006.pdf) | AGTGCGTATACCTGTGCAAGCACTTGTGCAGATACCAATGTAGACACCTGTGCAAGCACTTGTGCAAGCACTTGTGCAAGCACTTGTGCAAGCATGTGTGCAGATACCAATGTAGACACCTGTGCAAGCACCTGTGCAAACACCTGTGCAAGCACAGAATAC |
| 113 | 36/Ss/05 | Italy | DP | 2005 | FR668411 | I | [Giammarioli et al 2011](http://asf-referencelab.info/asf/images/files/publicaciones/Gallardo-et-al-2009a.pdf) | ATGCAGCCTACTCACCACGCAGAGATAAGCTTTCAGGATAGAGATACAGCTCTTCCAGACGCATGTTCATCTATATCGGATATTAGCCCCGTTACGTATCCGATCACATTACCTATTATTAAAAACATTTCCGTAACTGCTCATGGTATCAATCTTATCGATAAGTTTCCATCAAAGTTCTGCAGCTCTTACATACCCTTCCACTACGGAGGCAATGCAATTAAAACCCCCGATGATCCGGGTGCGATGATGATTACCTTTGCTTTGAAGCCACGGGAGGAATACCAACCCAGTGGTCATATTAACGTATCCAGAGCAAGAGAATTTTATATTAGTTGGGACACGGATTACGTGGGGTCTATCACTACGGCTGATCTTGTGGTATCGGCATCTGCTATTAACTT | FR686543 | [Giammarioli et al 2011](http://asf-referencelab.info/asf/images/files/publicaciones/Nix_et_al_2006.pdf) | AGTGCGTATACCTGTGCAAGCACTTGTGCAGATACCAATGTAGACACCTGTGCAAGCACTTGTGCAAGCACTTGTGCAAGCACTTGTGCAAGCATGTGTGCAGATACCAATGTAGACACCTGTGCAAGCACCTGTGCAAACACCTGTGCAAGCACAGAATAC |
| 114 | GH05/ASOb24 | Ghana | DP | 2005 |  | I | [This study (gallardo@inia.es)](http://asf-referencelab.info/asf/images/files/publicaciones/Gallardo-et-al-2009a.pdf) | ATGCAGCCTACTCACCACGCAGAGATAAGCTTTCAGGATAGAGATACAGCTCTTCCAGACGCATGTTCATCTATATCGGATATTAGCCCCGTTACGTATCCGATCACATTACCTATTATTAAAAACATTTCCGTAACTGCTCATGGTATCAATCTTATCGATAAGTTTCCATCAAAGTTCTGCAGCTCTTACATACCCTTCCACTACGGAGGCAATGCAATTAAAACCCCCGATGATCCGGGTGCGATGATGATTACCTTTGCTTTGAAGCCACGGGAGGAATACCAACCCAGTGGTCATATTAACGTATCCAGAGCAAGAGAATTTTATATTAGTTGGGACACGGATTACGTGGGGTCTATCACTACGGCTGATCTTGTGGTATCGGCATCTGCTATTAACTT |  | [This study (gallardo@inia.es)](http://asf-referencelab.info/asf/images/files/publicaciones/Nix_et_al_2006.pdf) | AGTGCGTATACTTGTGCAAGCACTTGTGCAGATACCAATGTAGACACCTGTGCAAGCACTTGTGCAAGCACTTGTGCAAGCACTTGTGCAAGCACTTGTGCAAGCACAGGTGCAAGCACTTGTGCAGATACCAATGTAGACACCTGTGCAAGCACTTGTGCAAGCACTTGTGCAAGCACTTGTGCAAGCACTTGTGCAAGCACAGGTGCAAGCACTTGTGCAGATACCAATGTAGACACCTGTGCAAGCACTTGTGCAAGCACTTGTGCAAGCACTTGTGCAAGCACTTGTGCAAGCACTTGTGCAAGCACAGGTGCAAGCACTTGTGCAGATACCAATGTAGACACCTGTGCAAGCACTTGTGCAAGCACTTGTGCAAGCACTTGTGCAAGCACTTGTGCAAGCACTTGTGCAAGCACAGGTGCAAGCACTTGTGCAGATACCAATGTAGACACCTGTGCAAGCACTTGTGCAAGCACTTGTGCAAGCACTTGTGCAAGCACTTGTGCAAGCACTTGTGCAAGCACAGGTGCAAGCACTTGTGCAGATACCAATGTAGACACCTGTGCAAGCACCTGTGCAAACACCTGTGCAAGCACAGAATAC |
| 115 | Ss05.3a | Italy | DP | 2005 | FR668267 | I | [Giammarioli et al 2011](http://asf-referencelab.info/asf/images/files/publicaciones/Gallardo-et-al-2009a.pdf) | ATGCAGCCTACTCACCACGCAGAGATAAGCTTTCAGGATAGAGATACAGCTCTTCCAGACGCATGTTCATCTATATCGGATATTAGCCCCGTTACGTATCCGATCACATTACCTATTATTAAAAACATTTCCGTAACTGCTCATGGTATCAATCTTATCGATAAGTTTCCATCAAAGTTCTGCAGCTCTTACATACCCTTCCACTACGGAGGCAATGCAATTAAAACCCCCGATGATCCGGGTGCGATGATGATTACCTTTGCTTTGAAGCCACGGGAGGAATACCAACCCAGTGGTCATATTAACGTATCCAGAGCAAGAGAATTTTATATTAGTTGGGACACGGATTACGTGGGGTCTATCACTACGGCTGATCTTGTGGTATCGGCATCTGCTATTAACTT | FR668260 | [Giammarioli et al 2011](http://asf-referencelab.info/asf/images/files/publicaciones/Nix_et_al_2006.pdf) | AGTGCGTATACCTGTGCAAGCACTTGTGCAGATACCAATGTAGACACCTGTGCAAGCACTTGTGCAAGCACTTGTGCAAGCACTTGTGCAAGCATGTGTGCAGATACCAATGTAGACACCTGTGCAAGCACCTGTGCAAACACCTGTGCAAGCACAGAATAC |
| 116 | Ss05.3b | Italy | DP | 2005 | FR668268 | I | [Giammarioli et al 2011](http://asf-referencelab.info/asf/images/files/publicaciones/Gallardo-et-al-2009a.pdf) | ATGCAGCCTACTCACCACGCAGAGATAAGCTTTCAGGATAGAGATACAGCTCTTCCAGACGCATGTTCATCTATATCGGATATTAGCCCCGTTACGTATCCGATCACATTACCTATTATTAAAAACATTTCCGTAACTGCTCATGGTATCAATCTTATCGATAAGTTTCCATCAAAGTTCTGCAGCTCTTACATACCCTTCCACTACGGAGGCAATGCAATTAAAACCCCCGATGATCCGGGTGCGATGATGATTACCTTTGCTTTGAAGCCACGGGAGGAATACCAACCCAGTGGTCATATTAACGTATCCAGAGCAAGAGAATTTTATATTAGTTGGGACACGGATTACGTGGGGTCTATCACTACGGCTGATCTTGTGGTATCGGCATCTGCTATTAACTT | FR668261 | [Giammarioli et al 2011](http://asf-referencelab.info/asf/images/files/publicaciones/Nix_et_al_2006.pdf) | AGTGCGTATACCTGTGCAAGCACTTGTGCAGATACCAATGTAGACACCTGTGCAAGCACTTGTGCAAGCACTTGTGCAAGCACTTGTGCAAGCATGTGTGCAGATACCAATGTAGACACCTGTGCAAGCACCTGTGCAAACACCTGTGCAAGCACAGAATAC |
| 117 | Ken05/Tk1 | Kenya | Tick | 2005 | HM745253 | X | [Gallardo et al 2011](http://asf-referencelab.info/asf/images/files/publicaciones/Gallardo-et-al-2009a.pdf) | ATGCAGCCTACCCACCACGCAGAGGTAAGCTTTCAGGATAGAGATACAGCTCTTCCAGATGCATGTTCATCCATATCTGATATTACCCCCATTACTTATCCGATCACATTACCTATTATTAAAAACATTTCCGTCACTGCTCATGGTATCAATCTTATCGATAAATTTCCATCAAAGTTCTGCAGCTCTTACATACCCTTTCACTACGGAGGCAATTCGATTAAAACCCCCGACGATCCGGGCGCGATGATGATTACCTTTGCTTTGAAACCACGGGAGGAATACCAACCCAGTGGTCATATTAACGTATCCAGAGCAAGAGAATTTTATATTAGCTGGGACACAGATTATGTGGGGTCTATCACCACGGCCGATCTTGTGGTATCGGCATCCGCTATTAACTT | HM745288 | [Gallardo et al 2011](http://asf-referencelab.info/asf/images/files/publicaciones/Nix_et_al_2006.pdf) | AGTGCGTATACCTGTGCAAGCACCTGTGCAAGCACCTGTGCAGGCACCAATGTAGACACTTGTGCAAGCACTTGTGTAAGCACCTGTGCAGACACTTGTGCAGACACCTGTGCAAGCACAGAATAC |
| 118 | Ken05/Tk10 | Kenya | Tick | 2005 | HM745262 | X | [Gallardo et al 2011](http://asf-referencelab.info/asf/images/files/publicaciones/Gallardo-et-al-2009a.pdf) | ATGCAGCCTACCCACCACGCAGAGGTAAGCTTTCAGGATAGAGATACAGCTCTTCCAGATGCATGTTCATCCATATCAGATATTACCCCCATTACTTATCCGATCACGTTACCTATTATTAAAAACATTTCCGTCACTGCTCATGGTATCAATCTTATCGATAAATTTCCATCAAAGTTCTGCAGCTCTTACATACCCTTTCACTACGGAGGCAATTCGATTAAAACCCCCGACGATCCGGGCGCGATGATGATTACCTTTGCTTTGAAACCACGGGAGGAATACCAACCCAGTGGTCATATTAACGTATCCAGAGCAAGAGAATTTTATATTAGCTGGGACACAGATTATGTGGGGTCTATCACCACGGCCGATCTTGTGGTATCGGCATCCGCTATTAACTT | HM745297 | [Gallardo et al 2011](http://asf-referencelab.info/asf/images/files/publicaciones/Nix_et_al_2006.pdf) | AGTGCGTATACCTGTGCAAGCACCTGTGCAAGCACCTGTGCAGACACCAATGTAGACACTTGTGCAAGCACTTGTGTAAGCACCTGTGCAGACACTTGTGCAGACACCTGTGCAAGCACAGAATAC |
| 119 | Ken05/Tk2 | Kenya | Tick | 2005 | HM745254 | X | [Gallardo et al 2011](http://asf-referencelab.info/asf/images/files/publicaciones/Gallardo-et-al-2009a.pdf) | ATGCAGCCTACCCACCACGCAGAGGTAAGCTTTCAGGATAGAGATACAGCTCTTCCAGATGCATGTTCATCCATATCTGATATTACCCCCATTACTTATCCGATCACATTACCTATTATTAAAAACATTTCCGTCACTGCTCATGGTATCAATCTTATCGATAAATTTCCATCAAAGTTCTGCAGCTCTTACATACCCTTTCACTACGGAGGCAATTCGATTAAAACCCCCGACGATCCGGGCGCGATGATGATTACCTTTGCTTTGAAACCACGGGAGGAATACCAACCCAGTGGTCATATTAACGTATCCAGAGCAAGAGAATTTTATATTAGCTGGGACACAGATTATGTGGGGTCTATCACCACGGCCGATCTTGTGGTATCGGCATCCGCTATTAACTT | HM745289 | [Gallardo et al 2011](http://asf-referencelab.info/asf/images/files/publicaciones/Nix_et_al_2006.pdf) | AGTGCGTATACCTGTGCAGACACCAATGTAGACACTTGTGCAAGCACTTGTGCAAGCACCAATGTAGACACTTGTGCAAGCACTTGTACAGACACTTGCGCAAGCACCTGTGCAAGCACAGAATAC |
| 120 | Ken05/Tk3 | Kenya | Tick | 2005 | HM745255 | X | [Gallardo et al 2011](http://asf-referencelab.info/asf/images/files/publicaciones/Gallardo-et-al-2009a.pdf) | ATGCAGCCTACCCACCACGCAGAGGTAAGCTTTCAGGATAGAGATACAGCTCTTCCAGATGCATGTTCATCCATATCTGATATTACCCCCATTACTTATCCGATCACATTACCTATTATTAAAAACATTTCCGTCACTGCTCATGGTATCAATCTTATCGATAAATTTCCATCAAAGTTCTGCAGCTCTTACATACCCTTTCACTACGGAGGCAATTCGATTAAAACCCCCGACGATCCGGGCGCGATGATGATTACCTTTGCTTTGAAACCACGGGAGGAATACCAACCCAGTGGTCATATTAACGTATCCAGAGCAAGAGAATTTTATATTAGCTGGGACACAGATTATGTGGGGTCTATCACCACGGCCGATCTTGTGGTATCGGCATCCGCTATTAACTT | HM745290 | [Gallardo et al 2011](http://asf-referencelab.info/asf/images/files/publicaciones/Nix_et_al_2006.pdf) | AGTGCGTATACCTGTGCAAGCACCTGTGCAAGCACCTGTGCAAGCACCAATGTAGACACTTGTGCAAGCACTTGTGTAAGCACCTGTGCAGACACTTGTGCAGACACCTGTGCAAGCACAGAATAC |
| 121 | Ken05/Tk4 | Kenya | Tick | 2005 | HM745256 | X | [Gallardo et al 2011](http://asf-referencelab.info/asf/images/files/publicaciones/Gallardo-et-al-2009a.pdf) | ATGCAGCCTACCCACCACGCAGAGGTAAGCTTTCAGGATAGAGATACAGCTCTTCCAGATGCATGTTCATCCATATCTGATATTACCCCCATTACTTATCCGATCACATTACCTATTATTAAAAACATTTCCGTCACTGCTCATGGTATCAATCTTATCGATAAATTTCCATCAAAGTTCTGCAGCTCTTACATACCCTTTCACTACGGAGGCAATTCGATTAAAACCCCCGACGATCCGGGCGCGATGATGATTACCTTTGCTTTGAAACCACGGGAGGAATACCAACCCAGTGGTCATATTAACGTATCCAGAGCAAGAGAATTTTATATTAGCTGGGACACAGATTATGTGGGGTCTATCACCACGGCCGATCTTGTGGTATCGGCATCCGCTATTAACTT | HM745291 | [Gallardo et al 2011](http://asf-referencelab.info/asf/images/files/publicaciones/Nix_et_al_2006.pdf) | AGTGCGTATACCTGTGCAAGCACCTGTGCAAGCACCTGTGCAGGCACCAATGTAGACACTTGTGCAAGCACTTGTGTAAGCACCTGTGCAGACACTTGTGCAGACACCTGTGCAAGCACAGAATAC |
| 122 | Ken05/Tk5 | Kenya | Tick | 2005 | HM745257 | X | [Gallardo et al 2011](http://asf-referencelab.info/asf/images/files/publicaciones/Gallardo-et-al-2009a.pdf) | ATGCAGCCTACCCACCACGCAGAGGTAAGCTTTCAGGATAGAGATACAGCTCTTCCAGATGCATGTTCATCCATATCAGATATTACCCCCATTACTTATCCGATCACGTTACCTATTATTAAAAACATTTCCGTCACTGCTCATGGTATCAATCTTATCGATAAATTTCCATCAAAGTTCTGCAGCTCTTACATACCCTTTCACTACGGAGGCAATTCGATTAAAACCCCCGACGATCCGGGCGCGATGATGATTACCTTTGCTTTGAAACCACGGGAGGAATACCAACCCAGTGGTCATATTAACGTATCCAGAGCAAGAGAATTTTATATTAGCTGGGACACAGATTATGTGGGGTCTATCACCACGGCCGATCTTGTGGTATCGGCATCCGCTATTAACTT | HM745292 | [Gallardo et al 2011](http://asf-referencelab.info/asf/images/files/publicaciones/Nix_et_al_2006.pdf) | AGTGCGTATACCTGTGCAAGCACCTGTGCAAGCACCTGTGCAGACACCAATGTAGACACTTGTGCAAGCACTTGTGTAAGCACCTGTGCAGACACTTGTGCAGACACCTGTGCAAGCACAGAATAC |
| 123 | Ken05/Tk6 | Kenya | Tick | 2005 | HM745258 | X | [Gallardo et al 2011](http://asf-referencelab.info/asf/images/files/publicaciones/Gallardo-et-al-2009a.pdf) | ATGCAGCCTACCCACCACGCAGAGGTAAGCTTTCAGGATAGAGATACAGCTCTTCCAGATGCATGTTCATCCATATCTGATATTACCCCCATTACTTATCCGATCACATTACCTATTATTAAAAACATTTCCGTCACTGCTCATGGTATCAATCTTATCGATAAATTTCCATCAAAGTTCTGCAGCTCTTACATACCCTTTCACTACGGAGGCAATTCGATTAAAACCCCCGACGATCCGGGCGCGATGATGATTACCTTTGCTTTGAAACCACGGGAGGAATACCAACCCAGTGGTCATATTAACGTATCCAGAGCAAGAGAATTTTATATTAGCTGGGACACAGATTATGTGGGGTCTATCACCACGGCCGATCTTGTGGTATCGGCATCCGCTATTAACTT | HM745293 | [Gallardo et al 2011](http://asf-referencelab.info/asf/images/files/publicaciones/Nix_et_al_2006.pdf) | AGTGCGTATACCTGTGCAAGCACCTGTGCAAGCACCTGTGCAAGCACCAATGTAGACACTTGTGCAAGCACTTGTGTAAGCACCTGTGCAGACACTTGTGCAGACACCTGTGCAAGCACAGAATAC |
| 124 | Ken05/Tk7 | Kenya | Tick | 2005 | HM745259 | X | [Gallardo et al 2011](http://asf-referencelab.info/asf/images/files/publicaciones/Gallardo_et_al_2011b_Uganda.pdf) | ATGCAGCCTACCCACCACGCAGAGGTAAGCTTTCAGGATAGAGATACAGCTCTTCCAGATGCATGTTCATCCATATCAGATATTACCCCCATTACTTATCCGATCACGTTACCTATTATTAAAAACATTTCCGTCACTGCTCATGGTATCAATCTTATCGATAAATTTCCATCAAAGTTCTGCAGCTCTTACATACCCTTTCACTACGGAGGCAATTCGATTAAAACCCCCGACGATCCGGGCGCGATGATGATTACCTTTGCTTTGAAACCACGGGAGGAATACCAACCCAGTGGTCATATTAACGTATCCAGAGCAAGAGAATTTTATATTAGCTGGGACACAGATTATGTGGGGTCTATCACCACGGCCGATCTTGTGGTATCGGCATCCGCTATTAACTT | HM745294 | [Gallardo et al 2011](http://asf-referencelab.info/asf/images/files/publicaciones/Nix_et_al_2006.pdf) | AGTGCGTATACCTGTGCAAGCACCTGTGCAAGCACCTGTGCAGACACCAATGTAGACACTTGTGCAAGCACTTGTGTAAGCACCTGTGCAGACACTTGTGCAGACACCTGTGCAAGCACAGAATAC |
| 125 | Ken05/Tk8 | Kenya | Tick | 2005 | HM745260 | X | [Gallardo et al 2011](http://asf-referencelab.info/asf/images/files/publicaciones/Gallardo_et_al_2011b_Uganda.pdf) | ATGCAGCCTACCCACCACGCAGAGGTAAGCTTTCAGGATAGAGATACAGCTCTTCCAGATGCATGTTCATCCATATCTGATATTACCCCCATTACTTATCCGATCACATTACCTATTATTAAAAACATTTCCGTCACTGCTCATGGTATCAATCTTATCGATAAATTTCCATCAAAGTTCTGCAGCTCTTACATACCCTTTCACTACGGAGGCAATTCGATTAAAACCCCCGACGATCCGGGCGCGATGATGATTACCTTTGCTTTGAAACCACGGGAGGAATACCAACCCAGTGGTCATATTAACGTATCCAGAGCAAGAGAATTTTATATTAGCTGGGACACAGATTATGTGGGGTCTATCACCACGGCCGATCTTGTGGTATCGGCATCCGCTATTAACTT | HM745295 | [Gallardo et al 2011](http://asf-referencelab.info/asf/images/files/publicaciones/Nix_et_al_2006.pdf) | AGTGCGTATACCTGTGCAGACACCAATGTAGACACTTGTGCAAGCACTTGTGCAAGCACCAATGTAGACACTTGTGCAAGCACTTGTACAGACACTTGCGCAAGCACCTGTGCAAGCACAGAATAC |
| 126 | Ken05/Tk9 | Kenya | Tick | 2005 | HM745261 | X | [Gallardo et al 2011](http://asf-referencelab.info/asf/images/files/publicaciones/Gallardo_et_al_2011b_Uganda.pdf) | ATGCAGCCTACCCACCACGCAGAGGTAAGCTTTCAGGATAGAGATACAGCTCTTCCAGATGCATGTTCATCCATATCAGATATTACCCCCATTACTTATCCGATCACGTTACCTATTATTAAAAACATTTCCGTCACTGCTCATGGTATCAATCTTATCGATAAATTTCCATCAAAGTTCTGCAGCTCTTACATACCCTTTCACTACGGAGGCAATTCGATTAAAACCCCCGACGATCCGGGCGCGATGATGATTACCTTTGCTTTGAAACCACGGGAGGAATACCAACCCAGCGGTCATATTAACGTATCCAGAGCAAGAGAATTTTATATTAGCTGGGACACAGATTATGTGGGGTCTATCACCACGGCCGATCTTGTGGTATCGGCATCCGCTATTAACTT | HM745296 | [Gallardo et al 2011](http://asf-referencelab.info/asf/images/files/publicaciones/Nix_et_al_2006.pdf) | AGTGCGTATACCTGTGCAAGCACCTGTGCAAGCACCTGTGCAGACACCAATGTAGACACTTGTGCAAGCACTTGTGTAAGCACCTGTGCAGACACTTGTGCAGACACCTGTGCAAGCACAGAATAC |
| 127 | Ken05.DPk16 | Kenya | DP | 2005 | HM745264 | X | [Gallardo et al 2011](http://asf-referencelab.info/asf/images/files/publicaciones/Gallardo_et_al_2011b_Uganda.pdf) | ATGCAGCCTACCCACCACGCAGAGGTAAGCTTTCAGGATAGAGATACAGCTCTTCCAGATGCATGTTCATCCATATCAGATATTACCCCCATTACTTATCCGATCACGTTACCTATTATTAAAAACATTTCCGTCACTGCTCATGGTATCAATCTTATCGATAAATTTCCATCAAAGTTCTGCAGCTCTTACATACCCTTTCACTACGGAGGCAATTCGATTAAAACCCCCGACGATCCGGGCGCGATGATGATTACCTTTGCTTTGAAACCACGGGAGGAATACCAACCCAGTGGTCATATTAACGTATCCAGAGCAAGAGAATTTTATATTAGCTGGGACACAGATTATGTGGGGTCTATCACCACGGCCGATCTTGTGGTATCGGCATCCGCTATTAACTT | HM745299 | [Gallardo et al 2011](http://asf-referencelab.info/asf/images/files/publicaciones/Nix_et_al_2006.pdf) | AGTGCGTATACCTGTGCAAGCACCTGTGCAAGCACCTGTGCAGACACCAATGTAGACACTTGTGCAAGCACTTGTGTAAGCACCTGTGCAGACACTTGTGCAGACACCTGTGCAAGCACAGAATAC |
| 128 | Ken05.DPk18 | Kenya | DP | 2005 | HM745265 | X | [Gallardo et al 2011](http://asf-referencelab.info/asf/images/files/publicaciones/Gallardo_et_al_2011b_Uganda.pdf) | ATGCAGCCTACCCACCACGCAGAGGTAAGCTTTCAGGATAGAGATACAGCTCTTCCAGATGCATGTTCATCCATATCAGATATTACCCCCATTACTTATCCGATCACGTTACCTATTATTAAAAACATTTCCGTCACTGCTCATGGTATCAATCTTATCGATAAATTTCCATCAAAGTTCTGCAGCTCTTACATACCCTTTCACTACGGAGGCAATTCGATTAAAACCCCCGACGATCCGGGCGCGATGATGATTACCTTTGCTTTGAAACCACGGGAGGAATACCAACCCAGTGGTCATATTAACGTATCCAGAGCAAGAGAATTTTATATTAGCTGGGACACAGATTATGTGGGGTCTATCACCACGGCCGATCTTGTGGTATCGGCATCCGCTATTAACTT | HM745300 | [Gallardo et al 2011](http://asf-referencelab.info/asf/images/files/publicaciones/Nix_et_al_2006.pdf) | AGTGCGTATACCTGTGCAAGCACCTGTGCAAGCACCTGTGCAGACACCAATGTAGACACTTGTGCAAGCACTTGTGTAAGCACCTGTGCAGACACTTGTGCAGACACCTGTGCAAGCACAGAATAC |
| 129 | Ken05.DPk2 | Kenya | DP | 2005 | HM745263 | X | [Gallardo et al 2011](http://asf-referencelab.info/asf/images/files/publicaciones/Gallardo_et_al_2011b_Uganda.pdf) | ATGCAGCCTACCCACCACGCAGAGGTAAGCTTTCAGGATAGAGATACAGCTCTTCCAGATGCATGTTCATCCATATCAGATATTACCCCCATTACTTATCCGATCACGTTACCTATTATTAAAAACATTTCCGTCACTGCTCATGGTATCAATCTTATCGATAAATTTCCATCAAAGTTCTGCAGCTCTTACATACCCTTTCACTACGGAGGCAATTCGATTAAAACCCCCGACGATCCGGGCGCGATGATGATTACCTTTGCTTTGAAACCACGGGAGGAATACCAACCCAGTGGTCATATTAACGTATCCAGAGCAAGAGAATTTTATATTAGCTGGGACACAGATTATGTGGGGTCTATCACCACGGCCGATCTTGTGGTATCGGCATCCGCTATTAACTT | HM745298 | [Gallardo et al 2011](http://asf-referencelab.info/asf/images/files/publicaciones/Nix_et_al_2006.pdf) | AGTGCGTATACCTGTGCAAGCACCTGTGCAAGCACCTGTGCAGACACCAATGTAGACACTTGTGCAAGCACTTGTGTAAGCACCTGTGCAGACACTTGTGCAGACACCTGTGCAAGCACAGAATAC |
| 130 | Ken05.DPk21 | Kenya | DP | 2005 | HM745266 | X | [Gallardo et al 2011](http://asf-referencelab.info/asf/images/files/publicaciones/Gallardo_et_al_2011b_Uganda.pdf) | ATGCAGCCTACCCACCACGCAGAGGTAAGCTTTCAGGATAGAGATACAGCTCTTCCAGATGCATGTTCATCCATATCAGATATTACCCCCATTACTTATCCGATCACGTTACCTATTATTAAAAACATTTCCGTCACTGCTCATGGTATCAATCTTATCGATAAATTTCCATCAAAGTTCTGCAGCTCTTACATACCCTTTCACTACGGAGGCAATTCGATTAAAACCCCCGACGATCCGGGCGCGATGATGATTACCTTTGCTTTGAAACCACGGGAGGAATACCAACCCAGTGGTCATATTAACGTATCCAGAGCAAGAGAATTTTATATTAGCTGGGACACAGATTATGTGGGGTCTATCACCACGGCCGATCTTGTGGTATCGGCATCCGCTATTAACTT | HM745301 | [Gallardo et al 2011](http://asf-referencelab.info/asf/images/files/publicaciones/Nix_et_al_2006.pdf) | AGTGCGTATACCTGTGCAAGCACCTGTGCAAGCACCTGTGCAGACACCAATGTAGACACTTGTGCAAGCACTTGTGTAAGCACCTGTGCAGACACTTGTGCAGACACCTGTGCAAGCACAGAATAC |
| 131 | Ken05.DPk27 | Kenya | DP | 2005 | HM745267 | X | [Gallardo et al 2011](http://asf-referencelab.info/asf/images/files/publicaciones/Gallardo_et_al_2011b_Uganda.pdf) | ATGCAGCCTACCCACCACGCAGAGGTAAGCTTTCAGGATAGAGATACAGCTCTTCCAGATGCATGTTCATCCATATCAGATATTACCCCCATTACTTATCCGATCACGTTACCTATTATTAAAAACATTTCCGTCACTGCTCATGGTATCAATCTTATCGATAAATTTCCATCAAAGTTCTGCAGCTCTTACATACCCTTTCACTACGGAGGCAATTCGATTAAAACCCCCGACGATCCGGGCGCGATGATGATTACCTTTGCTTTGAAACCACGGGAGGAATACCAACCCAGTGGTCATATTAACGTATCCAGAGCAAGAGAATTTTATATTAGCTGGGACACAGATTATGTGGGGTCTATCACCACGGCCGATCTTGTGGTATCGGCATCCGCTATTAACTT | HM745302 | [Gallardo et al 2011](http://asf-referencelab.info/asf/images/files/publicaciones/Nix_et_al_2006.pdf) | AGTGCGTATACCTGTGCAAGCACCTGTGCAAGCACCTGTGCAGACACCAATGTAGACACTTGTGCAAGCACTTGTGTAAGCACCTGTGCAGACACTTGTGCAGACACCTGTGCAAGCACAGAATAC |
| 132 | Ken05.DPN15 | Kenya | DP | 2005 | HM745269 | X | [Gallardo et al 2011](http://asf-referencelab.info/asf/images/files/publicaciones/Gallardo_et_al_2011b_Uganda.pdf) | ATGCAGCCTACCCACCACGCAGAGGTAAGCTTTCAGGATAGAGATACAGCTCTTCCAGATGCATGTTCATCCATATCAGATATTACCCCCATTACTTATCCGATCACGTTACCTATTATTAAAAACATTTCCGTCACTGCTCATGGTATCAATCTTATCGATAAATTTCCATCAAAGTTCTGCAGCTCTTACATACCCTTTCACTACGGAGGCAATTCGATTAAAACCCCCGACGATCCGGGCGCGATGATGATTACCTTTGCTTTGAAACCACGGGAGGAATACCAACCCAGTGGTCATATTAACGTATCCAGAGCAAGAGAATTTTATATTAGCTGGGACACAGATTATGTGGGGTCTATCACCACGGCCGATCTTGTGGTATCGGCATCCGCTATTAACTT | HM745304 | [Gallardo et al 2011](http://asf-referencelab.info/asf/images/files/publicaciones/Nix_et_al_2006.pdf) | AGTGCGTATACCTGTGCAAGCACCTGTGCAAGCACCTGTGCAGACACCAATGTAGACACTTGTGCAAGCACTTGTGTAAGCACCTGTGCAGACACTTGTGCAGACACCTGTGCAAGCACAGAATAC |
| 133 | Ken05.DPN2 | Kenya | DP | 2005 | HM745268 | X | [Gallardo et al 2011](http://asf-referencelab.info/asf/images/files/publicaciones/Gallardo_et_al_2011b_Uganda.pdf) | ATGCAGCCTACCCACCACGCAGAGGTAAGCTTTCAGGATAGAGATACAGCTCTTCCAGATGCATGTTCATCCATATCAGATATTACCCCCATTACTTATCCGATCACGTTACCTATTATTAAAAACATTTCCGTCACTGCTCATGGTATCAATCTTATCGATAAATTTCCATCAAAGTTCTGCAGCTCTTACATACCCTTTCACTACGGAGGCAATTCGATTAAAACCCCCGACGATCCGGGCGCGATGATGATTACCTTTGCTTTGAAACCACGGGAGGAATACCAACCCAGTGGTCATATTAACGTATCCAGAGCAAGAGAATTTTATATTAGCTGGGACACAGATTATGTGGGGTCTATCACCACGGCCGATCTTGTGGTATCGGCATCCGCTATTAACTT | HM745303 | [Gallardo et al 2011](http://asf-referencelab.info/asf/images/files/publicaciones/Gallardo_et_al_2011b_Uganda.pdf) | AGTGCGTATACCTGTGCAAGCACCTGTGCAAGCACCTGTGCAGACACCAATGTAGACACTTGTGCAAGCACTTGTGTAAGCACCTGTGCAGACACTTGTGCAGACACCTGTGCAAGCACAGAATAC |
| 134 | Ken05.DPN23 | Kenya | DP | 2005 | HM745270 | X | [Gallardo et al 2011](http://asf-referencelab.info/asf/images/files/publicaciones/Gallardo_et_al_2011b_Uganda.pdf) | ATGCAGCCTACCCACCACGCAGAGGTAAGCTTTCAGGATAGAGATACAGCTCTTCCAGATGCATGTTCATCCATATCAGATATTACCCCCATTACTTATCCGATCACGTTACCTATTATTAAAAACATTTCCGTCACTGCTCATGGTATCAATCTTATCGATAAATTTCCATCAAAGTTCTGCAGCTCTTACATACCCTTTCACTACGGAGGCAATTCGATTAAAACCCCCGACGATCCGGGCGCGATGATGATTACCTTTGCTTTGAAACCACGGGAGGAATACCAACCCAGTGGTCATATTAACGTATCCAGAGCAAGAGAATTTTATATTAGCTGGGACACAGATTATGTGGGGTCTATCACCACGGCCGATCTTGTGGTATCGGCATCCGCTATTAACTT | HM745305 | [Gallardo et al 2011](http://asf-referencelab.info/asf/images/files/publicaciones/Gallardo_et_al_2011b_Uganda.pdf) | AGTGCGTATACCTGTGCAAGCACCTGTGCAAGCACCTGTGCAGACACCAATGTAGACACTTGTGCAAGCACTTGTGTAAGCACCTGTGCAGACACTTGTGCAGACACCTGTGCAAGCACAGAATAC |
| 135 | Ken05.DPU1 | Kenya | DP | 2005 | HM745271 | X | [Gallardo et al 2011](http://asf-referencelab.info/asf/images/files/publicaciones/Gallardo_et_al_2011b_Uganda.pdf) | ATGCAGCCTACCCACCACGCAGAGGTAAGCTTTCAGGATAGAGATACAGCTCTTCCAGATGCATGTTCATCCATATCAGATATTACCCCCATTACTTATCCGATCACGTTACCTATTATTAAAAACATTTCCGTCACTGCTCATGGTATCAATCTTATCGATAAATTTCCATCAAAGTTCTGCAGCTCTTACATACCCTTTCACTACGGAGGCAATTCGATTAAAACCCCCGACGATCCGGGCGCGATGATGATTACCTTTGCTTTGAAACCACGGGAGGAATACCAACCCAGTGGTCATATTAACGTATCCAGAGCAAGAGAATTTTATATTAGCTGGGACACAGATTATGTGGGGTCTATCACCACGGCCGATCTTGTGGTATCGGCATCCGCTATTAACTT | HM745306 | [Gallardo et al 2011](http://asf-referencelab.info/asf/images/files/publicaciones/Gallardo_et_al_2011b_Uganda.pdf) | AGTGCGTATACCTGTGCAAGCACCTGTGCAAGCACCTGTGCAGACACCAATGTAGACACTTGTGCAAGCACTTGTGTAAGCACCTGTGCAGACACTTGTGCAGACACCTGTGCAAGCACAGAATAC |
| 136 | Ken05.DPU11 | Kenya | DP | 2005 | HM745273 | X | [Gallardo et al 2011](http://asf-referencelab.info/asf/images/files/publicaciones/Gallardo_et_al_2011b_Uganda.pdf) | ATGCAGCCTACCCACCACGCAGAGGTAAGCTTTCAGGATAGAGATACAGCTCTTCCAGATGCATGTTCATCCATATCAGATATTACCCCCATTACTTATCCGATCACGTTACCTATTATTAAAAACATTTCCGTCACTGCTCATGGTATCAATCTTATCGATAAATTTCCATCAAAGTTCTGCAGCTCTTACATACCCTTTCACTACGGAGGCAATTCGATTAAAACCCCCGACGATCCGGGCGCGATGATGATTACCTTTGCTTTGAAACCACGGGAGGAATACCAACCCAGTGGTCATATTAACGTATCCAGAGCAAGAGAATTTTATATTAGCTGGGACACAGATTATGTGGGGTCTATCACCACGGCCGATCTTGTGGTATCGGCATCCGCTATTAACTT | HM745308 | [Gallardo et al 2011](http://asf-referencelab.info/asf/images/files/publicaciones/Gallardo_et_al_2011b_Uganda.pdf) | AGTGCGTATACCTGTGCAAGCACCTGTGCAAGCACCTGTGCAGACACCAATGTAGACACTTGTGCAAGCACTTGTGTAAGCACCTGTGCAGACACTTGTGCAGACACCTGTGCAAGCACAGAATAC |
| 137 | Ken05.DPU2 | Kenya | DP | 2005 | HM745272 | X | [Gallardo et al 2011](http://asf-referencelab.info/asf/images/files/publicaciones/Gallardo_et_al_2011b_Uganda.pdf) | ATGCAGCCTACCCACCACGCAGAGGTAAGCTTTCAGGATAGAGATACAGCTCTTCCAGATGCATGTTCATCCATATCAGATATTACCCCCATTACTTATCCGATCACGTTACCTATTATTAAAAACATTTCCGTCACTGCTCATGGTATCAATCTTATCGATAAATTTCCATCAAAGTTCTGCAGCTCTTACATACCCTTTCACTACGGAGGCAATTCGATTAAAACCCCCGACGATCCGGGCGCGATGATGATTACCTTTGCTTTGAAACCACGGGAGGAATACCAACCCAGTGGTCATATTAACGTATCCAGAGCAAGAGAATTTTATATTAGCTGGGACACAGATTATGTGGGGTCTATCACCACGGCCGATCTTGTGGTATCGGCATCCGCTATTAACTT | HM745307 | [Gallardo et al 2011](http://asf-referencelab.info/asf/images/files/publicaciones/Gallardo_et_al_2011b_Uganda.pdf) | AGTGCGTATACCTGTGCAAGCACCTGTGCAAGCACCTGTGCAGACACCAATGTAGACACTTGTGCAAGCACTTGTGTAAGCACCTGTGCAGACACTTGTGCAGACACCTGTGCAAGCACAGAATAC |
| 138 | Ken05.DPU22 | Kenya | DP | 2005 | HM745274 | X | [Gallardo et al 2011](http://asf-referencelab.info/asf/images/files/publicaciones/Gallardo_et_al_2011b_Uganda.pdf) | ATGCAGCCTACCCACCACGCAGAGGTAAGCTTTCAGGATAGAGATACAGCTCTTCCAGATGCATGTTCATCCATATCAGATATTACCCCCATTACTTATCCGATCACGTTACCTATTATTAAAAACATTTCCGTCACTGCTCATGGTATCAATCTTATCGATAAATTTCCATCAAAGTTCTGCAGCTCTTACATACCCTTTCACTACGGAGGCAATTCGATTAAAACCCCCGACGATCCGGGCGCGATGATGATTACCTTTGCTTTGAAACCACGGGAGGAATACCAACCCAGTGGTCATATTAACGTATCCAGAGCAAGAGAATTTTATATTAGCTGGGACACAGATTATGTGGGGTCTATCACCACGGCCGATCTTGTGGTATCGGCATCCGCTATTAACTT | HM745309 | [Gallardo et al 2011](http://asf-referencelab.info/asf/images/files/publicaciones/Gallardo_et_al_2011b_Uganda.pdf) | AGTGCGTATACCTGTGCAAGCACCTGTGCAAGCACCTGTGCAGACACCAATGTAGACACTTGTGCAAGCACTTGTGTAAGCACCTGTGCAGACACTTGTGCAGACACCTGTGCAAGCACAGAATAC |
| 139 | Ken06.B1 | Kenya | DP | 2006 | FJ154434 | IX | [Gallardo et al., 2009](http://asf-referencelab.info/asf/images/files/publicaciones/Gallardo_et_al_2011b_Uganda.pdf) | ATGCAGCCTACCCACCACGCAGAGGTAAGCTTTCAGGATAGAGATACAGCTCTTCCAGATGCATGTTCATCCATATCTGATATTACCCCCATTACTTATCCGATCACATTACCTATTATTAAAAACATTTCCGTTACTGCTCACGGTATCAATCTTATCGATAAATTTCCATCAAAGTTCTGCAGCTCTTACATACCCTTCCACTACGGAGGCAATTCGATTAAAACCCCCGACGATCCGGGCGCGATGATGATTACCTTTGCTTTGAAACCACGGGAGGAATACCAACCCAGCGGTCATATTAACGTATCCAGAGCAAGAGAATTTTATATTAGCTGGGACACAGATTATGTGGGGTCTATCACCACGGCTGATCTTGTGGTATCGGCATCCGCTATTAACTT | FJ174329 | [Gallardo et al., 2009](http://asf-referencelab.info/asf/images/files/publicaciones/Gallardo_et_al_2011b_Uganda.pdf) | AGTGCGTATACCTGTGCAAGCACTTGTGCAAGCACTTGTGCAAGCACTTGTGCAGACACCAATGTAGACACTTGTGCAAGCACTTGTGCAGACATTTGTGCAGACACCAATGTAGACACTTGTGCAAGCACTTGTGCAGATACTTGTGCAGACACTTGTGTAAGCACTTGTGTAAGCACTTGTGCAGATACTTGTGCAGACACCAATGTAGACACTTGTGCAAGCACTTGTGCAGACACCAATGTAGACACTTGTGTAAGCACTTGTGCAGACACCTGTGCAAGCACAGAATAC |
| 140 | Ken06.B2 | Kenya | DP | 2006 | FJ154435 | IX | [Gallardo et al., 2009](http://asf-referencelab.info/asf/images/files/publicaciones/Gallardo_et_al_2011b_Uganda.pdf) | ATGCAGCCTACCCACCACGCAGAGGTAAGCTTTCAGGATAGAGATACAGCTCTTCCAGATGCATGTTCATCCATATCTGATATTACCCCCATTACTTATCCGATCACATTACCTATTATTAAAAACATTTCCGTTACTGCTCACGGTATCAATCTTATCGATAAATTTCCATCAAAGTTCTGCAGCTCTTACATACCCTTCCACTACGGAGGCAATTCGATTAAAACCCCCGACGATCCGGGCGCGATGATGATTACCTTTGCTTTGAAACCACGGGAGGAATACCAACCCAGCGGTCATATTAACGTATCCAGAGCAAGAGAATTTTATATTAGCTGGGACACAGATTATGTGGGGTCTATCACCACGGCTGATCTTGTGGTATCGGCATCCGCTATTAACTT | FJ174330 | [Gallardo et al., 2009](http://asf-referencelab.info/asf/images/files/publicaciones/Gallardo_et_al_2011b_Uganda.pdf) | AGTGCGTATACCTGTGCAAGCACTTGTGCAAGCACTTGTGCAAGCACTTGTGCAGACACCAATGTAGACACTTGTGCAAGCACTTGTGCAGACATTTGTGCAGACACCAATGTAGACACTTGTGCAAGCACTTGTGCAGATACTTGTGCAGACACTTGTGTAAGCACTTGTGTAAGCACTTGTGCAGATACTTGTGCAGACACCAATGTAGACACTTGTGCAAGCACTTGTGCAGACACCAATGTAGACACTTGTGTAAGCACTTGTGCAGACACCTGTGCAAGCACAGAATAC |
| 141 | Ken06.B3 | Kenya | DP | 2006 | FJ154436 | IX | [Gallardo et al., 2009](http://asf-referencelab.info/asf/images/files/publicaciones/Gallardo_et_al_2011b_Uganda.pdf) | ATGCAGCCTACCCACCACGCAGAGGTAAGCTTTCAGGATAGAGATACAGCTCTTCCAGATGCATGTTCATCCATATCTGATATTACCCCCATTACTTATCCGATCACATTACCTATTATTAAAAACATTTCCGTTACTGCTCACGGTATCAATCTTATCGATAAATTTCCATCAAAGTTCTGCAGCTCTTACATACCCTTCCACTACGGAGGCAATTCGATTAAAACCCCCGACGATCCGGGCGCGATGATGATTACCTTTGCTTTGAAACCACGGGAGGAATACCAACCCAGCGGTCATATTAACGTATCCAGAGCAAGAGAATTTTATATTAGCTGGGACACAGATTATGTGGGGTCTATCACCACGGCTGATCTTGTGGTATCGGCATCCGCTATTAACTT | FJ174331 | [Gallardo et al., 2009](http://asf-referencelab.info/asf/images/files/publicaciones/Gallardo_et_al_2011b_Uganda.pdf) | AGTGCGTATACCTGTGCAAGCACTTGTGCAAGCACTTGTGCAAGCACTTGTGCAGACACCAATGTAGACACTTGTGCAAGCACTTGTGCAGACATTTGTGCAGACACCAATGTAGACACTTGTGCAAGCACTTGTGCAGATACTTGTGCAGACACTTGTGTAAGCACTTGTGTAAGCACTTGTGCAGATACTTGTGCAGACACCAATGTAGACACTTGTGCAAGCACTTGTGCAGACACCAATGTAGACACTTGTGTAAGCACTTGTGCAGACACCTGTGCAAGCACAGAATAC |
| 142 | Ken06.B4 | Kenya | DP | 2006 | FJ154437 | IX | [Gallardo et al., 2009](http://asf-referencelab.info/asf/images/files/publicaciones/Gallardo_et_al_2011b_Uganda.pdf) | ATGCAGCCTACCCACCACGCAGAGGTAAGCTTTCAGGATAGAGATACAGCTCTTCCAGATGCATGTTCATCCATATCTGATATTACCCCCATTACTTATCCGATCACATTACCTATTATTAAAAACATTTCCGTTACTGCTCACGGTATCAATCTTATCGATAAATTTCCATCAAAGTTCTGCAGCTCTTACATACCCTTCCACTACGGAGGCAATTCGATTAAAACCCCCGACGATCCGGGCGCGATGATGATTACCTTTGCTTTGAAACCACGGGAGGAATACCAACCCAGCGGTCATATTAACGTATCCAGAGCAAGAGAATTTTATATTAGCTGGGACACAGATTATGTGGGGTCTATCACCACGGCTGATCTTGTGGTATCGGCATCCGCTATTAACTT | FJ174332 | [Gallardo et al., 2009](http://asf-referencelab.info/asf/images/files/publicaciones/Gallardo_et_al_2011b_Uganda.pdf) | AGTGCGTATACCTGTGCAAGCACTTGTGCAAGCACTTGTGCAAGCACTTGTGCAGACACCAATGTAGACACTTGTGCAAGCACTTGTGCAGACATTTGTGCAGACACCAATGTAGACACTTGTGCAAGCACTTGTGCAGATACTTGTGCAGACACTTGTGTAAGCACTTGTGTAAGCACTTGTGCAGATACTTGTGCAGACACCAATGTAGACACTTGTGCAAGCACTTGTGCAGACACCAATGTAGACACTTGTGTAAGCACTTGTGCAGACACCTGTGCAAGCACAGAATAC |
| 143 | Ken06.B5 | Kenya | DP | 2006 | FJ154438 | IX | [Gallardo et al., 2009](http://asf-referencelab.info/asf/images/files/publicaciones/Gallardo-et-al-2009a.pdf) | ATGCAGCCTACCCACCACGCAGAGGTAAGCTTTCAGGATAGAGATACAGCTCTTCCAGATGCATGTTCATCCATATCTGATATTACCCCCATTACTTATCCGATCACATTACCTATTATTAAAAACATTTCCGTTACTGCTCACGGTATCAATCTTATCGATAAATTTCCATCAAAGTTCTGCAGCTCTTACATACCCTTCCACTACGGAGGCAATTCGATTAAAACCCCCGACGATCCGGGCGCGATGATGATTACCTTTGCTTTGAAACCACGGGAGGAATACCAACCCAGCGGTCATATTAACGTATCCAGAGCAAGAGAATTTTATATTAGCTGGGACACAGATTATGTGGGGTCTATCACCACGGCTGATCTTGTGGTATCGGCATCCGCTATTAACTT | FJ174333 | [Gallardo et al., 2009](http://asf-referencelab.info/asf/images/files/publicaciones/Nix_et_al_2006.pdf) | AGTGCGTATACCTGTGCAAGCACTTGTGCAAGCACTTGTGCAAGCACTTGTGCAGACACCAATGTAGACACTTGTGCAAGCACTTGTGCAGACATTTGTGCAGACACCAATGTAGACACTTGTGCAAGCACTTGTGCAGATACTTGTGCAGACACTTGTGTAAGCACTTGTGTAAGCACTTGTGCAGATACTTGTGCAGACACCAATGTAGACACTTGTGCAAGCACTTGTGCAGACACCAATGTAGACACTTGTGTAAGCACTTGTGCAGACACCTGTGCAAGCACAGAATAC |
| 144 | Nig06/PLJs16 | Nigeria | DP | 2006 |  | I | [This study (gallardo@inia.es)](http://asf-referencelab.info/asf/images/files/publicaciones/Gallardo-et-al-2009a.pdf) | ATGCAGCCTACTCACCACGCAGAGATAAGCTTTCAGGATAGAGATACAGCTCTTCCAGACGCATGTTCATCTATATCGGATATTAGCCCCGTTACGTATCCGATCACATTACCTATTATTAAAAACATTTCCGTAACTGCTCATGGTATCAATCTTATCGATAAGTTTCCATCAAAGTTCTGCAGCTCTTACATACCCTTCCACTACGGAGGCAATGCAATTAAAACCCCCGATGATCCGGGTGCGATGATGATTACCTTTGCTTTGAAGCCACGGGAGGAATACCAACCCAGTGGTCATATTAACGTATCCAGAGCAAGAGAATTTTATATTAGTTGGGACACGGATTACGTGGGGTCTATCACTACGGCTGATCTTGTGGTATCGGCATCTGCTATTAACTT |  | [This study (gallardo@inia.es)](http://asf-referencelab.info/asf/images/files/publicaciones/Nix_et_al_2006.pdf) | AGTGCGTATACTTGTGCAAGCACTTGTGCAGATACCAATGTAGACACCTGTGCAAGCACTTGTGCAAGCACTTGTGCAAGCACTTGTGCAAGCACAGGTGCAAGCACTTGTGCAAGCACAGGTGCAAGCACTTGTGCAGATACCAATGTAGACACCTGTGCAAGCACTTGTGCAAGCACTTGTGCAAGCACTTGTGCAAGCACAGGTGCAAGCACTTGTGCAGATACCAATGTAGACACCTGTGCAAGCACCTGTGCAAACACCTGTGCAAGCACAGAATAC |
| 145 | Nig06/PLJs42 | Nigeria | DP | 2006 |  | I | [This study (gallardo@inia.es)](http://asf-referencelab.info/asf/images/files/publicaciones/Gallardo-et-al-2009a.pdf) | ATGCAGCCTACTCACCACGCAGAGATAAGCTTTCAGGATAGAGATACAGCTCTTCCAGACGCATGTTCATCTATATCGGATATTAGCCCCGTTACGTATCCGATCACATTACCTATTATTAAAAACATTTCCGTAACTGCTCATGGTATCAATCTTATCGATAAGTTTCCATCAAAGTTCTGCAGCTCTTACATACCCTTCCACTACGGAGGCAATGCAATTAAAACCCCCGATGATCCGGGTGCGATGATGATTACCTTTGCTTTGAAGCCACGGGAGGAATACCAACCCAGTGGTCATATTAACGTATCCAGAGCAAGAGAATTTTATATTAGTTGGGACACGGATTACGTGGGGTCTATCACTACGGCTGATCTTGTGGTATCGGCATCTGCTATTAACTT |  | [This study (gallardo@inia.es)](http://asf-referencelab.info/asf/images/files/publicaciones/Nix_et_al_2006.pdf) | AGTGCGTATACTTGTGCAAGCACTTGTGCAGATACCAATGTAGACACCTGTGCAAGCACTTGTGCAGATACCAATGTAGACACCTGTGCAAGCACTTGTGCAAGCACTTGTGCAAGCACTTGTGCAAGCACTTGTGCAAGCACAGGTGCAAGCACTTGTGCAGATACCAATGTAGACACCTGTGCAAGCACCTGTGCAAACACCTGTGCAAGCACAGAATAC |
| 146 | Nig06/PLJs43 | Nigeria | DP | 2006 |  | I | [This study (gallardo@inia.es)](http://asf-referencelab.info/asf/images/files/publicaciones/Gallardo-et-al-2009a.pdf) | ATGCAGCCTACTCACCACGCAGAGATAAGCTTTCAGGATAGAGATACAGCTCTTCCAGACGCATGTTCATCTATATCGGATATTAGCCCCGTTACGTATCCGATCACATTACCTATTATTAAAAACATTTCCGTAACTGCTCATGGTATCAATCTTATCGATAAGTTTCCATCAAAGTTCTGCAGCTCTTACATACCCTTCCACTACGGAGGCAATGCAATTAAAACCCCCGATGATCCGGGTGCGATGATGATTACCTTTGCTTTGAAGCCACGGGAGGAATACCAACCCAGTGGTCATATTAACGTATCCAGAGCAAGAGAATTTTATATTAGTTGGGACACGGATTACGTGGGGTCTATCACTACGGCTGATCTTGTGGTATCGGCATCTGCTATTAACTT |  | [This study (gallardo@inia.es)](http://asf-referencelab.info/asf/images/files/publicaciones/Nix_et_al_2006.pdf) | AGTGCGTATACTTGTGCAAGCACTTGTGCAGATACCAATGTAGACACCTGTGCAAGCACTTGTGCAAGCACTTGTGCAAGCACTTGTGCAAGCACAGGTGCAAGCACTTGTGCAAGCACAGGTGCAAGCACTTGTGCAGATACCAATGTAGACACCTGTGCAAGCACTTGTGCAAGCACTTGTGCAAGCACTTGTGCAAGCACAGGTGCAAGCACTTGTGCAGATACCAATGTAGACACCTGTGCAAGCACCTGTGCAAACACCTGTGCAAGCACAGAATAC |
| 147 | Ken06.Bus | Kenya | DP | 2006 | FJ154439 | IX | [Gallardo et al., 2009](http://asf-referencelab.info/asf/images/files/publicaciones/Gallardo-et-al-2009a.pdf) | ATGCAGCCTACCCACCACGCAGAGGTAAGCTTTCAGGATAGAGATACAGCTCTTCCAGATGCATGTTCATCCATATCTGATATTACCCCCATTACTTATCCGATCACATTACCTATTATTAAAAACATTTCCGTTACTGCTCACGGTATCAATCTTATCGATAAATTTCCATCAAAGTTCTGCAGCTCTTACATACCCTTCCACTACGGAGGCAATTCGATTAAAACCCCCGACGATCCGGGCGCGATGATGATTACCTTTGCTTTGAAACCACGGGAGGAATACCAACCCAGCGGTCATATTAACGTATCCAGAGCAAGAGAATTTTATATTAGCTGGGACACAGATTATGTGGGGTCTATCACCACGGCTGATCTTGTGGTATCGGCATCCGCTATTAACTT | FJ174334 | [Gallardo et al., 2009](http://asf-referencelab.info/asf/images/files/publicaciones/Nix_et_al_2006.pdf) | AGTGCGTATACCTGTGCAAGCACTTGTGCAAGCACTTGTGCAAGCACTTGTGCAGACACCAATGTAGACACTTGTGCAAGCACTTGTGCAGACATTTGTGCAGACACCAATGTAGACACTTGTGCAAGCACTTGTGCAGATACTTGTGCAGACACTTGTGTAAGCACTTGTGTAAGCACTTGTGCAGACACCAATGTAGACACTTGTGCAAGCACTTGTGCAGACACCAATGTAGACACTTGTGTAAGCACTTGTGCAGACACCTGTGCAAGCACAGAATAC |
| 148 | GH06/NE26 | Ghana | DP | 2006 |  | I | [This study (gallardo@inia.es)](http://asf-referencelab.info/asf/images/files/publicaciones/Gallardo-et-al-2009a.pdf) | ATGCAGCCTACTCACCACGCAGAGATAAGCTTTCAGGATAGAGATACAGCTCTTCCAGACGCATGTTCATCTATATCGGATATTAGCCCCGTTACGTATCCGATCACATTACCTATTATTAAAAACATTTCCGTAACTGCTCATGGTATCAATCTTATCGATAAGTTTCCATCAAAGTTCTGCAGCTCTTACATACCCTTCCACTACGGAGGCAATGCAATTAAAACCCCCGATGATCCGGGTGCGATGATGATTACCTTTGCTTTGAAGCCACGGGAGGAATACCAACCCAGTGGTCATATTAACGTATCCAGAGCAAGAGAATTTTATATTAGTTGGGACACGGATTACGTGGGGTCTATCACTACGGCTGATCTTGTGGTATCGGCATCTGCTATTAACTT |  | [This study (gallardo@inia.es)](http://asf-referencelab.info/asf/images/files/publicaciones/Nix_et_al_2006.pdf) | AGTGCGTATACTTGTGCAAGCACTTGTGCAGATACCAATGTAGACACCTGTGCAAGCACTTGTGCAAGCACTTGTGCAAGCACTTGTGCAAGCACTTGTGCAAGCACTTGTGCAAGCACAGGTGCAAGCACTTGTGCAGATACCAATGTAGACACCTGTGCAAGCACTTGTGCAAGCACTTGTGCAAGCACTTGTGCAAGCACTTGTGCAAGCACTTGTGCAAGCACAGGTGCAAGCACTTGTGCAGATACCAATGTAGACACCTGTGCAAGCACTTGTGCAAGCACTTGTGCAAGCACTTGTGCAAGCACTTGTGCAAGCACTTGTGCAAGCACAGGTGCAAGCACTTGTGCAGATACCAATGTAGACACCTGTGCAAGCACCTGTGCAAACACCTGTGCAAGCACAGAATAC |
| 149 | Ken06.Kis | Kenya | DP | 2006 | FJ154440 | IX | [Gallardo et al., 2009](http://asf-referencelab.info/asf/images/files/publicaciones/Gallardo-et-al-2009a.pdf) | ATGCAGCCTACCCACCACGCAGAGGTAAGCTTTCAGGATAGAGATACAGCTCTTCCAGATGCATGTTCATCCATATCTGATATTACCCCCATTACTTATCCGATCACATTACCTATTATTAAAAACATTTCCGTTACTGCTCACGGTATCAATCTTATCGATAAATTTCCATCAAAGTTCTGCAGCTCTTACATACCCTTCCACTACGGAGGCAATTCGATTAAAACCCCCGACGATCCGGGCGCGATGATGATTACCTTTGCTTTGAAACCACGGGAGGAATACCAACCCAGCGGTCATATTAACGTATCCAGAGCAAGAGAATTTTATATTAGCTGGGACACAGATTATGTGGGGTCTATCACCACGGCTGATCTTGTGGTATCGGCATCCGCTATTAACTT | FJ174337 | [Gallardo et al., 2009](http://asf-referencelab.info/asf/images/files/publicaciones/Nix_et_al_2006.pdf) | AGTGCGTATACCTGTGCAAGCACTTGTGCAAGCACTTGTGCAAGCACTTGTGCAGACACCAATGTAGACACTTGTGCAAGCACTTGTGCAGACATTTGTGCAGACACCAATGTAGACACTTGTGCAAGCACTTGTGCAGATACTTGTGCAGACACTTGTGTAAGCACTTGTGTAAGCACTTGTGCAGACACCAATGTAGACACTTGTGCAAGCACTTGTGCAGACACCAATGTAGACACTTGTGTAAGCACTTGTGCAGACACCTGTGCAAGCACAGAATAC |
| 150 | Ken07.Kia | Kenya | DP | 2007 | FJ154443 | IX | [Gallardo et al., 2009](http://asf-referencelab.info/asf/images/files/publicaciones/Gallardo-et-al-2009a.pdf) | ATGCAGCCTACCCACCACGCAGAGGTAAGCTTTCAGGATAGAGATACAGCTCTTCCAGATGCATGTTCATCCATATCTGATATTACCCCCATTACTTATCCGATCACATTACCTATTATTAAAAACATTTCCGTTACTGCTCACGGTATCAATCTTATCGATAAATTTCCATCAAAGTTCTGCAGCTCTTACATACCCTTCCACTACGGAGGCAATTCGATTAAAACCCCCGACGATCCGGGCGCGATGATGATTACCTTTGCTTTGAAACCACGGGAGGAATACCAACCCAGCGGTCATATTAACGTATCCAGAGCAAGAGAATTTTATATTAGCTGGGACACAGATTATGTGGGGTCTATCACCACGGCTGATCTTGTGGTATCGGCATCCGCTATTAACTT | FJ238539 | [Gallardo et al., 2009](http://asf-referencelab.info/asf/images/files/publicaciones/Nix_et_al_2006.pdf) | AGTGCGTATACCTGTGCAAGCACTTGTGCAAGCACTTGTGCAAGCACTTGTGCAGACACCAATGTAGACACTTGTGCAAGCACTTGTGCAGACATTTGTGCAGACACCAATGTAGACACTTGTGCAAGCACTTGTGCAGATACTTGTGCAGACACTTGTGTAAGCACTTGTGTAAGCACTTGTGCAGACACCAATGTAGACACTTGTGCAAGCACTTGTGCAGACACCAATGTAGACACTTGTGTAAGCACTTGTGCAGACACCTGTGCAAGCACAGAATAC |
| 151 | Ken07.Eld1 | Kenya | DP | 2007 | FJ154441 | IX | [Gallardo et al., 2009](http://asf-referencelab.info/asf/images/files/publicaciones/Gallardo-et-al-2009a.pdf) | ATGCAGCCTACCCACCACGCAGAGGTAAGCTTTCAGGATAGAGATACAGCTCTTCCAGATGCATGTTCATCCATATCTGATATTACCCCCATTACTTATCCGATCACATTACCTATTATTAAAAACATTTCCGTTACTGCTCACGGTATCAATCTTATCGATAAATTTCCATCAAAGTTCTGCAGCTCTTACATACCCTTCCACTACGGAGGCAATTCGATTAAAACCCCCGACGATCCGGGCGCGATGATGATTACCTTTGCTTTGAAACCACGGGAGGAATACCAACCCAGCGGTCATATTAACGTATCCAGAGCAAGAGAATTTTATATTAGCTGGGACACAGATTATGTGGGGTCTATCACCACGGCTGATCTTGTGGTATCGGCATCCGCTATTAACTT | FJ174335 | [Gallardo et al., 2009](http://asf-referencelab.info/asf/images/files/publicaciones/Nix_et_al_2006.pdf) | AGTGCGTATACCTGTGCAAGCACTTGTGCAAGCACTTGTGCAAGCACTTGTGCAGACACCAATGTAGACACTTGTGCAAGCACTTGTGCAGACATTTGTGCAGACACCAATGTAGACACTTGTGCAAGCACTTGTGCAGATACTTGTGCAGACACTTGTGTAAGCACTTGTGTAAGCACTTGTGCAGACACCAATGTAGACACTTGTGCAAGCACTTGTGCAGACACCAATGTAGACACTTGTGTAAGCACTTGTGCAGACACCTGTGCAAGCACAGAATAC |
| 152 | Ken07.Eld2 | Kenya | DP | 2007 | FJ154442 | IX | [Gallardo et al., 2009](http://asf-referencelab.info/asf/images/files/publicaciones/Gallardo-et-al-2009a.pdf) | ATGCAGCCTACCCACCACGCAGAGGTAAGCTTTCAGGATAGAGATACAGCTCTTCCAGATGCATGTTCATCCATATCTGATATTACCCCCATTACTTATCCGATCACATTACCTATTATTAAAAACATTTCCGTTACTGCTCACGGTATCAATCTTATCGATAAATTTCCATCAAAGTTCTGCAGCTCTTACATACCCTTCCACTACGGAGGCAATTCGATTAAAACCCCCGACGATCCGGGCGCGATGATGATTACCTTTGCTTTGAAACCACGGGAGGAATACCAACCCAGCGGTCATATTAACGTATCCAGAGCAAGAGAATTTTATATTAGCTGGGACACAGATTATGTGGGGTCTATCACCACGGCTGATCTTGTGGTATCGGCATCCGCTATTAACTT | FJ174336 | [Gallardo et al., 2009](http://asf-referencelab.info/asf/images/files/publicaciones/Nix_et_al_2006.pdf) | AGTGCGTATACCTGTGCAAGCACTTGTGCAAGCACTTGTGCAAGCACTTGTGCAGACACCAATGTAGACACTTGTGCAAGCACTTGTGCAGACATTTGTGCAGACACCAATGTAGACACTTGTGCAAGCACTTGTGCAGATACTTGTGCAGACACTTGTGTAAGCACTTGTGTAAGCACTTGTGCAGACACCAATGTAGACACTTGTGCAAGCACTTGTGCAGACACCAATGTAGACACTTGTGTAAGCACTTGTGCAGACACCTGTGCAAGCACAGAATAC |
| 153 | Ken07.Nak | Kenya | DP | 2007 | FJ154444 | IX | [Gallardo et al., 2009](http://asf-referencelab.info/asf/images/files/publicaciones/Gallardo-et-al-2009a.pdf) | ATGCAGCCTACCCACCACGCAGAGGTAAGCTTTCAGGATAGAGATACAGCTCTTCCAGATGCATGTTCATCCATATCTGATATTACCCCCATTACTTATCCGATCACATTACCTATTATTAAAAACATTTCCGTTACTGCTCACGGTATCAATCTTATCGATAAATTTCCATCAAAGTTCTGCAGCTCTTACATACCCTTCCACTACGGAGGCAATTCGATTAAAACCCCCGACGATCCGGGCGCGATGATGATTACCTTTGCTTTGAAACCACGGGAGGAATACCAACCCAGCGGTCATATTAACGTATCCAGAGCAAGAGAATTTTATATTAGCTGGGACACAGATTATGTGGGGTCTATCACCACGGCTGATCTTGTGGTATCGGCATCCGCTATTAACTT | FJ174338 | [Gallardo et al., 2009](http://asf-referencelab.info/asf/images/files/publicaciones/Nix_et_al_2006.pdf) | AGTGCGTATACCTGTGCAAGCACTTGTGCAAGCACTTGTGCAAGCACTTGTGCAGACACCAATGTAGACACTTGTGCAAGCACTTGTGCAGACATTTGTGCAGACACCAATGTAGACACTTGTGCAAGCACTTGTGCAGATACTTGTGCAGACACTTGTGTAAGCACTTGTGTAAGCACTTGTGCAGACACCAATGTAGACACTTGTGCAAGCACTTGTGCAGACACCAATGTAGACACTTGTGTAAGCACTTGTGCAGACACCTGTGCAAGCACAGAATAC |
| 154 | BF07/IpB1 | Burkina Faso | DP | 2007 |  | I | [This study (gallardo@inia.es)](http://asf-referencelab.info/asf/images/files/publicaciones/Gallardo-et-al-2009a.pdf) | ATGCAGCCTACTCACCACGCAGAGATAAGCTTTCAGGATAGAGATACAGCTCTTCCAGACGCATGTTCATCTATATCGGATATTAGCCCCGTTACGTATCCGATCACATTACCTATTATTAAAAACATTTCCGTAACTGCTCATGGTATCAATCTTATCGATAAGTTTCCATCAAAGTTCTGCAGCTCTTACATACCCTTCCACTACGGAGGCAATGCAATTAAAACCCCCGATGATCCGGGTGCGATGATGATTACCTTTGCTTTGAAGCCACGGGAGGAATACCAACCCAGTGGTCATATTAACGTATCCAGAGCAAGAGAATTTTATATTAGTTGGGACACGGATTACGTGGGGTCTATCACTACGGCTGATCTTGTGGTATCGGCATCTGCTATTAACTT |  | [This study (gallardo@inia.es)](http://asf-referencelab.info/asf/images/files/publicaciones/Nix_et_al_2006.pdf) | AGTGCGTATACTTGTGCAAGCACTTGTGCAGATACCAATGTAGACACCTGTGCAAGCACTTGTGCAAGCACTTGTGCAAGCACTTGTGCAAGCACAGGTGCAAGCACTTGTGCAGATACCAATGTAGACACCTGTGCAAGCACTTGTGCAAGCACTTGTGCAAGCACTTGTGCAAGCACTTGTGCAAGCACAGGTGCAAGCACTTGTGCAGATACCAATGTAGACACCTGTGCAAGCACTTGTGCAAGCACTTGTGCAAGCACTTGTGCAAGCACTTGTGCAAGCACAGGTGCAAGCACTTGTGCAGATACCAATGTAGACACCTGTGCAAGCACTTGTGCAAGCACTTGTGCAAGCACTTGTGCAAGCACTTGTGCAAGCACTTGTGCAAGCACAGAATAC |
| 155 | BF07/IpTC | Burkina Faso | DP | 2007 |  | I | [This study (gallardo@inia.es)](http://asf-referencelab.info/asf/images/files/publicaciones/Gallardo-et-al-2009a.pdf) | ATGCAGCCTACTCACCACGCAGAGATAAGCTTTCAGGATAGAGATACAGCTCTTCCAGACGCATGTTCATCTATATCGGATATTAGCCCCGTTACGTATCCGATCACATTACCTATTATTAAAAACATTTCCGTAACTGCTCATGGTATCAATCTTATCGATAAGTTTCCATCAAAGTTCTGCAGCTCTTACATACCCTTCCACTACGGAGGCAATGCAATTAAAACCCCCGATGATCCGGGTGCGATGATGATTACCTTTGCTTTGAAGCCACGGGAGGAATACCAACCCAGTGGTCATATTAACGTATCCAGAGCAAGAGAATTTTATATTAGTTGGGACACGGATTACGTGGGGTCTATCACTACGGCTGATCTTGTGGTATCGGCATCTGCTATTAACTT |  | [This study (gallardo@inia.es)](http://asf-referencelab.info/asf/images/files/publicaciones/Nix_et_al_2006.pdf) | AGTGCGTATACTTGTGCAAGCACTTGTGCAGATACCAATGTAGACACCTGTGCAAGCACTTGTGCAAGCACTTGTGCAAGCACTTGTGCAAGCACAGGTGCAAGCACTTGTGCAGATACCAATGTAGACACCTGTGCAAGCACTTGTGCAAGCACTTGTGCAAGCACTTGTGCAAGCACTTGTGCAAGCACAGGTGCAAGCACTTGTGCAGATACCAATGTAGACACCTGTGCAAGCACTTGTGCAAGCACTTGTGCAAGCACTTGTGCAAGCACTTGTGCAAGCACAGGTGCAAGCACTTGTGCAGATACCAATGTAGACACCTGTGCAAGCACTTGTGCAAGCACTTGTGCAAGCACTTGTGCAAGCACTTGTGCAAGCACTTGTGCAAGCACAGAATAC |
| 156 | BF07/CkAB | Burkina Faso | DP | 2007 |  | I | [This study (gallardo@inia.es)](http://asf-referencelab.info/asf/images/files/publicaciones/Gallardo-et-al-2009a.pdf) | ATGCAGCCTACTCACCACGCAGAGATAAGCTTTCAGGATAGAGATACAGCTCTTCCAGACGCATGTTCATCTATATCGGATATTAGCCCCGTTACGTATCCGATCACATTACCTATTATTAAAAACATTTCCGTAACTGCTCATGGTATCAATCTTATCGATAAGTTTCCATCAAAGTTCTGCAGCTCTTACATACCCTTCCACTACGGAGGCAATGCAATTAAAACCCCCGATGATCCGGGTGCGATGATGATTACCTTTGCTTTGAAGCCACGGGAGGAATACCAACCCAGTGGTCATATTAACGTATCCAGAGCAAGAGAATTTTATATTAGTTGGGACACGGATTACGTGGGGTCTATCACTACGGCTGATCTTGTGGTATCGGCATCTGCTATTAACTT |  | [This study (gallardo@inia.es)](http://asf-referencelab.info/asf/images/files/publicaciones/Nix_et_al_2006.pdf) | AGTGCGTATACTTGTGCAAGCACTTGTGCAGATACCAATGTAGACACCTGTGCAAGCACTTGTGCAAGCACTTGTGCAAGCACTTGTGCAAGCACTTGTGCAAGCACTTGTGCAAGCACAGGTGCAAGCACTTGTGCAGATACCAATGTAGACACCTGTGCAAGCACTTGTGCAAGCACTTGTGCAAGCACTTGTGCAAGCACTTGTGCAAGCACTTGTGCAAGCACAGGTGCAAGCACTTGTGCAGATACCAATGTAGACACCTGTGCAAGCACTTGTGCAAGCACTTGTGCAAGCACTTGTGCAAGCACTTGTGCAAGCACAGGTGCAAGCACTTGTGCAGATACCAATGTAGACACCTGTGCAAGCACCTGTGCAAACACCTGTGCAAGCACAGAATAC |
| 157 | BF07/CkAC | Burkina Faso | DP | 2007 |  | I | [This study (gallardo@inia.es)](http://asf-referencelab.info/asf/images/files/publicaciones/Gallardo-et-al-2009a.pdf) | ATGCAGCCTACTCACCACGCAGAGATAAGCTTTCAGGATAGAGATACAGCTCTTCCAGACGCATGTTCATCTATATCGGATATTAGCCCCGTTACGTATCCGATCACATTACCTATTATTAAAAACATTTCCGTAACTGCTCATGGTATCAATCTTATCGATAAGTTTCCATCAAAGTTCTGCAGCTCTTACATACCCTTCCACTACGGAGGCAATGCAATTAAAACCCCCGATGATCCGGGTGCGATGATGATTACCTTTGCTTTGAAGCCACGGGAGGAATACCAACCCAGTGGTCATATTAACGTATCCAGAGCAAGAGAATTTTATATTAGTTGGGACACGGATTACGTGGGGTCTATCACTACGGCTGATCTTGTGGTATCGGCATCTGCTATTAACTT |  | [This study (gallardo@inia.es)](http://asf-referencelab.info/asf/images/files/publicaciones/Nix_et_al_2006.pdf) | AGTGCGTATACTTGTGCAAGCACTTGTGCAGATACCAATGTAGACACCTGTGCAAGCACTTGTGCAAGCACTTGTGCAAGCACTTGTGCAAGCACTTGTGCAAGCACTTGTGCAAGCACAGGTGCAAGCACTTGTGCAGATACCAATGTAGACACCTGTGCAAGCACTTGTGCAAGCACTTGTGCAAGCACTTGTGCAAGCACTTGTGCAAGCACTTGTGCAAGCACAGGTGCAAGCACTTGTGCAGATACCAATGTAGACACCTGTGCAAGCACTTGTGCAAGCACTTGTGCAAGCACTTGTGCAAGCACTTGTGCAAGCACAGGTGCAAGCACTTGTGCAGATACCAATGTAGACACCTGTGCAAGCACCTGTGCAAACACCTGTGCAAGCACAGAATAC |
| 158 | 38/Ss/07 | Italy | DP | 2007 | FR668412 | I | [Giammarioli et al 2011](http://asf-referencelab.info/asf/images/files/publicaciones/Gallardo-et-al-2009a.pdf) | ATGCAGCCTACTCACCACGCAGAGATAAGCTTTCAGGATAGAGATACAGCTCTTCCAGACGCATGTTCATCTATATCGGATATTAGCCCCGTTACGTATCCGATCACATTACCTATTATTAAAAACATTTCCGTAACTGCTCATGGTATCAATCTTATCGATAAGTTTCCATCAAAGTTCTGCAGCTCTTACATACCCTTCCACTACGGAGGCAATGCAATTAAAACCCCCGATGATCCGGGTGCGATGATGATTACCTTTGCTTTGAAGCCACGGGAGGAATACCAACCCAGTGGTCATATTAACGTATCCAGAGCAAGAGAATTTTATATTAGTTGGGACACGGATTACGTGGGGTCTATCACTACGGCTGATCTTGTGGTATCGGCATCTGCTATTAACTT | FR686544 | [Giammarioli et al 2011](http://asf-referencelab.info/asf/images/files/publicaciones/Nix_et_al_2006.pdf) | AGTGCGTATACCTGTGCAGATACCAATGTAGACACTTGTGCAAGCATGTGTGCATATACCAATGTAGACACCTGTGCAAGCATGTGTGCAGATACCAATGTAGATACCTGTGCAAGCACTTGTACAAGCACAGAATAC |
| 159 | 41/Og/07 | Italy | WP | 2007 | FR668413 | I | [Giammarioli et al 2011](http://asf-referencelab.info/asf/images/files/publicaciones/Gallardo-et-al-2009a.pdf) | ATGCAGCCTACTCACCACGCAGAGATAAGCTTTCAGGATAGAGATACAGCTCTTCCAGACGCATGTTCATCTATATCGGATATTAGCCCCGTTACGTATCCGATCACATTACCTATTATTAAAAACATTTCCGTAACTGCTCATGGTATCAATCTTATCGATAAGTTTCCATCAAAGTTCTGCAGCTCTTACATACCCTTCCACTACGGAGGCAATGCAATTAAAACCCCCGATGATCCGGGTGCGATGATGATTACCTTTGCTTTGAAGCCACGGGAGGAATACCAACCCAGTGGTCATATTAACGTATCCAGAGCAAGAGAATTTTATATTAGTTGGGACACGGATTACGTGGGGTCTATCACTACGGCTGATCTTGTGGTATCGGCATCTGCTATTAACTT | FR681799.2 | [Giammarioli et al 2011](http://asf-referencelab.info/asf/images/files/publicaciones/Nix_et_al_2006.pdf) | AGTGCGTATACCTGTGCAAGCACTTGTGCAGATACAAATGTAGACACCTGTGCAAGCACTTGTGCAAGCACTTGTGCAAGCACTTGTGCAAGCATGTGTGCAGATACCAATGTAGACACCTGTGCAAGCACCTGTGCAAACACCTGTGCAAGCACAGAATAC |
| 160 | 42/Og/07 | Italy | WP | 2007 | FR668414 | I | [Giammarioli et al 2011](http://asf-referencelab.info/asf/images/files/publicaciones/Gallardo-et-al-2009a.pdf) | ATGCAGCCTACTCACCACGCAGAGATAAGCTTTCAGGATAGAGATACAGCTCTTCCAGACGCATGTTCATCTATATCGGATATTAGCCCCGTTACGTATCCGATCACATTACCTATTATTAAAAACATTTCCGTAACTGCTCATGGTATCAATCTTATCGATAAGTTTCCATCAAAGTTCTGCAGCTCTTACATACCCTTCCACTACGGAGGCAATGCAATTAAAACCCCCGATGATCCGGGTGCGATGATGATTACCTTTGCTTTGAAGCCACGGGAGGAATACCAACCCAGTGGTCATATTAACGTATCCAGAGCAAGAGAATTTTATATTAGTTGGGACACGGATTACGTGGGGTCTATCACTACGGCTGATCTTGTGGTATCGGCATCTGCTATTAACTT | FR681800.2 | [Giammarioli et al 2011](http://asf-referencelab.info/asf/images/files/publicaciones/Nix_et_al_2006.pdf) | AGTGCGTATACCTGTGCAAGCACTTGTGCAGATACAAATGTAGACACCTGTGCAAGCACTTGTGCAAGCACTTGTGCAAGCACTTGTGCAAGCATGTGTGCAGATACCAATGTAGACACCTGTGCAAGCACCTGTGCAAACACCTGTGCAAGCACAGAATAC |
| 161 | 43/Og/07 | Italy | WP | 2007 | FR668415 | I | [Giammarioli et al 2011](http://asf-referencelab.info/asf/images/files/publicaciones/Gallardo-et-al-2009a.pdf) | ATGCAGCCTACTCACCACGCAGAGATAAGCTTTCAGGATAGAGATACAGCTCTTCCAGACGCATGTTCATCTATATCGGATATTAGCCCCGTTACGTATCCGATCACATTACCTATTATTAAAAACATTTCCGTAACTGCTCATGGTATCAATCTTATCGATAAGTTTCCATCAAAGTTCTGCAGCTCTTACATACCCTTCCACTACGGAGGCAATGCAATTAAAACCCCCGATGATCCGGGTGCGATGATGATTACCTTTGCTTTGAAGCCACGGGAGGAATACCAACCCAGTGGTCATATTAACGTATCCAGAGCAAGAGAATTTTATATTAGTTGGGACACGGATTACGTGGGGTCTATCACTACGGCTGATCTTGTGGTATCGGCATCTGCTATTAACTT | FR681801.2 | [Giammarioli et al 2011](http://asf-referencelab.info/asf/images/files/publicaciones/Nix_et_al_2006.pdf) | AGTGCGTATACCTGTGCAAGCACTTGCGCAGATACCAATGTAGACACCTGTGCAAGCACTTGTGCAAGCACTTGTGCAAGCACTTGTGCAAGCATGTGTGCAGATACCAATGTAGACACCTGTGCAAGCACCTGTGCAAACACCTGTGCAAGCACAGAATAC |
| 162 | Georgia2007 | Georgia | DP | 2007 | FR682468 | II | Chapman et al 2011 | ATGCAGCCCACTCACCACGCAGAGATAAGCTTTCAGGATAGAGATACAGCTCTTCCAGACGCATGTTCATCTATATCTGATATTAGCCCCGTTACGTATCCGATCACATTACCTATTATTAAAAACATTTCCGTAACTGCTCATGGTATCAATCTTATCGATAAATTTCCATCAAAGTTCTGCAGCTCTTACATACCCTTCCACTACGGAGGCAATGCGATTAAAACCCCCGATGATCCGGGTGCGATGATGATTACCTTTGCTTTGAAGCCACGGGAGGAATACCAACCCAGTGGTCATATTAACGTATCCAGAGCAAGAGAATTTTATATTAGTTGGGACACGGATTACGTGGGGTCTATCACTACGGCTGATCTTGTGGTATCGGCATCTGCTATTAACTT | FR682468 | Chapman et al 2011 | AGTGCGTATACCTGTGCAGATACCAATGTAGACACTTGTGCAAGCATGTGTGCAGATACCAATGTAGACACCTGTGCAAGCATGTGTGCAGATACCAATGTAGATACCTGTGCAAGCACTTGTACAAGCACAGAATAC |
| 163 | Abk07 | Georgia | DP | 2007 | JX857509 | II | [Gallardo et al 2014](http://asf-referencelab.info/asf/images/files/publicaciones/Gallardo-et-al-2009a.pdf) | ATGCAGCCCACTCACCACGCAGAGATAAGCTTTCAGGATAGAGATACAGCTCTTCCAGACGCATGTTCATCTATATCTGATATTAGCCCCGTTACGTATCCGATCACATTACCTATTATTAAAAACATTTCCGTAACTGCTCATGGTATCAATCTTATCGATAAATTTCCATCAAAGTTCTGCAGCTCTTACATACCCTTCCACTACGGAGGCAATGCGATTAAAACCCCCGATGATCCGGGTGCGATGATGATTACCTTTGCTTTGAAGCCACGGGAGGAATACCAACCCAGTGGTCATATTAACGTATCCAGAGCAAGAGAATTTTATATTAGTTGGGACACGGATTACGTGGGGTCTATCACTACGGCTGATCTTGTGGTATCGGCATCTGCTATTAACTT | JX857523 | [Gallardo et al 2014](http://asf-referencelab.info/asf/images/files/publicaciones/Nix_et_al_2006.pdf) | AGTGCGTATACCTGTGCAGATACCAATGTAGACACTTGTGCAAGCATGTGTGCAGATACCAATGTAGACACCTGTGCAAGCATGTGTGCAGATACCAATGTAGATACCTGTGCAAGCACTTGTACAAGCACAGAATAC |
| 164 | Arm07 | Armenia | DP | 2007 | JX857508 | II | [Gallardo et al 2014](http://wwwnc.cdc.gov/eid/article/20/9/14-0554_article) | ATGCAGCCCACTCACCACGCAGAGATAAGCTTTCAGGATAGAGATACAGCTCTTCCAGACGCATGTTCATCTATATCTGATATTAGCCCCGTTACGTATCCGATCACATTACCTATTATTAAAAACATTTCCGTAACTGCTCATGGTATCAATCTTATCGATAAATTTCCATCAAAGTTCTGCAGCTCTTACATACCCTTCCACTACGGAGGCAATGCGATTAAAACCCCCGATGATCCGGGTGCGATGATGATTACCTTTGCTTTGAAGCCACGGGAGGAATACCAACCCAGTGGTCATATTAACGTATCCAGAGCAAGAGAATTTTATATTAGTTGGGACACGGATTACGTGGGGTCTATCACTACGGCTGATCTTGTGGTATCGGCATCTGCTATTAACTT | JX857522 | [Gallardo et al 2014](http://asf-referencelab.info/asf/images/files/publicaciones/Nix_et_al_2006.pdf) | AGTGCGTATACCTGTGCAGATACCAATGTAGACACTTGTGCAAGCATGTGTGCAGATACCAATGTAGACACCTGTGCAAGCATGTGTGCAGATACCAATGTAGATACCTGTGCAAGCACTTGTACAAGCACAGAATAC |
| 165 | UG07.F7 | Uganda | DP | 2007 | GQ477143 | IX | [Gallardo et al 2011](http://asf-referencelab.info/asf/images/files/publicaciones/Gallardo-et-al-2009a.pdf) | ATGCAGCCTACCCACCACGCAGAGGTAAGCTTTCAGGATAGAGATACAGCTCTTCCAGATGCATGTTCATCCATATCTGATATTACCCCCATTACTTATCCGATCACATTACCTATTATTAAAAACATTTCCGTTACTGCTCACGGTATCAATCTTATCGATAAATTTCCATCAAAGTTCTGCAGCTCTTACATACCCTTCCACTACGGAGGCAATTCGATTAAAACCCCCGACGATCCGGGCGCGATGATGATTACCTTTGCTTTGAAACCACGGGAGGAATACCAACCCAGCGGTCATATTAACGTATCCAGAGCAAGAGAATTTTATATTAGCTGGGACACAGATTATGTGGGGTCTATCACCACGGCTGATCTTGTGGTATCGGCATCCGCTATTAACTT | GQ477157 | [Gallardo et al 2011](http://asf-referencelab.info/asf/images/files/publicaciones/Nix_et_al_2006.pdf) | AGTGCGTATACCTGTGCAAGCACTTGTGCAAGCACTTGTGCAAGCACTTGTGCAGACACCAATGTAGACACTTGTGCAAGCACTTGTGCAGACATTTGTGCAGACACCAATGTAGACACTTGTGCAAGCACTTGTGCAGATACTTGTGCAGACACTTGTGTAAGCACTTGTGTAAGCACTTGTGCAGACACCAATGTAGACACTTGTGCAAGCACTTGTGCAGACACCAATGTAGACACTTGTGTAAGCACTTGTGCAGACACCTGTGCAAGCACAGAATAC |
| 166 | UG07.F8 | Uganda | DP | 2007 | GQ477144 | IX | [Gallardo et al 2011](http://asf-referencelab.info/asf/images/files/publicaciones/Gallardo-et-al-2009a.pdf) | ATGCAGCCTACCCACCACGCAGAGGTAAGCTTTCAGGATAGAGATACAGCTCTTCCAGATGCATGTTCATCCATATCTGATATTACCCCCATTACTTATCCGATCACATTACCTATTATTAAAAACATTTCCGTTACTGCTCACGGTATCAATCTTATCGATAAATTTCCATCAAAGTTCTGCAGCTCTTACATACCCTTCCACTACGGAGGCAATTCGATTAAAACCCCCGACGATCCGGGCGCGATGATGATTACCTTTGCTTTGAAACCACGGGAGGAATACCAACCCAGCGGTCATATTAACGTATCCAGAGCAAGAGAATTTTATATTAGCTGGGACACAGATTATGTGGGGTCTATCACCACGGCTGATCTTGTGGTATCGGCATCCGCTATTAACTT | GQ477158 | [Gallardo et al 2011](http://asf-referencelab.info/asf/images/files/publicaciones/Nix_et_al_2006.pdf) | AGTGCGTATACCTGTGCAAGCACTTGTGCAAGCACTTGTGCAAGCACTTGTGCAGACACCAATGTAGACACTTGTGCAAGCACTTGTGCAGACATTTGTGCAGACACCAATGTAGACACTTGTGCAAGCACTTGTGCAGATACTTGTGCAGACACTTGTGTAAGCACTTGTGTAAGCACTTGTGCAGACACCAATGTAGACACTTGTGCAAGCACTTGTGCAGACACCAATGTAGACACTTGTGTAAGCACTTGTGCAGACACCTGTGCAAGCACAGAATAC |
| 167 | UG07.Mukono | Uganda | DP | 2007 | GQ477142 | IX | [Gallardo et al 2011](http://asf-referencelab.info/asf/images/files/publicaciones/Gallardo-et-al-2009a.pdf) | ATGCAGCCTACCCACCACGCAGAGGTAAGCTTTCAGGATAGAGATACAGCTCTTCCAGATGCATGTTCATCCATATCTGATATTACCCCCATTACTTATCCGATCACATTACCTATTATTAAAAACATTTCCGTTACTGCTCACGGTATCAATCTTATCGATAAATTTCCATCAAAGTTCTGCAGCTCTTACATACCCTTCCACTACGGAGGCAATTCGATTAAAACCCCCGACGATCCGGGCGCGATGATGATTACCTTTGCTTTGAAACCACGGGAGGAATACCAACCCAGCGGTCATATTAACGTATCCAGAGCAAGAGAATTTTATATTAGCTGGGACACAGATTATGTGGGGTCTATCACCACGGCTGATCTTGTGGTATCGGCATCCGCTATTAACTT | GQ477156 | [Gallardo et al 2011](http://asf-referencelab.info/asf/images/files/publicaciones/Nix_et_al_2006.pdf) | AGTGCGTATACCTGTGCAAGCACTTGTGCAAGCACTTGTGCAAGCACTTGTGCAGACACCAATGTAGACACTTGTGCAAGCACTTGTGCAGACATTTGTGCAGACACCAATGTAGACACTTGTGCAAGCACTTGTGCAGATACTTGTGCAGACACTTGTGTAAGCACTTGTGTAAGCACTTGTGCAGACACCAATGTAGACACTTGTGCAAGCACTTGTGCAGACACCAATGTAGACACTTGTGTAAGCACTTGTGCAGACACCTGTGCAAGCACAGAATAC |
| 168 | UG07.Wak1 | Uganda | DP | 2007 | GQ477138 | IX | [Gallardo et al 2011](http://asf-referencelab.info/asf/images/files/publicaciones/Gallardo_et_al_2011b_Uganda.pdf) | ATGCAGCCTACCCACCACGCAGAGGTAAGCTTTCAGGATAGAGATACAGCTCTTCCAGATGCATGTTCATCCATATCTGATATTACCCCCATTACTTATCCGATCACATTACCTATTATTAAAAACATTTCCGTTACTGCTCACGGTATCAATCTTATCGATAAATTTCCATCAAAGTTCTGCAGCTCTTACATACCCTTCCACTACGGAGGCAATTCGATTAAAACCCCCGACGATCCGGGCGCGATGATGATTACCTTTGCTTTGAAACCACGGGAGGAATACCAACCCAGCGGTCATATTAACGTATCCAGAGCAAGAGAATTTTATATTAGCTGGGACACAGATTATGTGGGGTCTATCACCACGGCTGATCTTGTGGTATCGGCATCCGCTATTAACTT | GQ477152 | [Gallardo et al 2011](http://asf-referencelab.info/asf/images/files/publicaciones/Nix_et_al_2006.pdf) | AGTGCGTATACCTGTGCAAGCACTTGTGCAAGCACTTGTGCAAGCACTTGTGCAGACACCAATGTAGACACTTGTGCAAGCACTTGTGCAGACATTTGTGCAGACACCAATGTAGACACTTGTGCAAGCACTTGTGCAGATACTTGTGCAGACACTTGTGTAAGCACTTGTGTAAGCACTTGTGCAGACACCAATGTAGACACTTGTGCAAGCACTTGTGCAGACACCAATGTAGACACTTGTGTAAGCACTTGTGCAGACACCTGTGCAAGCACAGAATAC |
| 169 | UG07.Wak2 | Uganda | DP | 2007 | GQ477139 | IX | [Gallardo et al 2011](http://asf-referencelab.info/asf/images/files/publicaciones/Gallardo-et-al-2009a.pdf) | ATGCAGCCTACCCACCACGCAGAGGTAAGCTTTCAGGATAGAGATACAGCTCTTCCAGATGCATGTTCATCCATATCTGATATTACCCCCATTACTTATCCGATCACATTACCTATTATTAAAAACATTTCCGTTACTGCTCACGGTATCAATCTTATCGATAAATTTCCATCAAAGTTCTGCAGCTCTTACATACCCTTCCACTACGGAGGCAATTCGATTAAAACCCCCGACGATCCGGGCGCGATGATGATTACCTTTGCTTTGAAACCACGGGAGGAATACCAACCCAGCGGTCATATTAACGTATCCAGAGCAAGAGAATTTTATATTAGCTGGGACACAGATTATGTGGGGTCTATCACCACGGCTGATCTTGTGGTATCGGCATCCGCTATTAACTT | GQ477153 | [Gallardo et al 2011](http://asf-referencelab.info/asf/images/files/publicaciones/Nix_et_al_2006.pdf) | AGTGCGTATACCTGTGCAAGCACTTGTGCAAGCACTTGTGCAAGCACTTGTGCAGACACCAATGTAGACACTTGTGCAAGCACTTGTGCAGACATTTGTGCAGACACCAATGTAGACACTTGTGCAAGCACTTGTGCAGATACTTGTGCAGACACTTGTGTAAGCACTTGTGTAAGCACTTGTGCAGACACCAATGTAGACACTTGTGCAAGCACTTGTGCAGACACCAATGTAGACACTTGTGTAAGCACTTGTGCAGACACCTGTGCAAGCACAGAATAC |
| 170 | UG07.Wak3 | Uganda | DP | 2007 | GQ477140 | IX | [Gallardo et al 2011](http://asf-referencelab.info/asf/images/files/publicaciones/Gallardo-et-al-2009a.pdf) | ATGCAGCCTACCCACCACGCAGAGGTAAGCTTTCAGGATAGAGATACAGCTCTTCCAGATGCATGTTCATCCATATCTGATATTACCCCCATTACTTATCCGATCACATTACCTATTATTAAAAACATTTCCGTTACTGCTCACGGTATCAATCTTATCGATAAATTTCCATCAAAGTTCTGCAGCTCTTACATACCCTTCCACTACGGAGGCAATTCGATTAAAACCCCCGACGATCCGGGCGCGATGATGATTACCTTTGCTTTGAAACCACGGGAGGAATACCAACCCAGCGGTCATATTAACGTATCCAGAGCAAGAGAATTTTATATTAGCTGGGACACAGATTATGTGGGGTCTATCACCACGGCTGATCTTGTGGTATCGGCATCCGCTATTAACTT | GQ477154 | [Gallardo et al 2011](http://asf-referencelab.info/asf/images/files/publicaciones/Nix_et_al_2006.pdf) | AGTGCGTATACCTGTGCAAGCACTTGTGCAAGCACTTGTGCAAGCACTTGTGCAGACACCAATGTAGACACTTGTGCAAGCACTTGTGCAGACATTTGTGCAGACACCAATGTAGACACTTGTGCAAGCACTTGTGCAGATACTTGTGCAGACACTTGTGTAAGCACTTGTGTAAGCACTTGTGCAGACACCAATGTAGACACTTGTGCAAGCACTTGTGCAGACACCAATGTAGACACTTGTGTAAGCACTTGTGCAGACACCTGTGCAAGCACAGAATAC |
| 171 | UG07.Wak4 | Uganda | DP | 2007 | GQ477141 | IX | [Gallardo et al 2011](http://asf-referencelab.info/asf/images/files/publicaciones/Gallardo-et-al-2009a.pdf) | ATGCAGCCTACCCACCACGCAGAGGTAAGCTTTCAGGATAGAGATACAGCTCTTCCAGATGCATGTTCATCCATATCTGATATTACCCCCATTACTTATCCGATCACATTACCTATTATTAAAAACATTTCCGTTACTGCTCACGGTATCAATCTTATCGATAAATTTCCATCAAAGTTCTGCAGCTCTTACATACCCTTCCACTACGGAGGCAATTCGATTAAAACCCCCGACGATCCGGGCGCGATGATGATTACCTTTGCTTTGAAACCACGGGAGGAATACCAACCCAGCGGTCATATTAACGTATCCAGAGCAAGAGAATTTTATATTAGCTGGGACACAGATTATGTGGGGTCTATCACCACGGCTGATCTTGTGGTATCGGCATCCGCTATTAACTT | GQ477155 | [Gallardo et al 2011](http://asf-referencelab.info/asf/images/files/publicaciones/Nix_et_al_2006.pdf) | AGTGCGTATACCTGTGCAAGCACTTGTGCAAGCACTTGTGCAAGCACTTGTGCAGACACCAATGTAGACACTTGTGCAAGCACTTGTGCAGACATTTGTGCAGACACCAATGTAGACACTTGTGCAAGCACTTGTGCAGATACTTGTGCAGACACTTGTGTAAGCACTTGTGTAAGCACTTGTGCAGACACCAATGTAGACACTTGTGCAAGCACTTGTGCAGACACCAATGTAGACACTTGTGTAAGCACTTGTGCAGACACCTGTGCAAGCACAGAATAC |
| 172 | GH07/GAPk4 | Ghana | DP | 2007 |  | I | [This study (gallardo@inia.es)](http://asf-referencelab.info/asf/images/files/publicaciones/Gallardo-et-al-2009a.pdf) | ATGCAGCCTACTCACCACGCAGAGATAAGCTTTCAGGATAGAGATACAGCTCTTCCAGACGCATGTTCATCTATATCGGATATTAGCCCCGTTACGTATCCGATCACATTACCTATTATTAAAAACATTTCCGTAACTGCTCATGGTATCAATCTTATCGATAAGTTTCCATCAAAGTTCTGCAGCTCTTACATACCCTTCCACTACGGAGGCAATGCAATTAAAACCCCCGATGATCCGGGTGCGATGATGATTACCTTTGCTTTGAAGCCACGGGAGGAATACCAACCCAGTGGTCATATTAACGTATCCAGAGCAAGAGAATTTTATATTAGTTGGGACACGGATTACGTGGGGTCTATCACTACGGCTGATCTTGTGGTATCGGCATCTGCTATTAACTT |  | [This study (gallardo@inia.es)](http://asf-referencelab.info/asf/images/files/publicaciones/Nix_et_al_2006.pdf) | AGTGCGTATACTTGTGCAAGCACTTGTGCAGATACCAATGTAGACACCTGTGCAAGCACTTGTGCAAGCACTTGTGCAAGCACTTGTGCAAGCACAGGTGCAAGCACTTGTGCAGATACCAATGTAGACACCTGTGCAAGCACTTGTGCAAGCACTTGTGCAAGCACTTGTGCAAGCACTTGTGCAAGCACTTGTGCAAGCACTTGTGCAAGCACAGGTGCAAGCACTTGTGCAGATACCAATGTAGACACCTGTGCAAGCACCTGTGCAAACACCTGTGCAAGCACAGAATAC |
| 173 | GH07/ERAs9 | Ghana | DP | 2007 |  | I | [This study (gallardo@inia.es)](http://asf-referencelab.info/asf/images/files/publicaciones/Gallardo-et-al-2009a.pdf) | ATGCAGCCTACTCACCACGCAGAGATAAGCTTTCAGGATAGAGATACAGCTCTTCCAGACGCATGTTCATCTATATCGGATATTAGCCCCGTTACGTATCCGATCACATTACCTATTATTAAAAACATTTCCGTAACTGCTCATGGTATCAATCTTATCGATAAGTTTCCATCAAAGTTCTGCAGCTCTTACATACCCTTCCACTACGGAGGCAATGCAATTAAAACCCCCGATGATCCGGGTGCGATGATGATTACCTTTGCTTTGAAGCCACGGGAGGAATACCAACCCAGTGGTCATATTAACGTATCCAGAGCAAGAGAATTTTATATTAGTTGGGACACGGATTACGTGGGGTCTATCACTACGGCTGATCTTGTGGTATCGGCATCTGCTATTAACTT |  | [This study (gallardo@inia.es)](http://asf-referencelab.info/asf/images/files/publicaciones/Nix_et_al_2006.pdf) | AGTGCGTATACTTGTGCAAGCACTTGTGCAGATACCAATGTAGACACCTGTGCAAGCACTTGTGCAAGCACTTGTGCAAGCACTTGTGCAAGCACAGGTGCAAGCACTTGTGCAGATACCAATGTAGACACCTGTGCAAGCACTTGTGCAAGCACTTGTGCAAGCACTTGTGCAAGCACTTGTGCAAGCACTTGTGCAAGCACTTGTGCAAGCACAGGTGCAAGCACTTGTGCAGATACCAATGTAGACACCTGTGCAAGCACCTGTGCAAACACCTGTGCAAGCACAGAATAC |
| 174 | GH07/GAPk6 | Ghana | DP | 2007 |  | I | [This study (gallardo@inia.es)](http://asf-referencelab.info/asf/images/files/publicaciones/Gallardo-et-al-2009a.pdf) | ATGCAGCCTACTCACCACGCAGAGATAAGCTTTCAGGATAGAGATACAGCTCTTCCAGACGCATGTTCATCTATATCGGATATTAGCCCCGTTACGTATCCGATCACATTACCTATTATTAAAAACATTTCCGTAACTGCTCATGGTATCAATCTTATCGATAAGTTTCCATCAAAGTTCTGCAGCTCTTACATACCCTTCCACTACGGAGGCAATGCAATTAAAACCCCCGATGATCCGGGTGCGATGATGATTACCTTTGCTTTGAAGCCACGGGAGGAATACCAACCCAGTGGTCATATTAACGTATCCAGAGCAAGAGAATTTTATATTAGTTGGGACACGGATTACGTGGGGTCTATCACTACGGCTGATCTTGTGGTATCGGCATCTGCTATTAACTT |  | [This study (gallardo@inia.es)](http://asf-referencelab.info/asf/images/files/publicaciones/Nix_et_al_2006.pdf) | AGTGCGTATACTTGTGCAAGCACTTGTGCAGATACCAATGTAGACACCTGTGCAAGCACTTGTGCAAGCACTTGTGCAAGCACTTGTGCAAGCACAGGTGCAAGCACTTGTGCAGATACCAATGTAGACACCTGTGCAAGCACTTGTGCAAGCACTTGTGCAAGCACTTGTGCAAGCACTTGTGCAAGCACTTGTGCAAGCACTTGTGCAAGCACAGGTGCAAGCACTTGTGCAGATACCAATGTAGACACCTGTGCAAGCACCTGTGCAAACACCTGTGCAAGCACAGAATAC |
| 175 | Che07 | Russia | Wild pig | 2007 | JX857510 | II | [Gallardo et al 2014](http://asf-referencelab.info/asf/images/files/publicaciones/Gallardo-et-al-2009a.pdf) | ATGCAGCCCACTCACCACGCAGAGATAAGCTTTCAGGATAGAGATACAGCTCTTCCAGACGCATGTTCATCTATATCTGATATTAGCCCCGTTACGTATCCGATCACATTACCTATTATTAAAAACATTTCCGTAACTGCTCATGGTATCAATCTTATCGATAAATTTCCATCAAAGTTCTGCAGCTCTTACATACCCTTCCACTACGGAGGCAATGCGATTAAAACCCCCGATGATCCGGGTGCGATGATGATTACCTTTGCTTTGAAGCCACGGGAGGAATACCAACCCAGTGGTCATATTAACGTATCCAGAGCAAGAGAATTTTATATTAGTTGGGACACGGATTACGTGGGGTCTATCACTACGGCTGATCTTGTGGTATCGGCATCTGCTATTAACTT | JX857524 | [Gallardo et al 2014](http://asf-referencelab.info/asf/images/files/publicaciones/Nix_et_al_2006.pdf) | AGTGCGTATACTTGTGCAGATACCAATGTAGACACTTGTGCAAGCATGTGTGCAGATACCAATGTAGACACCTGTGCAAGCATGTGTGCAGATACCAATGTAGATACCTGTGCAAGCACTTGTACAAGCACAGAATAC |
| 176 | Az08D | Azerbaijan | DP | 2008 | JX857515 | II | [Gallardo et al 2014](http://asf-referencelab.info/asf/images/files/publicaciones/Gallardo-et-al-2009a.pdf) | ATGCAGCCCACTCACCACGCAGAGATAAGCTTTCAGGATAGAGATACAGCTCTTCCAGACGCATGTTCATCTATATCTGATATTAGCCCCGTTACGTATCCGATCACATTACCTATTATTAAAAACATTTCCGTAACTGCTCATGGTATCAATCTTATCGATAAATTTCCATCAAAGTTCTGCAGCTCTTACATACCCTTCCACTACGGAGGCAATGCGATTAAAACCCCCGATGATCCGGGTGCGATGATGATTACCTTTGCTTTGAAGCCACGGGAGGAATACCAACCCAGTGGTCATATTAACGTATCCAGAGCAAGAGAATTTTATATTAGTTGGGACACGGATTACGTGGGGTCTATCACTACGGCTGATCTTGTGGTATCGGCATCTGCTATTAACTT | JX857530 | [Gallardo et al 2014](http://asf-referencelab.info/asf/images/files/publicaciones/Nix_et_al_2006.pdf) | AGTGCGTATACCTGTGCAGATACCAATGTAGACACTTGTGCAAGCATGTGTGCAGATACCAATGTAGACACCTGTGCAAGCATGTGTGCAGATACCAATGTAGATACCTGTGCAAGCACTTGTACAAGCACAGAATAC |
| 177 | Az08B | Azerbaijan | DP | 2008 | JX857516 | II | [Gallardo et al 2014](http://asf-referencelab.info/asf/images/files/publicaciones/Gallardo-et-al-2009a.pdf) | ATGCAGCCCACTCACCACGCAGAGATAAGCTTTCAGGATAGAGATACAGCTCTTCCAGACGCATGTTCATCTATATCTGATATTAGCCCCGTTACGTATCCGATCACATTACCTATTATTAAAAACATTTCCGTAACTGCTCATGGTATCAATCTTATCGATAAATTTCCATCAAAGTTCTGCAGCTCTTACATACCCTTCCACTACGGAGGCAATGCGATTAAAACCCCCGATGATCCGGGTGCGATGATGATTACCTTTGCTTTGAAGCCACGGGAGGAATACCAACCCAGTGGTCATATTAACGTATCCAGAGCAAGAGAATTTTATATTAGTTGGGACACGGATTACGTGGGGTCTATCACTACGGCTGATCTTGTGGTATCGGCATCTGCTATTAACTT | JX857529 | [Gallardo et al 2014](http://asf-referencelab.info/asf/images/files/publicaciones/Nix_et_al_2006.pdf) | AGTGCGTATACCTGTGCAGATACCAATGTAGACACTTGTGCAAGCATGTGTGCAGATACCAATGTAGACACCTGTGCAAGCATGTGTGCAGATACCAATGTAGATACCTGTGCAAGCACTTGTACAAGCACAGAATAC |
| 178 | TAN/08/MABIBO | Tanzania | DP | 2008 | GQ410766 | XV | [Misinzo,G. et al 2011](file:///C:\Users\Cgallardo\Dropbox\Carmina%20AGOSTO%202014\SECUENCIAS%20CRL\Desktop\Carmina%20MARZO%202014\ARTICULOS\VPPA\GENOTIPADO\2010%20%20Misinzo%20et%20al.pdf) | ATGCAGCCTACTCACCACGCAGAAATAAGCTTTCAGGACAGAGATACAGCTCTTCCAGACGCATGTTCATCTATATCTGATATTAATCCCGTTACTTATCCGATCACATTACCTATTATTAAAAACATTTCCGTAACAGCTCATGGGATCAATCTTATCGATAAGTTTCCATCAAAGTTCTGCAGCTCTTACATACCCTTCCACTACGGAGGCAATTCGATTAAAACCCCTGACGATCCGGGCGCGATGATGATTACCTTCGCTTTGAAGCCACGGGAGGAATACCAACCCAGTGGTCATATTAACGTATCCAGAGCAAGAGAATTTTATATTAGCTGGGACACGGATTATGTGGGATCTATCACCACGGCTGATCTTGTGGTATCGGCATCTGCTATTAACTT | GQ410770 | [Misinzo,G. and Van Doorsselaere,J.](http://asf-referencelab.info/asf/images/files/publicaciones/Nix_et_al_2006.pdf) | AGTGCGTATACTTGTGCAAGCACAAATGCAGACACCAATATAGATACCTGTGCAAGCACAAATGCAAGCACAAATACAGACACTAATGCAGATACCTGTGCAAGCACAAATGCAAGCACAAATGCAGACACCAATATAGATACCTGTGCAAGCACAAATGCAAGCACAAATACAGACACTAATGCAGATACCTGTGCAAGCACAAATGCAAGCACAAATACAGACACTAATGCAGATACCTGTGCAAGCACAAATGCAAGCACAAATGCAGACACCAATATAGATACCTGTGCAAGCACAAATGCAAGCACAAATGCAAGCACAAATACAGACACTAATACAGACACCAATATAGACACTAATACAGACATCAATGCAAACACAGAATAC |
| 179 | TAN/08/MAZIMBU | Tanzania | DP | 2008 | KJ028027 | XV | [Misinzo,G. et al 2011](file:///C:\Users\Cgallardo\Dropbox\Carmina%20AGOSTO%202014\SECUENCIAS%20CRL\Desktop\Carmina%20MARZO%202014\ARTICULOS\VPPA\GENOTIPADO\2010%20%20Misinzo%20et%20al.pdf) | ATGCAGCCTACTCACCACGCAGAAATAAGCTTTCAGGACAGAGATACAGCTCTTCCAGACGCATGTTCATCTATATCTGATATTAATCCCGTTACTTATCCGATCACATTACCTATTATTAAAAACATTTCCGTAACAGCTCATGGGATCAATCTTATCGATAAGTTTCCATCAAAGTTCTGCAGCTCTTACATACCCTTCCACTACGGAGGCAATTCGATTAAAACCCCTGACGATCCGGGCGCGATGATGATTACCTTCGCTTTGAAGCCACGGGAGGAATACCAACCCAGTGGTCATATTAACGTATCCAGAGCAAGAGAATTTTATATTAGCTGGGACACGGATTATGTGGGATCTATCACCACGGCTGATCTTGTGGTATCGGCATCTGCTATTAACTT | GQ410769 | [Misinzo,G. and Van Doorsselaere,J.](http://asf-referencelab.info/asf/images/files/publicaciones/Nix_et_al_2006.pdf) | AGTGCGTATACTTGTGCAAGCACAAATGCAGACACCAATATAGATACCTGTGCAAGCACAAATGCAAGCACAAATACAGACACTAATGCAGATACCTGTGCAAGCACAAATGCAAGCACAAATGCAGACACCAATATAGATACCTGTGCAAGCACAAATGCAAGCACAAATACAGACACTAATGCAGATACCTGTGCAAGCACAAATGCAAGCACAAATACAGACACTAATGCAGATACCTGTGCAAGCACAAATGCAAGCACAAATGCAGACACCAATATAGATACCTGTGCAAGCACAAATGCAAGCACAAATGCAAGCACAAATACAGACACTAATACAGACACCAATATAGACACTAATACAGACATCAATGCAAACACAGAATAC |
| 180 | 46/Ca/08 | Italy | DP | 2008 | FR668416 | I | [Giammarioli et al 2011](http://asf-referencelab.info/asf/images/files/publicaciones/Gallardo-et-al-2009a.pdf) | ATGCAGCCTACTCACCACGCAGAGATAAGCTTTCAGGATAGAGATACAGCTCTTCCAGACGCATGTTCATCTATATCGGATATTAGCCCCGTTACGTATCCGATCACATTACCTATTATTAAAAACATTTCCGTAACTGCTCATGGTATCAATCTTATCGATAAGTTTCCATCAAAGTTCTGCAGCTCTTACATACCCTTCCACTACGGAGGCAATGCAATTAAAACCCCCGATGATCCGGGTGCGATGATGATTACCTTTGCTTTGAAGCCACGGGAGGAATACCAACCCAGTGGTCATATTAACGTATCCAGAGCAAGAGAATTTTATATTAGTTGGGACACGGATTACGTGGGGTCTATCACTACGGCTGATCTTGTGGTATCGGCATCTGCTATTAACTT | FR681802 | [Giammarioli et al 2011](http://asf-referencelab.info/asf/images/files/publicaciones/Gallardo-et-al-2009a.pdf) | AGTGCGTATACCTGTGCAAGCACTTGTGCAGATACCAATGTAGACACCTGTGCAAGCACTTGTGCAAGCACTTGTGCAAGCACTTGTGCAAGCACTTGTGCAAGCATGTGTGCAGATACCAATGTAGACACCTGTGCAAGCACCTGTGCAAACACCTGTGCAAGCACAGAATAC |
| 181 | 47/Ss/08 | Italy | DP | 2008 | FR668417 | I | [Giammarioli et al 2011](http://asf-referencelab.info/asf/images/files/publicaciones/Gallardo-et-al-2009a.pdf) | ATGCAGCCTACTCACCACGCAGAGATAAGCTTTCAGGATAGAGATACAGCTCTTCCAGACGCATGTTCATCTATATCGGATATTAGCCCCGTTACGTATCCGATCACATTACCTATTATTAAAAACATTTCCGTAACTGCTCATGGTATCAATCTTATCGATAAGTTTCCATCAAAGTTCTGCAGCTCTTACATACCCTTCCACTACGGAGGCAATGCAATTAAAACCCCCGATGATCCGGGTGCGATGATGATTACCTTTGCTTTGAAGCCACGGGAGGAATACCAACCCAGTGGTCATATTAACGTATCCAGAGCAAGAGAATTTTATATTAGTTGGGACACGGATTACGTGGGGTCTATCACTACGGCTGATCTTGTGGTATCGGCATCTGCTATTAACTT | FR681803 | [Giammarioli et al 2011](http://asf-referencelab.info/asf/images/files/publicaciones/Gallardo-et-al-2009a.pdf) | AGTGCGTATACCTGTGCAAGCACTTGTCAAGATACCAATGTACACACCTGTGCCAGCACTTGTGCAAGCACTTGTGCAAGCACTTGTGCAAGCACTTGTGCAAGCATGTGTGCAGATACCAATGTACACACCTGTGCAAGCACCTGTGCAAACACCTGTGCAAGCACAGAATAC |
| 182 | 48/Ss/08 | Italy | DP | 2008 | FR668418 | I | [Giammarioli et al 2011](http://asf-referencelab.info/asf/images/files/publicaciones/Gallardo-et-al-2009a.pdf) | ATGCAGCCTACTCACCACGCAGAGATAAGCTTTCAGGATAGAGATACAGCTCTTCCAGACGCATGTTCATCTATATCGGATATTAGCCCCGTTACGTATCCGATCACATTACCTATTATTAAAAACATTTCCGTAACTGCTCATGGTATCAATCTTATCGATAAGTTTCCATCAAAGTTCTGCAGCTCTTACATACCCTTCCACTACGGAGGCAATGCAATTAAAACCCCCGATGATCCGGGTGCGATGATGATTACCTTTGCTTTGAAGCCACGGGAGGAATACCAACCCAGTGGTCATATTAACGTATCCAGAGCAAGAGAATTTTATATTAGTTGGGACACGGATTACGTGGGGTCTATCACTACGGCTGATCTTGTGGTATCGGCATCTGCTATTAACTT | FR681804 | [Giammarioli et al 2011](http://asf-referencelab.info/asf/images/files/publicaciones/Gallardo-et-al-2009a.pdf) | AGTGCGTATACCTGTGCAAGCACTTGTGCAGATACCAATGTAGACACCTGTGCAAGCACTTGTGCAAGCACTTGTGCAAGCACTTGTGCAAGCATGTGTGCAGATACGTGTGCAGATACCAATGTCGACACCTGTGCAAGCACCTGTGCAAACACCTGTGCAAGCACAGAATAC |
| 183 | Ken08BP/HB | Kenya | Wild pig | 2008 | JN590911 | X | [This study (gallardo@inia.es)](http://asf-referencelab.info/asf/images/files/publicaciones/Gallardo-et-al-2009a.pdf) | ATGCAGCCTACCCACCACGCAGAGGTAAGCTTTCAGGATAGAGATACAGCTCTTCCAGATGCATGTTCATCCATATCAGATATTTCCCCCATTACTTATCCGATCACGTTACCTATTATTAAAAACATTTCCGTCACTGCTCATGGTATCAATCTTATCGATAAATTTCCATCAAAGTTCTGCAGCTCTTACATACCCTTTCACTACGGAGGCAATTCGATTAAAACCCCCGACGATCCGGGCGCGATGATGATTACCTTTGCTTTGAAACCACGGGAGGAATACCAACCCAGTGGTCATATTAACGTATCCAGAGCAAGAGAGTTTTATATTAGCTGGGACACAGATTATGTGGGGTCTATCACCACGGCCGATCTTGTGGTATCGGCATCCGCTATTAACTT | JN590917 | [This study (gallardo@inia.es)](http://asf-referencelab.info/asf/images/files/publicaciones/Nix_et_al_2006.pdf) | AGTGCGTATACCTGTGCAAGCACTTGTGCAAGCACTTGTGCAAGCACCTGTGCAGACACCAATGTAGACACTTGTGCAAGCACTTGTGCAAGCACTTGTGCAAGCACTTGTGCAAGCACTTGTGCAGACACCTGTGCAAGCACAGAATACACCGAT |
| 184 | Ken08DP/Ndhiwa | Kenya | DP | 2008 | JN590912 | X | [This study (gallardo@inia.es)](http://asf-referencelab.info/asf/images/files/publicaciones/Gallardo-et-al-2009a.pdf) | ATGCAGCCTACCCACCACGCAGAGGTAAGCTTTCAGGATAGAGATACAGCTCTTCCAGATGCATGTTCATCCATATCAGATATTTCCCCCATTACTTATCCGATCACGTTACCTATTATTAAAAACATTTCCGTCACTGCTCATGGTATCAATCTTATCGATAAATTTCCATCAAAGTTCTGCAGCTCTTACATACCCTTTCACTACGGAGGCAATTCGATTAAAACCCCCGACGATCCGGGCGCGATGATGATTACCTTTGCTTTGAAACCACGGGAGGAATACCAACCCAGTGGTCATATTAACGTATCCAGAGCAAGAGAGTTTTATATTAGCTGGGACACAGATTATGTGGGGTCTATCACCACGGCCGATCTTGTGGTATCGGCATCCGCTATTAACTT | JN590918 | [This study (gallardo@inia.es)](http://asf-referencelab.info/asf/images/files/publicaciones/Nix_et_al_2006.pdf) | AGTGCGTATACCTGTGCAAGCACTTGTGCAAGCACTTGTGCAAGCACCTGTGCAGACACCAATGTAGACACTTGTGCAAGCACTTGTGCAAGCACTTGTGCAAGCACTTGTGCAAGCACTTGTGCAGACACCTGTGCAAGCACAGAATACACCGAT |
| 185 | Ken08DP/Nyarongi | Kenya | DP | 2008 | JN590913 | X | [This study (gallardo@inia.es)](http://asf-referencelab.info/asf/images/files/publicaciones/Gallardo-et-al-2009a.pdf) | ATGCAGCCTACCCACCACGCAGAGGTAAGCTTTCAGGATAGAGATACAGCTCTTCCAGATGCATGTTCATCCATATCAGATATTTCCCCCATTACTTATCCGATCACGTTACCTATTATTAAAAACATTTCCGTCACTGCTCATGGTATCAATCTTATCGATAAATTTCCATCAAAGTTCTGCAGCTCTTACATACCCTTTCACTACGGAGGCAATTCGATTAAAACCCCCGACGATCCGGGCGCGATGATGATTACCTTTGCTTTGAAACCACGGGAGGAATACCAACCCAGTGGTCATATTAACGTATCCAGAGCAAGAGAGTTTTATATTAGCTGGGACACAGATTATGTGGGGTCTATCACCACGGCCGATCTTGTGGTATCGGCATCCGCTATTAACTT | JN590919 | [This study (gallardo@inia.es)](http://asf-referencelab.info/asf/images/files/publicaciones/Nix_et_al_2006.pdf) | AGTGCGTATACCTGTGCAAGCACTTGTGCAAGCACTTGTGCAAGCACCTGTGCAGACACCAATGTAGACACTTGTGCAAGCACTTGTGCAAGCACTTGTGCAAGCACTTGTGCAAGCACTTGTGCAGACACCTGTGCAAGCACAGAATACACCGAT |
| 186 | Nig08/BNGb10 | Nigeria | DP | 2008 |  | I | [This study (gallardo@inia.es)](http://asf-referencelab.info/asf/images/files/publicaciones/Gallardo-et-al-2009a.pdf) | ATGCAGCCTACTCACCACGCAGAGATAAGCTTTCAGGATAGAGATACAGCTCTTCCAGACGCATGTTCATCTATATCGGATATTAGCCCCGTTACGTATCCGATCACATTACCTATTATTAAAAACATTTCCGTAACTGCTCATGGTATCAATCTTATCGATAAGTTTCCATCAAAGTTCTGCAGCTCTTACATACCCTTCCACTACGGAGGCAATGCAATTAAAACCCCCGATGATCCGGGTGCGATGATGATTACCTTTGCTTTGAAGCCACGGGAGGAATACCAACCCAGTGGTCATATTAACGTATCCAGAGCAAGAGAATTTTATATTAGTTGGGACACGGATTACGTGGGGTCTATCACTACGGCTGATCTTGTGGTATCGGCATCTGCTATTAACTT |  | [This study (gallardo@inia.es)](http://asf-referencelab.info/asf/images/files/publicaciones/Nix_et_al_2006.pdf) | AGTGCGTATACTTGTGCAAGCACTTGTGCAGATACCAATGTAGACACCTGTGCAAGCACTTGTGCAGATACCAATGTAGACACCTGTGCAAGCACTTGTGCAAGCACTTGTGCAAGCACTTGTGCAAGCACTTGTGCAAGCACAGGTGCAAGCACTTGTGCAGATACCAATGTAGACACCTGTGCAAGCACCTGTGCAAACACCTGTGCAAGCACAGAATAC |
| 187 | Nig08/BNGb15 | Nigeria | DP | 2008 |  | I | [This study (gallardo@inia.es)](http://asf-referencelab.info/asf/images/files/publicaciones/Gallardo-et-al-2009a.pdf) | ATGCAGCCTACTCACCACGCAGAGATAAGCTTTCAGGATAGAGATACAGCTCTTCCAGACGCATGTTCATCTATATCGGATATTAGCCCCGTTACGTATCCGATCACATTACCTATTATTAAAAACATTTCCGTAACTGCTCATGGTATCAATCTTATCGATAAGTTTCCATCAAAGTTCTGCAGCTCTTACATACCCTTCCACTACGGAGGCAATGCAATTAAAACCCCCGATGATCCGGGTGCGATGATGATTACCTTTGCTTTGAAGCCACGGGAGGAATACCAACCCAGTGGTCATATTAACGTATCCAGAGCAAGAGAATTTTATATTAGTTGGGACACGGATTACGTGGGGTCTATCACTACGGCTGATCTTGTGGTATCGGCATCTGCTATTAACTT |  | [This study (gallardo@inia.es)](http://asf-referencelab.info/asf/images/files/publicaciones/Nix_et_al_2006.pdf) | AGTGCGTATACTTGTGCAAGCACTTGTGCAGATACCAATGTAGACACCTGTGCAAGCACTTGTGCAAGCACTTGTGCAAGCACTTGTGCAAGCACAGGTGCAAGCACTTGTGCAGATACCAATGTAGACACCTGTGCAAGCACTTGTGCAAGCACTTGTGCAAGCACTTGTGCAAGCACAGGTGCAAGCACTTGTGCAGATACCAATGTAGACACCTGTGCAAGCACTTGTGCAAGCACTTGTGCAAGCACTTGTGCAAGCACAGGTGCAAGCACTTGTGCAGATACCAATGTAGACACCTGTGCAAGCACCTGTGCAAACACCTGTGCAAGCACAGAATAC |
| 188 | Nig08/BNGb2 | Nigeria | DP | 2008 |  | I | [This study (gallardo@inia.es)](http://asf-referencelab.info/asf/images/files/publicaciones/Gallardo-et-al-2009a.pdf) | ATGCAGCCTACTCACCACGCAGAGATAAGCTTTCAGGATAGAGATACAGCTCTTCCAGACGCATGTTCATCTATATCGGATATTAGCCCCGTTACGTATCCGATCACATTACCTATTATTAAAAACATTTCCGTAACTGCTCATGGTATCAATCTTATCGATAAGTTTCCATCAAAGTTCTGCAGCTCTTACATACCCTTCCACTACGGAGGCAATGCAATTAAAACCCCCGATGATCCGGGTGCGATGATGATTACCTTTGCTTTGAAGCCACGGGAGGAATACCAACCCAGTGGTCATATTAACGTATCCAGAGCAAGAGAATTTTATATTAGTTGGGACACGGATTACGTGGGGTCTATCACTACGGCTGATCTTGTGGTATCGGCATCTGCTATTAACTT |  | [This study (gallardo@inia.es)](http://asf-referencelab.info/asf/images/files/publicaciones/Nix_et_al_2006.pdf) | AGTGCGTATACTTGTGCAAGCACTTGTGCAGATACCAATGTAGACACCTGTGCAAGCACTTGTGCAAGCACTTGTGCAAGCACTTGTGCAAGCACAGGTGCAAGCACTTGTGCAGATACCAATGTAGACACCTGTGCAAGCACTTGTGCAAGCACTTGTGCAAGCACTTGTGCAAGCACAGGTGCAAGCACTTGTGCAGATACCAATGTAGACACCTGTGCAAGCACTTGTGCAAGCACTTGTGCAAGCACTTGTGCAAGCACAGGTGCAAGCACTTGTGCAGATACCAATGTAGACACCTGTGCAAGCACTTGTGCAAGCACTTGTGCAAGCACTTGTGCAAGCACAGGTGCAAGCACTTGTGCAGATACCAATGTAGACACCTGTGCAAGCACCTGTGCAAACACCTGTGCAAGCACAGAATAC |
| 189 | Nig08/BNGb24 | Nigeria | DP | 2008 |  | I | [This study (gallardo@inia.es)](http://asf-referencelab.info/asf/images/files/publicaciones/Gallardo-et-al-2009a.pdf) | ATGCAGCCTACTCACCACGCAGAGATAAGCTTTCAGGATAGAGATACAGCTCTTCCAGACGCATGTTCATCTATATCGGATATTAGCCCCGTTACGTATCCGATCACATTACCTATTATTAAAAACATTTCCGTAACTGCTCATGGTATCAATCTTATCGATAAGTTTCCATCAAAGTTCTGCAGCTCTTACATACCCTTCCACTACGGAGGCAATGCAATTAAAACCCCCGATGATCCGGGTGCGATGATGATTACCTTTGCTTTGAAGCCACGGGAGGAATACCAACCCAGTGGTCATATTAACGTATCCAGAGCAAGAGAATTTTATATTAGTTGGGACACGGATTACGTGGGGTCTATCACTACGGCTGATCTTGTGGTATCGGCATCTGCTATTAACTT |  | [This study (gallardo@inia.es)](http://asf-referencelab.info/asf/images/files/publicaciones/Nix_et_al_2006.pdf) | AGTGCGTATACTTGTGCAAGCACTTGTGCAGATACCAATGTAGACACCTGTGCAAGCACTTGTGCAAGCACTTGTGCAAGCACTTGTGCAAGCACTTGTGCAAGCACAGGTGCAAGCACTTGTGCAGATACCAATGTAGACACCTGTGCAAGCACTTGTGCAAGCACTTGTGCAAGCACTTGTGCAAGCACTTGTGCAAGCACAGGTGCAAGCACTTGTGCAGATACCAATGTAGACACCTGTGCAAGCACCTGTGCAAACACCTGTGCAAGCACAGAATAC |
| 190 | Nig08/BNGb4 | Nigeria | DP | 2008 |  | I | [This study (gallardo@inia.es)](http://asf-referencelab.info/asf/images/files/publicaciones/Gallardo-et-al-2009a.pdf) | ATGCAGCCTACTCACCACGCAGAGATAAGCTTTCAGGATAGAGATACAGCTCTTCCAGACGCATGTTCATCTATATCGGATATTAGCCCCGTTACGTATCCGATCACATTACCTATTATTAAAAACATTTCCGTAACTGCTCATGGTATCAATCTTATCGATAAGTTTCCATCAAAGTTCTGCAGCTCTTACATACCCTTCCACTACGGAGGCAATGCAATTAAAACCCCCGATGATCCGGGTGCGATGATGATTACCTTTGCTTTGAAGCCACGGGAGGAATACCAACCCAGTGGTCATATTAACGTATCCAGAGCAAGAGAATTTTATATTAGTTGGGACACGGATTACGTGGGGTCTATCACTACGGCTGATCTTGTGGTATCGGCATCTGCTATTAACTT |  | [This study (gallardo@inia.es)](http://asf-referencelab.info/asf/images/files/publicaciones/Nix_et_al_2006.pdf) | AGTGCGTATACTTGTGCAAGCACTTGTGCAGATACCAATGTAGACACCTGTGCAAGCACTTGTGCAAGCACTTGTGCAAGCACTTGTGCAAGCACAGGTGCAAGCACTTGTGCAGATACCAATGTAGACACCTGTGCAAGCACTTGTGCAAGCACTTGTGCAAGCACTTGTGCAAGCACAGGTGCAAGCACTTGTGCAGATACCAATGTAGACACCTGTGCAAGCACTTGTGCAAGCACTTGTGCAAGCACTTGTGCAAGCACAGGTGCAAGCACTTGTGCAGATACCAATGTAGACACCTGTGCAAGCACCTGTGCAAACACCTGTGCAAGCACAGAATAC |
| 191 | Nig08/BNGb6 | Nigeria | DP | 2008 |  | I | [This study (gallardo@inia.es)](http://asf-referencelab.info/asf/images/files/publicaciones/Gallardo-et-al-2009a.pdf) | ATGCAGCCTACTCACCACGCAGAGATAAGCTTTCAGGATAGAGATACAGCTCTTCCAGACGCATGTTCATCTATATCGGATATTAGCCCCGTTACGTATCCGATCACATTACCTATTATTAAAAACATTTCCGTAACTGCTCATGGTATCAATCTTATCGATAAGTTTCCATCAAAGTTCTGCAGCTCTTACATACCCTTCCACTACGGAGGCAATGCAATTAAAACCCCCGATGATCCGGGTGCGATGATGATTACCTTTGCTTTGAAGCCACGGGAGGAATACCAACCCAGTGGTCATATTAACGTATCCAGAGCAAGAGAATTTTATATTAGTTGGGACACGGATTACGTGGGGTCTATCACTACGGCTGATCTTGTGGTATCGGCATCTGCTATTAACTT |  | [This study (gallardo@inia.es)](http://asf-referencelab.info/asf/images/files/publicaciones/Nix_et_al_2006.pdf) | AGTGCGTATACTTGTGCAAGCACTTGTGCAGATACCAATGTAGACACCTGTGCAAGCACTTGTGCAGATACCAATGTAGACACCTGTGCAAGCACTTGTGCAAGCACTTGTGCAAGCACTTGTGCAAGCACTTGTGCAAGCACAGGTGCAAGCACTTGTGCAGATACCAATGTAGACACCTGTGCAAGCACCTGTGCAAACACCTGTGCAAGCACAGAATAC |
| 192 | Nig08/BNGb9 | Nigeria | DP | 2008 |  | I | [This study (gallardo@inia.es)](http://asf-referencelab.info/asf/images/files/publicaciones/Gallardo-et-al-2009a.pdf) | ATGCAGCCTACTCACCACGCAGAGATAAGCTTTCAGGATAGAGATACAGCTCTTCCAGACGCATGTTCATCTATATCGGATATTAGCCCCGTTACGTATCCGATCACATTACCTATTATTAAAAACATTTCCGTAACTGCTCATGGTATCAATCTTATCGATAAGTTTCCATCAAAGTTCTGCAGCTCTTACATACCCTTCCACTACGGAGGCAATGCAATTAAAACCCCCGATGATCCGGGTGCGATGATGATTACCTTTGCTTTGAAGCCACGGGAGGAATACCAACCCAGTGGTCATATTAACGTATCCAGAGCAAGAGAATTTTATATTAGTTGGGACACGGATTACGTGGGGTCTATCACTACGGCTGATCTTGTGGTATCGGCATCTGCTATTAACTT |  | [This study (gallardo@inia.es)](http://asf-referencelab.info/asf/images/files/publicaciones/Nix_et_al_2006.pdf) | AGTGCGTATACTTGTGCAAGCACTTGTGCAGATACCAATGTAGACACCTGTGCAAGCACTTGTGCAAGCACTTGTGCAAGCACTTGTGCAAGCACAGGTGCAAGCACTTGTGCAAGCACAGGTGCAAGCACTTGTGCAGATACCAATGTAGACACCTGTGCAAGCACTTGTGCAAGCACTTGTGCAAGCACTTGTGCAAGCACAGGTGCAAGCACTTGTGCAGATACCAATGTAGACACCTGTGCAAGCACCTGTGCAAACACCTGTGCAAGCACAGAATAC |
| 193 | Nig08/BNMk42 | Nigeria | DP | 2008 |  | I | [This study (gallardo@inia.es)](http://asf-referencelab.info/asf/images/files/publicaciones/Gallardo_et_al_2011c_Congo.pdf) | ATGCAGCCTACTCACCACGCAGAGATAAGCTTTCAGGATAGAGATACAGCTCTTCCAGACGCATGTTCATCTATATCGGATATTAGCCCCGTTACGTATCCGATCACATTACCTATTATTAAAAACATTTCCGTAACTGCTCATGGTATCAATCTTATCGATAAGTTTCCATCAAAGTTCTGCAGCTCTTACATACCCTTCCACTACGGAGGCAATGCAATTAAAACCCCCGATGATCCGGGTGCGATGATGATTACCTTTGCTTTGAAGCCACGGGAGGAATACCAACCCAGTGGTCATATTAACGTATCCAGAGCAAGAGAATTTTATATTAGTTGGGACACGGATTACGTGGGGTCTATCACTACGGCTGATCTTGTGGTATCGGCATCTGCTATTAACTT |  | [This study (gallardo@inia.es)](http://asf-referencelab.info/asf/images/files/publicaciones/Gallardo_et_al_2011c_Congo.pdf) | AGTGCGTATACTTGTGCAAGCACTTGTGCAGATACCAATGTAGACACCTGTGCAAGCACTTGTGCAGATACCAATGTAGACACCTGTGCAAGCACTTGTGCAAGCACTTGTGCAAGCACTTGTGCAAGCACTTGTGCAAGCACAGGTGCAAGCACTTGTGCAGATACCAATGTAGACACCTGTGCAAGCACCTGTGCAAACACCTGTGCAAGCACAGAATAC |
| 194 | Nig08/Cr15 | Nigeria | DP | 2008 |  | I | [This study (gallardo@inia.es)](http://asf-referencelab.info/asf/images/files/publicaciones/Gallardo_et_al_2011c_Congo.pdf) | ATGCAGCCTACTCACCACGCAGAGATAAGCTTTCAGGATAGAGATACAGCTCTTCCAGACGCATGTTCATCTATATCGGATATTAGCCCCGTTACGTATCCGATCACATTACCTATTATTAAAAACATTTCCGTAACTGCTCATGGTATCAATCTTATCGATAAGTTTCCATCAAAGTTCTGCAGCTCTTACATACCCTTCCACTACGGAGGCAATGCAATTAAAACCCCCGATGATCCGGGTGCGATGATGATTACCTTTGCTTTGAAGCCACGGGAGGAATACCAACCCAGTGGTCATATTAACGTATCCAGAGCAAGAGAATTTTATATTAGTTGGGACACGGATTACGTGGGGTCTATCACTACGGCTGATCTTGTGGTATCGGCATCTGCTATTAACTT |  | [This study (gallardo@inia.es)](http://asf-referencelab.info/asf/images/files/publicaciones/Gallardo_et_al_2011c_Congo.pdf) | AGTGCGTATACTTGTGCAAGCACTTGTGCAGATACCAATGTAGACACCTGTGCAAGCACTTGTGCAAGCACTTGTGCAAGCACTTGTGCAAGCACTTGTGCAAGCACAGGTGCAAGCACTTGTGCAGATACCAATGTAGACACCTGTGCAAGCACTTGTGCAAGCACTTGTGCAAGCACTTGTGCAAGCACTTGTGCAAGCACTTGTGCAAGCACAGGTGCAAGCACTTGTGCAGATACCAATGTAGACACCTGTGCAAGCACCTGTGCAAACACCTGTGCAAGCACAGAATAC |
| 195 | Nig08/LAOk1 | Nigeria | DP | 2008 |  | I | [This study (gallardo@inia.es)](http://asf-referencelab.info/asf/images/files/publicaciones/Gallardo_et_al_2011c_Congo.pdf) | ATGCAGCCTACTCACCACGCAGAGATAAGCTTTCAGGATAGAGATACAGCTCTTCCAGACGCATGTTCATCTATATCGGATATTAGCCCCGTTACGTATCCGATCACATTACCTATTATTAAAAACATTTCCGTAACTGCTCATGGTATCAATCTTATCGATAAGTTTCCATCAAAGTTCTGCAGCTCTTACATACCCTTCCACTACGGAGGCAATGCAATTAAAACCCCCGATGATCCGGGTGCGATGATGATTACCTTTGCTTTGAAGCCACGGGAGGAATACCAACCCAGTGGTCATATTAACGTATCCAGAGCAAGAGAATTTTATATTAGTTGGGACACGGATTACGTGGGGTCTATCACTACGGCTGATCTTGTGGTATCGGCATCTGCTATTAACTT |  | [This study (gallardo@inia.es)](http://asf-referencelab.info/asf/images/files/publicaciones/Gallardo_et_al_2011c_Congo.pdf) | AGTGCGTATACTTGTGCAAGCACTTGTGCAGATACCAATGTAGACACCTGTGCAAGCACTTGTGCAAGCACTTGTGCAAGCACTTGTGCAAGCACAGGTGCAAGCACTTGTGCAGATACCAATGTAGACACCTGTGCAAGCACTTGTGCAAGCACTTGTGCAAGCACTTGTGCAAGCACTTGTGCAAGCACAGGTGCAAGCACTTGTGCAGATACCAATGTAGACACCTGTGCAAGCACTTGTGCAAGCACTTGTGCAAGCACTTGTGCAAGCACTTGTGCAAGCACAGGTGCAAGCACTTGTGCAGATACCAATGTAGACACCTGTGCAAGCACTTGTGCAAGCACTTGTGCAAGCACTTGTGCAAGCACAGGTGCAAGCACTTGTGCAGATACCAATGTAGACACCTGTGCAAGCACCTGTGCAAACACCTGTGCAAGCACAGAATAC |
| 196 | Nig08/LAOk2 | Nigeria | DP | 2008 |  | I | [This study (gallardo@inia.es)](http://asf-referencelab.info/asf/images/files/publicaciones/Gallardo_et_al_2011c_Congo.pdf) | ATGCAGCCTACTCACCACGCAGAGATAAGCTTTCAGGATAGAGATACAGCTCTTCCAGACGCATGTTCATCTATATCGGATATTAGCCCCGTTACGTATCCGATCACATTACCTATTATTAAAAACATTTCCGTAACTGCTCATGGTATCAATCTTATCGATAAGTTTCCATCAAAGTTCTGCAGCTCTTACATACCCTTCCACTACGGAGGCAATGCAATTAAAACCCCCGATGATCCGGGTGCGATGATGATTACCTTTGCTTTGAAGCCACGGGAGGAATACCAACCCAGTGGTCATATTAACGTATCCAGAGCAAGAGAATTTTATATTAGTTGGGACACGGATTACGTGGGGTCTATCACTACGGCTGATCTTGTGGTATCGGCATCTGCTATTAACTT |  | [This study (gallardo@inia.es)](http://asf-referencelab.info/asf/images/files/publicaciones/Gallardo_et_al_2011c_Congo.pdf) | AGTGCGTATACTTGTGCAAGCACTTGTGCAGATACCAATGTAGACACCTGTGCAAGCACTTGTGCAAGCACTTGTGCAAGCACTTGTGCAAGCACAGGTGCAAGCACTTGTGCAGATACCAATGTAGACACCTGTGCAAGCACTTGTGCAAGCACTTGTGCAAGCACTTGTGCAAGCACTTGTGCAAGCACAGGTGCAAGCACTTGTGCAGATACCAATGTAGACACCTGTGCAAGCACTTGTGCAAGCACTTGTGCAAGCACTTGTGCAAGCACTTGTGCAAGCACAGGTGCAAGCACTTGTGCAGATACCAATGTAGACACCTGTGCAAGCACTTGTGCAAGCACTTGTGCAAGCACTTGTGCAAGCACTTGTGCAAGCACAGGTGCAAGCACTTGTGCAGATACCAATGTAGACACCTGTGCAAGCACCTGTGCAAACACCTGTGCAAGCACAGAATAC |
| 197 | Nig08/NW10 | Nigeria | DP | 2008 |  | I | [This study (gallardo@inia.es)](http://asf-referencelab.info/asf/images/files/publicaciones/Gallardo-et-al-2009a.pdf) | ATGCAGCCTACTCACCACGCAGAGATAAGCTTTCAGGATAGAGATACAGCTCTTCCAGACGCATGTTCATCTATATCGGATATTAGCCCCGTTACGTATCCGATCACATTACCTATTATTAAAAACATTTCCGTAACTGCTCATGGTATCAATCTTATCGATAAGTTTCCATCAAAGTTCTGCAGCTCTTACATACCCTTCCACTACGGAGGCAATGCAATTAAAACCCCCGATGATCCGGGTGCGATGATGATTACCTTTGCTTTGAAGCCACGGGAGGAATACCAACCCAGTGGTCATATTAACGTATCCAGAGCAAGAGAATTTTATATTAGTTGGGACACGGATTACGTGGGGTCTATCACTACGGCTGATCTTGTGGTATCGGCATCTGCTATTAACTT |  | [This study (gallardo@inia.es)](http://asf-referencelab.info/asf/images/files/publicaciones/Nix_et_al_2006.pdf) | AGTGCGTATACTTGTGCAAGCACTTGTGCAGATACCAATGTAGACACCTGTGCAAGCACTTGTGCAAGCACTTGTGCAAGCACTTGTGCAAGCACTTGTGCAAGCACAGGTGCAAGCACTTGTGCAAGCACAGGTGCAAGCACTTGTGCAGATACCAATGTAGACACCTGTGCAAGCACTTGTGCAAGCACTTGTGCAAGCACTTGTGCAAGCACAGGTGCAAGCACTTGTGCAAGCACAGGTGCAAGCACTTGTGCAGATACCAATGTAGACACCTGTGCAAGCACTTGTGCAAGCACAGGTGCAAGCACTTGTGCAGATACCAATGTAGACACCTGTGCAAGCACTTGTGCAAGCACTTGTGCAAGCACTTGTGCAAGCACAGGTGCAAGCACTTGTGCAGATACCAATGTAGACACCTGTGCAAGCACCTGTGCAAACACCTGTGCAAGCACAGAATAC |
| 198 | Nig08/NW12 | Nigeria | DP | 2008 |  | I | [This study (gallardo@inia.es)](http://asf-referencelab.info/asf/images/files/publicaciones/Gallardo-et-al-2009a.pdf) | ATGCAGCCTACTCACCACGCAGAGATAAGCTTTCAGGATAGAGATACAGCTCTTCCAGACGCATGTTCATCTATATCGGATATTAGCCCCGTTACGTATCCGATCACATTACCTATTATTAAAAACATTTCCGTAACTGCTCATGGTATCAATCTTATCGATAAGTTTCCATCAAAGTTCTGCAGCTCTTACATACCCTTCCACTACGGAGGCAATGCAATTAAAACCCCCGATGATCCGGGTGCGATGATGATTACCTTTGCTTTGAAGCCACGGGAGGAATACCAACCCAGTGGTCATATTAACGTATCCAGAGCAAGAGAATTTTATATTAGTTGGGACACGGATTACGTGGGGTCTATCACTACGGCTGATCTTGTGGTATCGGCATCTGCTATTAACTT |  | [This study (gallardo@inia.es)](http://asf-referencelab.info/asf/images/files/publicaciones/Nix_et_al_2006.pdf) | AGTGCGTATACTTGTGCAAGCACTTGTGCAGATACCAATGTAGACACCTGTGCAAGCACTTGTGCAAGCACTTGTGCAAGCACTTGTGCAAGCACTTGTGCAAGCACAGGTGCAAGCACTTGTGCAAGCACAGGTGCAAGCACTTGTGCAGATACCAATGTAGACACCTGTGCAAGCACTTGTGCAAGCACTTGTGCAAGCACTTGTGCAAGCACAGGTGCAAGCACTTGTGCAAGCACAGGTGCAAGCACTTGTGCAGATACCAATGTAGACACCTGTGCAAGCACTTGTGCAAGCACAGGTGCAAGCACTTGTGCAGATACCAATGTAGACACCTGTGCAAGCACTTGTGCAAGCACTTGTGCAAGCACTTGTGCAAGCACAGGTGCAAGCACTTGTGCAGATACCAATGTAGACACCTGTGCAAGCACCTGTGCAAACACCTGTGCAAGCACAGAATAC |
| 199 | Nig08/NW6 | Nigeria | DP | 2008 |  | I | [This study (gallardo@inia.es)](http://asf-referencelab.info/asf/images/files/publicaciones/Gallardo-et-al-2009a.pdf) | ATGCAGCCTACTCACCACGCAGAGATAAGCTTTCAGGATAGAGATACAGCTCTTCCAGACGCATGTTCATCTATATCGGATATTAGCCCCGTTACGTATCCGATCACATTACCTATTATTAAAAACATTTCCGTAACTGCTCATGGTATCAATCTTATCGATAAGTTTCCATCAAAGTTCTGCAGCTCTTACATACCCTTCCACTACGGAGGCAATGCAATTAAAACCCCCGATGATCCGGGTGCGATGATGATTACCTTTGCTTTGAAGCCACGGGAGGAATACCAACCCAGTGGTCATATTAACGTATCCAGAGCAAGAGAATTTTATATTAGTTGGGACACGGATTACGTGGGGTCTATCACTACGGCTGATCTTGTGGTATCGGCATCTGCTATTAACTT |  | [This study (gallardo@inia.es)](http://asf-referencelab.info/asf/images/files/publicaciones/Nix_et_al_2006.pdf) | AGTGCGTATACTTGTGCAAGCACTTGTGCAGATACCAATGTAGACACCTGTGCAAGCACTTGTGCAGATACCAATGTAGACACCTGTGCAAGCACTTGTGCAAGCACTTGTGCAAGCACTTGTGCAAGCACTTGTGCAAGCACAGGTGCAAGCACTTGTGCAGATACCAATGTAGACACCTGTGCAAGCACCTGTGCAAACACCTGTGCAAGCACAGAATAC |
| 200 | Oren08 | Russia | DP | 2008 | JX857512 | II | [Gallardo et al 2014](http://asf-referencelab.info/asf/images/files/publicaciones/Gallardo-et-al-2009a.pdf) | ATGCAGCCCACTCACCACGCAGAGATAAGCTTTCAGGATAGAGATACAGCTCTTCCAGACGCATGTTCATCTATATCTGATATTAGCCCCGTTACGTATCCGATCACATTACCTATTATTAAAAACATTTCCGTAACTGCTCATGGTATCAATCTTATCGATAAATTTCCATCAAAGTTCTGCAGCTCTTACATACCCTTCCACTACGGAGGCAATGCGATTAAAACCCCCGATGATCCGGGTGCGATGATGATTACCTTTGCTTTGAAGCCACGGGAGGAATACCAACCCAGTGGTCATATTAACGTATCCAGAGCAAGAGAATTTTATATTAGTTGGGACACGGATTACGTGGGGTCTATCACTACGGCTGATCTTGTGGTATCGGCATCTGCTATTAACTT | JX857526 | [Gallardo et al 2014](http://asf-referencelab.info/asf/images/files/publicaciones/Nix_et_al_2006.pdf) | AGTGCGTATACTTGTGCAGATACCAATGTAGACACTTGTGCAAGCATGTGTGCAGATACCAATGTAGACACCTGTGCAAGCATGTGTGCAGATACCAATGTAGATACCTGTGCAAGCACTTGTACAAGCACAGAATAC |
| 201 | NO08/Av | Russia | DP | 2008 | JX857513 | II | [Gallardo et al 2014](http://asf-referencelab.info/asf/images/files/publicaciones/Gallardo-et-al-2009a.pdf) | ATGCAGCCCACTCACCACGCAGAGATAAGCTTTCAGGATAGAGATACAGCTCTTCCAGACGCATGTTCATCTATATCTGATATTAGCCCCGTTACGTATCCGATCACATTACCTATTATTAAAAACATTTCCGTAACTGCTCATGGTATCAATCTTATCGATAAATTTCCATCAAAGTTCTGCAGCTCTTACATACCCTTCCACTACGGAGGCAATGCGATTAAAACCCCCGATGATCCGGGTGCGATGATGATTACCTTTGCTTTGAAGCCACGGGAGGAATACCAACCCAGTGGTCATATTAACGTATCCAGAGCAAGAGAATTTTATATTAGTTGGGACACGGATTACGTGGGGTCTATCACTACGGCTGATCTTGTGGTATCGGCATCTGCTATTAACTT | JX857527 | [Gallardo et al 2014](http://asf-referencelab.info/asf/images/files/publicaciones/Nix_et_al_2006.pdf) | AGTGCGTATACTTGTGCAGATACCAATGTAGACACTTGTGCAAGCATGTGTGCAGATACCAATGTAGACACCTGTGCAAGCATGTGTGCAGATACCAATGTAGATACCTGTGCAAGCACTTGTACAAGCACAGAATAC |
| 202 | Ing08 | Russia | Wild pig | 2008 | JX857511 | II | [Gallardo et al 2014](http://asf-referencelab.info/asf/images/files/publicaciones/Gallardo-et-al-2009a.pdf) | ATGCAGCCCACTCACCACGCAGAGATAAGCTTTCAGGATAGAGATACAGCTCTTCCAGACGCATGTTCATCTATATCTGATATTAGCCCCGTTACGTATCCGATCACATTACCTATTATTAAAAACATTTCCGTAACTGCTCATGGTATCAATCTTATCGATAAATTTCCATCAAAGTTCTGCAGCTCTTACATACCCTTCCACTACGGAGGCAATGCGATTAAAACCCCCGATGATCCGGGTGCGATGATGATTACCTTTGCTTTGAAGCCACGGGAGGAATACCAACCCAGTGGTCATATTAACGTATCCAGAGCAAGAGAATTTTATATTAGTTGGGACACGGATTACGTGGGGTCTATCACTACGGCTGATCTTGTGGTATCGGCATCTGCTATTAACTT | JX857525 | [Gallardo et al 2014](http://asf-referencelab.info/asf/images/files/publicaciones/Nix_et_al_2006.pdf) | AGTGCGTATACTTGTGCAGATACCAATGTAGACACTTGTGCAAGCATGTGTGCAGATACCAATGTAGACACCTGTGCAAGCATGTGTGCAGATACCAATGTAGATACCTGTGCAAGCACTTGTACAAGCACAGAATAC |
| 203 | NO08/Ap | Russia | DP | 2008 | JX857514 | II | [Gallardo et al 2014](http://asf-referencelab.info/asf/images/files/publicaciones/Gallardo-et-al-2009a.pdf) | ATGCAGCCCACTCACCACGCAGAGATAAGCTTTCAGGATAGAGATACAGCTCTTCCAGACGCATGTTCATCTATATCTGATATTAGCCCCGTTACGTATCCGATCACATTACCTATTATTAAAAACATTTCCGTAACTGCTCATGGTATCAATCTTATCGATAAATTTCCATCAAAGTTCTGCAGCTCTTACATACCCTTCCACTACGGAGGCAATGCGATTAAAACCCCCGATGATCCGGGTGCGATGATGATTACCTTTGCTTTGAAGCCACGGGAGGAATACCAACCCAGTGGTCATATTAACGTATCCAGAGCAAGAGAATTTTATATTAGTTGGGACACGGATTACGTGGGGTCTATCACTACGGCTGATCTTGTGGTATCGGCATCTGCTATTAACTT | JX857528 | [Gallardo et al 2014](http://asf-referencelab.info/asf/images/files/publicaciones/Nix_et_al_2006.pdf) | AGTGCGTATACTTGTGCAGATACCAATGTAGACACTTGTGCAAGCATGTGTGCAGATACCAATGTAGACACCTGTGCAAGCATGTGTGCAGATACCAATGTAGATACCTGTGCAAGCACTTGTACAAGCACAGAATAC |
| 204 | Ken08Tk.2/1 | Kenya | Tick | 2008 | HM745275 | X | [Gallardo et al 2011](http://asf-referencelab.info/asf/images/files/publicaciones/Gallardo-et-al-2009a.pdf) | ATGCAGCCTACCCACCACGCAGAGGTAAGCTTTCAGGATAGAGATACAGCTCTTCCAGATGCATGTTCATCCATATCAGATATTACCCCCATTACTTATCCGATCACGTTACCTATTATTAAAAACATTTCCGTCACTGCTCATGGTATCAATCTTATCGATAAATTTCCATCAAAGTTCTGCAGCTCTTACATACCCTTTCACTACGGAGGCAATTCGATTAAAACCCCCGACGATCCGGGCGCGATGATGATTACCTTTGCTTTGAAACCACGGGAGGAATACCAACCCAGCGGTCATATTAACGTATCCAGAGCAAGAGAATTTTATATTAGCTGGGACACAGATTATGTGGGGTCTATCACCACGGCCGATCTTGTGGTATCGGCATCCGCTATTAACTT | HM745310 | [Gallardo et al 2011](http://asf-referencelab.info/asf/images/files/publicaciones/Nix_et_al_2006.pdf) | AGTGCGTATACCTGTGCAAGCACCTGTGCAAGCACCTGTGCAGACACCAATGTAGACACTTGTGCAAGCACTTGTGTAAGCACCTGTGCAGACACTTGTGCAGACACCTGTGCAAGCACAGAATAC |
| 205 | Ken08Tk.2/3 | Kenya | Tick | 2008 | HM745276 | X | [Gallardo et al 2011](http://asf-referencelab.info/asf/images/files/publicaciones/Gallardo-et-al-2009a.pdf) | ATGCAGCCTACCCACCACGCAGAGGTAAGCTTTCAGGATAGAGATACAGCTCTTCCAGATGCATGTTCATCCATATCAGATATTACCCCCATTACTTATCCGATCACGTTACCTATTATTAAAAACATTTCCGTCACTGCTCATGGTATCAATCTTATCGATAAATTTCCATCAAAGTTCTGCAGCTCTTACATACCCTTTCACTACGGAGGCAATTCGATTAAAACCCCCGACGATCCGGGCGCGATGATGATTACCTTTGCTTTGAAACCACGGGAGGAATACCAACCCAGTGGTCATATTAACGTATCCAGAGCAAGAGAATTTTATATTAGCTGGGACACAGATTATGTGGGGTCTATCACCACGGCCGATCTTGTGGTATCGGCATCCGCTATTAACTT | HM745311 | [Gallardo et al 2011](http://asf-referencelab.info/asf/images/files/publicaciones/Nix_et_al_2006.pdf) | AGTGCGTATACCTGTGCAAGCACCTGTGCAAGCACCTGTGCAGACACCAATGTAGACACTTGTGCAAGCACTTGTGTAAGCACCTGTGCAGACACTTGTGCAGACACCTGTGCAAGCACAGAATAC |
| 205 | Pol15/WB-02978#38 | Poland | Wild pig | 2008 |  | II | [This study (gallardo@inia.es)](http://asf-referencelab.info/asf/images/files/publicaciones/Gallardo-et-al-2009a.pdf) | ATGCAGCCCACTCACCACGCAGAGATAAGCTTTCAGGATAGAGATACAGCTCTTCCAGACGCATGTTCATCTATATCTGATATTAGCCCCGTTACGTATCCGATCACATTACCTATTATTAAAAACATTTCCGTAACTGCTCATGGTATCAATCTTATCGATAAATTTCCATCAAAGTTCTGCAGCTCTTACATACCCTTCCACTACGGAGGCAATGCGATTAAAACCCCCGATGATCCGGGTGCGATGATGATTACCTTTGCTTTGAAGCCACGGGAGGAATACCAACCCAGTGGTCATATTAACGTATCCAGAGCAAGAGAATTTTATATTAGTTGGGACACGGATTACGTGGGGTCTATCACTACGGCTGATCTTGTGGTATCGGCATCTGCTATTAACTT |  | [This study (gallardo@inia.es)](http://asf-referencelab.info/asf/images/files/publicaciones/Nix_et_al_2006.pdf) | AGTGCGTATACTTGTGCAGATACCAATGTAGACACTTGTGCAAGCATGTGTGCAGATACCAATGTAGACACCTGTGCAAGCATGTGTGCAGATACCAATGTAGATACCTGTGCAAGCACTTGTACAAGCACAGAATAC |
| 206 | Ken08WH/4 | Kenya | Wild pig | 2008 | HM745285 | IX | [Gallardo et al 2011](http://asf-referencelab.info/asf/images/files/publicaciones/Gallardo-et-al-2009a.pdf) | ATGCAGCCTACCCACCACGCAGAGGTAAGCTTTCAGGATAGAGATACAGCTCTTCCAGATGCATGTTCATCCATATCTGATATTACCCCCATTACTTATCCGATCACATTACCTATTATTAAAAACATTTCCGTTACTGCTCACGGTATCAATCTTATCGATAAATTTCCATCAAAGTTCTGCAGCTCTTACATACCCTTCCACTACGGAGGCAATTCGATTAAAACCCCCGACGATCCGGGCGCGATGATGATTACCTTTGCTTTGAAACCACGGGAGGAATACCAACCCAGCGGTCATATTAACGTATCCAGAGCAAGAGAATTTTATATTAGCTGGGACACAGATTATGTGGGGTCTATCACCACGGCTGATCTTGTGGTATCGGCATCCGCTATTAACTT | HM745320 | [Gallardo et al 2011](http://asf-referencelab.info/asf/images/files/publicaciones/Nix_et_al_2006.pdf) | AGTGCGTATACCTGTGCAAGCACTTGTGCAAGCACTTGTGCAAGCACTTGTGCAGACACCAATGTAGACACTTGTGCAAGCACTTGTGCAGACATTTGTGCAGACACCAATGTAGACACTTGTGCAAGCACTTGTGCAGATACTTGTGCAGACACTTGTGTAAGCACTTGTGTAAGCACTTGTGCAGACACCAATGTAGACACTTGTGCAAGCACTTGTGCAGACACCAATGTAGACACTTGTGTAAGCACTTGTGCAGACACCTGTGCAAGCACAGAATAC |
| 207 | Ken08WH/5 | Kenya | Wild pig | 2008 | HM745286 | IX | [Gallardo et al 2011](http://asf-referencelab.info/asf/images/files/publicaciones/Gallardo-et-al-2009a.pdf) | ATGCAGCCTACCCACCACGCAGAGGTAAGCTTTCAGGATAGAGATACAGCTCTTCCAGATGCATGTTCATCCATATCTGATATTACCCCCATTACTTATCCGATCACATTACCTATTATTAAAAACATTTCCGTTACTGCTCACGGTATCAATCTTATCGATAAATTTCCATCAAAGTTCTGCAGCTCTTACATACCCTTCCACTACGGAGGCAATTCGATTAAAACCCCCGACGATCCGGGCGCGATGATGATTACCTTTGCTTTGAAACCACGGGAGGAATACCAACCCAGCGGTCATATTAACGTATCCAGAGCAAGAGAATTTTATATTAGCTGGGACACAGATTATGTGGGGTCTATCACCACGGCTGATCTTGTGGTATCGGCATCCGCTATTAACTT | HM745321 | [Gallardo et al 2011](http://asf-referencelab.info/asf/images/files/publicaciones/Nix_et_al_2006.pdf) | AGTGCGTATACCTGTGCAAGCACTTGTGCAAGCACTTGTGCAAGCACTTGTGCAGACACCAATGTAGACACTTGTGCAAGCACTTGTGCAGACATTTGTGCAGACACCAATGTAGACACTTGTGCAAGCACTTGTGCAGATACTTGTGCAGACACTTGTGTAAGCACTTGTGTAAGCACTTGTGCAGACACCAATGTAGACACTTGTGCAAGCACTTGTGCAGACACCAATGTAGACACTTGTGTAAGCACTTGTGCAGACACCTGTGCAAGCACAGAATAC |
| 208 | Ken08WH/8 | Kenya | Wild pig | 2008 | HM745287 | IX | [Gallardo et al 2011](http://asf-referencelab.info/asf/images/files/publicaciones/Gallardo-et-al-2009a.pdf) | ATGCAGCCTACCCACCACGCAGAGGTAAGCTTTCAGGATAGAGATACAGCTCTTCCAGATGCATGTTCATCCATATCTGATATTACCCCCATTACTTATCCGATCACATTACCTATTATTAAAAACATTTCCGTTACTGCTCACGGTATCAATCTTATCGATAAATTTCCATCAAAGTTCTGCAGCTCTTACATACCCTTCCACTACGGAGGCAATTCGATTAAAACCCCCGACGATCCGGGCGCGATGATGATTACCTTTGCTTTGAAACCACGGGAGGAATACCAACCCAGCGGTCATATTAACGTATCCAGAGCAAGAGAATTTTATATTAGCTGGGACACAGATTATGTGGGGTCTATCACCACGGCTGATCTTGTGGTATCGGCATCCGCTATTAACTT | HM745322 | [Gallardo et al 2011](http://asf-referencelab.info/asf/images/files/publicaciones/Nix_et_al_2006.pdf) | AGTGCGTATACCTGTGCAAGCACTTGTGCAAGCACTTGTGCAAGCACTTGTGCAGACACCAATGTAGACACTTGTGCAAGCACTTGTGCAGACATTTGTGCAGACACCAATGTAGACACTTGTGCAAGCACTTGTGCAGATACTTGTGCAGACACTTGTGTAAGCACTTGTGTAAGCACTTGTGCAGACACCAATGTAGACACTTGTGCAAGCACTTGTGCAGACACCAATGTAGACACTTGTGTAAGCACTTGTGCAGACACCTGTGCAAGCACAGAATAC |
| 209 | GH08/BASu17 | Ghana | DP | 2008 |  | I | [This study (gallardo@inia.es)](http://asf-referencelab.info/asf/images/files/publicaciones/Gallardo-et-al-2009a.pdf) | ATGCAGCCTACTCACCACGCAGAGATAAGCTTTCAGGATAGAGATACAGCTCTTCCAGACGCATGTTCATCTATATCGGATATTAGCCCCGTTACGTATCCGATCACATTACCTATTATTAAAAACATTTCCGTAACTGCTCATGGTATCAATCTTATCGATAAGTTTCCATCAAAGTTCTGCAGCTCTTACATACCCTTCCACTACGGAGGCAATGCAATTAAAACCCCCGATGATCCGGGTGCGATGATGATTACCTTTGCTTTGAAGCCACGGGAGGAATACCAACCCAGTGGTCATATTAACGTATCCAGAGCAAGAGAATTTTATATTAGTTGGGACACGGATTACGTGGGGTCTATCACTACGGCTGATCTTGTGGTATCGGCATCTGCTATTAACTT |  | [This study (gallardo@inia.es)](http://asf-referencelab.info/asf/images/files/publicaciones/Nix_et_al_2006.pdf) | AGTGCGTATACTTGTGCAAGCACTTGTGCAGATACCAATGTAGACACCTGTGCAAGCACTTGTGCAAGCACTTGTGCAAGCACTTGTGCAAGCACAGGTGCAAGCACTTGTGCAGATACCAATGTAGACACCTGTGCAAGCACTTGTGCAAGCACTTGTGCAAGCACTTGTGCAAGCACTTGTGCAAGCACTTGTGCAAGCACTTGTGCAAGCACTTGTGCAAGCACAGGTGCAAGCACTTGTGCAGATACCAATGTAGACACCTGTGCAAGCACCTGTGCAAACACCTGTGCAAGCACAGAATAC |
| 210 | GH08/GADg2 | Ghana | DP | 2008 |  | I | [This study (gallardo@inia.es)](http://asf-referencelab.info/asf/images/files/publicaciones/Gallardo-et-al-2009a.pdf) | ATGCAGCCTACTCACCACGCAGAGATAAGCTTTCAGGATAGAGATACAGCTCTTCCAGACGCATGTTCATCTATATCGGATATTAGCCCCGTTACGTATCCGATCACATTACCTATTATTAAAAACATTTCCGTAACTGCTCATGGTATCAATCTTATCGATAAGTTTCCATCAAAGTTCTGCAGCTCTTACATACCCTTCCACTACGGAGGCAATGCAATTAAAACCCCCGATGATCCGGGTGCGATGATGATTACCTTTGCTTTGAAGCCACGGGAGGAATACCAACCCAGTGGTCATATTAACGTATCCAGAGCAAGAGAATTTTATATTAGTTGGGACACGGATTACGTGGGGTCTATCACTACGGCTGATCTTGTGGTATCGGCATCTGCTATTAACTT |  | [This study (gallardo@inia.es)](http://asf-referencelab.info/asf/images/files/publicaciones/Nix_et_al_2006.pdf) | AGTGCGTATACTTGTGCAAGCACTTGTGCAGATACCAATGTAGACACCTGTGCAAGCACTTGTGCAAGCACTTGTGCAAGCACTTGTGCAAGCACAGGTGCAAGCACTTGTGCAGATACCAATGTAGACACCTGTGCAAGCACTTGTGCAAGCACTTGTGCAAGCACTTGTGCAAGCACAGGTGCAAGCACTTGTGCAGATACCAATGTAGACACCTGTGCAAGCACTTGTGCAAGCACTTGTGCAAGCACTTGTGCAAGCACTTGTGCAAGCACTTGTGCAAGCACTTGTGCAAGCACTTGTGCAAGCACTTGTGCAAGCACAGGTGCAAGCACTTGTGCAGATACCAATGTAGACACCTGTGCAAGCACCTGTGCAAACACCTGTGCAAGCACAGAATAC |
| 211 | Ben09/Z8 | Benin | DP | 2009 |  | I | [This study (gallardo@inia.es)](http://asf-referencelab.info/asf/images/files/publicaciones/Gallardo-et-al-2009a.pdf) | ATGCAGCCTACTCACCACGCAGAGATAAGCTTTCAGGATAGAGATACAGCTCTTCCAGACGCATGTTCATCTATATCGGATATTAGCCCCGTTACGTATCCGATCACATTACCTATTATTAAAAACATTTCCGTAACTGCTCATGGTATCAATCTTATCGATAAGTTTCCATCAAAGTTCTGCAGCTCTTACATACCCTTCCACTACGGAGGCAATGCAATTAAAACCCCCGATGATCCGGGTGCGATGATGATTACCTTTGCTTTGAAGCCACGGGAGGAATACCAACCCAGTGGTCATATTAACGTATCCAGAGCAAGAGAATTTTATATTAGTTGGGACACGGATTACGTGGGGTCTATCACTACGGCTGATCTTGTGGTATCGGCATCTGCTATTAACTT |  | [This study (gallardo@inia.es)](http://asf-referencelab.info/asf/images/files/publicaciones/Nix_et_al_2006.pdf) | AGTGCGTATACCTGTGCAAGCACTTGTGCAGATACCAATGTAGACACCTGTGCAAGCACTTGTGCAAGCACTTGTGCAAGCACTTGTGCAAGCACTTGTGCAAGCACTTGTGCAAGCACTTGTGCAAGCACAGGTGCAAGCACTTGTGCAGATACCAATGTAGACACCTGTGCAAGCACTTGTGCAAGCACTTGTGCAAGCACTTGTGCAAGCACTTGTGCAAGCACAGGTGCAAGCACTTGTGCAGATACCAATGTAGACACCTGTGCAAGCACTTGTGCAAGCACTTGTGCAAGCACTTGTGCAAGCACTTGTGCAAGCACAGGTGCAAGCACTTGTGCAGATACCAATGTAGACACCTGTGCAAGCACCTGTGCAAACACCTGTGCAAGCACAGAATAC |
| 212 | BF09/BAG1 | Burkina Faso | DP | 2009 |  | I | [This study (gallardo@inia.es)](http://asf-referencelab.info/asf/images/files/publicaciones/Gallardo-et-al-2009a.pdf) | ATGCAGCCTACTCACCACGCAGAGATAAGCTTTCAGGATAGAGATACAGCTCTTCCAGACGCATGTTCATCTATATCGGATATTAGCCCCGTTACGTATCCGATCACATTACCTATTATTAAAAACATTTCCGTAACTGCTCATGGTATCAATCTTATCGATAAGTTTCCATCAAAGTTCTGCAGCTCTTACATACCCTTCCACTACGGAGGCAATGCAATTAAAACCCCCGATGATCCGGGTGCGATGATGATTACCTTTGCTTTGAAGCCACGGGAGGAATACCAACCCAGTGGTCATATTAACGTATCCAGAGCAAGAGAATTTTATATTAGTTGGGACACGGATTACGTGGGGTCTATCACTACGGCTGATCTTGTGGTATCGGCATCTGCTATTAACTT |  | [This study (gallardo@inia.es)](http://asf-referencelab.info/asf/images/files/publicaciones/Nix_et_al_2006.pdf) | AGTGCGTATACTTGTGCAAGCACTTGTGCAGATACCAATGTAGACACCTGTGCAAGCACTTGTGCAAGCACTTGTGCAAGCACTTGTGCAAGCACAGGTGCAAGCACTTGTGCAGATACCAATGTAGACACCTGTGCAAGCACTTGTGCAAGCACTTGTGCAAGCACTTGTGCAAGCACTTGTGCAAGCACTTGTGCAAGCACTTGTGCAAGCACTTGTGCAAGCACAGGTGCAAGCACTTGTGCAGATACCAATGTAGACACCTGTGCAAGCACCTGTGCAAACACCTGTGCAAGCACAGAATAC |
| 213 | BF09/K1 | Burkina Faso | DP | 2009 |  | I | [This study (gallardo@inia.es)](http://asf-referencelab.info/asf/images/files/publicaciones/Gallardo-et-al-2009a.pdf) | ATGCAGCCTACTCACCACGCAGAGATAAGCTTTCAGGATAGAGATACAGCTCTTCCAGACGCATGTTCATCTATATCGGATATTAGCCCCGTTACGTATCCGATCACATTACCTATTATTAAAAACATTTCCGTAACTGCTCATGGTATCAATCTTATCGATAAGTTTCCATCAAAGTTCTGCAGCTCTTACATACCCTTCCACTACGGAGGCAATGCAATTAAAACCCCCGATGATCCGGGTGCGATGATGATTACCTTTGCTTTGAAGCCACGGGAGGAATACCAACCCAGTGGTCATATTAACGTATCCAGAGCAAGAGAATTTTATATTAGTTGGGACACGGATTACGTGGGGTCTATCACTACGGCTGATCTTGTGGTATCGGCATCTGCTATTAACTT |  | [This study (gallardo@inia.es)](http://asf-referencelab.info/asf/images/files/publicaciones/Nix_et_al_2006.pdf) | AGTGCGTATACTTGTGCAAGCACTTGTGCAGATACCAATGTAGACACCTGTGCAAGCACTTGTGCAAGCACTTGTGCAAGCACTTGTGCAAGCACAGGTGCAAGCACTTGTGCAAGCACTTGTGCAAGCACTTGTGCAAGCACAGGTGCAAGCACTTGTGCAAGCACTTGTGCAGATACCAATGTAGACACCTGTGCAAGCACTTGTGCAAGCACTTGTGCAGATACCAATGTAGACACCTGTGCAAGCACTTGTGCAAGCACTTGTGCAAGCACTTGTGCAAGCACTTGTGCAAGCACTTGTGCAAGCACAGGTGCAAGCACTTGTGCAGATACCAATGTAGACACCTGTGCAAGCACTTGTGCAAGCACTTGTGCAAGCACTTGTGCAAGCACTTGTGCAAGCACTTGTGCAAGCACAGGTGCAAGCACTTGTGCAGATACCAATGTAGACACCTGTGCAAGCACTTGTGCAAGCACTTGTGCAAGCACTTGTGCAAGCACTTGTGCAAGCACTTGTGCAAGCACAGGTGCAAGCACTTGTGCAGATACCAATGTAGACACCTGTGCAAGCACCTGTGCAAACACCTGTGCAAGCACAGAATAC |
| 214 | BF09/S1 | Burkina Faso | DP | 2009 |  | I | [This study (gallardo@inia.es)](http://asf-referencelab.info/asf/images/files/publicaciones/Gallardo-et-al-2009a.pdf) | ATGCAGCCTACTCACCACGCAGAGATAAGCTTTCAGGATAGAGATACAGCTCTTCCAGACGCATGTTCATCTATATCGGATATTAGCCCCGTTACGTATCCGATCACATTACCTATTATTAAAAACATTTCCGTAACTGCTCATGGTATCAATCTTATCGATAAGTTTCCATCAAAGTTCTGCAGCTCTTACATACCCTTCCACTACGGAGGCAATGCAATTAAAACCCCCGATGATCCGGGTGCGATGATGATTACCTTTGCTTTGAAGCCACGGGAGGAATACCAACCCAGTGGTCATATTAACGTATCCAGAGCAAGAGAATTTTATATTAGTTGGGACACGGATTACGTGGGGTCTATCACTACGGCTGATCTTGTGGTATCGGCATCTGCTATTAACTT |  | [This study (gallardo@inia.es)](http://asf-referencelab.info/asf/images/files/publicaciones/Nix_et_al_2006.pdf) | AGTGCGTATACTTGTGCAAGCACTTGTGCAGATACCAATGTAGACACCTGTGCAAGCACTTGTGCAAGCACTTGTGCAAGCACTTGTGCAAGCACAGGTGCAAGCACTTGTGCAGATACCAATGTAGACACCTGTGCAAGCACTTGTGCAAGCACTTGTGCAAGCACTTGTGCAAGCACTTGTGCAAGCACTTGTGCAAGCACAGGTGCAAGCACTTGTGCAGATACCAATGTAGACACCTGTGCAAGCACTTGTGCAAGCACTTGTGCAAGCACTTGTGCAAGCACTTGTGCAAGCACTTGTGCAAGCACAGGTGCAAGCACTTGTGCAGATACCAATGTAGACACCTGTGCAAGCACTTGTGCAAGCACTTGTGCAAGCACTTGTGCAAGCACTTGTGCAAGCACTTGTGCAAGCACAGGTGCAAGCACTTGTGCAGATACCAATGTAGACACCTGTGCAAGCACTTGTGCAAGCACTTGTGCAAGCACTTGTGCAAGCACTTGTGCAAGCACTTGTGCAAGCACAGGTGCAAGCACTTGTGCAGATACCAATGTAGACACCTGTGCAAGCACCTGTGCAAACACCTGTGCAAGCACAGAATAC |
| 215 | Ken09Tk.13/1 | Kenya | Tick | 2009 | HM745277 | X | [Gallardo et al 2011](http://asf-referencelab.info/asf/images/files/publicaciones/Gallardo-et-al-2009a.pdf) | ATGCAGCCTACCCACCACGCAGAGGTAAGCTTTCAGGATAGAGATACAGCTCTTCCAGATGCATGTTCATCCATATCAGATATTACCCCCATTACTTATCCGATCACGTTACCTATTATTAAAAACATTTCCGTCACTGCTCATGGTATCAATCTTATCGATAAATTTCCATCAAAGTTCTGCAGCTCTTACATACCCTTTCACTACGGAGGCAATTCGATTAAAACCCCCGACGATCCGGGCGCGATGATGATTACCTTTGCTTTGAAACCACGGGAGGAATACCAACCCAGTGGTCATATTAACGTATCCAGAGCAAGAGAATTTTATATTAGCTGGGACACAGATTATGTGGGGTCTATCACCACGGCCGATCTTGTGGTATCGGCATCCGCTATTAACTT | HM745312 | [Gallardo et al 2011](http://asf-referencelab.info/asf/images/files/publicaciones/Nix_et_al_2006.pdf) | AGTGCGTATACCTGTGCAAGCACCTGTGCAAGCACCTGTGCAGACACCAATGTAGACACTTGTGCAAGCACTTGTGTAAGCACCTGTGCAGACACTTGTGCAGACACCTGTGCAAGCACAGAATAC |
| 216 | Ken09Tk.13/2 | Kenya | Tick | 2009 | HM745278 | X | [Gallardo et al 2011](http://asf-referencelab.info/asf/images/files/publicaciones/Gallardo-et-al-2009a.pdf) | ATGCAGCCTACCCACCACGCAGAGGTAAGCTTTCAGGATAGAGATACAGCTCTTCCAGATGCATGTTCATCCATATCAGATATTACCCCCATTACTTATCCGATCACGTTACCTATTATTAAAAACATTTCCGTCACTGCTCATGGTATCAATCTTATCGATAAATTTCCATCAAAGTTCTGCAGCTCTTACATACCCTTTCACTACGGAGGCAATTCGATTAAAACCCCCGACGATCCGGGCGCGATGATGATTACCTTTGCTTTGAAACCACGGGAGGAATACCAACCCAGCGGTCATATTAACGTATCCAGAGCAAGAGAATTTTATATTAGCTGGGACACAGATTATGTGGGGTCTATCACCACGGCCGATCTTGTGGTATCGGCATCCGCTATTAACTT | HM745313 | [Gallardo et al 2011](http://asf-referencelab.info/asf/images/files/publicaciones/Nix_et_al_2006.pdf) | AGTGCGTATACCTGTGCAAGCACCTGTGCAAGCACCTGTGCAGACACCAATGTAGACACTTGTGCAAGCACTTGTGTAAGCACCTGTGCAGACACTTGTGCAGACACCTGTGCAAGCACAGAATAC |
| 217 | Ken09Tk.15/4 | Kenya | Tick | 2009 | HM745279 | X | [Gallardo et al 2011](http://asf-referencelab.info/asf/images/files/publicaciones/Gallardo-et-al-2009a.pdf) | ATGCAGCCTACCCACCACGCAGAGGTAAGCTTTCAGGATAGAGATACAGCTCTTCCAGATGCATGTTCATCCATATCAGATATTACCCCCATTACTTATCCGATCACGTTACCTATTATTAAAAACATTTCCGTCACTGCTCATGGTATCAATCTTATCGATAAATTTCCATCAAAGTTCTGCAGCTCTTACATACCCTTTCACTACGGAGGCAATTCGATTAAAACCCCCGACGATCCGGGCGCGATGATGATTACCTTTGCTTTGAAACCACGGGAGGAATACCAACCCAGCGGTCATATTAACGTATCCAGAGCAAGAGAATTTTATATTAGCTGGGACACAGATTATGTGGGGTCTATCACCACGGCCGATCTTGTGGTATCGGCATCCGCTATTAACTT | HM745314 | [Gallardo et al 2011](http://asf-referencelab.info/asf/images/files/publicaciones/Nix_et_al_2006.pdf) | AGTGCGTATACCTGTGCAAGCACCTGTGCAAGCACCTGTGCAGACACCAATGTAGACACTTGTGCAAGCACTTGTGTAAGCACCTGTGCAGACACTTGTGCAGACACCTGTGCAAGCACAGAATAC |
| 218 | Ken09Tk.15/6 | Kenya | Tick | 2009 | HM745280 | X | [Gallardo et al 2011](http://asf-referencelab.info/asf/images/files/publicaciones/Gallardo-et-al-2009a.pdf) | ATGCAGCCTACCCACCACGCAGAGGTAAGCTTTCAGGATAGAGATACAGCTCTTCCAGATGCATGTTCATCCATATCAGATATTACCCCCATTACTTATCCGATCACGTTACCTATTATTAAAAACATTTCCGTCACTGCTCATGGTATCAATCTTATCGATAAATTTCCATCAAAGTTCTGCAGCTCTTACATACCCTTTCACTACGGAGGCAATTCGATTAAAACCCCCGACGATCCGGGCGCGATGATGATTACCTTTGCTTTGAAACCACGGGAGGAATACCAACCCAGTGGTCATATTAACGTATCCAGAGCAAGAGAATTTTATATTAGCTGGGACACAGATTATGTGGGGTCTATCACCACGGCCGATCTTGTGGTATCGGCATCCGCTATTAACTT | HM745315 | [Gallardo et al 2011](http://asf-referencelab.info/asf/images/files/publicaciones/Nix_et_al_2006.pdf) | AGTGCGTATACCTGTGCAAGCACCTGTGCAAGCACCTGTGCAGACACCAATGTAGACACTTGTGCAAGCACTTGTGTAAGCACCTGTGCAGACACTTGTGCAGACACCTGTGCAAGCACAGAATAC |
| 219 | Ken09Tk.19/2 | Kenya | Tick | 2009 | HM745281 | X | [Gallardo et al 2011](http://asf-referencelab.info/asf/images/files/publicaciones/Gallardo-et-al-2009a.pdf) | ATGCAGCCTACCCACCACGCAGAGGTAAGCTTTCAGGATAGAGATACAGCTCTTCCAGATGCATGTTCATCCATATCAGATATTACCCCCATTACTTATCCGATCACGTTACCTATTATTAAAAACATTTCCGTCACTGCTCATGGTATCAATCTTATCGATAAATTTCCATCAAAGTTCTGCAGCTCTTACATACCCTTTCACTACGGAGGCAATTCGATTAAAACCCCCGACGATCCGGGCGCGATGATGATTACCTTTGCTTTGAAACCACGGGAGGAATACCAACCCAGCGGTCATATTAACGTATCCAGAGCAAGAGAATTTTATATTAGCTGGGACACAGATTATGTGGGGTCTATCACCACGGCCGATCTTGTGGTATCGGCATCCGCTATTAACTT | HM745316 | [Gallardo et al 2011](http://asf-referencelab.info/asf/images/files/publicaciones/Nix_et_al_2006.pdf) | AGTGCGTATACCTGTGCAAGCACCTGTGCAAGCACCTGTGCAGACACCAATGTAGACACTTGTGCAAGCACTTGTGTAAGCACCTGTGCAGACACTTGTGCAGACACCTGTGCAAGCACAGAATAC |
| 220 | Ken09Tk.19/7 | Kenya | Tick | 2009 | HM745282 | X | [Gallardo et al 2011](http://asf-referencelab.info/asf/images/files/publicaciones/Gallardo-et-al-2009a.pdf) | ATGCAGCCTACCCACCACGCAGAGGTAAGCTTTCAGGATAGAGATACAGCTCTTCCAGATGCATGTTCATCCATATCAGATATTACCCCCATTACTTATCCGATCACGTTACCTATTATTAAAAACATTTCCGTCACTGCTCATGGTATCAATCTTATCGATAAATTTCCATCAAAGTTCTGCAGCTCTTACATACCCTTTCACTACGGAGGCAATTCGATTAAAACCCCCGACGATCCGGGCGCGATGATGATTACCTTTGCTTTGAAACCACGGGAGGAATACCAACCCAGCGGTCATATTAACGTATCCAGAGCAAGAGAATTTTATATTAGCTGGGACACAGATTATGTGGGGTCTATCACCACGGCCGATCTTGTGGTATCGGCATCCGCTATTAACTT | HM745317 | [Gallardo et al 2011](http://asf-referencelab.info/asf/images/files/publicaciones/Nix_et_al_2006.pdf) | AGTGCGTATACCTGTGCAAGCACCTGTGCAAGCACCTGTGCAGACACCAATGTAGACACTTGTGCAAGCACTTGTGTAAGCACCTGTGCAGACACTTGTGCAGACACCTGTGCAAGCACAGAATAC |
| 221 | Ken09Tk.20/5 | Kenya | Tick | 2009 | HM745284 | X | [Gallardo et al 2011](http://asf-referencelab.info/asf/images/files/publicaciones/Gallardo-et-al-2009a.pdf) | ATGCAGCCTACCCACCACGCAGAGGTAAGCTTTCAGGATAGAGATACAGCTCTTCCAGATGCATGTTCATCCATATCAGATATTACCCCCATTACTTATCCGATCACGTTACCTATTATTAAAAACATTTCCGTCACTGCTCATGGTATCAATCTTATCGATAAATTTCCATCAAAGTTCTGCAGCTCTTACATACCCTTTCACTACGGAGGCAATTCGATTAAAACCCCCGACGATCCGGGCGCGATGATGATTACCTTTGCTTTGAAACCACGGGAGGAATACCAACCCAGCGGTCATATTAACGTATCCAGAGCAAGAGAATTTTATATTAGCTGGGACACAGATTATGTGGGGTCTATCACCACGGCCGATCTTGTGGTATCGGCATCCGCTATTAACTT | HM745319 | [Gallardo et al 2011](http://asf-referencelab.info/asf/images/files/publicaciones/Nix_et_al_2006.pdf) | AGTGCGTATACCTGTGCAAGCACCTGTGCAAGCACCTGTGCAGACACCAATGTAGACACTTGTGCAAGCACCTGTGCAGACACTTGTGCAGACACCTGTACAAGCACAGAATAC |
| 222 | Ken09Tk.19/11 | Kenya | Tick | 2009 | HM745283 | X | [Gallardo et al 2011](http://asf-referencelab.info/asf/images/files/publicaciones/Gallardo-et-al-2009a.pdf) | ATGCAGCCTACCCACCACGCAGAGGTAAGCTTTCAGGATAGAGATACAGCTCTTCCAGATGCATGTTCATCCATATCAGATATTACCCCCATTACTTATCCGATCACGTTACCTATTATTAAAAACATTTCCGTCACTGCTCATGGTATCAATCTTATCGATAAATTTCCATCAAAGTTCTGCAGCTCTTACATACCCTTTCACTACGGAGGCAATTCGATTAAAACCCCCGACGATCCGGGCGCGATGATGATTACCTTTGCTTTGAAACCACGGGAGGAATACCAACCCAGCGGTCATATTAACGTATCCAGAGCAAGAGAATTTTATATTAGCTGGGACACAGATTATGTGGGGTCTATCACCACGGCCGATCTTGTGGTATCGGCATCCGCTATTAACTT | HM745318 | [Gallardo et al 2011](http://asf-referencelab.info/asf/images/files/publicaciones/Nix_et_al_2006.pdf) | AGTGCGTATACCTGTGCAAGCACCTGTGCAAGCACCTGTGCAGACACCAATGTAGACACTTGTGCAAGCACTTGTGTAAGCACCTGTGCAGACACTTGTGCAGACACCTGTGCAAGCACAGAATAC |
| 223 | Ben09/AGL1 | Benin | DP | 2009 |  | I | [This study (gallardo@inia.es)](http://asf-referencelab.info/asf/images/files/publicaciones/Gallardo-et-al-2009a.pdf) | ATGCAGCCTACTCACCACGCAGAGATAAGCTTTCAGGATAGAGATACAGCTCTTCCAGACGCATGTTCATCTATATCGGATATTAGCCCCGTTACGTATCCGATCACATTACCTATTATTAAAAACATTTCCGTAACTGCTCATGGTATCAATCTTATCGATAAGTTTCCATCAAAGTTCTGCAGCTCTTACATACCCTTCCACTACGGAGGCAATGCAATTAAAACCCCCGATGATCCGGGTGCGATGATGATTACCTTTGCTTTGAAGCCACGGGAGGAATACCAACCCAGTGGTCATATTAACGTATCCAGAGCAAGAGAATTTTATATTAGTTGGGACACGGATTACGTGGGGTCTATCACTACGGCTGATCTTGTGGTATCGGCATCTGCTATTAACTT |  | [This study (gallardo@inia.es)](http://asf-referencelab.info/asf/images/files/publicaciones/Gianmaroli_et_al_2011.pdf) | AGTGCGTATACTTGTGCAAGCACTTGTGCAGATACCAATGTAGACACCTGTGCAAGCACTTGTGCAAGCACTTGTGCAAGCACTTGTGCAAGCACTTGTGCAAGCACAGGTGCAAGCACTTGTGCAGATACCAATGTAGACACCTGTGCAAGCACTTGTGCAAGCACTTGTGCAAGCACTTGTGCAAGCACTTGTGCAAGCACAGGTGCAAGCACTTGTGCAGATACCAATGTAGACACCTGTGCAAGCACCTGTGCAAACACCTGTGCAAGCACAGAATAC |
| 224 | Ben09/Ak3 | Benin | DP | 2009 |  | I | [This study (gallardo@inia.es)](http://asf-referencelab.info/asf/images/files/publicaciones/Gallardo-et-al-2009a.pdf) | ATGCAGCCTACTCACCACGCAGAGATAAGCTTTCAGGATAGAGATACAGCTCTTCCAGACGCATGTTCATCTATATCGGATATTAGCCCCGTTACGTATCCGATCACATTACCTATTATTAAAAACATTTCCGTAACTGCTCATGGTATCAATCTTATCGATAAGTTTCCATCAAAGTTCTGCAGCTCTTACATACCCTTCCACTACGGAGGCAATGCAATTAAAACCCCCGATGATCCGGGTGCGATGATGATTACCTTTGCTTTGAAGCCACGGGAGGAATACCAACCCAGTGGTCATATTAACGTATCCAGAGCAAGAGAATTTTATATTAGTTGGGACACGGATTACGTGGGGTCTATCACTACGGCTGATCTTGTGGTATCGGCATCTGCTATTAACTT |  | [This study (gallardo@inia.es)](http://asf-referencelab.info/asf/images/files/publicaciones/Gianmaroli_et_al_2011.pdf) | AGTGCGTATACTTGTGCAAGCACTTGTGCAGATACCAATGTAGACACCTGTGCAAGCACTTGTGCAAGCACTTGTGCAAGCACTTGTGCAAGCACTTGTGCAAGCACAGGTGCAAGCACTTGTGCAGATACCAATGTAGACACCTGTGCAAGCACTTGTGCAAGCACTTGTGCAAGCACTTGTGCAAGCACTTGTGCAAGCACAGGTGCAAGCACTTGTGCAGATACCAATGTAGACACCTGTGCAAGCACTTGTGCAAACACTTGTGCAAGCACTTGTGCAAGCACAGGTGCAAGCACTTGTGCAGATACCAATGTAGACACCTGTGCAAGCACCTGTGCAAACACCTGTGCAAGCACAGAATAC |
| 225 | 51/Nu/09 | Italy | DP | 2009 | FR668419 | I | [Giammarioli et al 2011](http://asf-referencelab.info/asf/images/files/publicaciones/Gallardo-et-al-2009a.pdf) | ATGCAGCCTACTCACCACGCAGAGATAAGCTTTCAGGATAGAGATACAGCTCTTCCAGACGCATGTTCATCTATATCGGATATTAGCCCCGTTACGTATCCGATCACATTACCTATTATTAAAAACATTTCCGTAACTGCTCATGGTATCAATCTTATCGATAAGTTTCCATCAAAGTTCTGCAGCTCTTACATACCCTTCCACTACGGAGGCAATGCAATTAAAACCCCCGATGATCCGGGTGCGATGATGATTACCTTTGCTTTGAAGCCACGGGAGGAATACCAACCCAGTGGTCATATTAACGTATCCAGAGCAAGAGAATTTTATATTAGTTGGGACACGGATTACGTGGGGTCTATCACTACGGCTGATCTTGTGGTATCGGCATCTGCTATTAACTT | FR686545 | [Giammarioli et al 2011](http://asf-referencelab.info/asf/images/files/publicaciones/Gianmaroli_et_al_2011.pdf) | AGTGCGTATACCTGTGCAAGCACTTGCGCAGATACCAATGTGGACACCTGTGCAAGCACTTGTGCAAGCACTTGTGCAAGCACTTGTGCAAGCATGTGTGCAGATACCAATGTAGACACCTGTGCAAGCACCTGTGCAAACACCTGTGCAAGCACAGAATAC |
| 226 | 4/Ol/02 | Italy | DP | 2009 | FR668401 | I | [Giammarioli et al 2011](http://asf-referencelab.info/asf/images/files/publicaciones/Gallardo-et-al-2009a.pdf) | ATGCAGCCTACTCACCACGCAGAGATAAGCTTTCAGGATAGAGATACAGCTCTTCCAGACGCATGTTCATCTATATCGGATATTAGCCCCGTTACGTATCCGATCACATTACCTATTATTAAAAACATTTCCGTAACTGCTCATGGTATCAATCTTATCGATAAGTTTCCATCAAAGTTCTGCAGCTCTTACATACCCTTCCACTACGGAGGCAATGCAATTAAAACCCCCGATGATCCGGGTGCGATGATGATTACCTTTGCTTTGAAGCCACGGGAGGAATACCAACCCAGTGGTCATATTAACGTATCCAGAGCAAGAGAATTTTATATTAGTTGGGACACGGATTACGTGGGGTCTATCACTACGGCTGATCTTGTGGTATCGGCATCTGCTATTAACTT | FR686538 | [Giammarioli et al 2011](http://asf-referencelab.info/asf/images/files/publicaciones/Gianmaroli_et_al_2011.pdf) | AGTGCGTATACCTGTGCGAGCACTTGTGCAGATACCAATGTAGACACCTGTGCCAGCACTTGTGCAAGCACTTGTGCAAGCACTTGTGCAAGCATGTGTGCAGATACCAATGTAGACACCTGCGCAAGCACCTGTGCAAACACCTGTGCAAGCACAGAATAC |
| 227 | 5/Ca/02 | Italy | DP | 2009 | FR668402 | I | [Giammarioli et al 2011](http://asf-referencelab.info/asf/images/files/publicaciones/Gallardo-et-al-2009a.pdf) | ATGCAGCCTACTCACCACGCAGAGATAAGCTTTCAGGATAGAGATACAGCTCTTCCAGACGCATGTTCATCTATATCGGATATTAGCCCCGTTACGTATCCGATCACATTACCTATTATTAAAAACATTTCCGTAACTGCTCATGGTATCAATCTTATCGATAAGTTTCCATCAAAGTTCTGCAGCTCTTACATACCCTTCCACTACGGAGGCAATGCAATTAAAACCCCCGATGATCCGGGTGCGATGATGATTACCTTTGCTTTGAAGCCACGGGAGGAATACCAACCCAGTGGTCATATTAACGTATCCAGAGCAAGAGAATTTTATATTAGTTGGGACACGGATTACGTGGGGTCTATCACTACGGCTGATCTTGTGGTATCGGCATCTGCTATTAACTT | FR686539 | [Giammarioli et al 2011](http://asf-referencelab.info/asf/images/files/publicaciones/Gianmaroli_et_al_2011.pdf) | AGTGCGTATACCTGTGCGAGCACTTGTGCAGATACCAATGTAGACACCTGTGCAAGCACTTGTGCAAGCACTTGTGCAAGCACTTGTGCAAGCATGTGTGCAGATACCAATGTAGACACCTGCGCAAGCACCTGTGCAAACACCTGTGCAAGCACAGAATAC |
| 228 | BF09/AO2 | Burkina Faso | DP | 2009 |  | I | [This study (gallardo@inia.es)](http://asf-referencelab.info/asf/images/files/publicaciones/Gianmaroli_et_al_2011.pdf) | ATGCAGCCTACTCACCACGCAGAGATAAGCTTTCAGGATAGAGATACAGCTCTTCCAGACGCATGTTCATCTATATCGGATATTAGCCCCGTTACGTATCCGATCACATTACCTATTATTAAAAACATTTCCGTAACTGCTCATGGTATCAATCTTATCGATAAGTTTCCATCAAAGTTCTGCAGCTCTTACATACCCTTCCACTACGGAGGCAATGCAATTAAAACCCCCGATGATCCGGGTGCGATGATGATTACCTTTGCTTTGAAGCCACGGGAGGAATACCAACCCAGTGGTCATATTAACGTATCCAGAGCAAGAGAATTTTATATTAGTTGGGACACGGATTACGTGGGGTCTATCACTACGGCTGATCTTGTGGTATCGGCATCTGCTATTAACTT |  | [This study (gallardo@inia.es)](http://asf-referencelab.info/asf/images/files/publicaciones/Gianmaroli_et_al_2011.pdf) | AGTGCGTATACTTGTGCAAGCACTTGTGCAGATACCAATGTAGACACCTGTGCAAGCACTTGTGCAAGCACTTGTGCAAGCACTTGTGCAAGCACAGGTGCAAGCACTTGTGCAGATACCAATGTAGACACCTGTGCAAGCACTTGTGCAAGCACTTGTGCAAGCACTTGTGCAAGCACTTGTGCAAGCACTTGTGCAAGCACAGGTGCAAGCACTTGTGCAGATACCAATGTAGACACCTGTGCAAGCACTTGTGCAAGCACTTGTGCAAGCACTTGTGCAAGCACTTGTGCAAGCACTTGTGCAAGCACAGGTGCAAGCACTTGTGCAGATACCAATGTAGACACCTGTGCAAGCACTTGTGCAAGCACTTGTGCAAGCACTTGTGCAAGCACTTGTGCAAGCACTTGTGCAAGCACAGGTGCAAGCACTTGTGCAGATACCAATGTAGACACCTGTGCAAGCACTTGTGCAAGCACTTGTGCAAGCACTTGTGCAAGCACTTGTGCAAGCACTTGTGCAAGCACAGGTGCAAGCACTTGTGCAGATACCAATGTAGACACCTGTGCAAGCACCTGTGCAAACACCTGTGCAAGCACAGAATAC |
| 229 | BF09/DO1 | Burkina Faso | DP | 2009 |  | I | [This study (gallardo@inia.es)](http://asf-referencelab.info/asf/images/files/publicaciones/Gianmaroli_et_al_2011.pdf) | ATGCAGCCTACTCACCACGCAGAGATAAGCTTTCAGGATAGAGATACAGCTCTTCCAGACGCATGTTCATCTATATCGGATATTAGCCCCGTTACGTATCCGATCACATTACCTATTATTAAAAACATTTCCGTAACTGCTCATGGTATCAATCTTATCGATAAGTTTCCATCAAAGTTCTGCAGCTCTTACATACCCTTCCACTACGGAGGCAATGCAATTAAAACCCCCGATGATCCGGGTGCGATGATGATTACCTTTGCTTTGAAGCCACGGGAGGAATACCAACCCAGTGGTCATATTAACGTATCCAGAGCAAGAGAATTTTATATTAGTTGGGACACGGATTACGTGGGGTCTATCACTACGGCTGATCTTGTGGTATCGGCATCTGCTATTAACTT |  | [This study (gallardo@inia.es)](http://asf-referencelab.info/asf/images/files/publicaciones/Gianmaroli_et_al_2011.pdf) | AGTGCGTATACTTGTGCAAGCACTTGTGCAGATACCAATGTAGACACCTGTGCAAGCACTTGTGCAAGCACTTGTGCAAGCACTTGTGCAAGCACAGGTGCAAGCACTTGTGCAGATACCAATGTAGACACCTGTGCAAGCACTTGTGCAAGCACTTGTGCAAGCACTTGTGCAAGCACTTGTGCAAGCACTTGTGCAAGCACTTGTGCAAGCACTTGTGCAAGCACTTGTGCAAGCACTTGTGCAAGCACTTGTGCAAGCACTTGTGCAAGCACAGGTGCAAGCACTTGTGCAGATACCAATGTAGACACCTGTGCAAGCACCTGTGCAAACACCTGTGCAAGCACAGAATAC |
| 230 | BF09/SAND1 | Burkina Faso | DP | 2009 |  | I | [This study (gallardo@inia.es)](http://asf-referencelab.info/asf/images/files/publicaciones/Gianmaroli_et_al_2011.pdf) | ATGCAGCCTACTCACCACGCAGAGATAAGCTTTCAGGATAGAGATACAGCTCTTCCAGACGCATGTTCATCTATATCGGATATTAGCCCCGTTACGTATCCGATCACATTACCTATTATTAAAAACATTTCCGTAACTGCTCATGGTATCAATCTTATCGATAAGTTTCCATCAAAGTTCTGCAGCTCTTACATACCCTTCCACTACGGAGGCAATGCAATTAAAACCCCCGATGATCCGGGTGCGATGATGATTACCTTTGCTTTGAAGCCACGGGAGGAATACCAACCCAGTGGTCATATTAACGTATCCAGAGCAAGAGAATTTTATATTAGTTGGGACACGGATTACGTGGGGTCTATCACTACGGCTGATCTTGTGGTATCGGCATCTGCTATTAACTT |  | [This study (gallardo@inia.es)](http://asf-referencelab.info/asf/images/files/publicaciones/Gianmaroli_et_al_2011.pdf) | AGTGCGTATACTTGTGCAAGCACTTGTGCAGATACCAATGTAGACACCTGTGCAAGCACTTGTGCAAGCACTTGTGCAAGCACTTGTGCAAGCACAGGTGCAAGCACTTGTGCAGATACCAATGTAGACACCTGTGCAAGCACTTGTGCAAGCACTTGTGCAAGCACTTGTGCAAGCACTTGTGCAAGCACTTGTGCAAGCACAGGTGCAAGCACTTGTGCAGATACCAATGTAGACACCTGTGCAAGCACTTGTGCAAGCACTTGTGCAAGCACTTGTGCAAGCACTTGTGCAAGCACTTGTGCAAGCACAGGTGCAAGCACTTGTGCAGATACCAATGTAGACACCTGTGCAAGCACTTGTGCAAGCACTTGTGCAAGCACTTGTGCAAGCACTTGTGCAAGCACTTGTGCAAGCACAGGTGCAAGCACTTGTGCAGATACCAATGTAGACACCTGTGCAAGCACTTGTGCAAGCACTTGTGCAAGCACTTGTGCAAGCACTTGTGCAAGCACTTGTGCAAGCACAGGTGCAAGCACTTGTGCAGATACCAATGTAGACACCTGTGCAAGCACCTGTGCAAACACCTGTGCAAGCACAGAATAC |
| 231 | BF09/SeA1 | Burkina Faso | DP | 2009 |  | I | [This study (gallardo@inia.es)](http://asf-referencelab.info/asf/images/files/publicaciones/Gianmaroli_et_al_2011.pdf) | ATGCAGCCTACTCACCACGCAGAGATAAGCTTTCAGGATAGAGATACAGCTCTTCCAGACGCATGTTCATCTATATCGGATATTAGCCCCGTTACGTATCCGATCACATTACCTATTATTAAAAACATTTCCGTAACTGCTCATGGTATCAATCTTATCGATAAGTTTCCATCAAAGTTCTGCAGCTCTTACATACCCTTCCACTACGGAGGCAATGCAATTAAAACCCCCGATGATCCGGGTGCGATGATGATTACCTTTGCTTTGAAGCCACGGGAGGAATACCAACCCAGTGGTCATATTAACGTATCCAGAGCAAGAGAATTTTATATTAGTTGGGACACGGATTACGTGGGGTCTATCACTACGGCTGATCTTGTGGTATCGGCATCTGCTATTAACTT |  | [This study (gallardo@inia.es)](http://asf-referencelab.info/asf/images/files/publicaciones/Gianmaroli_et_al_2011.pdf) | AGTGCGTATACTTGTGCAAGCACTTGTGCAGATACCAATGTAGACACCTGTGCAAGCACTTGTGCAAGCACTTGTGCAAGCACTTGTGCAAGCACAGGTGCAAGCACTTGTGCAGATACCAATGTAGACACCTGTGCAAGCACTTGTGCAAGCACTTGTGCAAGCACTTGTGCAAGCACTTGTGCAAGCACAGGTGCAAGCACTTGTGCAGATACCAATGTAGACACCTGTGCAAGCACTTGTGCAAGCACTTGTGCAAGCACTTGTGCAAGCACTTGTGCAAGCACTTGTGCAAGCACAGGTGCAAGCACTTGTGCAGATACCAATGTAGACACCTGTGCAAGCACCTGTGCAAACACCTGTGCAAGCACAGAATAC |
| 232 | BF09/SeB4 | Burkina Faso | DP | 2009 |  | I | [This study (gallardo@inia.es)](http://asf-referencelab.info/asf/images/files/publicaciones/Gianmaroli_et_al_2011.pdf) | ATGCAGCCTACTCACCACGCAGAGATAAGCTTTCAGGATAGAGATACAGCTCTTCCAGACGCATGTTCATCTATATCGGATATTAGCCCCGTTACGTATCCGATCACATTACCTATTATTAAAAACATTTCCGTAACTGCTCATGGTATCAATCTTATCGATAAGTTTCCATCAAAGTTCTGCAGCTCTTACATACCCTTCCACTACGGAGGCAATGCAATTAAAACCCCCGATGATCCGGGTGCGATGATGATTACCTTTGCTTTGAAGCCACGGGAGGAATACCAACCCAGTGGTCATATTAACGTATCCAGAGCAAGAGAATTTTATATTAGTTGGGACACGGATTACGTGGGGTCTATCACTACGGCTGATCTTGTGGTATCGGCATCTGCTATTAACTT |  | [This study (gallardo@inia.es)](http://asf-referencelab.info/asf/images/files/publicaciones/Gianmaroli_et_al_2011.pdf) | AGTGCGTATACTTGTGCAAGCACTTGTGCAGATACCAATGTAGACACCTGTGCAAGCACTTGTGCAAGCACTTGTGCAAGCACTTGTGCAAGCACAGGTGCAAGCACTTGTGCAGATACCAATGTAGACACCTGTGCAAGCACTTGTGCAAGCACTTGTGCAAGCACTTGTGCAAGCACTTGTGCAAGCACAGGTGCAAGCACTTGTGCAGATACCAATGTAGACACCTGTGCAAGCACTTGTGCAAGCACTTGTGCAAGCACTTGTGCAAGCACTTGTGCAAGCACTTGTGCAAGCACAGGTGCAAGCACTTGTGCAGATACCAATGTAGACACCTGTGCAAGCACCTGTGCAAACACCTGTGCAAGCACAGAATAC |
| 233 | BF09/SeC7 | Burkina Faso | DP | 2009 |  | I | [This study (gallardo@inia.es)](http://asf-referencelab.info/asf/images/files/publicaciones/Gianmaroli_et_al_2011.pdf) | ATGCAGCCTACTCACCACGCAGAGATAAGCTTTCAGGATAGAGATACAGCTCTTCCAGACGCATGTTCATCTATATCGGATATTAGCCCCGTTACGTATCCGATCACATTACCTATTATTAAAAACATTTCCGTAACTGCTCATGGTATCAATCTTATCGATAAGTTTCCATCAAAGTTCTGCAGCTCTTACATACCCTTCCACTACGGAGGCAATGCAATTAAAACCCCCGATGATCCGGGTGCGATGATGATTACCTTTGCTTTGAAGCCACGGGAGGAATACCAACCCAGTGGTCATATTAACGTATCCAGAGCAAGAGAATTTTATATTAGTTGGGACACGGATTACGTGGGGTCTATCACTACGGCTGATCTTGTGGTATCGGCATCTGCTATTAACTT |  | [This study (gallardo@inia.es)](http://asf-referencelab.info/asf/images/files/publicaciones/Gianmaroli_et_al_2011.pdf) | AGTGCGTATACTTGTGCAAGCACTTGTGCAGATACCAATGTAGACACCTGTGCAAGCACTTGTGCAAGCACTTGTGCAAGCACTTGTGCAAGCACAGGTGCAAGCACTTGTGCAGATACCAATGTAGACACCTGTGCAAGCACTTGTGCAAGCACTTGTGCAAGCACTTGTGCAAGCACTTGTGCAAGCACAGGTGCAAGCACTTGTGCAGATACCAATGTAGACACCTGTGCAAGCACTTGTGCAAGCACTTGTGCAAGCACTTGTGCAAGCACTTGTGCAAGCACTTGTGCAAGCACAGGTGCAAGCACTTGTGCAGATACCAATGTAGACACCTGTGCAAGCACCTGTGCAAACACCTGTGCAAGCACAGAATAC |
| 234 | BF09/SeD3 | Burkina Faso | DP | 2009 |  | I | [This study (gallardo@inia.es)](http://asf-referencelab.info/asf/images/files/publicaciones/Gianmaroli_et_al_2011.pdf) | ATGCAGCCTACTCACCACGCAGAGATAAGCTTTCAGGATAGAGATACAGCTCTTCCAGACGCATGTTCATCTATATCGGATATTAGCCCCGTTACGTATCCGATCACATTACCTATTATTAAAAACATTTCCGTAACTGCTCATGGTATCAATCTTATCGATAAGTTTCCATCAAAGTTCTGCAGCTCTTACATACCCTTCCACTACGGAGGCAATGCAATTAAAACCCCCGATGATCCGGGTGCGATGATGATTACCTTTGCTTTGAAGCCACGGGAGGAATACCAACCCAGTGGTCATATTAACGTATCCAGAGCAAGAGAATTTTATATTAGTTGGGACACGGATTACGTGGGGTCTATCACTACGGCTGATCTTGTGGTATCGGCATCTGCTATTAACTT |  | [This study (gallardo@inia.es)](http://asf-referencelab.info/asf/images/files/publicaciones/Gianmaroli_et_al_2011.pdf) | AGTGCGTATACTTGTGCAAGCACTTGTGCAGATACCAATGTAGACACCTGTGCAAGCACTTGTGCAAGCACTTGTGCAAGCACTTGTGCAAGCACAGGTGCAAGCACTTGTGCAGATACCAATGTAGACACCTGTGCAAGCACTTGTGCAAGCACTTGTGCAAGCACTTGTGCAAGCACTTGTGCAAGCACAGGTGCAAGCACTTGTGCAGATACCAATGTAGACACCTGTGCAAGCACTTGTGCAAGCACTTGTGCAAGCACTTGTGCAAGCACTTGTGCAAGCACTTGTGCAAGCACAGGTGCAAGCACTTGTGCAGATACCAATGTAGACACCTGTGCAAGCACCTGTGCAAACACCTGTGCAAGCACAGAATAC |
| 235 | BF09/SeE1 | Burkina Faso | DP | 2009 |  | I | [This study (gallardo@inia.es)](http://asf-referencelab.info/asf/images/files/publicaciones/Gianmaroli_et_al_2011.pdf) | ATGCAGCCTACTCACCACGCAGAGATAAGCTTTCAGGATAGAGATACAGCTCTTCCAGACGCATGTTCATCTATATCGGATATTAGCCCCGTTACGTATCCGATCACATTACCTATTATTAAAAACATTTCCGTAACTGCTCATGGTATCAATCTTATCGATAAGTTTCCATCAAAGTTCTGCAGCTCTTACATACCCTTCCACTACGGAGGCAATGCAATTAAAACCCCCGATGATCCGGGTGCGATGATGATTACCTTTGCTTTGAAGCCACGGGAGGAATACCAACCCAGTGGTCATATTAACGTATCCAGAGCAAGAGAATTTTATATTAGTTGGGACACGGATTACGTGGGGTCTATCACTACGGCTGATCTTGTGGTATCGGCATCTGCTATTAACTT |  | [This study (gallardo@inia.es)](http://asf-referencelab.info/asf/images/files/publicaciones/Gianmaroli_et_al_2011.pdf) | AGTGCGTATACTTGTGCAAGCACTTGTGCAGATACCAATGTAGACACCTGTGCAAGCACTTGTGCAAGCACTTGTGCAAGCACTTGTGCAAGCACAGGTGCAAGCACTTGTGCAGATACCAATGTAGACACCTGTGCAAGCACTTGTGCAAGCACTTGTGCAAGCACTTGTGCAAGCACTTGTGCAAGCACAGGTGCAAGCACTTGTGCAGATACCAATGTAGACACCTGTGCAAGCACTTGTGCAAGCACTTGTGCAAGCACTTGTGCAAGCACTTGTGCAAGCACTTGTGCAAGCACAGGTGCAAGCACTTGTGCAGATACCAATGTAGACACCTGTGCAAGCACCTGTGCAAACACCTGTGCAAGCACAGAATAC |
| 236 | CON09/PN003 | Republic of Congo | DP | 2009 | HQ645947 | I | [Gallardo et al 2011](http://asf-referencelab.info/asf/images/files/publicaciones/Gianmaroli_et_al_2011.pdf) | ATGCAGCCTACTCACCACGCAGAGATAAGCTTTCAGGATAGAGATACAGCTCTTCCAGACGCATGTTCATCTATATCGGATATTAGCCCCGTTACGTATCCGATCACATTACCTATTATTAAAAACATTTCCGTAACTGCTCATGGTATCAATCTTATCGATAAGTTTCCATCAAAGTTCTGCAGCTCTTACATACCCTTCCACTACGGAGGCAATGCAATTAAAACCCCCGATGATCCGGGTGCGATGATGATTACCTTTGCTTTGAAGCCACGGGAGGAATACCAACCCAGTGGTCATATTAACGTATCCAGAGCAAGAGAATTTTATATTAGTTGGGACACGGATTACGTGGGGTCTATCACTACGGCTGATCTTGTGGTATCGGCATCTGCTATTAACTT | HQ645949 | [Gallardo et al 2011](http://asf-referencelab.info/asf/images/files/publicaciones/Gianmaroli_et_al_2011.pdf) | AGTGCGTATACTTGTGCAAGCACTTGTGCAAGCACTTGTGCAGATACCAATGTAGACACCTGTGCAAGCACTTGTGCAGATACCAATGTAAACACTTGTGCAAGCATGTGTGCAGATACCAATGTAGACACCTGTGCAAGCACCTGTGCAAGCACTTGTGCAAGCACTTGTGCAAGCACAGAATAC |
| 237 | CON09/Ni16 | Republic of Congo | DP | 2009 | HQ645943 | I | [Gallardo et al 2011](http://asf-referencelab.info/asf/images/files/publicaciones/Gallardo_et_al_2011c_Congo.pdf) | ATGCAGCCTACTCACCACGCAGAGATAAGCTTTCAGGATAGAGATACAGCTCTTCCAGACGCATGTTCATCTATATCGGATATTAGCCCCGTTACGTATCCGATCACATTACCTATTATTAAAAACATTTCCGTAACTGCTCATGGTATCAATCTTATCGATAAGTTTCCATCAAAGTTCTGCAGCTCTTACATACCCTTCCACTACGGAGGCAATGCAATTAAAACCCCCGATGATCCGGGTGCGATGATGATTACCTTTGCTTTGAAGCCACGGGAGGAATACCAACCCAGTGGTCATATTAACGTATCCAGAGCAAGAGAATTTTATATTAGTTGGGACACGGATTACGTGGGGTCTATCACTACGGCTGATCTTGTGGTATCGGCATCTGCTATTAACTT | HQ645957 | [Gallardo et al 2011](http://asf-referencelab.info/asf/images/files/publicaciones/Gallardo_et_al_2011c_Congo.pdf) | AGTGCGTATACTTGTGCAAGCACTTGTGCAAGCACTTGTGCAAGCACTTGTGCAAGCACTTGTGCAAGCACTTGTGCAAGCACTTGTGCAAGCACTTGTGCAAGCACTTGTGCAAGCACTTGTGCAAACACAGAATAC |
| 238 | Tog09/P1 | Togo | DP | 2009 |  | I | [This study (gallardo@inia.es)](http://wwwnc.cdc.gov/eid/article/20/9/14-0554_article) | ATGCAGCCTACTCACCACGCAGAGATAAGCTTTCAGGATAGAGATACAGCTCTTCCAGACGCATGTTCATCTATATCGGATATTAGCCCCGTTACGTATCCGATCACATTACCTATTATTAAAAACATTTCCGTAACTGCTCATGGTATCAATCTTATCGATAAGTTTCCATCAAAGTTCTGCAGCTCTTACATACCCTTCCACTACGGAGGCAATGCAATTAAAACCCCCGATGATCCGGGTGCGATGATGATTACCTTTGCTTTGAAGCCACGGGAGGAATACCAACCCAGTGGTCATATTAACGTATCCAGAGCAAGAGAATTTTATATTAGTTGGGACACGGATTACGTGGGGTCTATCACTACGGCTGATCTTGTGGTATCGGCATCTGCTATTAACTT |  | [This study (gallardo@inia.es)](http://wwwnc.cdc.gov/eid/article/20/9/14-0554_article) | AGTGCGTATACTTGTGCAAGCACTTGTGCAGATACCAATGTAGACACCTGTGCAAGCACTTGTGCAAGCACTTGTGCAAGCACTTGTGCAAGCACTTGTGCAAGCACAGGTGCAAGCACTTGTGCAGATACCAATGTAGACACCTGTGCAAGCACTTGTGCAAGCACTTGTGCAAGCACTTGTGCAAGCACTTGTGCAAGCACAGGTGCAAGCACTTGTGCAGATACCAATGTAGACACCTGTGCAAGCACCTGTGCAAACACCTGTGCAAGCACAGAATAC |
| 239 | Tog09/P2 | Togo | DP | 2009 |  | I | [This study (gallardo@inia.es)](http://wwwnc.cdc.gov/eid/article/20/9/14-0554_article) | ATGCAGCCTACTCACCACGCAGAGATAAGCTTTCAGGATAGAGATACAGCTCTTCCAGACGCATGTTCATCTATATCGGATATTAGCCCCGTTACGTATCCGATCACATTACCTATTATTAAAAACATTTCCGTAACTGCTCATGGTATCAATCTTATCGATAAGTTTCCATCAAAGTTCTGCAGCTCTTACATACCCTTCCACTACGGAGGCAATGCAATTAAAACCCCCGATGATCCGGGTGCGATGATGATTACCTTTGCTTTGAAGCCACGGGAGGAATACCAACCCAGTGGTCATATTAACGTATCCAGAGCAAGAGAATTTTATATTAGTTGGGACACGGATTACGTGGGGTCTATCACTACGGCTGATCTTGTGGTATCGGCATCTGCTATTAACTT |  | [This study (gallardo@inia.es)](http://wwwnc.cdc.gov/eid/article/20/9/14-0554_article) | AGTGCGTATACTTGTGCAAGCACTTGTGCAGATACCAATGTAGACACCTGTGCAAGCACTTGTGCAAGCACTTGTGCAAGCACTTGTGCAAGCACTTGTGCAAGCACAGGTGCAAGCACTTGTGCAGATACCAATGTAGACACCTGTGCAAGCACTTGTGCAAGCACTTGTGCAAGCACTTGTGCAAGCACTTGTGCAAGCACAGGTGCAAGCACTTGTGCAGATACCAATGTAGACACCTGTGCAAGCACCTGTGCAAACACCTGTGCAAGCACAGAATAC |
| 240 | Tog09/P3 | Togo | DP | 2009 |  | I | [This study (gallardo@inia.es)](http://wwwnc.cdc.gov/eid/article/20/9/14-0554_article) | ATGCAGCCTACTCACCACGCAGAGATAAGCTTTCAGGATAGAGATACAGCTCTTCCAGACGCATGTTCATCTATATCGGATATTAGCCCCGTTACGTATCCGATCACATTACCTATTATTAAAAACATTTCCGTAACTGCTCATGGTATCAATCTTATCGATAAGTTTCCATCAAAGTTCTGCAGCTCTTACATACCCTTCCACTACGGAGGCAATGCAATTAAAACCCCCGATGATCCGGGTGCGATGATGATTACCTTTGCTTTGAAGCCACGGGAGGAATACCAACCCAGTGGTCATATTAACGTATCCAGAGCAAGAGAATTTTATATTAGTTGGGACACGGATTACGTGGGGTCTATCACTACGGCTGATCTTGTGGTATCGGCATCTGCTATTAACTT |  | [This study (gallardo@inia.es)](http://wwwnc.cdc.gov/eid/article/20/9/14-0554_article) | AGTGCGTATACTTGTGCAAGCACTTGTGCAGATACCAATGTAGACACCTGTGCAAGCACTTGTGCAAGCACTTGTGCAAGCACTTGTGCAAGCACTTGTGCAAGCACAGGTGCAAGCACTTGTGCAGATACCAATGTAGACACCTGTGCAAGCACTTGTGCAAGCACTTGTGCAAGCACTTGTGCAAGCACTTGTGCAAGCACAGGTGCAAGCACTTGTGCAGATACCAATGTAGACACCTGTGCAAGCACCTGTGCAAACACCTGTGCAAGCACAGAATAC |
| 241 | StPet09 | Russia | DP | 2009 | JX857520 | II | [Gallardo et al 2014](http://wwwnc.cdc.gov/eid/article/20/9/14-0554_article) | ATGCAGCCCACTCACCACGCAGAGATAAGCTTTCAGGATAGAGATACAGCTCTTCCAGACGCATGTTCATCTATATCTGATATTAGCCCCGTTACGTATCCGATCACATTACCTATTATTAAAAACATTTCCGTAACTGCTCATGGTATCAATCTTATCGATAAATTTCCATCAAAGTTCTGCAGCTCTTACATACCCTTCCACTACGGAGGCAATGCGATTAAAACCCCCGATGATCCGGGTGCGATGATGATTACCTTTGCTTTGAAGCCACGGGAGGAATACCAACCCAGTGGTCATATTAACGTATCCAGAGCAAGAGAATTTTATATTAGTTGGGACACGGATTACGTGGGGTCTATCACTACGGCTGATCTTGTGGTATCGGCATCTGCTATTAACTT | JX857534 | [Gallardo et al 2014](http://wwwnc.cdc.gov/eid/article/20/9/14-0554_article) | AGTGCGTATACTTGTGCAGATACCAATGTAGACACTTGTGCAAGCATGTGTGCAGATACCAATGTAGACACCTGTGCAAGCATGTGTGCAGATACCAATGTAGATACCTGTGCAAGCACTTGTACAAGCACAGAATAC |
| 242 | Rostov09 | Russia | DP | 2009 | JX857518 | II | [Gallardo et al 2014](http://wwwnc.cdc.gov/eid/article/20/9/14-0554_article) | ATGCAGCCCACTCACCACGCAGAGATAAGCTTTCAGGATAGAGATACAGCTCTTCCAGACGCATGTTCATCTATATCTGATATTAGCCCCGTTACGTATCCGATCACATTACCTATTATTAAAAACATTTCCGTAACTGCTCATGGTATCAATCTTATCGATAAATTTCCATCAAAGTTCTGCAGCTCTTACATACCCTTCCACTACGGAGGCAATGCGATTAAAACCCCCGATGATCCGGGTGCGATGATGATTACCTTTGCTTTGAAGCCACGGGAGGAATACCAACCCAGTGGTCATATTAACGTATCCAGAGCAAGAGAATTTTATATTAGTTGGGACACGGATTACGTGGGGTCTATCACTACGGCTGATCTTGTGGTATCGGCATCTGCTATTAACTT | JX857532 | [Gallardo et al 2014](http://wwwnc.cdc.gov/eid/article/20/9/14-0554_article) | AGTGCGTATACTTGTGCAGATACCAATGTAGACACTTGTGCAAGCATGTGTGCAGATACCAATGTAGACACCTGTGCAAGCATGTGTGCAGATACCAATGTAGATACCTGTGCAAGCACTTGTACAAGCACAGAATAC |
| 243 | Dagestan09 | Russia | Wild pig | 2009 | JX857517 | II | [Gallardo et al 2014](http://wwwnc.cdc.gov/eid/article/20/9/14-0554_article) | ATGCAGCCCACTCACCACGCAGAGATAAGCTTTCAGGATAGAGATACAGCTCTTCCAGACGCATGTTCATCTATATCTGATATTAGCCCCGTTACGTATCCGATCACATTACCTATTATTAAAAACATTTCCGTAACTGCTCATGGTATCAATCTTATCGATAAATTTCCATCAAAGTTCTGCAGCTCTTACATACCCTTCCACTACGGAGGCAATGCGATTAAAACCCCCGATGATCCGGGTGCGATGATGATTACCTTTGCTTTGAAGCCACGGGAGGAATACCAACCCAGTGGTCATATTAACGTATCCAGAGCAAGAGAATTTTATATTAGTTGGGACACGGATTACGTGGGGTCTATCACTACGGCTGATCTTGTGGTATCGGCATCTGCTATTAACTT | JX857531 | [Gallardo et al 2014](http://wwwnc.cdc.gov/eid/article/20/9/14-0554_article) | AGTGCGTATACTTGTGCAGATACCAATGTAGACACTTGTGCAAGCATGTGTGCAGATACCAATGTAGACACCTGTGCAAGCATGTGTGCAGATACCAATGTAGATACCTGTGCAAGCACTTGTACAAGCACAGAATAC |
| 244 | Kalmykia09 | Russia | DP | 2009 | X857519 | II | [Gallardo et al 2014](http://wwwnc.cdc.gov/eid/article/20/9/14-0554_article) | ATGCAGCCCACTCACCACGCAGAGATAAGCTTTCAGGATAGAGATACAGCTCTTCCAGACGCATGTTCATCTATATCTGATATTAGCCCCGTTACGTATCCGATCACATTACCTATTATTAAAAACATTTCCGTAACTGCTCATGGTATCAATCTTATCGATAAATTTCCATCAAAGTTCTGCAGCTCTTACATACCCTTCCACTACGGAGGCAATGCGATTAAAACCCCCGATGATCCGGGTGCGATGATGATTACCTTTGCTTTGAAGCCACGGGAGGAATACCAACCCAGTGGTCATATTAACGTATCCAGAGCAAGAGAATTTTATATTAGTTGGGACACGGATTACGTGGGGTCTATCACTACGGCTGATCTTGTGGTATCGGCATCTGCTATTAACTT | JX857533 | [Gallardo et al 2014](http://wwwnc.cdc.gov/eid/article/20/9/14-0554_article) | AGTGCGTATACTTGTGCAGATACCAATGTAGACACTTGTGCAAGCATGTGTGCAGATACCAATGTAGACACCTGTGCAAGCATGTGTGCAGATACCAATGTAGATACCTGTGCAAGCACTTGTACAAGCACAGAATAC |
| 245 | Cam10/YGT9 | Cameroon | DP | 2010 |  | I | [This study (gallardo@inia.es)](http://wwwnc.cdc.gov/eid/article/20/9/14-0554_article) | ATGCAGCCTACTCACCACGCAGAGATAAGCTTTCAGGATAGAGATACAGCTCTTCCAGACGCATGTTCATCTATATCGGATATTAGCCCCGTTACGTATCCGATCACATTACCTATTATTAAAAACATTTCCGTAACTGCTCATGGTATCAATCTTATCGATAAGTTTCCATCAAAGTTCTGCAGCTCTTACATACCCTTCCACTACGGAGGCAATGCAATTAAAACCCCCGATGATCCGGGTGCGATGATGATTACCTTTGCTTTGAAGCCACGGGAGGAATACCAACCCAGTGGTCATATTAACGTATCCAGAGCAAGAGAATTTTATATTAGTTGGGACACGGATTACGTGGGGTCTATCACTACGGCTGATCTTGTGGTATCGGCATCTGCTATTAACTT |  | [This study (gallardo@inia.es)](http://wwwnc.cdc.gov/eid/article/20/9/14-0554_article) | AGTGCGTATACTTGTGCAAGCACTTGTGCAGATACCAATGTAGACACCTGTGCAAGCACTTGTGCAAGCACTTGTGCAAGCACTTGTGCAAGCACTTGTGCAAGCACAGGTGCAAGCACTTGTGCAGATACCAATGTAGACACCTGTGCAAGCACTTGTGCAGATACCAATGTAAACACTTGTGCAAGCATGTGTGCAGATACCAATGTAGACACCTGTGCAAGCACCTGTGCAAACACCTGTGCAAGCACAGAATAC |
| 246 | Cam10/GGT5 | Cameroon | DP | 2010 |  | I | [This study (gallardo@inia.es)](http://wwwnc.cdc.gov/eid/article/20/9/14-0554_article) | ATGCAGCCTACTCACCACGCAGAGATAAGCTTTCAGGATAGAGATACAGCTCTTCCAGACGCATGTTCATCTATATCGGATATTAGCCCCGTTACGTATCCGATCACATTACCTATTATTAAAAACATTTCCGTAACTGCTCATGGTATCAATCTTATCGATAAGTTTCCATCAAAGTTCTGCAGCTCTTACATACCCTTCCACTACGGAGGCAATGCAATTAAAACCCCCGATGATCCGGGTGCGATGATGATTACCTTTGCTTTGAAGCCACGGGAGGAATACCAACCCAGTGGTCATATTAACGTATCCAGAGCAAGAGAATTTTATATTAGTTGGGACACGGATTACGTGGGGTCTATCACTACGGCTGATCTTGTGGTATCGGCATCTGCTATTAACTT |  | [This study (gallardo@inia.es)](http://wwwnc.cdc.gov/eid/article/20/9/14-0554_article) | AGTGCGTATACTTGTGCAAGCACTTGTGCAGATACCAATGTAGACACCTGTGCAAGCACTTGTGCAAGCACTTGTGCAAGCACTTGTGCAAGCACTTGTGCAAGCACAGGTGCAAGCACTTGTGCAGATACCAATGTAGACACCTGTGCAAGCACTTGTGCAGATACCAATGTAAACACTTGTGCAAGCATGTGTGCAGATACCAATGTAGACACCTGTGCAAGCACCTGTGCAAACACCTGTGCAAGCACAGAATAC |
| 247 | Cam10/OGT13 | Cameroon | DP | 2010 |  | I | [This study (gallardo@inia.es)](http://wwwnc.cdc.gov/eid/article/20/9/14-0554_article) | ATGCAGCCTACTCACCACGCAGAGATAAGCTTTCAGGATAGAGATACAGCTCTTCCAGACGCATGTTCATCTATATCGGATATTAGCCCCGTTACGTATCCGATCACATTACCTATTATTAAAAACATTTCCGTAACTGCTCATGGTATCAATCTTATCGATAAGTTTCCATCAAAGTTCTGCAGCTCTTACATACCCTTCCACTACGGAGGCAATGCAATTAAAACCCCCGATGATCCGGGTGCGATGATGATTACCTTTGCTTTGAAGCCACGGGAGGAATACCAACCCAGTGGTCATATTAACGTATCCAGAGCAAGAGAATTTTATATTAGTTGGGACACGGATTACGTGGGGTCTATCACTACGGCTGATCTTGTGGTATCGGCATCTGCTATTAACTT |  | [This study (gallardo@inia.es)](http://wwwnc.cdc.gov/eid/article/20/9/14-0554_article) | AGTGCGTATACTTGTGCAAGCACTTGTGCAGATACCAATGTAGACACCTGTGCAAGCACTTGTGCAAGCACTTGTGCAAGCACTTGTGCAAGCACTTGTGCAAGCACAGGTGCAAGCACTTGTGCAGATACCAATGTAGACACCTGTGCAAGCACTTGTGCAGATACCAATGTAAACACTTGTGCAAGCATGTGTGCAGATACCAATGTAGACACCTGTGCAAGCACCTGTGCAAACACCTGTGCAAGCACAGAATAC |
| 248 | UG10/Tk3.2 | Uganda | Tick | 2010 |  | X | [This study (gallardo@inia.es)](http://wwwnc.cdc.gov/eid/article/20/9/14-0554_article) | ATGCAGCCTACCCACCACGCAGAGGTAAGCTTTCAGGATAGAGATACAGCTCTTCCAGATGCATGTTCATCCATATCAGATATTTCCCCCATTACTTATCCGATCACGTTACCTATTATTAAAAACATTTCCGTCACTGCTCATGGTATCAATCTTATCGATAAATTTCCATCAAAGTTCTGCAGCTCTTACATACCCTTTCACTACGGAGGCAATTCGATTAAAACCCCCGACGATCCGGGCGCGATGATGATTACCTTTGCTTTGAAACCACGGGAGGAATACCAACCCAGTGGTCATATTAACGTATCCAGAGCAAGAGAGTTTTATATTAGCTGGGACACAGATTATGTGGGGTCTATCACCACGGCCGATCTTGTGGTATCGGCATCCGCTATTAACTT |  | [This study (gallardo@inia.es)](http://wwwnc.cdc.gov/eid/article/20/9/14-0554_article) | AGTGCGTATACCTGTGCAAGCACTTGTGCAAGCACTTGTGCAAGCACTTGTGCAAGCACCTGTGCAGACACCAATGTAGACACTTGTACAGACACCTGTGCAAGCACCTGTGCAGACACCAATGTAGACACTTGTGCAAGCACTTGTGCAAGCACTTGTGCAGACACCTGTGCAAGCACGGAATACACCGAT |
| 249 | BF10/DalO22 | Burkina Faso | DP | 2010 |  | I | [This study (gallardo@inia.es)](http://wwwnc.cdc.gov/eid/article/20/9/14-0554_article) | ATGCAGCCTACTCACCACGCAGAGATAAGCTTTCAGGATAGAGATACAGCTCTTCCAGACGCATGTTCATCTATATCGGATATTAGCCCCGTTACGTATCCGATCACATTACCTATTATTAAAAACATTTCCGTAACTGCTCATGGTATCAATCTTATCGATAAGTTTCCATCAAAGTTCTGCAGCTCTTACATACCCTTCCACTACGGAGGCAATGCAATTAAAACCCCCGATGATCCGGGTGCGATGATGATTACCTTTGCTTTGAAGCCACGGGAGGAATACCAACCCAGTGGTCATATTAACGTATCCAGAGCAAGAGAATTTTATATTAGTTGGGACACGGATTACGTGGGGTCTATCACTACGGCTGATCTTGTGGTATCGGCATCTGCTATTAACTT |  | [This study (gallardo@inia.es)](http://wwwnc.cdc.gov/eid/article/20/9/14-0554_article) | AGTGCGTATACTTGTGCAAGCACTTGTGCAGATACCAATGTAGACACCTGTGCAAGCACTTGTGCAAGCACTTGTGCAAGCACTTGTGCAAGCACTTGTGCAAGCACAGGTGCAAGCACTTGTGCAGATACCAATGTAGACACCTGTGCAAGCACTTGTGCAAGCACTTGTGCAAGCACTTGTGCAAGCACTTGTGCAAGCACTTGTGCAAGCACTTGTGCAAGCACTTGTGCAAGCACAGGTGCAAGCACTTGTGCAGATACCAATGTAGACACCTGTGCAAGCACCTGTGCAAACACCTGTGCAAGCACAGAATAC |
| 250 | BF10/GasN26 | Burkina Faso | DP | 2010 |  | I | [This study (gallardo@inia.es)](http://wwwnc.cdc.gov/eid/article/20/9/14-0554_article) | ATGCAGCCTACTCACCACGCAGAGATAAGCTTTCAGGATAGAGATACAGCTCTTCCAGACGCATGTTCATCTATATCGGATATTAGCCCCGTTACGTATCCGATCACATTACCTATTATTAAAAACATTTCCGTAACTGCTCATGGTATCAATCTTATCGATAAGTTTCCATCAAAGTTCTGCAGCTCTTACATACCCTTCCACTACGGAGGCAATGCAATTAAAACCCCCGATGATCCGGGTGCGATGATGATTACCTTTGCTTTGAAGCCACGGGAGGAATACCAACCCAGTGGTCATATTAACGTATCCAGAGCAAGAGAATTTTATATTAGTTGGGACACGGATTACGTGGGGTCTATCACTACGGCTGATCTTGTGGTATCGGCATCTGCTATTAACTT |  | [This study (gallardo@inia.es)](http://wwwnc.cdc.gov/eid/article/20/9/14-0554_article) | AGTGCGTATACTTGTGCAAGCACTTGTGCAGATACCAATGTAGACACCTGTGCAAGCACTTGTGCAAGCACTTGTGCAAGCACTTGTGCAAGCACTTGTGCAAGCACAGGTGCAAGCACTTGTGCAGATACCAATGTAGACACCTGTGCAAGCACTTGTGCAAGCACTTGTGCAAGCACTTGTGCAAGCACTTGTGCAAGCACTTGTGCAAGCACTTGTGCAAGCACTTGTGCAAGCACAGGTGCAAGCACTTGTGCAGATACCAATGTAGACACCTGTGCAAGCACCTGTGCAAACACCTGTGCAAGCACAGAATAC |
| 251 | BF10/NanB9 | Burkina Faso | DP | 2010 |  | I | [This study (gallardo@inia.es)](http://wwwnc.cdc.gov/eid/article/20/9/14-0554_article) | ATGCAGCCTACTCACCACGCAGAGATAAGCTTTCAGGATAGAGATACAGCTCTTCCAGACGCATGTTCATCTATATCGGATATTAGCCCCGTTACGTATCCGATCACATTACCTATTATTAAAAACATTTCCGTAACTGCTCATGGTATCAATCTTATCGATAAGTTTCCATCAAAGTTCTGCAGCTCTTACATACCCTTCCACTACGGAGGCAATGCAATTAAAACCCCCGATGATCCGGGTGCGATGATGATTACCTTTGCTTTGAAGCCACGGGAGGAATACCAACCCAGTGGTCATATTAACGTATCCAGAGCAAGAGAATTTTATATTAGTTGGGACACGGATTACGTGGGGTCTATCACTACGGCTGATCTTGTGGTATCGGCATCTGCTATTAACTT |  | [This study (gallardo@inia.es)](http://wwwnc.cdc.gov/eid/article/20/9/14-0554_article) | AGTGCGTATACTTGTGCAAGCACTTGTGCAGATACCAATGTAGACACCTGTGCAAGCACTTGTGCAAGCACTTGTGCAAGCACTTGTGCAAGCACAGGTGCAAGCACTTGTGCAGATACCAATGTAGACACCTGTGCAAGCACTTGTGCAAGCACTTGTGCAAGCACTTGTGCAAGCACTTGTGCAAGCACTTGTGCAAGCACAGGTGCAAGCACTTGTGCAGATACCAATGTAGACACCTGTGCAAGCACTTGTGCAAGCACTTGTGCAAGCACTTGTGCAAGCACTTGTGCAAGCACTTGTGCAAGCACAGGTGCAAGCACTTGTGCAGATACCAATGTAGACACCTGTGCAAGCACCTGTGCAAACACCTGTGCAAGCACAGAATAC |
| 252 | BF10/NiouK34 | Burkina Faso | DP | 2010 |  | I | [This study (gallardo@inia.es)](http://wwwnc.cdc.gov/eid/article/20/9/14-0554_article) | ATGCAGCCTACTCACCACGCAGAGATAAGCTTTCAGGATAGAGATACAGCTCTTCCAGACGCATGTTCATCTATATCGGATATTAGCCCCGTTACGTATCCGATCACATTACCTATTATTAAAAACATTTCCGTAACTGCTCATGGTATCAATCTTATCGATAAGTTTCCATCAAAGTTCTGCAGCTCTTACATACCCTTCCACTACGGAGGCAATGCAATTAAAACCCCCGATGATCCGGGTGCGATGATGATTACCTTTGCTTTGAAGCCACGGGAGGAATACCAACCCAGTGGTCATATTAACGTATCCAGAGCAAGAGAATTTTATATTAGTTGGGACACGGATTACGTGGGGTCTATCACTACGGCTGATCTTGTGGTATCGGCATCTGCTATTAACTT |  | [This study (gallardo@inia.es)](http://wwwnc.cdc.gov/eid/article/20/9/14-0554_article) | AGTGCGTATACTTGTGCAAGCACTTGTGCAGATACCAATGTAGACACCTGTGCAAGCACTTGTGCAAGCACTTGTGCAAGCACTTGTGCAAGCACAGGTGCAAGCACTTGTGCAGATACCAATGTAGACACCTGTGCAAGCACTTGTGCAAGCACTTGTGCAAGCACTTGTGCAAGCACTTGTGCAAGCACTTGTGCAAGCACAGGTGCAAGCACTTGTGCAGATACCAATGTAGACACCTGTGCAAGCACTTGTGCAAGCACTTGTGCAAGCACTTGTGCAAGCACTTGTGCAAGCACTTGTGCAAGCACTTGTGCAAGCACAGGTGCAAGCACTTGTGCAGATACCAATGTAGACACCTGTGCAAGCACCTGTGCAAACACCTGTGCAAGCACAGAATAC |
| 253 | BF10/SamP13 | Burkina Faso | DP | 2010 |  | I | [This study (gallardo@inia.es)](http://wwwnc.cdc.gov/eid/article/20/9/14-0554_article) | ATGCAGCCTACTCACCACGCAGAGATAAGCTTTCAGGATAGAGATACAGCTCTTCCAGACGCATGTTCATCTATATCGGATATTAGCCCCGTTACGTATCCGATCACATTACCTATTATTAAAAACATTTCCGTAACTGCTCATGGTATCAATCTTATCGATAAGTTTCCATCAAAGTTCTGCAGCTCTTACATACCCTTCCACTACGGAGGCAATGCAATTAAAACCCCCGATGATCCGGGTGCGATGATGATTACCTTTGCTTTGAAGCCACGGGAGGAATACCAACCCAGTGGTCATATTAACGTATCCAGAGCAAGAGAATTTTATATTAGTTGGGACACGGATTACGTGGGGTCTATCACTACGGCTGATCTTGTGGTATCGGCATCTGCTATTAACTT |  | [This study (gallardo@inia.es)](http://wwwnc.cdc.gov/eid/article/20/9/14-0554_article) | AGTGCGTATACTTGTGCAAGCACTTGTGCAGATACCAATGTAGACACCTGTGCAAGCACTTGTGCAAGCACTTGTGCAAGCACTTGTGCAAGCACAGGTGCAAGCACTTGTGCAGATACCAATGTAGACACCTGTGCAAGCACTTGTGCAAGCACTTGTGCAAGCACTTGTGCAAGCACTTGTGCAAGCACTTGTGCAAGCACAGGTGCAAGCACTTGTGCAGATACCAATGTAGACACCTGTGCAAGCACTTGTGCAAGCACTTGTGCAAGCACTTGTGCAAGCACTTGTGCAAGCACTTGTGCAAGCACTTGTGCAAGCACAGGTGCAAGCACTTGTGCAGATACCAATGTAGACACCTGTGCAAGCACCTGTGCAAACACCTGTGCAAGCACAGAATAC |
| 254 | Ug10.Adjumani | Uganda | DP | 2010 | KC990894 | IX | [Atuhaire DK et al 2013](http://wwwnc.cdc.gov/eid/article/20/9/14-0554_article) | ATGCAGCCTACCCACCACGCAGAGGTAAGCTTTCAGGATAGAGATACAGCTCTTCCAGATGCATGTTCATCCATATCTGATATTACCCCCATTACTTATCCGATCACATTACCTATTATTAAAAACATTTCCGTTACTGCTCACGGTATCAATCTTATCGATAAATTTCCATCAAAGTTCTGCAGCTCTTACATACCCTTCCACTACGGAGGCAATTCGATTAAAACCCCCGACGATCCGGGCGCGATGATGATTACCTTTGCTTTGAAACCACGGGAGGAATACCAACCCAGCGGTCATATTAACGTATCCAGAGCAAGAGAATTTTATATTAGCTGGGACACAGATTATGTGGGGTCTATCACCACGGCTGATCTTGTGGTATCGGCATCCGCTATTAACTT | KC990860 | [Atuhaire DK et al 2013](http://wwwnc.cdc.gov/eid/article/20/9/14-0554_article) | AGTGCGTATACCTGTGCAAGCACTTGTGCAAGCACTTGTGCAAGCACTTGTGCAAGCACTTGTGCAGACACCAATGTAGACACTTGTGCAAGCACTTGTGCAGACACTTGTGCAGACACCAATGTAGACACTTGTGCAAGCACTTGTGCAGATACTTGTGCAGACACTTGTGTAAGCACTTGTGTAAGCACTTGTGCAGATACTTGTGCAGACACCAATGTAGACACTTGTGCAAGCACTTGTGCAGACACCAATGTAGACACTTGTGTAAGCACTTGTGCAGACACCTGTGCAAGCACAGAATACCTTTACACATCATGCAAAAAACATTAAATGTAC |
| 255 | Ug10.Moyo1 | Uganda | DP | 2010 | KC990897 | IX | [Atuhaire DK et al 2013](http://wwwnc.cdc.gov/eid/article/20/9/14-0554_article) | ATGCAGCCTACCCACCACGCAGAGGTAAGCTTTCAGGATAGAGATACAGCTCTTCCAGATGCATGTTCATCCATATCTGATATTACCCCCATTACTTATCCGATCACATTACCTATTATTAAAAACATTTCCGTTACTGCTCACGGTATCAATCTTATCGATAAATTTCCATCAAAGTTCTGCAGCTCTTACATACCCTTCCACTACGGAGGCAATTCGATTAAAACCCCCGACGATCCGGGCGCGATGATGATTACCTTTGCTTTGAAACCACGGGAGGAATACCAACCCAGCGGTCATATTAACGTATCCAGAGCAAGAGAATTTTATATTAGCTGGGACACAGATTATGTGGGGTCTATCACCACGGCTGATCTTGTGGTATCGGCATCCGCTATTAACTT | KC990863 | [Atuhaire DK et al 2013](http://wwwnc.cdc.gov/eid/article/20/9/14-0554_article) | AGTGCGTATACCTGTGCAAGCACTTGTGCAAGCACTTGTGCAAGCACTTGTGCAAGCACTTGTGCAGACACCAATGTAGACACTTGTGCAAGCACTTGTGCAGACACTTGTGCAGACACCAATGTAGACACTTGTGCAAGCACTTGTGCAGATACTTGTGCAGACACTTGTGTAAGCACTTGTGTAAGCACTTGTGCAGATACTTGTGCAGACACCAATGTAGACACTTGTGCAAGCACTTGTGCAGACACCAATGTAGACACTTGTGTAAGCACTTGTGCAGACACCTGTGCAAGCACAGAATACCTTTACACATCATGCAAAAAACATTAAATGTAC |
| 256 | Ug10.Moyo2 | Uganda | DP | 2010 | KC990898 | IX | [Atuhaire DK et al 2013](http://wwwnc.cdc.gov/eid/article/20/9/14-0554_article) | ATGCAGCCTACCCACCACGCAGAGGTAAGCTTTCAGGATAGAGATACAGCTCTTCCAGATGCATGTTCATCCATATCTGATATTACCCCCATTACTTATCCGATCACATTACCTATTATTAAAAACATTTCCGTTACTGCTCACGGTATCAATCTTATCGATAAATTTCCATCAAAGTTCTGCAGCTCTTACATACCCTTCCACTACGGAGGCAATTCGATTAAAACCCCCGACGATCCGGGCGCGATGATGATTACCTTTGCTTTGAAACCACGGGAGGAATACCAACCCAGCGGTCATATTAACGTATCCAGAGCAAGAGAATTTTATATTAGCTGGGACACAGATTATGTGGGGTCTATCACCACGGCTGATCTTGTGGTATCGGCATCCGCTATTAACTT | KC990864 | [Atuhaire DK et al 2013](http://wwwnc.cdc.gov/eid/article/20/9/14-0554_article) | AGTGCGTATACCTGTGCAAGCACTTGTGCAAGCACTTGTGCAAGCACTTGTGCAGACACCAATGTAGACACTTGTGCAAGCACTTGTGCAGACATTTGTGCAGACACCAATGTAGACACTTGTGCAAGCACTTGTGCAGATACTTGTGCAGACACTTGTGTAAGCACTTGTGTAAGCACTTGTGCAGATACTTGTGCAGACACCAATGTAGACACTTGTGCAAGCACTTGTGCAGACACCAATGTAGACACTTGTGTAAGCACTTGTGCAGACACCTGTGCAAGCACAGAATACCTTTACACATCATGCAAAAAACATTAAATGTAC |
| 257 | Ug10.Tororo | Uganda | DP | 2010 | KC990896 | IX | [Atuhaire DK et al 2013](http://wwwnc.cdc.gov/eid/article/20/9/14-0554_article) | ATGCAGCCTACCCACCACGCAGAGGTAAGCTTTCAGGATAGAGATACAGCTCTTCCAGATGCATGTTCATCCATATCTGATATTACCCCCATTACTTATCCGATCACATTACCTATTATTAAAAACATTTCCGTTACTGCTCACGGTATCAATCTTATCGATAAATTTCCATCAAAGTTCTGCAGCTCTTACATACCCTTCCACTACGGAGGCAATTCGATTAAAACCCCCGACGATCCGGGCGCGATGATGATTACCTTTGCTTTGAAACCACGGGAGGAATACCAACCCAGCGGTCATATTAACGTATCCAGAGCAAGAGAATTTTATATTAGCTGGGACACAGATTATGTGGGGTCTATCACCACGGCTGATCTTGTGGTATCGGCATCCGGCATTAACTT | KC990862 | [Atuhaire DK et al 2013](http://wwwnc.cdc.gov/eid/article/20/9/14-0554_article) | AGTGCGTATACCTGTGCAAGCACTTGTGCAAGCACTTGTGCAAGCACTTGTGCAGACACCAATGTAGACACTTGTGCAAGCACTTGTGCAGACATTTGTGCAGACACCAATGTAGACACTTGTGCAAGCACTTGTGCAGATACTTGTGCAGACACTTGTGTAAGCACTTGTGTAAGCACTTGTGCAGATACTTGTGCAGACACCAATGTAGACACTTGTGCAAGCACTTGTGCAGACACCAATGTAGACACTTGTGTAAGCACTTGTGCAGACACCTGTGCAAGCACAGAATACCTTTACACATCATGCAAAAAACATTAAATGTAC |
| 258 | Ug10.Kumi | Uganda | DP | 2010 | KC990892 | IX | [Atuhaire DK et al 2013](http://wwwnc.cdc.gov/eid/article/20/9/14-0554_article) | ATGCAGCCTACCCACCACGCAGAGGTAAGCTTTCAGGATAGAGATACAGCTCTTCCAGATGCATGTTCATCCATATCTGATATTACCCCCATTACTTATCCGATCACATTACCTATTATTAAAAACATTTCCGTTACTGCTCACGGTATCAATCTTATCGATAAATTTCCATCAAAGTTCTGCAGCTCTTACATACCCTTCCACTACGGAGGCAATTCGATTAAAACCCCCGACGATCCGGGCGCGATGATGATTACCTTTGCTTTGAAACCACGGGAGGAATACCAACCCAGCGGTCATATTAACGTATCCAGAGCAAGAGAATTTTATATTAGCTGGGACACAGATTATGTGGGGTCTATCACCACGGCTGATCTTGTGGTATCGGCATCCGCTATTAACTT | KC990858 | [Atuhaire DK et al 2013](http://wwwnc.cdc.gov/eid/article/20/9/14-0554_article) | AGTGCGTATACCTGTGCAAGCACTTGTGCAAGCACTTGTGCAAGCACTTGTGCAGACACCAATGTAGACACTTGTGCAAGCACTTGTGCAGACATTTGTGCAGACACCAATGTAGACACTTGTGCAAGCACTTGTGCAGATACTTGTGCAGACACTTGTGTAAGCACTTGTGTAAGCACTTGTGCAGATACTTGTGCAGACACCAATGTAGACACTTGTGCAAGCACTTGTGCAGACACCAATGTAGACACTTGTGTAAGCACTTGTGCAGACACCTGTGCAAGCACAGAATACCTTTACACATCATGCAAAAAACATTAAATGTAC |
| 259 | Ken10/KakFA1 | Kenya | DP | 2010 | KC112561 | IX | [This study (gallardo@inia.es)](http://www.ars.usda.gov/GARA/publications/Atuhaire%20et%20al%202013%20ASF.pdf) | ATGCAGCCTACCCACCACGCAGAGGTAAGCTTTCAGGATAGAGATACAGCTCTTCCAGATGCATGTTCATCCATATCTGATATTACCCCCATTACTTATCCGATCACATTACCTATTATTAAAAACATTTCCGTTACTGCTCACGGTATCAATCTTATCGATAAATTTCCATCAAAGTTCTGCAGCTCTTACATACCCTTCCACTACGGAGGCAATTCGATTAAAACCCCCGACGATCCGGGCGCGATGATGATTACCTTTGCTTTGAAACCACGGGAGGAATACCAACCCAGCGGTCATATTAACGTATCCAGAGCAAGAGAATTTTATATTAGCTGGGACACAGATTATGTGGGGTCTATCACCACGGCTGATCTTGTGGTATCGGCATCCGCTATTAACTT |  | [This study (gallardo@inia.es)](http://asf-referencelab.info/asf/images/files/publicaciones/Nix_et_al_2006.pdf) | AGTGCGTATACCTGTGCAAGCACTTGTGCAAGCACTTGTGCAAGCACTTGTGCAGACACCAATGTAGACACTTGTGCAAGCACTTGTGCAGACATTTGTGCAGACACCAATGTAGACACTTGTGCAAGCACTTGTGCAGATACTTGTGCAGACACTTGTGTAAGCACTTGTGTAAGCACTTGTGCAGATACTTGTGCAGACACCAATGTAGACACTTGTGCAAGCACTTGTGCAGACACCAATGTAGACACTTGTGTAAGCACTTGTGCAGACACCTGTGCAAGCACAGAATACCCTGAGCGCATT |
| 260 | Ken10/Kis027 | Kenya | DP | 2010 | KC112562 | IX | [This study (gallardo@inia.es)](http://www.ars.usda.gov/GARA/publications/Atuhaire%20et%20al%202013%20ASF.pdf) | ATGCAGCCTACCCACCACGCAGAGGTAAGCTTTCAGGATAGAGATACAGCTCTTCCAGATGCATGTTCATCCATATCTGATATTACCCCCATTACTTATCCGATCACATTACCTATTATTAAAAACATTTCCGTTACTGCTCACGGTATCAATCTTATCGATAAATTTCCATCAAAGTTCTGCAGCTCTTACATACCCTTCCACTACGGAGGCAATTCGATTAAAACCCCCGACGATCCGGGCGCGATGATGATTACCTTTGCTTTGAAACCACGGGAGGAATACCAACCCAGCGGTCATATTAACGTATCCAGAGCAAGAGAATTTTATATTAGCTGGGACACAGATTATGTGGGGTCTATCACCACGGCTGATCTTGTGGTATCGGCATCCGCTATTAACTT |  | [This study (gallardo@inia.es)](http://asf-referencelab.info/asf/images/files/publicaciones/Nix_et_al_2006.pdf) | AGTGCGTATACCTGTGCAAGCACTTGTGCAAGCACTTGTGCAAGCACTTGTGCAGACACCAATGTAGACACTTGTGCAAGCACTTGTGCAGACATTTGTGCAGACACCAATGTAGACACTTGTGCAAGCACTTGTGCAGATACTTGTGCAGACACTTGTGTAAGCACTTGTGTAAGCACTTGTGCAGATACTTGTGCAGACACCAATGTAGACACTTGTGCAAGCACTTGTGCAGACACCAATGTAGACACTTGTGTAAGCACTTGTGCAGACACCTGTGCAAGCACAGAATAC |
| 261 | Ken10/Kis028 | Kenya | DP | 2010 | KC112563 | IX | [This study (gallardo@inia.es)](http://www.ars.usda.gov/GARA/publications/Atuhaire%20et%20al%202013%20ASF.pdf) | ATGCAGCCTACCCACCACGCAGAGGTAAGCTTTCAGGATAGAGATACAGCTCTTCCAGATGCATGTTCATCCATATCTGATATTACCCCCATTACTTATCCGATCACATTACCTATTATTAAAAACATTTCCGTTACTGCTCACGGTATCAATCTTATCGATAAATTTCCATCAAAGTTCTGCAGCTCTTACATACCCTTCCACTACGGAGGCAATTCGATTAAAACCCCCGACGATCCGGGCGCGATGATGATTACCTTTGCTTTGAAACCACGGGAGGAATACCAACCCAGCGGTCATATTAACGTATCCAGAGCAAGAGAATTTTATATTAGCTGGGACACAGATTATGTGGGGTCTATCACCACGGCTGATCTTGTGGTATCGGCATCCGCTATTAACTT |  | [This study (gallardo@inia.es)](http://asf-referencelab.info/asf/images/files/publicaciones/Nix_et_al_2006.pdf) | AGTGCGTATACCTGTGCAAGCACTTGTGCAAGCACTTGTGCAAGCACTTGTGCAGACACCAATGTAGACACTTGTGCAAGCACTTGTGCAGACATTTGTGCAGACACCAATGTAGACACTTGTGCAAGCACTTGTGCAGATACTTGTGCAGACACTTGTGTAAGCACTTGTGTAAGCACTTGTGCAGATACTTGTGCAGACACCAATGTAGACACTTGTGCAAGCACTTGTGCAGACACCAATGTAGACACTTGTGTAAGCACTTGTGCAGACACCTGTGCAAGCACAGAATAC |
| 262 | Ken11/Bus1.2 | Kenya | DP | 2011 | KC112564 | IX | [This study (gallardo@inia.es)](http://www.ars.usda.gov/GARA/publications/Atuhaire%20et%20al%202013%20ASF.pdf) | ATGCAGCCTACCCACCACGCAGAGGTAAGCTTTCAGGATAGAGATACAGCTCTTCCAGATGCATGTTCATCCATATCTGATATTACCCCCATTACTTATCCGATCACATTACCTATTATTAAAAACATTTCCGTTACTGCTCACGGTATCAATCTTATCGATAAATTTCCATCAAAGTTCTGCAGCTCTTACATACCCTTCCACTACGGAGGCAATTCGATTAAAACCCCCGACGATCCGGGCGCGATGATGATTACCTTTGCTTTGAAACCACGGGAGGAATACCAACCCAGCGGTCATATTAACGTATCCAGAGCAAGAGAATTTTATATTAGCTGGGACACAGATTATGTGGGGTCTATCACCACGGCTGATCTTGTGGTATCGGCATCCGCTATTAACTT |  | [This study (gallardo@inia.es)](http://www.ars.usda.gov/GARA/publications/Atuhaire%20et%20al%202013%20ASF.pdf) | AGTGCGTATACCTGTGCAAGCACTTGTGCAAGCACTTGTGCAAGCACTTGTGCAGACACCAATGTAGACACTTGTGCAAGCACTTGTGCAGACATTTGTGCAGACACCAATGTAGACACTTGTGCAAGCACTTGTGCAGATACTTGTGCAGACACTTGTGTAAGCACTTGTGTAAGCACTTGTGCAGATACTTGTGCAGACACCAATGTAGACACTTGTGCAAGCACTTGTGCAGACACCAATGTAGACACTTGTGTAAGCACTTGTGCAGACACCTGTGCAAGCACAGAATAC |
| 263 | Ken11/Kia2.1 | Kenya | DP | 2011 | KC112565 | IX | [This study (gallardo@inia.es)](http://www.ars.usda.gov/GARA/publications/Atuhaire%20et%20al%202013%20ASF.pdf) | ATGCAGCCTACCCACCACGCAGAGGTAAGCTTTCAGGATAGAGATACAGCTCTTCCAGATGCATGTTCATCCATATCTGATATTACCCCCATTACTTATCCGATCACATTACCTATTATTAAAAACATTTCCGTTACTGCTCACGGTATCAATCTTATCGATAAATTTCCATCAAAGTTCTGCAGCTCTTACATACCCTTCCACTACGGAGGCAATTCGATTAAAACCCCCGACGATCCGGGCGCGATGATGATTACCTTTGCTTTGAAACCACGGGAGGAATACCAACCCAGCGGTCATATTAACGTATCCAGAGCAAGAGAATTTTATATTAGCTGGGACACAGATTATGTGGGGTCTATCACCACGGCTGATCTTGTGGTATCGGCATCCGCTATTAACTT |  | [This study (gallardo@inia.es)](http://www.ars.usda.gov/GARA/publications/Atuhaire%20et%20al%202013%20ASF.pdf) | AGTGCGTATACCTGTGCAAGCACTTGTGCAAGCACTTGTGCAAGCACTTGTGCAGACACCAATGTAGACACTTGTGCAAGCACTTGTGCAGACATTTGTGCAGACACCAATGTAGACACTTGTGCAAGCACTTGTGCAGATACTTGTGCAGACACTTGTGTAAGCACTTGTGTAAGCACTTGTGCAGATACTTGTGCAGACACCAATGTAGACACTTGTGCAAGCACTTGTGCAGACACCAATGTAGACACTTGTGTAAGCACTTGTGCAGACACCTGTGCAAGCACAGAATAC |
| 264 | Ken11/ThikP06 | Kenya | DP | 2011 | KC112566 | IX | [This study (gallardo@inia.es)](http://www.ars.usda.gov/GARA/publications/Atuhaire%20et%20al%202013%20ASF.pdf) | ATGCAGCCTACCCACCACGCAGAGGTAAGCTTTCAGGATAGAGATACAGCTCTTCCAGATGCATGTTCATCCATATCTGATATTACCCCCATTACTTATCCGATCACATTACCTATTATTAAAAACATTTCCGTTACTGCTCACGGTATCAATCTTATCGATAAATTTCCATCAAAGTTCTGCAGCTCTTACATACCCTTCCACTACGGAGGCAATTCGATTAAAACCCCCGACGATCCGGGCGCGATGATGATTACCTTTGCTTTGAAACCACGGGAGGAATACCAACCCAGCGGTCATATTAACGTATCCAGAGCAAGAGAATTTTATATTAGCTGGGACACAGATTATGTGGGGTCTATCACCACGGCTGATCTTGTGGTATCGGCATCCGCTATTAACTT |  | [This study (gallardo@inia.es)](http://www.ars.usda.gov/GARA/publications/Atuhaire%20et%20al%202013%20ASF.pdf) | AGTGCGTATACCTGTGCAAGCACTTGTGCAAGCACTTGTGCAAGCACTTGTGCAGACACCAATGTAGACACTTGTGCAAGCACTTGTGCAGACATTTGTGCAGACACCAATGTAGACACTTGTGCAAGCACTTGTGCAGATACTTGTGCAGACACTTGTGTAAGCACTTGTGTAAGCACTTGTGCAGATACTTGTGCAGACACCAATGTAGACACTTGTGCAAGCACTTGTGCAGACACCAATGTAGACACTTGTGTAAGCACTTGTGCAGACACCTGTGCAAGCACAGAATAC |
| 265 | Ken11/KakSP | Kenya | DP | 2011 | KC112567 | IX | [This study (gallardo@inia.es)](http://www.ars.usda.gov/GARA/publications/Atuhaire%20et%20al%202013%20ASF.pdf) | ATGCAGCCTACCCACCACGCAGAGGTAAGCTTTCAGGATAGAGATACAGCTCTTCCAGATGCATGTTCATCCATATCTGATATTACCCCCATTACTTATCCGATCACATTACCTATTATTAAAAACATTTCCGTTACTGCTCACGGTATCAATCTTATCGATAAATTTCCATCAAAGTTCTGCAGCTCTTACATACCCTTCCACTACGGAGGCAATTCGATTAAAACCCCCGACGATCCGGGCGCGATGATGATTACCTTTGCTTTGAAACCACGGGAGGAATACCAACCCAGCGGTCATATTAACGTATCCAGAGCAAGAGAATTTTATATTAGCTGGGACACAGATTATGTGGGGTCTATCACCACGGCTGATCTTGTGGTATCGGCATCCGCTATTAACTT |  | [This study (gallardo@inia.es)](http://www.ars.usda.gov/GARA/publications/Atuhaire%20et%20al%202013%20ASF.pdf) | AGTGCGTATACCTGTGCAAGCACTTGTGCAAGCACTTGTGCAAGCACTTGTGCAGACACCAATGTAGACACTTGTGCAAGCACTTGTGCAGACATTTGTGCAGACACCAATGTAGACACTTGTGCAAGCACTTGTGCAGATACTTGTGCAGACACTTGTGTAAGCACTTGTGTAAGCACTTGTGCAGATACTTGTGCAGACACCAATGTAGACACTTGTGCAAGCACTTGTGCAGACACCAATGTAGACACTTGTGTAAGCACTTGTGCAGACACCTGTGCAAGCACAGAATAC |
| 266 | Ug11.Kampala2 | Uganda | DP | 2011 | KC990893 | IX | [Atuhaire DK et al 2013](http://www.ars.usda.gov/GARA/publications/Atuhaire%20et%20al%202013%20ASF.pdf) | ATGCAGCCTACCCACCACGCAGAGGTAAGCTTTCAGGATAGAGATACAGCTCTTCCAGATGCATGTTCATCCATATCTGATATTACCCCCATTACTTATCCGATCACATTACCTATTATTAAAAACATTTCCGTTACTGCTCACGGTATCAATCTTATCGATAAATTTCCATCAAAGTTCTGCAGCTCTTACATACCCTTCCACTACGGAGGCAATTCGATTAAAACCCCCGACGATCCGGGCGCGATGATGATTACCTTTGCTTTGAAACCACGGGAGGAATACCAACCCAGCGGTCATATTAACGTATCCAGAGCAAGAGAATTTTATATTAGCTGGGACACAGATTATGTGGGGTCTATCACCACGGCTGATCTTGTGGTATCGGCATCCGCTATTAACTT | KC990859 | [Atuhaire DK et al 2013](http://www.ars.usda.gov/GARA/publications/Atuhaire%20et%20al%202013%20ASF.pdf) | AGTGCGTATACCTGTGCAAGCACTTGTGCAAGCACTTGTGCAAGCACTTGTGCAGACACCAATGTAGACACTTGTGCAAGCACTTGTGCAGACATTTGTGCAGACACCAATGTAGACACTTGTGCAAGCACTTGTGCAGATACTTGTGCAGACACTTGTGTAAGCACTTGTGTAAGCACTTGTGCAGATACTTGTGCAGACACCAATGTAGACACTTGTGCAAGCACTTGTGCAGACACCAATGTAGACACTTGTGTAAGCACTTGTGCAGACACCTGTGCAAGCACAGAATACCTTTACACATCATGCAAAAAACATTAAATGTAC |
| 267 | Tver0511/Torjo | Russia | DP | 2011 | KJ627208 | II | [Gallardo et al 2014](http://www.ars.usda.gov/GARA/publications/Atuhaire%20et%20al%202013%20ASF.pdf) | ATGCAGCCCACTCACCACGCAGAGATAAGCTTTCAGGATAGAGATACAGCTCTTCCAGACGCATGTTCATCTATATCTGATATTAGCCCCGTTACGTATCCGATCACATTACCTATTATTAAAAACATTTCCGTAACTGCTCATGGTATCAATCTTATCGATAAATTTCCATCAAAGTTCTGCAGCTCTTACATACCCTTCCACTACGGAGGCAATGCGATTAAAACCCCCGATGATCCGGGTGCGATGATGATTACCTTTGCTTTGAAGCCACGGGAGGAATACCAACCCAGTGGTCATATTAACGTATCCAGAGCAAGAGAATTTTATATTAGTTGGGACACGGATTACGTGGGGTCTATCACTACGGCTGATCTTGTGGTATCGGCATCTGCTATTAACTT | KJ627197 | [Gallardo et al 2014](http://www.ars.usda.gov/GARA/publications/Atuhaire%20et%20al%202013%20ASF.pdf) | AGTGCGTATACTTGTGCAGATACCAATGTAGACACTTGTGCAAGCATGTGTGCAGATACCAATGTAGACACCTGTGCAAGCATGTGTGCAGATACCAATGTAGATACCTGTGCAAGCACTTGTACAAGCACAGAATAC |
| 268 | Ken11/KerP27 | Kenya | DP | 2011 |  | IX | [This study (gallardo@inia.es)](http://www.ars.usda.gov/GARA/publications/Atuhaire%20et%20al%202013%20ASF.pdf) | ATGCAGCCTACCCACCACGCAGAGGTAAGCTTTCAGGATAGAGATACAGCTCTTCCAGATGCATGTTCATCCATATCTGATATTACCCCCATTACTTATCCGATCACATTACCTATTATTAAAAACATTTCCGTTACTGCTCACGGTATCAATCTTATCGATAAATTTCCATCAAAGTTCTGCAGCTCTTACATACCCTTCCACTACGGAGGCAATTCGATTAAAACCCCCGACGATCCGGGCGCGATGATGATTACCTTTGCTTTGAAACCACGGGAGGAATACCAACCCAGCGGTCATATTAACGTATCCAGAGCAAGAGAATTTTATATTAGCTGGGACACAGATTATGTGGGGTCTATCACCACGGCTGATCTTGTGGTATCGGCATCCGCTATTAACTT |  | [This study (gallardo@inia.es)](http://www.ars.usda.gov/GARA/publications/Atuhaire%20et%20al%202013%20ASF.pdf) | AGTGCGTATACCTGTGCAAGCACTTGTGCAAGCACTTGTGCAAGCACTTGTGCAGACACCAATGTAGACACTTGTGCAAGCACTTGTGCAGACATTTGTGCAGACACCAATGTAGACACTTGTGCAAGCACTTGTGCAGATACTTGTGCAGACACTTGTGTAAGCACTTGTGTAAGCACTTGTGCAGATACTTGTGCAGACACCAATGTAGACACTTGTGCAAGCACTTGTGCAGACACCAATGTAGACACTTGTGTAAGCACTTGTGCAGACACCTGTGCAAGCACAGAATAC |
| 269 | Ken11/KiaP31 | Kenya | DP | 2011 |  | IX | [This study (gallardo@inia.es)](http://www.ars.usda.gov/GARA/publications/Atuhaire%20et%20al%202013%20ASF.pdf) | ATGCAGCCTACCCACCACGCAGAGGTAAGCTTTCAGGATAGAGATACAGCTCTTCCAGATGCATGTTCATCCATATCTGATATTACCCCCATTACTTATCCGATCACATTACCTATTATTAAAAACATTTCCGTTACTGCTCACGGTATCAATCTTATCGATAAATTTCCATCAAAGTTCTGCAGCTCTTACATACCCTTCCACTACGGAGGCAATTCGATTAAAACCCCCGACGATCCGGGCGCGATGATGATTACCTTTGCTTTGAAACCACGGGAGGAATACCAACCCAGCGGTCATATTAACGTATCCAGAGCAAGAGAATTTTATATTAGCTGGGACACAGATTATGTGGGGTCTATCACCACGGCTGATCTTGTGGTATCGGCATCCGCTATTAACTT |  | [This study (gallardo@inia.es)](http://www.ars.usda.gov/GARA/publications/Atuhaire%20et%20al%202013%20ASF.pdf) | AGTGCGTATACCTGTGCAAGCACTTGTGCAAGCACTTGTGCAAGCACTTGTGCAGACACCAATGTAGACACTTGTGCAAGCACTTGTGCAGACATTTGTGCAGACACCAATGTAGACACTTGTGCAAGCACTTGTGCAGATACTTGTGCAGACACTTGTGTAAGCACTTGTGTAAGCACTTGTGCAGATACTTGTGCAGACACCAATGTAGACACTTGTGCAAGCACTTGTGCAGACACCAATGTAGACACTTGTGTAAGCACTTGTGCAGACACCTGTGCAAGCACAGAATAC |
| 270 | Ken11/KisP52 | Kenya | DP | 2011 |  | IX | [This study (gallardo@inia.es)](http://www.ars.usda.gov/GARA/publications/Atuhaire%20et%20al%202013%20ASF.pdf) | ATGCAGCCTACCCACCACGCAGAGGTAAGCTTTCAGGATAGAGATACAGCTCTTCCAGATGCATGTTCATCCATATCTGATATTACCCCCATTACTTATCCGATCACATTACCTATTATTAAAAACATTTCCGTTACTGCTCACGGTATCAATCTTATCGATAAATTTCCATCAAAGTTCTGCAGCTCTTACATACCCTTCCACTACGGAGGCAATTCGATTAAAACCCCCGACGATCCGGGCGCGATGATGATTACCTTTGCTTTGAAACCACGGGAGGAATACCAACCCAGCGGTCATATTAACGTATCCAGAGCAAGAGAATTTTATATTAGCTGGGACACAGATTATGTGGGGTCTATCACCACGGCTGATCTTGTGGTATCGGCATCCGCTATTAACTT |  | [This study (gallardo@inia.es)](http://www.ars.usda.gov/GARA/publications/Atuhaire%20et%20al%202013%20ASF.pdf) | AGTGCGTATACCTGTGCAAGCACTTGTGCAAGCACTTGTGCAAGCACTTGTGCAGACACCAATGTAGACACTTGTGCAAGCACTTGTGCAGACATTTGTGCAGACACCAATGTAGACACTTGTGCAAGCACTTGTGCAGATACTTGTGCAGACACTTGTGTAAGCACTTGTGTAAGCACTTGTGCAGATACTTGTGCAGACACCAATGTAGACACTTGTGCAAGCACTTGTGCAGACACCAATGTAGACACTTGTGTAAGCACTTGTGCAGACACCTGTGCAAGCACAGAATAC |
| 271 | Ken11/NakP29 | Kenya | DP | 2011 |  | IX | [This study (gallardo@inia.es)](http://www.ars.usda.gov/GARA/publications/Atuhaire%20et%20al%202013%20ASF.pdf) | ATGCAGCCTACCCACCACGCAGAGGTAAGCTTTCAGGATAGAGATACAGCTCTTCCAGATGCATGTTCATCCATATCTGATATTACCCCCATTACTTATCCGATCACATTACCTATTATTAAAAACATTTCCGTTACTGCTCACGGTATCAATCTTATCGATAAATTTCCATCAAAGTTCTGCAGCTCTTACATACCCTTCCACTACGGAGGCAATTCGATTAAAACCCCCGACGATCCGGGCGCGATGATGATTACCTTTGCTTTGAAACCACGGGAGGAATACCAACCCAGCGGTCATATTAACGTATCCAGAGCAAGAGAATTTTATATTAGCTGGGACACAGATTATGTGGGGTCTATCACCACGGCTGATCTTGTGGTATCGGCATCCGCTATTAACTT |  | [This study (gallardo@inia.es)](http://www.ars.usda.gov/GARA/publications/Atuhaire%20et%20al%202013%20ASF.pdf) | AGTGCGTATACCTGTGCAAGCACTTGTGCAAGCACTTGTGCAAGCACTTGTGCAGACACCAATGTAGACACTTGTGCAAGCACTTGTGCAGACATTTGTGCAGACACCAATGTAGACACTTGTGCAAGCACTTGTGCAGATACTTGTGCAGACACTTGTGTAAGCACTTGTGTAAGCACTTGTGCAGATACTTGTGCAGACACCAATGTAGACACTTGTGCAAGCACTTGTGCAGACACCAATGTAGACACTTGTGTAAGCACTTGTGCAGACACCTGTGCAAGCACAGAATAC |
| 272 | Ken11/Thikp49 | Kenya | DP | 2011 |  | IX | [This study (gallardo@inia.es)](http://www.ars.usda.gov/GARA/publications/Atuhaire%20et%20al%202013%20ASF.pdf) | ATGCAGCCTACCCACCACGCAGAGGTAAGCTTTCAGGATAGAGATACAGCTCTTCCAGATGCATGTTCATCCATATCTGATATTACCCCCATTACTTATCCGATCACATTACCTATTATTAAAAACATTTCCGTTACTGCTCACGGTATCAATCTTATCGATAAATTTCCATCAAAGTTCTGCAGCTCTTACATACCCTTCCACTACGGAGGCAATTCGATTAAAACCCCCGACGATCCGGGCGCGATGATGATTACCTTTGCTTTGAAACCACGGGAGGAATACCAACCCAGCGGTCATATTAACGTATCCAGAGCAAGAGAATTTTATATTAGCTGGGACACAGATTATGTGGGGTCTATCACCACGGCTGATCTTGTGGTATCGGCATCCGCTATTAACTT |  | [This study (gallardo@inia.es)](http://www.ars.usda.gov/GARA/publications/Atuhaire%20et%20al%202013%20ASF.pdf) | AGTGCGTATACCTGTGCAAGCACTTGTGCAAGCACTTGTGCAAGCACTTGTGCAGACACCAATGTAGACACTTGTGCAAGCACTTGTGCAGACATTTGTGCAGACACCAATGTAGACACTTGTGCAAGCACTTGTGCAGATACTTGTGCAGACACTTGTGTAAGCACTTGTGTAAGCACTTGTGCAGATACTTGTGCAGACACCAATGTAGACACTTGTGCAAGCACTTGTGCAGACACCAATGTAGACACTTGTGTAAGCACTTGTGCAGACACCTGTGCAAGCACAGAATAC |
| 273 | Ug11.Mpigi | Uganda | DP | 2011 | KC990895 | IX | [Atuhaire DK et al 2013](http://www.ars.usda.gov/GARA/publications/Atuhaire%20et%20al%202013%20ASF.pdf) | ATGCAGCCTACCCACCACGCAGAGGTAAGCTTTCAGGATAGAGATACAGCTCTTCCAGATGCATGTTCATCCATATCTGATATTACCCCCATTACTTATCCGATCACATTACCTATTATTAAAAACATTTCCGTTACTGCTCACGGTATCAATCTTATCGATAAATTTCCATCAAAGTTCTGCAGCTCTTACATACCCTTCCACTACGGAGGCAATTCGATTAAAACCCCCGACGATCCGGGCGCGATGATGATTACCTTTGCTTTGAAACCACGGGAGGAATACCAACCCAGCGGTCATATTAACGTATCCAGAGCAAGAGAATTTTATATTAGCTGGGACACAGATTATGTGGGGTCTATCACCACGGCTGATCTTGTGGTATCGGCATCCGCTATTAACTT | KC990861 | [Atuhaire DK et al 2013](http://www.ars.usda.gov/GARA/publications/Atuhaire%20et%20al%202013%20ASF.pdf) | AGTGCGTATACCTGTGCAAGCACTTGTGCAAGCACTTGTGCAAGCACTTGTGCAAGCACTTGTGCAGACACCAATGTAGACACTTGTGCAAGCACTTGTGCAGACACTTGTGCAGACACCAATGTAGACACTTGTGCAAGCACTTGTGCAGATACTTGTGCAGACACTTGTGTAAGCACTTGTGTAAGCACTTGTGCAGATACTTGTGCAGACACCAATGTAGACACTTGTGCAAGCACTTGTGCAGACACCAATGTAGACACTTGTGTAAGCACTTGTGCAGACACCTGTGCAAGCACAGAATACCTTTACACATCATGCAAAAAACATTAAATGTAC |
| 274 | Ken11/Kilifili | Kenya | DP | 2011 |  | IX | [This study (gallardo@inia.es)](http://www.ars.usda.gov/GARA/publications/Atuhaire%20et%20al%202013%20ASF.pdf) | ATGCAGCCTACCCACCACGCAGAGGTAAGCTTTCAGGATAGAGATACAGCTCTTCCAGATGCATGTTCATCCATATCTGATATTACCCCCATTACTTATCCGATCACATTACCTATTATTAAAAACATTTCCGTTACTGCTCACGGTATCAATCTTATCGATAAATTTCCATCAAAGTTCTGCAGCTCTTACATACCCTTCCACTACGGAGGCAATTCGATTAAAACCCCCGACGATCCGGGCGCGATGATGATTACCTTTGCTTTGAAACCACGGGAGGAATACCAACCCAGCGGTCATATTAACGTATCCAGAGCAAGAGAATTTTATATTAGCTGGGACACAGATTATGTGGGGTCTATCACCACGGCTGATCTTGTGGTATCGGCATCCGCTATTAACTT |  | [This study (gallardo@inia.es)](http://www.ars.usda.gov/GARA/publications/Atuhaire%20et%20al%202013%20ASF.pdf) | AGTGCGTATACCTGTGCAAGCACTTGTGCAAGCACTTGTGCAAGCACTTGTGCAGACACCAATGTAGACACTTGTGCAAGCACTTGTGCAGACATTTGTGCAGACACCAATGTAGACACTTGTGCAAGCACTTGTGCAGATACTTGTGCAGACACTTGTGTAAGCACTTGTGTAAGCACTTGTGCAGATACTTGTGCAGACACCAATGTAGACACTTGTGCAAGCACTTGTGCAGACACCAATGTAGACACTTGTGTAAGCACTTGTGCAGACACCTGTGCAAGCACAGAATAC |
| 275 | Ken11/KisP1 | Kenya | DP | 2011 |  | IX | [This study (gallardo@inia.es)](http://www.ars.usda.gov/GARA/publications/Atuhaire%20et%20al%202013%20ASF.pdf) | ATGCAGCCTACCCACCACGCAGAGGTAAGCTTTCAGGATAGAGATACAGCTCTTCCAGATGCATGTTCATCCATATCTGATATTACCCCCATTACTTATCCGATCACATTACCTATTATTAAAAACATTTCCGTTACTGCTCACGGTATCAATCTTATCGATAAATTTCCATCAAAGTTCTGCAGCTCTTACATACCCTTCCACTACGGAGGCAATTCGATTAAAACCCCCGACGATCCGGGCGCGATGATGATTACCTTTGCTTTGAAACCACGGGAGGAATACCAACCCAGCGGTCATATTAACGTATCCAGAGCAAGAGAATTTTATATTAGCTGGGACACAGATTATGTGGGGTCTATCACCACGGCTGATCTTGTGGTATCGGCATCCGCTATTAACTT |  | [This study (gallardo@inia.es)](http://www.ars.usda.gov/GARA/publications/Atuhaire%20et%20al%202013%20ASF.pdf) | AGTGCGTATACCTGTGCAAGCACTTGTGCAAGCACTTGTGCAAGCACTTGTGCAGACACCAATGTAGACACTTGTGCAAGCACTTGTGCAGACATTTGTGCAGACACCAATGTAGACACTTGTGCAAGCACTTGTGCAGATACTTGTGCAGACACTTGTGTAAGCACTTGTGTAAGCACTTGTGCAGATACTTGTGCAGACACCAATGTAGACACTTGTGCAAGCACTTGTGCAGACACCAATGTAGACACTTGTGTAAGCACTTGTGCAGACACCTGTGCAAGCACAGAATAC |
| 276 | Ken11/Bum002 | Kenya | DP | 2011 |  | IX | [This study (gallardo@inia.es)](http://www.ars.usda.gov/GARA/publications/Atuhaire%20et%20al%202013%20ASF.pdf) | ATGCAGCCTACCCACCACGCAGAGGTAAGCTTTCAGGATAGAGATACAGCTCTTCCAGATGCATGTTCATCCATATCTGATATTACCCCCATTACTTATCCGATCACATTACCTATTATTAAAAACATTTCCGTTACTGCTCACGGTATCAATCTTATCGATAAATTTCCATCAAAGTTCTGCAGCTCTTACATACCCTTCCACTACGGAGGCAATTCGATTAAAACCCCCGACGATCCGGGCGCGATGATGATTACCTTTGCTTTGAAACCACGGGAGGAATACCAACCCAGCGGTCATATTAACGTATCCAGAGCAAGAGAATTTTATATTAGCTGGGACACAGATTATGTGGGGTCTATCACCACGGCTGATCTTGTGGTATCGGCATCCGCTATTAACTT |  | [This study (gallardo@inia.es)](http://www.ars.usda.gov/GARA/publications/Atuhaire%20et%20al%202013%20ASF.pdf) | AGTGCGTATACCTGTGCAAGCACTTGTGCAAGCACTTGTGCAAGCACTTGTGCAGACACCAATGTAGACACTTGTGCAAGCACTTGTGCAGACATTTGTGCAGACACCAATGTAGACACTTGTGCAAGCACTTGTGCAGATACTTGTGCAGACACTTGTGTAAGCACTTGTGTAAGCACTTGTGCAGATACTTGTGCAGACACCAATGTAGACACTTGTGCAAGCACTTGTGCAGACACCAATGTAGACACTTGTGTAAGCACTTGTGCAGACACCTGTGCAAGCACAGAATAC |
| 277 | Tver0312/Novo | Russia | DP | 2012 | KJ627212 | II | [Gallardo et al 2014](http://www.ars.usda.gov/GARA/publications/Atuhaire%20et%20al%202013%20ASF.pdf) | ATGCAGCCCACTCACCACGCAGAGATAAGCTTTCAGGATAGAGATACAGCTCTTCCAGACGCATGTTCATCTATATCTGATATTAGCCCCGTTACGTATCCGATCACATTACCTATTATTAAAAACATTTCCGTAACTGCTCATGGTATCAATCTTATCGATAAATTTCCATCAAAGTTCTGCAGCTCTTACATACCCTTCCACTACGGAGGCAATGCGATTAAAACCCCCGATGATCCGGGTGCGATGATGATTACCTTTGCTTTGAAGCCACGGGAGGAATACCAACCCAGTGGTCATATTAACGTATCCAGAGCAAGAGAATTTTATATTAGTTGGGACACGGATTACGTGGGGTCTATCACTACGGCTGATCTTGTGGTATCGGCATCTGCTATTAACTT | KJ627201 | [Gallardo et al 2014](http://www.ars.usda.gov/GARA/publications/Atuhaire%20et%20al%202013%20ASF.pdf) | AGTGCGTATACTTGTGCAGATACCAATGTAGACACTTGTGCAAGCATGTGTGCAGATACCAATGTAGACACCTGTGCAAGCATGTGTGCAGATACCAATGTAGATACCTGTGCAAGCACTTGTACAAGCACAGAATAC |
| 278 | Uga12.Nakasongola | Uganda | DP | 2012 | KF303310 | IX | [Atuhaire DK et al 2013](http://www.ars.usda.gov/GARA/publications/Atuhaire%20et%20al%202013%20ASF.pdf) | ATGCAGCCTACCCACCACGCAGAGGTAAGCTTTCAGGATAGAGATACAGCTCTTCCAGATGCATGTTCATCCGTATCTGATATTACCCCCATTACTTATCCGATCACATTACCTATTATTAAAAACATTTCCGTTACTGCTCACGGTATCAATCTTATCGATAAATTTCCATCAAAGTTCTGCAGCTGTTACATACCCTTCCACTACGGAGGCAATTCGATTAAAACCCCCGACGATCCGGGCGCGATGATGATTACCTTTGCTTTGAAACCACGGGAGGAATACCAACCCAGCGGTCATATTAACGTATCCAGAGCAAGAGAATTTTATATTAGCTGGGACACAGATTATGTGGGGTCTATCACCACGGCTGATCTTGTGGTATCGGCATCCGCTATTAACTT | KF303295 | [Atuhaire DK et al 2013](http://www.ars.usda.gov/GARA/publications/Atuhaire%20et%20al%202013%20ASF.pdf) | AGTGCGTATACCTGTGCAAGCACTTGTGCAAGCACTTGTGCAAGCACTTGTGCAAGCACTTGTGCAGACACCAATGTAGACACTTGTGCAAGCACTTGTGCAGACACTTGTGCAGACACCAATGTAGACACTTGTGTAAGCACTTGTGCAGACACCTGTGCAAGCACAGAATAC |
| 279 | Tver0312/Torjo | Russia | Wild pig | 2012 | KJ627211 | II | [Gallardo et al 2014](http://www.ars.usda.gov/GARA/publications/Atuhaire%20et%20al%202013%20ASF.pdf) | ATGCAGCCCACTCACCACGCAGAGATAAGCTTTCAGGATAGAGATACAGCTCTTCCAGACGCATGTTCATCTATATCTGATATTAGCCCCGTTACGTATCCGATCACATTACCTATTATTAAAAACATTTCCGTAACTGCTCATGGTATCAATCTTATCGATAAATTTCCATCAAAGTTCTGCAGCTCTTACATACCCTTCCACTACGGAGGCAATGCGATTAAAACCCCCGATGATCCGGGTGCGATGATGATTACCTTTGCTTTGAAGCCACGGGAGGAATACCAACCCAGTGGTCATATTAACGTATCCAGAGCAAGAGAATTTTATATTAGTTGGGACACGGATTACGTGGGGTCTATCACTACGGCTGATCTTGTGGTATCGGCATCTGCTATTAACTT | KJ627200 | [Gallardo et al 2014](http://www.ars.usda.gov/GARA/publications/Atuhaire%20et%20al%202013%20ASF.pdf) | AGTGCGTATACTTGTGCAGATACCAATGTAGACACTTGTGCAAGCATGTGTGCAGATACCAATGTAGACACCTGTGCAAGCATGTGTGCAGATACCAATGTAGATACCTGTGCAAGCACTTGTACAAGCACAGAATAC |
| 280 | Uga12.Busoga1 | Uganda | DP | 2012 | KF303317 | IX | [Atuhaire DK et al 2013](http://www.ars.usda.gov/GARA/publications/Atuhaire%20et%20al%202013%20ASF.pdf) | ATGCAGCCTACCCACCACGCAGAGGTAAGCTTTCAGGATAGAGATACAGCTCTTCCAGATGCATGTTCATCCATATCTGATATTACCCCCATTACTTATCCGATCACATTACCTATTATTAAAAACATTTCCGTTACTGCTCACGGTATCAATCTTATCGATAAATTTCCATCAAAGTTCTGCAGCTCTTACATACCCTTCCACTACGGAGGCAATTCGATTAAAACCCCCGACGATCCGGGCGCGATGATGATTACCTTTGCTTTGAAACCACGGGAGGAATACCAACCCAGCGGTCATATTAACGTATCCAGAGCAAGAGAATTTTATATTAGCTGGGACACAGATTATGTGGGGTCTATCACCACGGCTGATCTTGTGGTATCGGCATCCGCTATTAACTT | KF303296 | [Atuhaire DK et al 2013](http://asf-referencelab.info/asf/images/files/publicaciones/Nix_et_al_2006.pdf) | AGTGCGTATACCTGTGCAAGCACTTGTGCAAGCACTTGTGCAAGCACTTGTGCAGACACCAATGTAGACACTTGTGCAAGCACTTGTGCAGACATTTGTGCAGACACCAATGTAGACACTTGTGCAAGCACTTGTGCAGATACTTGTGCAGACACTTGTGTAAGCACTTGTGTAAGCACTTGTGCAGATACTTGTGCAGACACCAATGTAGACACTTGTGCAAGCACTTGTGCAGACACCAATGTAGACACTTGTGTAAGCACTTGTGCAGACACCTGTGCAAGCACAGAATAC |
| 281 | Ug12.Wakiso | Uganda | DP | 2012 | KC990901 | IX | [Atuhaire DK et al 2013](http://www.ars.usda.gov/GARA/publications/Atuhaire%20et%20al%202013%20ASF.pdf) | ATGCAGCCTACCCACCACGCAGAGGTAAGCTTTCAGGATAGAGATACAGCTCTTCCAGATGCATGTTCATCCATATCTGATATTACCCCCATTACTTATCCGATCACATTACCTATTATTAAAAACATTTCCGTTACTGCTCACGGTATCAATCTTATCGATAAATTTCCATCAAAGTTCTGCAGCTCTTACATACCCTTCCACTACGGAGGCAATTCGATTAAAACCCCCGACGATCCGGGCGCGATGATGATTACCTTTGCTTTGAAACCACGGGAGGAATACCAACCCAGCGGTCATATTAACGTATCCAGAGCAAGAGAATTTTATATTAGCTGGGACACAGATTATGTGGGGTCTATCACCACGGCTGATCTTGTGGTATCGGCATCCGCTATTAACTT | KC990867 | [Atuhaire DK et al 2013](http://asf-referencelab.info/asf/images/files/publicaciones/Nix_et_al_2006.pdf) | AGTGCGTATACCTGTGCAAGCACTTGTGCAAGCACTTGTGCAAGCACTTGTGCAAGCACTTGTGCAGACACCAATGTAGACACTTGTGCAAGCACTTGTGCAGACACTTGTGCAGACACCAATGTAGACACTTGTGCAAGCACTTGTGCAGATACTTGTGCAGACACTTGTGTAAGCACTTGTGTAAGCACTTGTGCAGATACTTGTGCAGACACCAATGTAGACACTTGTGCAAGCACTTGTGCAGACACCAATGTAGACACTTGTGTAAGCACTTGTGCAGACACCTGTGCAAGCACAGAATACCTTTACACATCATGCAAAAAACATTAAATGTAC |
| 282 | Uga12.Kalungu1 | Uganda | DP | 2012 | KF303311 | IX | [Atuhaire DK et al 2013](http://www.ars.usda.gov/GARA/publications/Atuhaire%20et%20al%202013%20ASF.pdf) | ATGCAGCCTACCCACCACGCAGAGGTAAGCTTTCAGGATAGAGATACAGCTCTTCCAGATGCATGTTCATCCGTATCTGATATTACCCCCATTACTTATCCGATCACATTACCTATTATTAAAAACATTTCCGTTACTGCTCACGGTATCAATCTTATCGATAAATTTCCATCAAAGTTCTGCAGCTGTTACATACCCTTCCACTACGGAGGCAATTCGATTAAAACCCCCGACGATCCGGGCGCGATGATGATTACCTTTGCTTTGAAACCACGGGAGGAATACCAACCCAGCGGTCATATTAACGTATCCAGAGCAAGAGAATTTTATATTAGCTGGGACACAGATTATGTGGGGTCTATCACCACGGCTGATCTTGTGGTATCGGCATCCGCTATTAACTT | KF303300.1 | [Atuhaire DK et al 2013](http://www.ars.usda.gov/GARA/publications/Atuhaire%20et%20al%202013%20ASF.pdf) | AGTGCGTATACCTGTGCAAGCACTTGTGCAAGCACTTGTGCAAGCACTTGTGCAAGCACTTGTGCAAGCACTTGTGCAGACACCAATGTAGACACTTGTGCAAGCACGTGTGCAGACACTTGTGCAGACACCAATGTAGACACTTGGGCAAGCACGTGTGCAGATACTTGTGCAGACACTTGTGTAAGCACTTGTGTAAGCACTTGTGCAGATACTTGTGCAGACACCAATGTAGACACTTGTGCAAGCACTTGTGCAGACACCAATGTATACACTTGTGTAAGCACTTGTGCAGACACCTGTGCAAGCACAGAATAC |
| 283 | Ug12.Kampala3 | Uganda | DP | 2012 | KC990900 | IX | [Atuhaire DK et al 2013](http://www.ars.usda.gov/GARA/publications/Atuhaire%20et%20al%202013%20ASF.pdf) | ATGCAGCCTACCCACCACGCAGAGGTAAGCTTTCAGGATAGAGATACAGCTCTTCCAGATGCATGTTCATCCATATCTGATATTACCCCCATTACTTATCCGATCACATTACCTATTATTAAAAACATTTCCGTTACTGCTCACGGTATCAATCTTATCGATAAATTTCCATCAAAGTTCTGCAGCTCTTACATACCCTTCCACTACGGAGGCAATTCGATTAAAACCCCCGACGATCCGGGCGCGATGATGATTACCTTTGCTTTGAAACCACGGGAGGAATACCAACCCAGCGGTCATATTAACGTATCCAGAGCAAGAGAATTTTATATTAGCTGGGACACAGATTATGTGGGGTCTATCACCACGGCTGATCTTGTGGTATCGGCATCCGCTATTAACTT | KC990866 | [Atuhaire DK et al 2013](http://asf-referencelab.info/asf/images/files/publicaciones/Nix_et_al_2006.pdf) | AGTGCGTATACCTGTGCAAGCACTTGTGCAAGCACTTGTGCAAGCACTTGTGCAGACACCAATGTAGACACTTGTGCAAGCACTTGTGCAGACATTTGTGCAGACACCAATGTAGACACTTGTGCAAGCACTTGTGCAGATACTTGTGCAGACACTTGTGTAAGCACTTGTGTAAGCACTTGTGCAGATACTTGTGCAGACACCAATGTAGACACTTGTGCAAGCACTTGTGCAGACACCAATGTAGACACTTGTGTAAGCACTTGTGCAGACACCTGTGCAAGCACAGAATACCTTTACACATCATGCAAAAAACATTAAATGTAC |
| 284 | Ug12.Kampala4 | Uganda | DP | 2012 | KC990904 | IX | [Atuhaire DK et al 2013](http://www.ars.usda.gov/GARA/publications/Atuhaire%20et%20al%202013%20ASF.pdf) | ATGCAGCCTACCCACCACGCAGAGGTAAGCTTTCAGGATAGAGATACAGCTCTTCCAGATGCATGTTCATCCATATCTGATATTACCCCCATTACTTATCCGATCACATTACCTATTATTAAAAACATTTCCGTTACTGCTCACGGTATCAATCTTATCGATAAATTTCCATCAAAGTTCTGCAGCTCTTACATACCCTTCCACTACGGAGGCAATTCGATTAAAACCCCCGACGATCCGGGCGCGATGATGATTACCTTTGCTTTGAAACCACGGGAGGAATACCAACCCAGCGGTCATATTAACGTATCCAGAGCAAGAGAATTTTATATTAGCTGGGACACAGATTATGTGGGGTCTATCACCACGGCTGATCTTGTGGTATCGGCATCCGCTATTAACTT | KC990870 | [Atuhaire DK et al 2013](http://www.ars.usda.gov/GARA/publications/Atuhaire%20et%20al%202013%20ASF.pdf) | AGTGCGTATACCTGTGCAAGCACTTGTGCAAGCACTTGTGCAAGCACTTGTGCAAGCACTTGTGCAGACACCAATGTAGACACTTGTGCAAGCACTTGTGCAGACACTTGTGCAGACACCAATGTAGACACTTGTGCAAGCACTTGTGCAGATACTTGTGCAGACACTTGTGTAAGCACTTGTGTAAGCACTTGTGCAGATACTTGTGCAGACACCAATGTAGACACTTGTGCAAGCACTTGTGCAGACACCAATGTAGACACTTGTGTAAGCACTTGTGCAGACACCTGTGCAAGCACAGAATACCTTTACACATCATGCAAAAAACATTAAATGTAC |
| 285 | Tver0712/Les | Russia | DP | 2012 | KJ627210 | II | [Gallardo et al 2014](http://www.ars.usda.gov/GARA/publications/Atuhaire%20et%20al%202013%20ASF.pdf) | ATGCAGCCCACTCACCACGCAGAGATAAGCTTTCAGGATAGAGATACAGCTCTTCCAGACGCATGTTCATCTATATCTGATATTAGCCCCGTTACGTATCCGATCACATTACCTATTATTAAAAACATTTCCGTAACTGCTCATGGTATCAATCTTATCGATAAATTTCCATCAAAGTTCTGCAGCTCTTACATACCCTTCCACTACGGAGGCAATGCGATTAAAACCCCCGATGATCCGGGTGCGATGATGATTACCTTTGCTTTGAAGCCACGGGAGGAATACCAACCCAGTGGTCATATTAACGTATCCAGAGCAAGAGAATTTTATATTAGTTGGGACACGGATTACGTGGGGTCTATCACTACGGCTGATCTTGTGGTATCGGCATCTGCTATTAACTT | KJ627199 | [Gallardo et al 2014](http://www.ars.usda.gov/GARA/publications/Atuhaire%20et%20al%202013%20ASF.pdf) | AGTGCGTATACTTGTGCAGATACCAATGTAGACACTTGTGCAAGCATGTGTGCAGATACCAATGTAGACACCTGTGCAAGCATGTGTGCAGATACCAATGTAGATACCTGTGCAAGCACTTGTACAAGCACAGAATAC |
| 286 | Ukr12/Zapo | Ukraine | DP | 2012 | JX857521 | II | [Gallardo et al 2014](http://www.ars.usda.gov/GARA/publications/Atuhaire%20et%20al%202013%20ASF.pdf) | ATGCAGCCCACTCACCACGCAGAGATAAGCTTTCAGGATAGAGATACAGCTCTTCCAGACGCATGTTCATCTATATCTGATATTAGCCCCGTTACGTATCCGATCACATTACCTATTATTAAAAACATTTCCGTAACTGCTCATGGTATCAATCTTATCGATAAATTTCCATCAAAGTTCTGCAGCTCTTACATACCCTTCCACTACGGAGGCAATGCGATTAAAACCCCCGATGATCCGGGTGCGATGATGATTACCTTTGCTTTGAAGCCACGGGAGGAATACCAACCCAGTGGTCATATTAACGTATCCAGAGCAAGAGAATTTTATATTAGTTGGGACACGGATTACGTGGGGTCTATCACTACGGCTGATCTTGTGGTATCGGCATCTGCTATTAACTT | JX857535 | [Gallardo et al 2014](http://www.ars.usda.gov/GARA/publications/Atuhaire%20et%20al%202013%20ASF.pdf) | AGTGCGTATACTTGTGCAGATACCAATGTAGACACTTGTGCAAGCATGTGTGCAGATACCAATGTAGACACCTGTGCAAGCATGTGTGCAGATACCAATGTAGATACCTGTGCAAGCACTTGTACAAGCACAGAATAC |
| 287 | Tver0812/Bolo | Russia | Wild pig | 2012 | KJ627209 | II | [Gallardo et al 2014](http://www.ars.usda.gov/GARA/publications/Atuhaire%20et%20al%202013%20ASF.pdf) | ATGCAGCCCACTCACCACGCAGAGATAAGCTTTCAGGATAGAGATACAGCTCTTCCAGACGCATGTTCATCTATATCTGATATTAGCCCCGTTACGTATCCGATCACATTACCTATTATTAAAAACATTTCCGTAACTGCTCATGGTATCAATCTTATCGATAAATTTCCATCAAAGTTCTGCAGCTCTTACATACCCTTCCACTACGGAGGCAATGCGATTAAAACCCCCGATGATCCGGGTGCGATGATGATTACCTTTGCTTTGAAGCCACGGGAGGAATACCAACCCAGTGGTCATATTAACGTATCCAGAGCAAGAGAATTTTATATTAGTTGGGACACGGATTACGTGGGGTCTATCACTACGGCTGATCTTGTGGTATCGGCATCTGCTATTAACTT | KJ627198 | [Gallardo et al 2014](http://asf-referencelab.info/asf/images/files/publicaciones/Nix_et_al_2006.pdf) | AGTGCGTATACTTGTGCAGATACCAATGTAGACACTTGTGCAAGCATGTGTGCAGATACCAATGTAGACACCTGTGCAAGCATGTGTGCAGATACCAATGTAGATACCTGTGCAAGCACTTGTACAAGCACAGAATAC |
| 288 | Uga12.Nakaseke | Uganda | DP | 2012 | KF303316 | IX | [Atuhaire DK et al 2013](http://asf-referencelab.info/asf/images/files/publicaciones/Gallardo-et-al-2009a.pdf) | ATGCAGCCTACCCACCACGCAGAGGTAAGCTTTCAGGATAGAGATACAGCTCTTCCAGATGCATGTTCATCCATATCTGATATTACCCCCATTACTTATCCGATCACATTACCTATTATTAAAAACATTTCCGTTACTGCTCACGGTATCAATCTTATCGATAAATTTCCATCAAAGTTCTGCAGCTCTTACATACCCTTCCACTACGGAGGCAATTCGATTAAAACCCCCGACGATCCGGGCGCGATGATGATTACCTTTGCTTTGAAACCACGGGAGGAATACCAACCCAGCGGTCATATTAACGTATCCAGAGCAAGAGAATTTTATATTAGCTGGGACACAGATTATGTGGGGTCTATCACCACGGCTGATCTTGTGGTATCGGCATCCGCTATTAACTT | KF303301 | [Atuhaire DK et al 2013](http://asf-referencelab.info/asf/images/files/publicaciones/Nix_et_al_2006.pdf) | AGTGCGTATACCTGTGCAAGCACTTGTGCAAGCACTTGTGCAAGCACTTGTGCAGACACCAATGTAGACACTTGTGCAAGCACTTGTGTAGACATTTGTGCAGACACCAATGTAGACACTTGTGCAAGCACTTGTGCAGATACTTGTGCAGACACTTGTGTAAGCACTTGTGTAAGCACTTGTGCAGATACTTGTGCAGACACCAATGTAGACACTTGTGCAAGCACTTGTGCAGACACCAATGTAGACACTTGTGTAAGCACTTGTGCAGACACCTGTGCAAGCACAGAATAC |
| 289 | Ug12.Kabale1 | Uganda | DP | 2012 | KC990890 | IX | [Atuhaire DK et al 2013](http://asf-referencelab.info/asf/images/files/publicaciones/Gallardo-et-al-2009a.pdf) | ATGCAGCCTACCCACCACGCAGAGGTAAGCTTTCAGGATAGAGATACAGCTCTTCCAGATGCATGTTCATCCATATCTGATATTACCCCCATTACTTATCCGATCACATTACCTATTATTAAAAACATTTCCGTTACTGCTCACGGTATCAATCTTATCGATAAATTTCCATCAAAGTTCTGCAGCTCTTACATACCCTTCCACTACGGAGGCAATTCGATTAAAACCCCCGACGATCCGGGCGCGATGATGATTACCTTTGCTTTGAAACCACGGGAGGAATACCAACCCAGCGGTCATATTAACGTATCCAGAGCAAGAGAATTTTATATTAGCTGGGACACAGATTATGTGGGGTCTATCACCACGGCTGATCTTGTGGTATCGGCATCCGCTATTAACTT | KC990857 | [Atuhaire DK et al 2013](http://asf-referencelab.info/asf/images/files/publicaciones/Nix_et_al_2006.pdf) | AGTGCGTATACCTGTGCAAGCACTTGTGCAAGCACTTGTGCAAGCACTTGTGCAAGCACTTGTGCAGACACCAATGTAGACACTTGTGCAAGCACTTGTGCAGACACTTGTGCAGACACCAATGTAGACACTTGTGCAAGCACTTGTGCAGATACTTGTGCAGACACTTGTGTAAGCACTTGTGTAAGCACTTGTGCAGATACTTGTGCAGACACCAATGTAGACACTTGTGCAAGCACTTGTGCAGACACCAATGTAGACACTTGTGTAAGCACTTGTGCAGACACCTGTGCAAGCACAGAATACCTTTACACATCATGCAAAAAACATTAAATGTAC |
| 290 | Ug12.Lira | Uganda | DP | 2012 | KC990899 | IX | [Atuhaire DK et al 2013](http://asf-referencelab.info/asf/images/files/publicaciones/Gallardo-et-al-2009a.pdf) | ATGCAGCCTACCCACCACGCAGAGGTAAGCTTTCAGGATAGAGATACAGCTCTTCCAGATGCATGTTCATCCATATCTGATATTACCCCCATTACTTATCCGATCACATTACCTATTATTAAAAACATTTCCGTTACTGCTCACGGTATCAATCTTATCGATAAATTTCCATCAAAGTTCTGCAGCTCTTACATACCCTTCCACTACGGAGGCAATTCGATTAAAACCCCCGACGATCCGGGCGCGATGATGATTACCTTTGCTTTGAAACCACGGGAGGAATACCAACCCAGCGGTCATATTAACGTATCCAGAGCAAGAGAATTTTATATTAGCTGGGACACAGATTATGTGGGGTCTATCACCACGGCTGATCTTGTGGTATCGGCATCCGCTATTAACTT | KC990865 | [Atuhaire DK et al 2013](http://asf-referencelab.info/asf/images/files/publicaciones/Nix_et_al_2006.pdf) | AGTGCGTATACCTGTGCAAGCACTTGTGCAAGCACTTGTGCAAGCACTTGTGCAAGCACTTGTGCAGACACCAATGTAGACACTTGTGCAAGCACTTGTGCAGACACTTGTGCAGACACCAATGTAGACACTTGTGCAAGCACTTGTGCAGATACTTGTGCAGACACTTGTGTAAGCACTTGTGTAAGCACTTGTGCAGATACTTGTGCAGACACCAATGTAGACACTTGTGCAAGCACTTGTGCAGACACCAATGTAGACACTTGTGTAAGCACTTGTGCAGACACCTGTGCAAGCACAGAATACCTTTACACATCATGCAAAAAACATTAAATGTAC |
| 291 | Ug12.Kyenjojo | Uganda | DP | 2012 | KC990903 | IX | [Atuhaire DK et al 2013](http://asf-referencelab.info/asf/images/files/publicaciones/Gallardo-et-al-2009a.pdf) | ATGCAGCCTACCCACCACGCAGAGGTAAGCTTTCAGGATAGAGATACAGCTCTTCCAGATGCATGTTCATCCATATCTGATATTACCCCCATTACTTATCCGATCACATTACCTATTATTAAAAACATTTCCGTTACTGCTCACGGTATCAATCTTATCGATAAATTTCCATCAAAGTTCTGCAGCTCTTACATACCCTTCCACTACGGAGGCAATTCGATTAAAACCCCCGACGATCCGGGCGCGATGATGATTACCTTTGCTTTGAAACCACGGGAGGAATACCAACCCAGCGGTCATATTAACGTATCCAGAGCAAGAGAATTTTATATTAGCTGGGACACAGATTATGTGGGGTCTATCACCACGGCTGATCTTGTGGTATCGGCATCCGCTATTAACTT | KC990869 | [Atuhaire DK et al 2013](http://asf-referencelab.info/asf/images/files/publicaciones/Nix_et_al_2006.pdf) | AGTGCGTATACCTGTGCAAGCACTTGTGCAAGCACTTGTGCAAGCACTTGTGCAGACACCAATGTAGACACTTGTGCAAGCACTTGTGCAGACATTTGTGCAGACACCAATGTAGACACTTGTGCAAGCACTTGTGCAGATACTTGTGCAGACACTTGTGTAAGCACTTGTGTAAGCACTTGTGCAGATACTTGTGCAGACACCAATGTAGACACTTGTGCAAGCACTTGTGCAGACACCAATGTAGACACTTGTGTAAGCACTTGTGCAGACACCTGTGCAAGCACAGAATACCTTTACACATCATGCAAAAAACATTAAATGTAC |
| 292 | Tver1112/Zavi | Russia | Wild pig | 2012 | KJ627214 | II | [Gallardo et al 2014](http://asf-referencelab.info/asf/images/files/publicaciones/Gallardo-et-al-2009a.pdf) | ATGCAGCCCACTCACCACGCAGAGATAAGCTTTCAGGATAGAGATACAGCTCTTCCAGACGCATGTTCATCTATATCTGATATTAGCCCCGTTACGTATCCGATCACATTACCTATTATTAAAAACATTTCCGTAACTGCTCATGGTATCAATCTTATCGATAAATTTCCATCAAAGTTCTGCAGCTCTTACATACCCTTCCACTACGGAGGCAATGCGATTAAAACCCCCGATGATCCGGGTGCGATGATGATTACCTTTGCTTTGAAGCCACGGGAGGAATACCAACCCAGTGGTCATATTAACGTATCCAGAGCAAGAGAATTTTATATTAGTTGGGACACGGATTACGTGGGGTCTATCACTACGGCTGATCTTGTGGTATCGGCATCTGCTATTAACTT | KJ627202 | [Gallardo et al 2014](http://asf-referencelab.info/asf/images/files/publicaciones/Nix_et_al_2006.pdf) | AGTGCGTATACTTGTGCAGATACCAATGTAGACACTTGTGCAAGCATGTGTGCAGATACCAATGTAGACACCTGTGCAAGCATGTGTGCAGATACCAATGTAGATACCTGTGCAAGCACTTGTACAAGCACAGAATAC |
| 293 | Uga12.Kibaale | Uganda | DP | 2012 | KF303315 | IX | [Atuhaire DK et al 2013](http://asf-referencelab.info/asf/images/files/publicaciones/Gallardo-et-al-2009a.pdf) | ATGCAGCCTACCCACCACGCAGAGGTAAGCTTTCAGGATAGAGATACAGCTCTTCCAGATGCATGTTCATCCATATCTGATATTACCCCCATTACTTATCCGATCACATTACCTATTATTAAAAACATTTCCGTTACTGCTCACGGTATCAATCTTATCGATAAATTTCCATCAAAGTTCTGCAGCTCTTACATACCCTTCCACTACGGAGGCAATTCGATTAAAACCCCCGACGATCCGGGCGCGATGATGATTACCTTTGCTTTGAAACCACGGGAGGAATACCAACCCAGCGGTCATATTAACGTATCCAGAGCAAGAGAATTTTATATTAGCTGGGACACAGATTATGTGGGGTCTATCACCACGGCTGATCTTGTGGTATCGGCATCCGCTATTAACTT | KF303299.1 | [Atuhaire DK et al 2013](http://asf-referencelab.info/asf/images/files/publicaciones/Nix_et_al_2006.pdf) | AGTGCGTATACCTGTGCAAGCACTTGTGCAAGCACTTGTGCAAGCACTTGTGCAAGCACTTGTGCAAGCACTTGTGCAGACACCAATGTAGACACTTGTGCAAGCACTTGTGCAGACACTTGTGCAGACACCAATGTAGACACTTGTGCAAGCACTTGTGCAGATACTTGTGCAGACACTTGTGTAAGCACTTGTGTAAGCACTTGTGCAGATACTTGTGCAGACACCAATGTAGACACTTGTGCAAGCACTTGTGCAGACACCAATGTAGACACTTGTGTAAGCACTTGTGCAGACACCTGTGCAAGCACAGAATAC |
| 294 | Ug13.Kampala1 | Uganda | DP | 2013 | KC990891 | IX | [Atuhaire DK et al 2013](http://asf-referencelab.info/asf/images/files/publicaciones/Gallardo-et-al-2009a.pdf) | ATGCAGCCTACCCACCACGCAGAGGTAAGCTTTCAGGATAGAGATACAGCTCTTCCAGATGCATGTTCATCCATATCTGATATTACCCCCATTACTTATCCGATCACATTACCTATTATTAAAAACATTTCCGTTACTGCTCACGGTATCAATCTTATCGATAAATTTCCATCAAAGTTCTGCAGCTCTTACATACCCTTCCACTACGGAGGCAATTCGATTAAAACCCCCGACGATCCGGGCGCGATGATGATTACCTTTGCTTTGAAACCACGGGAGGAATACCAACCCAGCGGTCATATTAACGTATCCAGAGCAAGAGAATTTTATATTAGCTGGGACACAGATTATGTGGGGTCTATCACCACGGCTGATCTTGTGGTATCGGCATCCGCTATTAACTT | KC990856 | [Atuhaire DK et al 2013](http://asf-referencelab.info/asf/images/files/publicaciones/Nix_et_al_2006.pdf) | AGTGCGTATACCTGTGCAAGCACTTGTGCAAGCACTTGTGCAAGCACTTGTGCAAGCACTTGTGCAAGCACTTGTGCAGACACCAATGTAGACACTTGTGCAAGCGCAGACACCAATGTAGACACTTGTGTAAGCACTTGTGCAGACACCTGTGCAAGCACAGAATACCTTTACACATCATGCAAAAAACATTAAATGTAC |
| 295 | Ug13.Busia1 | Uganda | DP | 2013 | KC990905 | IX | [Atuhaire DK et al 2013](http://asf-referencelab.info/asf/images/files/publicaciones/Gallardo-et-al-2009a.pdf) | ATGCAGCCTACCCACCACGCAGAGGTAAGCTTTCAGGATAGAGATACAGCTCTTCCAGATGCATGTTCATCCATATCTGATATTACCCCCATTACTTATCCGATCACATTACCTATTATTAAAAACATTTCCGTTACTGCTCACGGTATCAATCTTATCGATAAATTTCCATCAAAGTTCTGCAGCTCTTACATACCCTTCCACTACGGAGGCAATTCGATTAAAACCCCCGACGATCCGGGCGCGATGATGATTACCTTTGCTTTGAAACCACGGGAGGAATACCAACCCAGCGGTCATATTAACGTATCCAGAGCAAGAGAATTTTATATTAGCTGGGACACAGATTATGTGGGGTCTATCACCACGGCTGATCTTGTGGTATCGGCATCCGCTATTAACTT | KC990871 | [Atuhaire DK et al 2013](http://asf-referencelab.info/asf/images/files/publicaciones/Nix_et_al_2006.pdf) | AGTGCGTATACCTGTGCAAGCACTTGTGCAAGCACTTGTGCAAGCACTTGTGCAGACACCAATGTAGACACTTGTGCAAGCACTTGTGCAGACATTTGTGCAGACACCAATGTAGACACTTGTGCAAGCACTTGTGCAGATACTTGTGCAGACACTTGTGTAAGCACTTGTGTAAGCACTTGTGCAGATACTTGTGCAGACACCAATGTAGACACTTGTGCAAGCACTTGTGCAGACACCAATGTAGACACTTGTGTAAGCACTTGTGCAGACACCTGTGCAAGCACAGAATACCTTTACACATCATGCAAAAAACATTAAATGTAC |
| 296 | Ug13.Busia2 | Uganda | DP | 2013 | KC990906 | IX | [Atuhaire DK et al 2013](http://asf-referencelab.info/asf/images/files/publicaciones/Gallardo-et-al-2009a.pdf) | ATGCAGCCTACCCACCACGCAGAGGTAAGCTTTCAGGATAGAGATACAGCTCTTCCAGATGCATGTTCATCCATATCTGATATTACCCCCATTACTTATCCGATCACATTACCTATTATTAAAAACATTTCCGTTACTGCTCACGGTATCAATCTTATCGATAAATTTCCATCAAAGTTCTGCAGCTCTTACATACCCTTCCACTACGGAGGCAATTCGATTAAAACCCCCGACGATCCGGGCGCGATGATGATTACCTTTGCTTTGAAACCACGGGAGGAATACCAACCCAGCGGTCATATTAACGTATCCAGAGCAAGAGAATTTTATATTAGCTGGGACACAGATTATGTGGGGTCTATCACCACGGCTGATCTTGTGGTATCGGCATCCGCTATTAACTT | KC990872 | [Atuhaire DK et al 2013](http://asf-referencelab.info/asf/images/files/publicaciones/Nix_et_al_2006.pdf) | AGTGCGTATACCTGTGCAAGCACTTGTGCAAGCACTTGTGCAAGCACTTGTGCAGACACCAATGTAGACACTTGTGCAAGCACTTGTGCAGACATTTGTGCAGACACCAATGTAGACACTTGTGCAAGCACTTGTGCAGATACTTGTGCAGACACTTGTGTAAGCACTTGTGTAAGCACTTGTGCAGATACTTGTGCAGACACCAATGTAGACACTTGTGCAAGCACTTGTGCAGACACCAATGTAGACACTTGTGTAAGCACTTGTGCAGACACCTGTGCAAGCACAGAATACCTTTACACATCATGCAAAAAACATTAAATGTAC |
| 297 | Bel13/Grodno | Belarus | DP | 2013 | KJ627215 | II | [Gallardo et al 2014](http://asf-referencelab.info/asf/images/files/publicaciones/Gallardo-et-al-2009a.pdf) | ATGCAGCCCACTCACCACGCAGAGATAAGCTTTCAGGATAGAGATACAGCTCTTCCAGACGCATGTTCATCTATATCTGATATTAGCCCCGTTACGTATCCGATCACATTACCTATTATTAAAAACATTTCCGTAACTGCTCATGGTATCAATCTTATCGATAAATTTCCATCAAAGTTCTGCAGCTCTTACATACCCTTCCACTACGGAGGCAATGCGATTAAAACCCCCGATGATCCGGGTGCGATGATGATTACCTTTGCTTTGAAGCCACGGGAGGAATACCAACCCAGTGGTCATATTAACGTATCCAGAGCAAGAGAATTTTATATTAGTTGGGACACGGATTACGTGGGGTCTATCACTACGGCTGATCTTGTGGTATCGGCATCTGCTATTAACTT | KJ627203 | [Gallardo et al 2014](http://asf-referencelab.info/asf/images/files/publicaciones/Nix_et_al_2006.pdf) | AGTGCGTATACTTGTGCAGATACCAATGTAGACACTTGTGCAAGCATGTGTGCAGATACCAATGTAGACACCTGTGCAAGCATGTGTGCAGATACCAATGTAGATACCTGTGCAAGCACTTGTACAAGCACAGAATAC |
| 298 | Lv15/WB/DAU-1 | Latvia | Wild pig | 2015 |  | II | [This study (gallardo@inia.es)](http://asf-referencelab.info/asf/images/files/publicaciones/Gallardo-et-al-2009a.pdf) | ATGCAGCCCACTCACCACGCAGAGATAAGCTTTCAGGATAGAGATACAGCTCTTCCAGACGCATGTTCATCTATATCTGATATTAGCCCCGTTACGTATCCGATCACATTACCTATTATTAAAAACATTTCCGTAACTGCTCATGGTATCAATCTTATCGATAAATTTCCATCAAAGTTCTGCAGCTCTTACATACCCTTCCACTACGGAGGCAATGCGATTAAAACCCCCGATGATCCGGGTGCGATGATGATTACCTTTGCTTTGAAGCCACGGGAGGAATACCAACCCAGTGGTCATATTAACGTATCCAGAGCAAGAGAATTTTATATTAGTTGGGACACGGATTACGTGGGGTCTATCACTACGGCTGATCTTGTGGTATCGGCATCTGCTATTAACTT |  | [This study (gallardo@inia.es)](http://asf-referencelab.info/asf/images/files/publicaciones/Nix_et_al_2006.pdf) | AGTGCGTATACTTGTGCAGATACCAATGTAGACACTTGTGCAAGCATGTGTGCAGATACCAATGTAGACACCTGTGCAAGCATGTGTGCAGATACCAATGTAGATACCTGTGCAAGCACTTGTACAAGCACAGAATAC |
| 299 | LT14/1482 | Lithuania | Wild pig | 2014 | KJ627216 | II | [Gallardo et al 2014](http://asf-referencelab.info/asf/images/files/publicaciones/Gallardo-et-al-2009a.pdf) | ATGCAGCCCACTCACCACGCAGAGATAAGCTTTCAGGATAGAGATACAGCTCTTCCAGACGCATGTTCATCTATATCTGATATTAGCCCCGTTACGTATCCGATCACATTACCTATTATTAAAAACATTTCCGTAACTGCTCATGGTATCAATCTTATCGATAAATTTCCATCAAAGTTCTGCAGCTCTTACATACCCTTCCACTACGGAGGCAATGCGATTAAAACCCCCGATGATCCGGGTGCGATGATGATTACCTTTGCTTTGAAGCCACGGGAGGAATACCAACCCAGTGGTCATATTAACGTATCCAGAGCAAGAGAATTTTATATTAGTTGGGACACGGATTACGTGGGGTCTATCACTACGGCTGATCTTGTGGTATCGGCATCTGCTATTAACTT | KJ627205 | [Gallardo et al 2014](http://asf-referencelab.info/asf/images/files/publicaciones/Nix_et_al_2006.pdf) | AGTGCGTATACTTGTGCAGATACCAATGTAGACACTTGTGCAAGCATGTGTGCAGATACCAATGTAGACACCTGTGCAAGCATGTGTGCAGATACCAATGTAGATACCTGTGCAAGCACTTGTACAAGCACAGAATAC |
| 300 | LT14/1490 | Lithuania | Wild pig | 2014 | KJ627215 | II | [Gallardo et al 2014](http://asf-referencelab.info/asf/images/files/publicaciones/Gallardo-et-al-2009a.pdf) | ATGCAGCCCACTCACCACGCAGAGATAAGCTTTCAGGATAGAGATACAGCTCTTCCAGACGCATGTTCATCTATATCTGATATTAGCCCCGTTACGTATCCGATCACATTACCTATTATTAAAAACATTTCCGTAACTGCTCATGGTATCAATCTTATCGATAAATTTCCATCAAAGTTCTGCAGCTCTTACATACCCTTCCACTACGGAGGCAATGCGATTAAAACCCCCGATGATCCGGGTGCGATGATGATTACCTTTGCTTTGAAGCCACGGGAGGAATACCAACCCAGTGGTCATATTAACGTATCCAGAGCAAGAGAATTTTATATTAGTTGGGACACGGATTACGTGGGGTCTATCACTACGGCTGATCTTGTGGTATCGGCATCTGCTATTAACTT | KJ627204 | [Gallardo et al 2014](http://asf-referencelab.info/asf/images/files/publicaciones/Nix_et_al_2006.pdf) | AGTGCGTATACTTGTGCAGATACCAATGTAGACACTTGTGCAAGCATGTGTGCAGATACCAATGTAGACACCTGTGCAAGCATGTGTGCAGATACCAATGTAGATACCTGTGCAAGCACTTGTACAAGCACAGAATAC |
| 301 | Pol14/Sz | Poland | Wild pig | 2014 | KJ627218 | II | [Gallardo et al 2014](http://link.springer.com/article/10.1007%2Fs00705-005-0602-1) | ATGCAGCCCACTCACCACGCAGAGATAAGCTTTCAGGATAGAGATACAGCTCTTCCAGACGCATGTTCATCTATATCTGATATTAGCCCCGTTACGTATCCGATCACATTACCTATTATTAAAAACATTTCCGTAACTGCTCATGGTATCAATCTTATCGATAAATTTCCATCAAAGTTCTGCAGCTCTTACATACCCTTCCACTACGGAGGCAATGCGATTAAAACCCCCGATGATCCGGGTGCGATGATGATTACCTTTGCTTTGAAGCCACGGGAGGAATACCAACCCAGTGGTCATATTAACGTATCCAGAGCAAGAGAATTTTATATTAGTTGGGACACGGATTACGTGGGGTCTATCACTACGGCTGATCTTGTGGTATCGGCATCTGCTATTAACTT | KJ627206 | [Gallardo et al 2014](http://asf-referencelab.info/asf/images/files/publicaciones/Nix_et_al_2006.pdf) | AGTGCGTATACTTGTGCAGATACCAATGTAGACACTTGTGCAAGCATGTGTGCAGATACCAATGTAGACACCTGTGCAAGCATGTGTGCAGATACCAATGTAGATACCTGTGCAAGCACTTGTACAAGCACAGAATAC |
| 302 | Pol14/Krus | Poland | Wild pig | 2014 | KJ627219 | II | [Gallardo et al 2014](http://link.springer.com/article/10.1007%2Fs00705-005-0602-1) | ATGCAGCCCACTCACCACGCAGAGATAAGCTTTCAGGATAGAGATACAGCTCTTCCAGACGCATGTTCATCTATATCTGATATTAGCCCCGTTACGTATCCGATCACATTACCTATTATTAAAAACATTTCCGTAACTGCTCATGGTATCAATCTTATCGATAAATTTCCATCAAAGTTCTGCAGCTCTTACATACCCTTCCACTACGGAGGCAATGCGATTAAAACCCCCGATGATCCGGGTGCGATGATGATTACCTTTGCTTTGAAGCCACGGGAGGAATACCAACCCAGTGGTCATATTAACGTATCCAGAGCAAGAGAATTTTATATTAGTTGGGACACGGATTACGTGGGGTCTATCACTACGGCTGATCTTGTGGTATCGGCATCTGCTATTAACTT | KJ627207 | [Gallardo et al 2014](http://asf-referencelab.info/asf/images/files/publicaciones/Nix_et_al_2006.pdf) | AGTGCGTATACTTGTGCAGATACCAATGTAGACACTTGTGCAAGCATGTGTGCAGATACCAATGTAGACACCTGTGCAAGCATGTGTGCAGATACCAATGTAGATACCTGTGCAAGCACTTGTACAAGCACAGAATAC |
| 303 | Pol14/WB-Los | Poland | Wild pig | 2014 | KM374540 | II | [This study (gallardo@inia.es)](http://link.springer.com/article/10.1007%2Fs00705-005-0602-1) | ATGCAGCCCACTCACCACGCAGAGATAAGCTTTCAGGATAGAGATACAGCTCTTCCAGACGCATGTTCATCTATATCTGATATTAGCCCCGTTACGTATCCGATCACATTACCTATTATTAAAAACATTTCCGTAACTGCTCATGGTATCAATCTTATCGATAAATTTCCATCAAAGTTCTGCAGCTCTTACATACCCTTCCACTACGGAGGCAATGCGATTAAAACCCCCGATGATCCGGGTGCGATGATGATTACCTTTGCTTTGAAGCCACGGGAGGAATACCAACCCAGTGGTCATATTAACGTATCCAGAGCAAGAGAATTTTATATTAGTTGGGACACGGATTACGTGGGGTCTATCACTACGGCTGATCTTGTGGTATCGGCATCTGCTATTAACTT |  | [This study (gallardo@inia.es)](http://asf-referencelab.info/asf/images/files/publicaciones/Nix_et_al_2006.pdf) | AGTGCGTATACTTGTGCAGATACCAATGTAGACACTTGTGCAAGCATGTGTGCAGATACCAATGTAGACACCTGTGCAAGCATGTGTGCAGATACCAATGTAGATACCTGTGCAAGCACTTGTACAAGCACAGAATAC |
| 304 | Pol14/WB1-10986 | Poland | Wild pig | 2014 | KM374541 | II | [This study (gallardo@inia.es)](http://link.springer.com/article/10.1007%2Fs00705-005-0602-1) | ATGCAGCCCACTCACCACGCAGAGATAAGCTTTCAGGATAGAGATACAGCTCTTCCAGACGCATGTTCATCTATATCTGATATTAGCCCCGTTACGTATCCGATCACATTACCTATTATTAAAAACATTTCCGTAACTGCTCATGGTATCAATCTTATCGATAAATTTCCATCAAAGTTCTGCAGCTCTTACATACCCTTCCACTACGGAGGCAATGCGATTAAAACCCCCGATGATCCGGGTGCGATGATGATTACCTTTGCTTTGAAGCCACGGGAGGAATACCAACCCAGTGGTCATATTAACGTATCCAGAGCAAGAGAATTTTATATTAGTTGGGACACGGATTACGTGGGGTCTATCACTACGGCTGATCTTGTGGTATCGGCATCTGCTATTAACTT |  | [This study (gallardo@inia.es)](http://asf-referencelab.info/asf/images/files/publicaciones/Nix_et_al_2006.pdf) | AGTGCGTATACTTGTGCAGATACCAATGTAGACACTTGTGCAAGCATGTGTGCAGATACCAATGTAGACACCTGTGCAAGCATGTGTGCAGATACCAATGTAGATACCTGTGCAAGCACTTGTACAAGCACAGAATAC |
| 305 | Pol14/WB2-10986 | Poland | Wild pig | 2014 | KM374542 | II | [This study (gallardo@inia.es)](http://asf-referencelab.info/asf/images/files/publicaciones/Gallardo-et-al-2009a.pdf) | ATGCAGCCCACTCACCACGCAGAGATAAGCTTTCAGGATAGAGATACAGCTCTTCCAGACGCATGTTCATCTATATCTGATATTAGCCCCGTTACGTATCCGATCACATTACCTATTATTAAAAACATTTCCGTAACTGCTCATGGTATCAATCTTATCGATAAATTTCCATCAAAGTTCTGCAGCTCTTACATACCCTTCCACTACGGAGGCAATGCGATTAAAACCCCCGATGATCCGGGTGCGATGATGATTACCTTTGCTTTGAAGCCACGGGAGGAATACCAACCCAGTGGTCATATTAACGTATCCAGAGCAAGAGAATTTTATATTAGTTGGGACACGGATTACGTGGGGTCTATCACTACGGCTGATCTTGTGGTATCGGCATCTGCTATTAACTT |  | [This study (gallardo@inia.es)](http://asf-referencelab.info/asf/images/files/publicaciones/Nix_et_al_2006.pdf) | AGTGCGTATACTTGTGCAGATACCAATGTAGACACTTGTGCAAGCATGTGTGCAGATACCAATGTAGACACCTGTGCAAGCATGTGTGCAGATACCAATGTAGATACCTGTGCAAGCACTTGTACAAGCACAGAATAC |
| 306 | Pol14/WB3-10986 | Poland | Wild pig | 2014 | KM374543 | II | [This study (gallardo@inia.es)](http://asf-referencelab.info/asf/images/files/publicaciones/Gallardo-et-al-2009a.pdf) | ATGCAGCCCACTCACCACGCAGAGATAAGCTTTCAGGATAGAGATACAGCTCTTCCAGACGCATGTTCATCTATATCTGATATTAGCCCCGTTACGTATCCGATCACATTACCTATTATTAAAAACATTTCCGTAACTGCTCATGGTATCAATCTTATCGATAAATTTCCATCAAAGTTCTGCAGCTCTTACATACCCTTCCACTACGGAGGCAATGCGATTAAAACCCCCGATGATCCGGGTGCGATGATGATTACCTTTGCTTTGAAGCCACGGGAGGAATACCAACCCAGTGGTCATATTAACGTATCCAGAGCAAGAGAATTTTATATTAGTTGGGACACGGATTACGTGGGGTCTATCACTACGGCTGATCTTGTGGTATCGGCATCTGCTATTAACTT |  | [This study (gallardo@inia.es)](http://asf-referencelab.info/asf/images/files/publicaciones/Nix_et_al_2006.pdf) | AGTGCGTATACTTGTGCAGATACCAATGTAGACACTTGTGCAAGCATGTGTGCAGATACCAATGTAGACACCTGTGCAAGCATGTGTGCAGATACCAATGTAGATACCTGTGCAAGCACTTGTACAAGCACAGAATAC |
| 307 | Pol14/WB-13119 | Poland | Wild pig | 2014 | KM374544 | II | [This study (gallardo@inia.es)](http://asf-referencelab.info/asf/images/files/publicaciones/Gallardo-et-al-2009a.pdf) | ATGCAGCCCACTCACCACGCAGAGATAAGCTTTCAGGATAGAGATACAGCTCTTCCAGACGCATGTTCATCTATATCTGATATTAGCCCCGTTACGTATCCGATCACATTACCTATTATTAAAAACATTTCCGTAACTGCTCATGGTATCAATCTTATCGATAAATTTCCATCAAAGTTCTGCAGCTCTTACATACCCTTCCACTACGGAGGCAATGCGATTAAAACCCCCGATGATCCGGGTGCGATGATGATTACCTTTGCTTTGAAGCCACGGGAGGAATACCAACCCAGTGGTCATATTAACGTATCCAGAGCAAGAGAATTTTATATTAGTTGGGACACGGATTACGTGGGGTCTATCACTACGGCTGATCTTGTGGTATCGGCATCTGCTATTAACTT |  | [This study (gallardo@inia.es)](http://asf-referencelab.info/asf/images/files/publicaciones/Nix_et_al_2006.pdf) | AGTGCGTATACTTGTGCAGATACCAATGTAGACACTTGTGCAAGCATGTGTGCAGATACCAATGTAGACACCTGTGCAAGCATGTGTGCAGATACCAATGTAGATACCTGTGCAAGCACTTGTACAAGCACAGAATAC |
| 308 | Pol14/WB-13167 | Poland | Wild pig | 2014 | KM374545 | II | [This study (gallardo@inia.es)](http://asf-referencelab.info/asf/images/files/publicaciones/Gallardo-et-al-2009a.pdf) | ATGCAGCCCACTCACCACGCAGAGATAAGCTTTCAGGATAGAGATACAGCTCTTCCAGACGCATGTTCATCTATATCTGATATTAGCCCCGTTACGTATCCGATCACATTACCTATTATTAAAAACATTTCCGTAACTGCTCATGGTATCAATCTTATCGATAAATTTCCATCAAAGTTCTGCAGCTCTTACATACCCTTCCACTACGGAGGCAATGCGATTAAAACCCCCGATGATCCGGGTGCGATGATGATTACCTTTGCTTTGAAGCCACGGGAGGAATACCAACCCAGTGGTCATATTAACGTATCCAGAGCAAGAGAATTTTATATTAGTTGGGACACGGATTACGTGGGGTCTATCACTACGGCTGATCTTGTGGTATCGGCATCTGCTATTAACTT |  | [This study (gallardo@inia.es)](http://asf-referencelab.info/asf/images/files/publicaciones/Nix_et_al_2006.pdf) | AGTGCGTATACTTGTGCAGATACCAATGTAGACACTTGTGCAAGCATGTGTGCAGATACCAATGTAGACACCTGTGCAAGCATGTGTGCAGATACCAATGTAGATACCTGTGCAAGCACTTGTACAAGCACAGAATAC |
| 309 | Lv14/DP/Robez1 | Latvia | DP | 2014 | KM374549 | II | [This study (gallardo@inia.es)](http://asf-referencelab.info/asf/images/files/publicaciones/Gallardo-et-al-2009a.pdf) | ATGCAGCCCACTCACCACGCAGAGATAAGCTTTCAGGATAGAGATACAGCTCTTCCAGACGCATGTTCATCTATATCTGATATTAGCCCCGTTACGTATCCGATCACATTACCTATTATTAAAAACATTTCCGTAACTGCTCATGGTATCAATCTTATCGATAAATTTCCATCAAAGTTCTGCAGCTCTTACATACCCTTCCACTACGGAGGCAATGCGATTAAAACCCCCGATGATCCGGGTGCGATGATGATTACCTTTGCTTTGAAGCCACGGGAGGAATACCAACCCAGTGGTCATATTAACGTATCCAGAGCAAGAGAATTTTATATTAGTTGGGACACGGATTACGTGGGGTCTATCACTACGGCTGATCTTGTGGTATCGGCATCTGCTATTAACTT |  | [This study (gallardo@inia.es)](http://asf-referencelab.info/asf/images/files/publicaciones/Nix_et_al_2006.pdf) | AGTGCGTATACTTGTGCAGATACCAATGTAGACACTTGTGCAAGCATGTGTGCAGATACCAATGTAGACACCTGTGCAAGCATGTGTGCAGATACCAATGTAGATACCTGTGCAAGCACTTGTACAAGCACAGAATAC |
| 310 | Lv14/DP/Robez2 | Latvia | DP | 2014 | KM374550 | II | [This study (gallardo@inia.es)](http://asf-referencelab.info/asf/images/files/publicaciones/Gallardo-et-al-2009a.pdf) | ATGCAGCCCACTCACCACGCAGAGATAAGCTTTCAGGATAGAGATACAGCTCTTCCAGACGCATGTTCATCTATATCTGATATTAGCCCCGTTACGTATCCGATCACATTACCTATTATTAAAAACATTTCCGTAACTGCTCATGGTATCAATCTTATCGATAAATTTCCATCAAAGTTCTGCAGCTCTTACATACCCTTCCACTACGGAGGCAATGCGATTAAAACCCCCGATGATCCGGGTGCGATGATGATTACCTTTGCTTTGAAGCCACGGGAGGAATACCAACCCAGTGGTCATATTAACGTATCCAGAGCAAGAGAATTTTATATTAGTTGGGACACGGATTACGTGGGGTCTATCACTACGGCTGATCTTGTGGTATCGGCATCTGCTATTAACTT |  | [This study (gallardo@inia.es)](http://asf-referencelab.info/asf/images/files/publicaciones/Nix_et_al_2006.pdf) | AGTGCGTATACTTGTGCAGATACCAATGTAGACACTTGTGCAAGCATGTGTGCAGATACCAATGTAGACACCTGTGCAAGCATGTGTGCAGATACCAATGTAGATACCTGTGCAAGCACTTGTACAAGCACAGAATAC |
| 311 | Lv14/DP/Robez3 | Latvia | DP | 2014 | KM374551 | II | [This study (gallardo@inia.es)](http://asf-referencelab.info/asf/images/files/publicaciones/Gallardo-et-al-2009a.pdf) | ATGCAGCCCACTCACCACGCAGAGATAAGCTTTCAGGATAGAGATACAGCTCTTCCAGACGCATGTTCATCTATATCTGATATTAGCCCCGTTACGTATCCGATCACATTACCTATTATTAAAAACATTTCCGTAACTGCTCATGGTATCAATCTTATCGATAAATTTCCATCAAAGTTCTGCAGCTCTTACATACCCTTCCACTACGGAGGCAATGCGATTAAAACCCCCGATGATCCGGGTGCGATGATGATTACCTTTGCTTTGAAGCCACGGGAGGAATACCAACCCAGTGGTCATATTAACGTATCCAGAGCAAGAGAATTTTATATTAGTTGGGACACGGATTACGTGGGGTCTATCACTACGGCTGATCTTGTGGTATCGGCATCTGCTATTAACTT |  | [This study (gallardo@inia.es)](http://asf-referencelab.info/asf/images/files/publicaciones/Nix_et_al_2006.pdf) | AGTGCGTATACTTGTGCAGATACCAATGTAGACACTTGTGCAAGCATGTGTGCAGATACCAATGTAGACACCTGTGCAAGCATGTGTGCAGATACCAATGTAGATACCTGTGCAAGCACTTGTACAAGCACAGAATAC |
| 312 | Lv14/WB/Kepo1 | Latvia | Wild pig | 2014 | KM374552 | II | [This study (gallardo@inia.es)](http://asf-referencelab.info/asf/images/files/publicaciones/Gallardo-et-al-2009a.pdf) | ATGCAGCCCACTCACCACGCAGAGATAAGCTTTCAGGATAGAGATACAGCTCTTCCAGACGCATGTTCATCTATATCTGATATTAGCCCCGTTACGTATCCGATCACATTACCTATTATTAAAAACATTTCCGTAACTGCTCATGGTATCAATCTTATCGATAAATTTCCATCAAAGTTCTGCAGCTCTTACATACCCTTCCACTACGGAGGCAATGCGATTAAAACCCCCGATGATCCGGGTGCGATGATGATTACCTTTGCTTTGAAGCCACGGGAGGAATACCAACCCAGTGGTCATATTAACGTATCCAGAGCAAGAGAATTTTATATTAGTTGGGACACGGATTACGTGGGGTCTATCACTACGGCTGATCTTGTGGTATCGGCATCTGCTATTAACTT |  | [This study (gallardo@inia.es)](http://asf-referencelab.info/asf/images/files/publicaciones/Nix_et_al_2006.pdf) | AGTGCGTATACTTGTGCAGATACCAATGTAGACACTTGTGCAAGCATGTGTGCAGATACCAATGTAGACACCTGTGCAAGCATGTGTGCAGATACCAATGTAGATACCTGTGCAAGCACTTGTACAAGCACAGAATAC |
| 313 | Lv14/WB/Kepo2 | Latvia | Wild pig | 2014 | KM374553 | II | [This study (gallardo@inia.es)](http://asf-referencelab.info/asf/images/files/publicaciones/Gallardo-et-al-2009a.pdf) | ATGCAGCCCACTCACCACGCAGAGATAAGCTTTCAGGATAGAGATACAGCTCTTCCAGACGCATGTTCATCTATATCTGATATTAGCCCCGTTACGTATCCGATCACATTACCTATTATTAAAAACATTTCCGTAACTGCTCATGGTATCAATCTTATCGATAAATTTCCATCAAAGTTCTGCAGCTCTTACATACCCTTCCACTACGGAGGCAATGCGATTAAAACCCCCGATGATCCGGGTGCGATGATGATTACCTTTGCTTTGAAGCCACGGGAGGAATACCAACCCAGTGGTCATATTAACGTATCCAGAGCAAGAGAATTTTATATTAGTTGGGACACGGATTACGTGGGGTCTATCACTACGGCTGATCTTGTGGTATCGGCATCTGCTATTAACTT |  | [This study (gallardo@inia.es)](http://asf-referencelab.info/asf/images/files/publicaciones/Nix_et_al_2006.pdf) | AGTGCGTATACTTGTGCAGATACCAATGTAGACACTTGTGCAAGCATGTGTGCAGATACCAATGTAGACACCTGTGCAAGCATGTGTGCAGATACCAATGTAGATACCTGTGCAAGCACTTGTACAAGCACAGAATAC |
| 314 | Pol14/WB-13713 | Poland | Wild pig | 2014 | KM374546 | II | [This study (gallardo@inia.es)](http://asf-referencelab.info/asf/images/files/publicaciones/Gallardo-et-al-2009a.pdf) | ATGCAGCCCACTCACCACGCAGAGATAAGCTTTCAGGATAGAGATACAGCTCTTCCAGACGCATGTTCATCTATATCTGATATTAGCCCCGTTACGTATCCGATCACATTACCTATTATTAAAAACATTTCCGTAACTGCTCATGGTATCAATCTTATCGATAAATTTCCATCAAAGTTCTGCAGCTCTTACATACCCTTCCACTACGGAGGCAATGCGATTAAAACCCCCGATGATCCGGGTGCGATGATGATTACCTTTGCTTTGAAGCCACGGGAGGAATACCAACCCAGTGGTCATATTAACGTATCCAGAGCAAGAGAATTTTATATTAGTTGGGACACGGATTACGTGGGGTCTATCACTACGGCTGATCTTGTGGTATCGGCATCTGCTATTAACTT |  | [This study (gallardo@inia.es)](http://asf-referencelab.info/asf/images/files/publicaciones/Nix_et_al_2006.pdf) | AGTGCGTATACTTGTGCAGATACCAATGTAGACACTTGTGCAAGCATGTGTGCAGATACCAATGTAGACACCTGTGCAAGCATGTGTGCAGATACCAATGTAGATACCTGTGCAAGCACTTGTACAAGCACAGAATAC |
| 315 | Lv14/WB/Kepo3 | Latvia | Wild pig | 2014 | KM374554 | II | [This study (gallardo@inia.es)](http://asf-referencelab.info/asf/images/files/publicaciones/Gallardo-et-al-2009a.pdf) | ATGCAGCCCACTCACCACGCAGAGATAAGCTTTCAGGATAGAGATACAGCTCTTCCAGACGCATGTTCATCTATATCTGATATTAGCCCCGTTACGTATCCGATCACATTACCTATTATTAAAAACATTTCCGTAACTGCTCATGGTATCAATCTTATCGATAAATTTCCATCAAAGTTCTGCAGCTCTTACATACCCTTCCACTACGGAGGCAATGCGATTAAAACCCCCGATGATCCGGGTGCGATGATGATTACCTTTGCTTTGAAGCCACGGGAGGAATACCAACCCAGTGGTCATATTAACGTATCCAGAGCAAGAGAATTTTATATTAGTTGGGACACGGATTACGTGGGGTCTATCACTACGGCTGATCTTGTGGTATCGGCATCTGCTATTAACTT |  | [This study (gallardo@inia.es)](http://asf-referencelab.info/asf/images/files/publicaciones/Nix_et_al_2006.pdf) | AGTGCGTATACTTGTGCAGATACCAATGTAGACACTTGTGCAAGCATGTGTGCAGATACCAATGTAGACACCTGTGCAAGCATGTGTGCAGATACCAATGTAGATACCTGTGCAAGCACTTGTACAAGCACAGAATAC |
| 316 | Pol14/WB-13784 | Poland | Wild pig | 2014 | KM374547 | II | [This study (gallardo@inia.es)](http://asf-referencelab.info/asf/images/files/publicaciones/Gallardo-et-al-2009a.pdf) | ATGCAGCCCACTCACCACGCAGAGATAAGCTTTCAGGATAGAGATACAGCTCTTCCAGACGCATGTTCATCTATATCTGATATTAGCCCCGTTACGTATCCGATCACATTACCTATTATTAAAAACATTTCCGTAACTGCTCATGGTATCAATCTTATCGATAAATTTCCATCAAAGTTCTGCAGCTCTTACATACCCTTCCACTACGGAGGCAATGCGATTAAAACCCCCGATGATCCGGGTGCGATGATGATTACCTTTGCTTTGAAGCCACGGGAGGAATACCAACCCAGTGGTCATATTAACGTATCCAGAGCAAGAGAATTTTATATTAGTTGGGACACGGATTACGTGGGGTCTATCACTACGGCTGATCTTGTGGTATCGGCATCTGCTATTAACTT |  | [This study (gallardo@inia.es)](http://asf-referencelab.info/asf/images/files/publicaciones/Nix_et_al_2006.pdf) | AGTGCGTATACTTGTGCAGATACCAATGTAGACACTTGTGCAAGCATGTGTGCAGATACCAATGTAGACACCTGTGCAAGCATGTGTGCAGATACCAATGTAGATACCTGTGCAAGCACTTGTACAAGCACAGAATAC |
| 317 | Pol14/WB-13785 | Poland | Wild pig | 2014 | KM374548 | II | [This study (gallardo@inia.es)](http://asf-referencelab.info/asf/images/files/publicaciones/Gallardo-et-al-2009a.pdf) | ATGCAGCCCACTCACCACGCAGAGATAAGCTTTCAGGATAGAGATACAGCTCTTCCAGACGCATGTTCATCTATATCTGATATTAGCCCCGTTACGTATCCGATCACATTACCTATTATTAAAAACATTTCCGTAACTGCTCATGGTATCAATCTTATCGATAAATTTCCATCAAAGTTCTGCAGCTCTTACATACCCTTCCACTACGGAGGCAATGCGATTAAAACCCCCGATGATCCGGGTGCGATGATGATTACCTTTGCTTTGAAGCCACGGGAGGAATACCAACCCAGTGGTCATATTAACGTATCCAGAGCAAGAGAATTTTATATTAGTTGGGACACGGATTACGTGGGGTCTATCACTACGGCTGATCTTGTGGTATCGGCATCTGCTATTAACTT |  | [This study (gallardo@inia.es)](http://asf-referencelab.info/asf/images/files/publicaciones/Nix_et_al_2006.pdf) | AGTGCGTATACTTGTGCAGATACCAATGTAGACACTTGTGCAAGCATGTGTGCAGATACCAATGTAGACACCTGTGCAAGCATGTGTGCAGATACCAATGTAGATACCTGTGCAAGCACTTGTACAAGCACAGAATAC |
| 318 | Lv14/DP/Pied | Latvia | DP | 2014 | KM374557 | II | [This study (gallardo@inia.es)](http://asf-referencelab.info/asf/images/files/publicaciones/Gallardo-et-al-2009a.pdf) | ATGCAGCCCACTCACCACGCAGAGATAAGCTTTCAGGATAGAGATACAGCTCTTCCAGACGCATGTTCATCTATATCTGATATTAGCCCCGTTACGTATCCGATCACATTACCTATTATTAAAAACATTTCCGTAACTGCTCATGGTATCAATCTTATCGATAAATTTCCATCAAAGTTCTGCAGCTCTTACATACCCTTCCACTACGGAGGCAATGCGATTAAAACCCCCGATGATCCGGGTGCGATGATGATTACCTTTGCTTTGAAGCCACGGGAGGAATACCAACCCAGTGGTCATATTAACGTATCCAGAGCAAGAGAATTTTATATTAGTTGGGACACGGATTACGTGGGGTCTATCACTACGGCTGATCTTGTGGTATCGGCATCTGCTATTAACTT |  | [This study (gallardo@inia.es)](http://asf-referencelab.info/asf/images/files/publicaciones/Nix_et_al_2006.pdf) | AGTGCGTATACTTGTGCAGATACCAATGTAGACACTTGTGCAAGCATGTGTGCAGATACCAATGTAGACACCTGTGCAAGCATGTGTGCAGATACCAATGTAGATACCTGTGCAAGCACTTGTACAAGCACAGAATAC |
| 319 | Lv14/WB/Indras1 | Latvia | Wild pig | 2014 | KM374555 | II | [This study (gallardo@inia.es)](http://asf-referencelab.info/asf/images/files/publicaciones/Gallardo-et-al-2009a.pdf) | ATGCAGCCCACTCACCACGCAGAGATAAGCTTTCAGGATAGAGATACAGCTCTTCCAGACGCATGTTCATCTATATCTGATATTAGCCCCGTTACGTATCCGATCACATTACCTATTATTAAAAACATTTCCGTAACTGCTCATGGTATCAATCTTATCGATAAATTTCCATCAAAGTTCTGCAGCTCTTACATACCCTTCCACTACGGAGGCAATGCGATTAAAACCCCCGATGATCCGGGTGCGATGATGATTACCTTTGCTTTGAAGCCACGGGAGGAATACCAACCCAGTGGTCATATTAACGTATCCAGAGCAAGAGAATTTTATATTAGTTGGGACACGGATTACGTGGGGTCTATCACTACGGCTGATCTTGTGGTATCGGCATCTGCTATTAACTT |  | [This study (gallardo@inia.es)](http://asf-referencelab.info/asf/images/files/publicaciones/Nix_et_al_2006.pdf) | AGTGCGTATACTTGTGCAGATACCAATGTAGACACTTGTGCAAGCATGTGTGCAGATACCAATGTAGACACCTGTGCAAGCATGTGTGCAGATACCAATGTAGATACCTGTGCAAGCACTTGTACAAGCACAGAATAC |
| 320 | Lv14/WB/Indras2 | Latvia | Wild pig | 2014 | KM374556 | II | [This study (gallardo@inia.es)](http://asf-referencelab.info/asf/images/files/publicaciones/Gallardo-et-al-2009a.pdf) | ATGCAGCCCACTCACCACGCAGAGATAAGCTTTCAGGATAGAGATACAGCTCTTCCAGACGCATGTTCATCTATATCTGATATTAGCCCCGTTACGTATCCGATCACATTACCTATTATTAAAAACATTTCCGTAACTGCTCATGGTATCAATCTTATCGATAAATTTCCATCAAAGTTCTGCAGCTCTTACATACCCTTCCACTACGGAGGCAATGCGATTAAAACCCCCGATGATCCGGGTGCGATGATGATTACCTTTGCTTTGAAGCCACGGGAGGAATACCAACCCAGTGGTCATATTAACGTATCCAGAGCAAGAGAATTTTATATTAGTTGGGACACGGATTACGTGGGGTCTATCACTACGGCTGATCTTGTGGTATCGGCATCTGCTATTAACTT |  | [This study (gallardo@inia.es)](http://asf-referencelab.info/asf/images/files/publicaciones/Nix_et_al_2006.pdf) | AGTGCGTATACTTGTGCAGATACCAATGTAGACACTTGTGCAAGCATGTGTGCAGATACCAATGTAGACACCTGTGCAAGCATGTGTGCAGATACCAATGTAGATACCTGTGCAAGCACTTGTACAAGCACAGAATAC |
| 321 | Pol14/WB-14309#7 | Poland | Wild pig | 2014 |  | II | [This study (gallardo@inia.es)](http://asf-referencelab.info/asf/images/files/publicaciones/Gallardo-et-al-2009a.pdf) | ATGCAGCCCACTCACCACGCAGAGATAAGCTTTCAGGATAGAGATACAGCTCTTCCAGACGCATGTTCATCTATATCTGATATTAGCCCCGTTACGTATCCGATCACATTACCTATTATTAAAAACATTTCCGTAACTGCTCATGGTATCAATCTTATCGATAAATTTCCATCAAAGTTCTGCAGCTCTTACATACCCTTCCACTACGGAGGCAATGCGATTAAAACCCCCGATGATCCGGGTGCGATGATGATTACCTTTGCTTTGAAGCCACGGGAGGAATACCAACCCAGTGGTCATATTAACGTATCCAGAGCAAGAGAATTTTATATTAGTTGGGACACGGATTACGTGGGGTCTATCACTACGGCTGATCTTGTGGTATCGGCATCTGCTATTAACTT |  | [This study (gallardo@inia.es)](http://asf-referencelab.info/asf/images/files/publicaciones/Nix_et_al_2006.pdf) | AGTGCGTATACTTGTGCAGATACCAATGTAGACACTTGTGCAAGCATGTGTGCAGATACCAATGTAGACACCTGTGCAAGCATGTGTGCAGATACCAATGTAGATACCTGTGCAAGCACTTGTACAAGCACAGAATAC |
| 322 | Pol14/WB-14310#7 | Poland | Wild pig | 2014 |  | II | [This study (gallardo@inia.es)](http://asf-referencelab.info/asf/images/files/publicaciones/Gallardo-et-al-2009a.pdf) | ATGCAGCCCACTCACCACGCAGAGATAAGCTTTCAGGATAGAGATACAGCTCTTCCAGACGCATGTTCATCTATATCTGATATTAGCCCCGTTACGTATCCGATCACATTACCTATTATTAAAAACATTTCCGTAACTGCTCATGGTATCAATCTTATCGATAAATTTCCATCAAAGTTCTGCAGCTCTTACATACCCTTCCACTACGGAGGCAATGCGATTAAAACCCCCGATGATCCGGGTGCGATGATGATTACCTTTGCTTTGAAGCCACGGGAGGAATACCAACCCAGTGGTCATATTAACGTATCCAGAGCAAGAGAATTTTATATTAGTTGGGACACGGATTACGTGGGGTCTATCACTACGGCTGATCTTGTGGTATCGGCATCTGCTATTAACTT |  | [This study (gallardo@inia.es)](http://asf-referencelab.info/asf/images/files/publicaciones/Nix_et_al_2006.pdf) | AGTGCGTATACTTGTGCAGATACCAATGTAGACACTTGTGCAAGCATGTGTGCAGATACCAATGTAGACACCTGTGCAAGCATGTGTGCAGATACCAATGTAGATACCTGTGCAAGCACTTGTACAAGCACAGAATAC |
| 323 | Pol14/WB-14311#7 | Poland | Wild pig | 2014 |  | II | [This study (gallardo@inia.es)](http://asf-referencelab.info/asf/images/files/publicaciones/Gallardo-et-al-2009a.pdf) | ATGCAGCCCACTCACCACGCAGAGATAAGCTTTCAGGATAGAGATACAGCTCTTCCAGACGCATGTTCATCTATATCTGATATTAGCCCCGTTACGTATCCGATCACATTACCTATTATTAAAAACATTTCCGTAACTGCTCATGGTATCAATCTTATCGATAAATTTCCATCAAAGTTCTGCAGCTCTTACATACCCTTCCACTACGGAGGCAATGCGATTAAAACCCCCGATGATCCGGGTGCGATGATGATTACCTTTGCTTTGAAGCCACGGGAGGAATACCAACCCAGTGGTCATATTAACGTATCCAGAGCAAGAGAATTTTATATTAGTTGGGACACGGATTACGTGGGGTCTATCACTACGGCTGATCTTGTGGTATCGGCATCTGCTATTAACTT |  | [This study (gallardo@inia.es)](http://asf-referencelab.info/asf/images/files/publicaciones/Nix_et_al_2006.pdf) | AGTGCGTATACTTGTGCAGATACCAATGTAGACACTTGTGCAAGCATGTGTGCAGATACCAATGTAGACACCTGTGCAAGCATGTGTGCAGATACCAATGTAGATACCTGTGCAAGCACTTGTACAAGCACAGAATAC |
| 324 | Pol14/WB-14312#7 | Poland | Wild pig | 2014 |  | II | [This study (gallardo@inia.es)](http://asf-referencelab.info/asf/images/files/publicaciones/Gallardo-et-al-2009a.pdf) | ATGCAGCCCACTCACCACGCAGAGATAAGCTTTCAGGATAGAGATACAGCTCTTCCAGACGCATGTTCATCTATATCTGATATTAGCCCCGTTACGTATCCGATCACATTACCTATTATTAAAAACATTTCCGTAACTGCTCATGGTATCAATCTTATCGATAAATTTCCATCAAAGTTCTGCAGCTCTTACATACCCTTCCACTACGGAGGCAATGCGATTAAAACCCCCGATGATCCGGGTGCGATGATGATTACCTTTGCTTTGAAGCCACGGGAGGAATACCAACCCAGTGGTCATATTAACGTATCCAGAGCAAGAGAATTTTATATTAGTTGGGACACGGATTACGTGGGGTCTATCACTACGGCTGATCTTGTGGTATCGGCATCTGCTATTAACTT |  | [This study (gallardo@inia.es)](http://asf-referencelab.info/asf/images/files/publicaciones/Nix_et_al_2006.pdf) | AGTGCGTATACTTGTGCAGATACCAATGTAGACACTTGTGCAAGCATGTGTGCAGATACCAATGTAGACACCTGTGCAAGCATGTGTGCAGATACCAATGTAGATACCTGTGCAAGCACTTGTACAAGCACAGAATAC |
| 325 | Pol14/WB-14313#7 | Poland | Wild pig | 2014 |  | II | [This study (gallardo@inia.es)](http://asf-referencelab.info/asf/images/files/publicaciones/Gallardo-et-al-2009a.pdf) | ATGCAGCCCACTCACCACGCAGAGATAAGCTTTCAGGATAGAGATACAGCTCTTCCAGACGCATGTTCATCTATATCTGATATTAGCCCCGTTACGTATCCGATCACATTACCTATTATTAAAAACATTTCCGTAACTGCTCATGGTATCAATCTTATCGATAAATTTCCATCAAAGTTCTGCAGCTCTTACATACCCTTCCACTACGGAGGCAATGCGATTAAAACCCCCGATGATCCGGGTGCGATGATGATTACCTTTGCTTTGAAGCCACGGGAGGAATACCAACCCAGTGGTCATATTAACGTATCCAGAGCAAGAGAATTTTATATTAGTTGGGACACGGATTACGTGGGGTCTATCACTACGGCTGATCTTGTGGTATCGGCATCTGCTATTAACTT |  | [This study (gallardo@inia.es)](http://asf-referencelab.info/asf/images/files/publicaciones/Nix_et_al_2006.pdf) | AGTGCGTATACTTGTGCAGATACCAATGTAGACACTTGTGCAAGCATGTGTGCAGATACCAATGTAGACACCTGTGCAAGCATGTGTGCAGATACCAATGTAGATACCTGTGCAAGCACTTGTACAAGCACAGAATAC |
| 326 | Pol14/WB-14314#7 | Poland | Wild pig | 2014 |  | II | [This study (gallardo@inia.es)](http://asf-referencelab.info/asf/images/files/publicaciones/Gallardo-et-al-2009a.pdf) | ATGCAGCCCACTCACCACGCAGAGATAAGCTTTCAGGATAGAGATACAGCTCTTCCAGACGCATGTTCATCTATATCTGATATTAGCCCCGTTACGTATCCGATCACATTACCTATTATTAAAAACATTTCCGTAACTGCTCATGGTATCAATCTTATCGATAAATTTCCATCAAAGTTCTGCAGCTCTTACATACCCTTCCACTACGGAGGCAATGCGATTAAAACCCCCGATGATCCGGGTGCGATGATGATTACCTTTGCTTTGAAGCCACGGGAGGAATACCAACCCAGTGGTCATATTAACGTATCCAGAGCAAGAGAATTTTATATTAGTTGGGACACGGATTACGTGGGGTCTATCACTACGGCTGATCTTGTGGTATCGGCATCTGCTATTAACTT |  | [This study (gallardo@inia.es)](http://asf-referencelab.info/asf/images/files/publicaciones/Nix_et_al_2006.pdf) | AGTGCGTATACTTGTGCAGATACCAATGTAGACACTTGTGCAAGCATGTGTGCAGATACCAATGTAGACACCTGTGCAAGCATGTGTGCAGATACCAATGTAGATACCTGTGCAAGCACTTGTACAAGCACAGAATAC |
| 327 | Pol14/WB-14473#8 | Poland | Wild pig | 2014 |  | II | [This study (gallardo@inia.es)](http://asf-referencelab.info/asf/images/files/publicaciones/Gallardo-et-al-2009a.pdf) | ATGCAGCCCACTCACCACGCAGAGATAAGCTTTCAGGATAGAGATACAGCTCTTCCAGACGCATGTTCATCTATATCTGATATTAGCCCCGTTACGTATCCGATCACATTACCTATTATTAAAAACATTTCCGTAACTGCTCATGGTATCAATCTTATCGATAAATTTCCATCAAAGTTCTGCAGCTCTTACATACCCTTCCACTACGGAGGCAATGCGATTAAAACCCCCGATGATCCGGGTGCGATGATGATTACCTTTGCTTTGAAGCCACGGGAGGAATACCAACCCAGTGGTCATATTAACGTATCCAGAGCAAGAGAATTTTATATTAGTTGGGACACGGATTACGTGGGGTCTATCACTACGGCTGATCTTGTGGTATCGGCATCTGCTATTAACTT |  | [This study (gallardo@inia.es)](http://asf-referencelab.info/asf/images/files/publicaciones/Nix_et_al_2006.pdf) | AGTGCGTATACTTGTGCAGATACCAATGTAGACACTTGTGCAAGCATGTGTGCAGATACCAATGTAGACACCTGTGCAAGCATGTGTGCAGATACCAATGTAGATACCTGTGCAAGCACTTGTACAAGCACAGAATAC |
| 328 | Lv14/WB/VALKA 1 | Latvia | Wild pig | 2014 |  | II | [This study (gallardo@inia.es)](http://asf-referencelab.info/asf/images/files/publicaciones/Gallardo-et-al-2009a.pdf) | ATGCAGCCCACTCACCACGCAGAGATAAGCTTTCAGGATAGAGATACAGCTCTTCCAGACGCATGTTCATCTATATCTGATATTAGCCCCGTTACGTATCCGATCACATTACCTATTATTAAAAACATTTCCGTAACTGCTCATGGTATCAATCTTATCGATAAATTTCCATCAAAGTTCTGCAGCTCTTACATACCCTTCCACTACGGAGGCAATGCGATTAAAACCCCCGATGATCCGGGTGCGATGATGATTACCTTTGCTTTGAAGCCACGGGAGGAATACCAACCCAGTGGTCATATTAACGTATCCAGAGCAAGAGAATTTTATATTAGTTGGGACACGGATTACGTGGGGTCTATCACTACGGCTGATCTTGTGGTATCGGCATCTGCTATTAACTT |  | [This study (gallardo@inia.es)](http://asf-referencelab.info/asf/images/files/publicaciones/Nix_et_al_2006.pdf) | AGTGCGTATACTTGTGCAGATACCAATGTAGACACTTGTGCAAGCATGTGTGCAGATACCAATGTAGACACCTGTGCAAGCATGTGTGCAGATACCAATGTAGATACCTGTGCAAGCACTTGTACAAGCACAGAATAC |
| 329 | Pol14/WB-15094#9 | Poland | Wild pig | 2014 |  | II | [This study (gallardo@inia.es)](http://asf-referencelab.info/asf/images/files/publicaciones/Gallardo-et-al-2009a.pdf) | ATGCAGCCCACTCACCACGCAGAGATAAGCTTTCAGGATAGAGATACAGCTCTTCCAGACGCATGTTCATCTATATCTGATATTAGCCCCGTTACGTATCCGATCACATTACCTATTATTAAAAACATTTCCGTAACTGCTCATGGTATCAATCTTATCGATAAATTTCCATCAAAGTTCTGCAGCTCTTACATACCCTTCCACTACGGAGGCAATGCGATTAAAACCCCCGATGATCCGGGTGCGATGATGATTACCTTTGCTTTGAAGCCACGGGAGGAATACCAACCCAGTGGTCATATTAACGTATCCAGAGCAAGAGAATTTTATATTAGTTGGGACACGGATTACGTGGGGTCTATCACTACGGCTGATCTTGTGGTATCGGCATCTGCTATTAACTT |  | [This study (gallardo@inia.es)](http://asf-referencelab.info/asf/images/files/publicaciones/Nix_et_al_2006.pdf) | AGTGCGTATACTTGTGCAGATACCAATGTAGACACTTGTGCAAGCATGTGTGCAGATACCAATGTAGACACCTGTGCAAGCATGTGTGCAGATACCAATGTAGATACCTGTGCAAGCACTTGTACAAGCACAGAATAC |
| 330 | Lv14/DP/SKA-1 | Latvia | DP | 2014 |  | II | [This study (gallardo@inia.es)](http://asf-referencelab.info/asf/images/files/publicaciones/Gallardo-et-al-2009a.pdf) | ATGCAGCCCACTCACCACGCAGAGATAAGCTTTCAGGATAGAGATACAGCTCTTCCAGACGCATGTTCATCTATATCTGATATTAGCCCCGTTACGTATCCGATCACATTACCTATTATTAAAAACATTTCCGTAACTGCTCATGGTATCAATCTTATCGATAAATTTCCATCAAAGTTCTGCAGCTCTTACATACCCTTCCACTACGGAGGCAATGCGATTAAAACCCCCGATGATCCGGGTGCGATGATGATTACCTTTGCTTTGAAGCCACGGGAGGAATACCAACCCAGTGGTCATATTAACGTATCCAGAGCAAGAGAATTTTATATTAGTTGGGACACGGATTACGTGGGGTCTATCACTACGGCTGATCTTGTGGTATCGGCATCTGCTATTAACTT |  | [This study (gallardo@inia.es)](http://asf-referencelab.info/asf/images/files/publicaciones/Nix_et_al_2006.pdf) | AGTGCGTATACTTGTGCAGATACCAATGTAGACACTTGTGCAAGCATGTGTGCAGATACCAATGTAGACACCTGTGCAAGCATGTGTGCAGATACCAATGTAGATACCTGTGCAAGCACTTGTACAAGCACAGAATAC |
| 331 | Pol14/DP-15590 | Poland | DP | 2014 | KM374558 | II | [This study (gallardo@inia.es)](http://asf-referencelab.info/asf/images/files/publicaciones/Gallardo-et-al-2009a.pdf) | ATGCAGCCCACTCACCACGCAGAGATAAGCTTTCAGGATAGAGATACAGCTCTTCCAGACGCATGTTCATCTATATCTGATATTAGCCCCGTTACGTATCCGATCACATTACCTATTATTAAAAACATTTCCGTAACTGCTCATGGTATCAATCTTATCGATAAATTTCCATCAAAGTTCTGCAGCTCTTACATACCCTTCCACTACGGAGGCAATGCGATTAAAACCCCCGATGATCCGGGTGCGATGATGATTACCTTTGCTTTGAAGCCACGGGAGGAATACCAACCCAGTGGTCATATTAACGTATCCAGAGCAAGAGAATTTTATATTAGTTGGGACACGGATTACGTGGGGTCTATCACTACGGCTGATCTTGTGGTATCGGCATCTGCTATTAACTT |  | [This study (gallardo@inia.es)](http://asf-referencelab.info/asf/images/files/publicaciones/Nix_et_al_2006.pdf) | AGTGCGTATACTTGTGCAGATACCAATGTAGACACTTGTGCAAGCATGTGTGCAGATACCAATGTAGACACCTGTGCAAGCATGTGTGCAGATACCAATGTAGATACCTGTGCAAGCACTTGTACAAGCACAGAATAC |
| 332 | Pol14/DP-15591 | Poland | DP | 2014 | KM374559 | II | [This study (gallardo@inia.es)](http://asf-referencelab.info/asf/images/files/publicaciones/Gallardo-et-al-2009a.pdf) | ATGCAGCCCACTCACCACGCAGAGATAAGCTTTCAGGATAGAGATACAGCTCTTCCAGACGCATGTTCATCTATATCTGATATTAGCCCCGTTACGTATCCGATCACATTACCTATTATTAAAAACATTTCCGTAACTGCTCATGGTATCAATCTTATCGATAAATTTCCATCAAAGTTCTGCAGCTCTTACATACCCTTCCACTACGGAGGCAATGCGATTAAAACCCCCGATGATCCGGGTGCGATGATGATTACCTTTGCTTTGAAGCCACGGGAGGAATACCAACCCAGTGGTCATATTAACGTATCCAGAGCAAGAGAATTTTATATTAGTTGGGACACGGATTACGTGGGGTCTATCACTACGGCTGATCTTGTGGTATCGGCATCTGCTATTAACTT |  | [This study (gallardo@inia.es)](http://asf-referencelab.info/asf/images/files/publicaciones/Nix_et_al_2006.pdf) | AGTGCGTATACTTGTGCAGATACCAATGTAGACACTTGTGCAAGCATGTGTGCAGATACCAATGTAGACACCTGTGCAAGCATGTGTGCAGATACCAATGTAGATACCTGTGCAAGCACTTGTACAAGCACAGAATAC |
| 333 | Pol14/DP-15747-1 | Poland | DP | 2014 | KM374560 | II | [This study (gallardo@inia.es)](http://asf-referencelab.info/asf/images/files/publicaciones/Gallardo-et-al-2009a.pdf) | ATGCAGCCCACTCACCACGCAGAGATAAGCTTTCAGGATAGAGATACAGCTCTTCCAGACGCATGTTCATCTATATCTGATATTAGCCCCGTTACGTATCCGATCACATTACCTATTATTAAAAACATTTCCGTAACTGCTCATGGTATCAATCTTATCGATAAATTTCCATCAAAGTTCTGCAGCTCTTACATACCCTTCCACTACGGAGGCAATGCGATTAAAACCCCCGATGATCCGGGTGCGATGATGATTACCTTTGCTTTGAAGCCACGGGAGGAATACCAACCCAGTGGTCATATTAACGTATCCAGAGCAAGAGAATTTTATATTAGTTGGGACACGGATTACGTGGGGTCTATCACTACGGCTGATCTTGTGGTATCGGCATCTGCTATTAACTT |  | [This study (gallardo@inia.es)](http://asf-referencelab.info/asf/images/files/publicaciones/Nix_et_al_2006.pdf) | AGTGCGTATACTTGTGCAGATACCAATGTAGACACTTGTGCAAGCATGTGTGCAGATACCAATGTAGACACCTGTGCAAGCATGTGTGCAGATACCAATGTAGATACCTGTGCAAGCACTTGTACAAGCACAGAATAC |
| 334 | Pol14/DP-15747-2 | Poland | DP | 2014 | KM374561 | II | [This study (gallardo@inia.es)](http://asf-referencelab.info/asf/images/files/publicaciones/Gallardo-et-al-2009a.pdf) | ATGCAGCCCACTCACCACGCAGAGATAAGCTTTCAGGATAGAGATACAGCTCTTCCAGACGCATGTTCATCTATATCTGATATTAGCCCCGTTACGTATCCGATCACATTACCTATTATTAAAAACATTTCCGTAACTGCTCATGGTATCAATCTTATCGATAAATTTCCATCAAAGTTCTGCAGCTCTTACATACCCTTCCACTACGGAGGCAATGCGATTAAAACCCCCGATGATCCGGGTGCGATGATGATTACCTTTGCTTTGAAGCCACGGGAGGAATACCAACCCAGTGGTCATATTAACGTATCCAGAGCAAGAGAATTTTATATTAGTTGGGACACGGATTACGTGGGGTCTATCACTACGGCTGATCTTGTGGTATCGGCATCTGCTATTAACTT |  | [This study (gallardo@inia.es)](http://asf-referencelab.info/asf/images/files/publicaciones/Nix_et_al_2006.pdf) | AGTGCGTATACTTGTGCAGATACCAATGTAGACACTTGTGCAAGCATGTGTGCAGATACCAATGTAGACACCTGTGCAAGCATGTGTGCAGATACCAATGTAGATACCTGTGCAAGCACTTGTACAAGCACAGAATAC |
| 335 | Pol14/DP-15747-3 | Poland | DP | 2014 | KM374562 | II | [This study (gallardo@inia.es)](http://asf-referencelab.info/asf/images/files/publicaciones/Gallardo-et-al-2009a.pdf) | ATGCAGCCCACTCACCACGCAGAGATAAGCTTTCAGGATAGAGATACAGCTCTTCCAGACGCATGTTCATCTATATCTGATATTAGCCCCGTTACGTATCCGATCACATTACCTATTATTAAAAACATTTCCGTAACTGCTCATGGTATCAATCTTATCGATAAATTTCCATCAAAGTTCTGCAGCTCTTACATACCCTTCCACTACGGAGGCAATGCGATTAAAACCCCCGATGATCCGGGTGCGATGATGATTACCTTTGCTTTGAAGCCACGGGAGGAATACCAACCCAGTGGTCATATTAACGTATCCAGAGCAAGAGAATTTTATATTAGTTGGGACACGGATTACGTGGGGTCTATCACTACGGCTGATCTTGTGGTATCGGCATCTGCTATTAACTT |  | [This study (gallardo@inia.es)](http://asf-referencelab.info/asf/images/files/publicaciones/Nix_et_al_2006.pdf) | AGTGCGTATACTTGTGCAGATACCAATGTAGACACTTGTGCAAGCATGTGTGCAGATACCAATGTAGACACCTGTGCAAGCATGTGTGCAGATACCAATGTAGATACCTGTGCAAGCACTTGTACAAGCACAGAATAC |
| 336 | Lt14/DP-Ignalina-1 | Lithuania | DP | 2014 | KM374563 | II | [This study (gallardo@inia.es)](file:///C:\Users\Cgallardo\Dropbox\Carmina%20AGOSTO%202014\SECUENCIAS%20CRL\Desktop\Carmina%20MARZO%202014\ARTICULOS\VPPA\GENOTIPADO\2003%20Bastos%20et%20al.pdf) | ATGCAGCCCACTCACCACGCAGAGATAAGCTTTCAGGATAGAGATACAGCTCTTCCAGACGCATGTTCATCTATATCTGATATTAGCCCCGTTACGTATCCGATCACATTACCTATTATTAAAAACATTTCCGTAACTGCTCATGGTATCAATCTTATCGATAAATTTCCATCAAAGTTCTGCAGCTCTTACATACCCTTCCACTACGGAGGCAATGCGATTAAAACCCCCGATGATCCGGGTGCGATGATGATTACCTTTGCTTTGAAGCCACGGGAGGAATACCAACCCAGTGGTCATATTAACGTATCCAGAGCAAGAGAATTTTATATTAGTTGGGACACGGATTACGTGGGGTCTATCACTACGGCTGATCTTGTGGTATCGGCATCTGCTATTAACTT |  | [This study (gallardo@inia.es)](http://asf-referencelab.info/asf/images/files/publicaciones/Nix_et_al_2006.pdf) | AGTGCGTATACTTGTGCAGATACCAATGTAGACACTTGTGCAAGCATGTGTGCAGATACCAATGTAGACACCTGTGCAAGCATGTGTGCAGATACCAATGTAGATACCTGTGCAAGCACTTGTACAAGCACAGAATAC |
| 337 | Lt14/DP-Ignalina-10 | Lithuania | DP | 2014 | KM374572 | II | [This study (gallardo@inia.es)](file:///C:\Users\Cgallardo\Dropbox\Carmina%20AGOSTO%202014\SECUENCIAS%20CRL\Desktop\Carmina%20MARZO%202014\ARTICULOS\VPPA\GENOTIPADO\2003%20Bastos%20et%20al.pdf) | ATGCAGCCCACTCACCACGCAGAGATAAGCTTTCAGGATAGAGATACAGCTCTTCCAGACGCATGTTCATCTATATCTGATATTAGCCCCGTTACGTATCCGATCACATTACCTATTATTAAAAACATTTCCGTAACTGCTCATGGTATCAATCTTATCGATAAATTTCCATCAAAGTTCTGCAGCTCTTACATACCCTTCCACTACGGAGGCAATGCGATTAAAACCCCCGATGATCCGGGTGCGATGATGATTACCTTTGCTTTGAAGCCACGGGAGGAATACCAACCCAGTGGTCATATTAACGTATCCAGAGCAAGAGAATTTTATATTAGTTGGGACACGGATTACGTGGGGTCTATCACTACGGCTGATCTTGTGGTATCGGCATCTGCTATTAACTT |  | [This study (gallardo@inia.es)](file:///C:\Users\Cgallardo\Dropbox\Carmina%20AGOSTO%202014\SECUENCIAS%20CRL\Desktop\Carmina%20MARZO%202014\ARTICULOS\VPPA\GENOTIPADO\2004%20Bastos%20et%20al.pdf) | AGTGCGTATACTTGTGCAGATACCAATGTAGACACTTGTGCAAGCATGTGTGCAGATACCAATGTAGACACCTGTGCAAGCATGTGTGCAGATACCAATGTAGATACCTGTGCAAGCACTTGTACAAGCACAGAATAC |
| 338 | Lt14/DP-Ignalina-11 | Lithuania | DP | 2014 | KM374573 | II | [This study (gallardo@inia.es)](file:///C:\Users\Cgallardo\Dropbox\Carmina%20AGOSTO%202014\SECUENCIAS%20CRL\Desktop\Carmina%20MARZO%202014\ARTICULOS\VPPA\GENOTIPADO\2003%20Bastos%20et%20al.pdf) | ATGCAGCCCACTCACCACGCAGAGATAAGCTTTCAGGATAGAGATACAGCTCTTCCAGACGCATGTTCATCTATATCTGATATTAGCCCCGTTACGTATCCGATCACATTACCTATTATTAAAAACATTTCCGTAACTGCTCATGGTATCAATCTTATCGATAAATTTCCATCAAAGTTCTGCAGCTCTTACATACCCTTCCACTACGGAGGCAATGCGATTAAAACCCCCGATGATCCGGGTGCGATGATGATTACCTTTGCTTTGAAGCCACGGGAGGAATACCAACCCAGTGGTCATATTAACGTATCCAGAGCAAGAGAATTTTATATTAGTTGGGACACGGATTACGTGGGGTCTATCACTACGGCTGATCTTGTGGTATCGGCATCTGCTATTAACTT |  | [This study (gallardo@inia.es)](file:///C:\Users\Cgallardo\Dropbox\Carmina%20AGOSTO%202014\SECUENCIAS%20CRL\Desktop\Carmina%20MARZO%202014\ARTICULOS\VPPA\GENOTIPADO\2004%20Bastos%20et%20al.pdf) | AGTGCGTATACTTGTGCAGATACCAATGTAGACACTTGTGCAAGCATGTGTGCAGATACCAATGTAGACACCTGTGCAAGCATGTGTGCAGATACCAATGTAGATACCTGTGCAAGCACTTGTACAAGCACAGAATAC |
| 339 | Lt14/DP-Ignalina-12 | Lithuania | DP | 2014 | KM374574 | II | [This study (gallardo@inia.es)](file:///C:\Users\Cgallardo\Dropbox\Carmina%20AGOSTO%202014\SECUENCIAS%20CRL\Desktop\Carmina%20MARZO%202014\ARTICULOS\VPPA\GENOTIPADO\2003%20Bastos%20et%20al.pdf) | ATGCAGCCCACTCACCACGCAGAGATAAGCTTTCAGGATAGAGATACAGCTCTTCCAGACGCATGTTCATCTATATCTGATATTAGCCCCGTTACGTATCCGATCACATTACCTATTATTAAAAACATTTCCGTAACTGCTCATGGTATCAATCTTATCGATAAATTTCCATCAAAGTTCTGCAGCTCTTACATACCCTTCCACTACGGAGGCAATGCGATTAAAACCCCCGATGATCCGGGTGCGATGATGATTACCTTTGCTTTGAAGCCACGGGAGGAATACCAACCCAGTGGTCATATTAACGTATCCAGAGCAAGAGAATTTTATATTAGTTGGGACACGGATTACGTGGGGTCTATCACTACGGCTGATCTTGTGGTATCGGCATCTGCTATTAACTT |  | [This study (gallardo@inia.es)](http://asf-referencelab.info/asf/images/files/publicaciones/Nix_et_al_2006.pdf) | AGTGCGTATACTTGTGCAGATACCAATGTAGACACTTGTGCAAGCATGTGTGCAGATACCAATGTAGACACCTGTGCAAGCATGTGTGCAGATACCAATGTAGATACCTGTGCAAGCACTTGTACAAGCACAGAATAC |
| 340 | Lt14/DP-Ignalina-2 | Lithuania | DP | 2014 | KM374564 | II | [This study (gallardo@inia.es)](http://asf-referencelab.info/asf/images/files/publicaciones/Gallardo-et-al-2009a.pdf) | ATGCAGCCCACTCACCACGCAGAGATAAGCTTTCAGGATAGAGATACAGCTCTTCCAGACGCATGTTCATCTATATCTGATATTAGCCCCGTTACGTATCCGATCACATTACCTATTATTAAAAACATTTCCGTAACTGCTCATGGTATCAATCTTATCGATAAATTTCCATCAAAGTTCTGCAGCTCTTACATACCCTTCCACTACGGAGGCAATGCGATTAAAACCCCCGATGATCCGGGTGCGATGATGATTACCTTTGCTTTGAAGCCACGGGAGGAATACCAACCCAGTGGTCATATTAACGTATCCAGAGCAAGAGAATTTTATATTAGTTGGGACACGGATTACGTGGGGTCTATCACTACGGCTGATCTTGTGGTATCGGCATCTGCTATTAACTT |  | [This study (gallardo@inia.es)](http://asf-referencelab.info/asf/images/files/publicaciones/Nix_et_al_2006.pdf) | AGTGCGTATACTTGTGCAGATACCAATGTAGACACTTGTGCAAGCATGTGTGCAGATACCAATGTAGACACCTGTGCAAGCATGTGTGCAGATACCAATGTAGATACCTGTGCAAGCACTTGTACAAGCACAGAATAC |
| 341 | Lt14/DP-Ignalina-3 | Lithuania | DP | 2014 | KM374565 | II | [This study (gallardo@inia.es)](http://asf-referencelab.info/asf/images/files/publicaciones/Gallardo-et-al-2009a.pdf) | ATGCAGCCCACTCACCACGCAGAGATAAGCTTTCAGGATAGAGATACAGCTCTTCCAGACGCATGTTCATCTATATCTGATATTAGCCCCGTTACGTATCCGATCACATTACCTATTATTAAAAACATTTCCGTAACTGCTCATGGTATCAATCTTATCGATAAATTTCCATCAAAGTTCTGCAGCTCTTACATACCCTTCCACTACGGAGGCAATGCGATTAAAACCCCCGATGATCCGGGTGCGATGATGATTACCTTTGCTTTGAAGCCACGGGAGGAATACCAACCCAGTGGTCATATTAACGTATCCAGAGCAAGAGAATTTTATATTAGTTGGGACACGGATTACGTGGGGTCTATCACTACGGCTGATCTTGTGGTATCGGCATCTGCTATTAACTT |  | [This study (gallardo@inia.es)](http://asf-referencelab.info/asf/images/files/publicaciones/Nix_et_al_2006.pdf) | AGTGCGTATACTTGTGCAGATACCAATGTAGACACTTGTGCAAGCATGTGTGCAGATACCAATGTAGACACCTGTGCAAGCATGTGTGCAGATACCAATGTAGATACCTGTGCAAGCACTTGTACAAGCACAGAATAC |
| 342 | Lt14/DP-Ignalina-4 | Lithuania | DP | 2014 | KM374566 | II | [This study (gallardo@inia.es)](http://asf-referencelab.info/asf/images/files/publicaciones/Gallardo-et-al-2009a.pdf) | ATGCAGCCCACTCACCACGCAGAGATAAGCTTTCAGGATAGAGATACAGCTCTTCCAGACGCATGTTCATCTATATCTGATATTAGCCCCGTTACGTATCCGATCACATTACCTATTATTAAAAACATTTCCGTAACTGCTCATGGTATCAATCTTATCGATAAATTTCCATCAAAGTTCTGCAGCTCTTACATACCCTTCCACTACGGAGGCAATGCGATTAAAACCCCCGATGATCCGGGTGCGATGATGATTACCTTTGCTTTGAAGCCACGGGAGGAATACCAACCCAGTGGTCATATTAACGTATCCAGAGCAAGAGAATTTTATATTAGTTGGGACACGGATTACGTGGGGTCTATCACTACGGCTGATCTTGTGGTATCGGCATCTGCTATTAACTT |  | [This study (gallardo@inia.es)](http://asf-referencelab.info/asf/images/files/publicaciones/Nix_et_al_2006.pdf) | AGTGCGTATACTTGTGCAGATACCAATGTAGACACTTGTGCAAGCATGTGTGCAGATACCAATGTAGACACCTGTGCAAGCATGTGTGCAGATACCAATGTAGATACCTGTGCAAGCACTTGTACAAGCACAGAATAC |
| 343 | Lt14/DP-Ignalina-5 | Lithuania | DP | 2014 | KM374567 | II | [This study (gallardo@inia.es)](file:///C:\Users\Cgallardo\Dropbox\Carmina%20AGOSTO%202014\SECUENCIAS%20CRL\Desktop\Carmina%20MARZO%202014\ARTICULOS\VPPA\GENOTIPADO\2004%20Bastos%20et%20al.pdf) | ATGCAGCCCACTCACCACGCAGAGATAAGCTTTCAGGATAGAGATACAGCTCTTCCAGACGCATGTTCATCTATATCTGATATTAGCCCCGTTACGTATCCGATCACATTACCTATTATTAAAAACATTTCCGTAACTGCTCATGGTATCAATCTTATCGATAAATTTCCATCAAAGTTCTGCAGCTCTTACATACCCTTCCACTACGGAGGCAATGCGATTAAAACCCCCGATGATCCGGGTGCGATGATGATTACCTTTGCTTTGAAGCCACGGGAGGAATACCAACCCAGTGGTCATATTAACGTATCCAGAGCAAGAGAATTTTATATTAGTTGGGACACGGATTACGTGGGGTCTATCACTACGGCTGATCTTGTGGTATCGGCATCTGCTATTAACTT |  | [This study (gallardo@inia.es)](http://asf-referencelab.info/asf/images/files/publicaciones/Nix_et_al_2006.pdf) | AGTGCGTATACTTGTGCAGATACCAATGTAGACACTTGTGCAAGCATGTGTGCAGATACCAATGTAGACACCTGTGCAAGCATGTGTGCAGATACCAATGTAGATACCTGTGCAAGCACTTGTACAAGCACAGAATAC |
| 344 | Lt14/DP-Ignalina-6 | Lithuania | DP | 2014 | KM374568 | II | [This study (gallardo@inia.es)](file:///C:\Users\Cgallardo\Dropbox\Carmina%20AGOSTO%202014\SECUENCIAS%20CRL\Desktop\Carmina%20MARZO%202014\ARTICULOS\VPPA\GENOTIPADO\2004%20Bastos%20et%20al.pdf) | ATGCAGCCCACTCACCACGCAGAGATAAGCTTTCAGGATAGAGATACAGCTCTTCCAGACGCATGTTCATCTATATCTGATATTAGCCCCGTTACGTATCCGATCACATTACCTATTATTAAAAACATTTCCGTAACTGCTCATGGTATCAATCTTATCGATAAATTTCCATCAAAGTTCTGCAGCTCTTACATACCCTTCCACTACGGAGGCAATGCGATTAAAACCCCCGATGATCCGGGTGCGATGATGATTACCTTTGCTTTGAAGCCACGGGAGGAATACCAACCCAGTGGTCATATTAACGTATCCAGAGCAAGAGAATTTTATATTAGTTGGGACACGGATTACGTGGGGTCTATCACTACGGCTGATCTTGTGGTATCGGCATCTGCTATTAACTT |  | [This study (gallardo@inia.es)](http://asf-referencelab.info/asf/images/files/publicaciones/Nix_et_al_2006.pdf) | AGTGCGTATACTTGTGCAGATACCAATGTAGACACTTGTGCAAGCATGTGTGCAGATACCAATGTAGACACCTGTGCAAGCATGTGTGCAGATACCAATGTAGATACCTGTGCAAGCACTTGTACAAGCACAGAATAC |
| 345 | Lt14/DP-Ignalina-7 | Lithuania | DP | 2014 | KM374569 | II | [This study (gallardo@inia.es)](file:///C:\Users\Cgallardo\Dropbox\Carmina%20AGOSTO%202014\SECUENCIAS%20CRL\Desktop\Carmina%20MARZO%202014\ARTICULOS\VPPA\GENOTIPADO\2004%20Bastos%20et%20al.pdf) | ATGCAGCCCACTCACCACGCAGAGATAAGCTTTCAGGATAGAGATACAGCTCTTCCAGACGCATGTTCATCTATATCTGATATTAGCCCCGTTACGTATCCGATCACATTACCTATTATTAAAAACATTTCCGTAACTGCTCATGGTATCAATCTTATCGATAAATTTCCATCAAAGTTCTGCAGCTCTTACATACCCTTCCACTACGGAGGCAATGCGATTAAAACCCCCGATGATCCGGGTGCGATGATGATTACCTTTGCTTTGAAGCCACGGGAGGAATACCAACCCAGTGGTCATATTAACGTATCCAGAGCAAGAGAATTTTATATTAGTTGGGACACGGATTACGTGGGGTCTATCACTACGGCTGATCTTGTGGTATCGGCATCTGCTATTAACTT |  | [This study (gallardo@inia.es)](http://asf-referencelab.info/asf/images/files/publicaciones/Nix_et_al_2006.pdf) | AGTGCGTATACTTGTGCAGATACCAATGTAGACACTTGTGCAAGCATGTGTGCAGATACCAATGTAGACACCTGTGCAAGCATGTGTGCAGATACCAATGTAGATACCTGTGCAAGCACTTGTACAAGCACAGAATAC |
| 346 | Lt14/DP-Ignalina-8 | Lithuania | DP | 2014 | KM374570 | II | [This study (gallardo@inia.es)](http://asf-referencelab.info/asf/images/files/publicaciones/Gallardo-et-al-2009a.pdf) | ATGCAGCCCACTCACCACGCAGAGATAAGCTTTCAGGATAGAGATACAGCTCTTCCAGACGCATGTTCATCTATATCTGATATTAGCCCCGTTACGTATCCGATCACATTACCTATTATTAAAAACATTTCCGTAACTGCTCATGGTATCAATCTTATCGATAAATTTCCATCAAAGTTCTGCAGCTCTTACATACCCTTCCACTACGGAGGCAATGCGATTAAAACCCCCGATGATCCGGGTGCGATGATGATTACCTTTGCTTTGAAGCCACGGGAGGAATACCAACCCAGTGGTCATATTAACGTATCCAGAGCAAGAGAATTTTATATTAGTTGGGACACGGATTACGTGGGGTCTATCACTACGGCTGATCTTGTGGTATCGGCATCTGCTATTAACTT |  | [This study (gallardo@inia.es)](file:///C:\Users\Cgallardo\Dropbox\Carmina%20AGOSTO%202014\SECUENCIAS%20CRL\Desktop\Carmina%20MARZO%202014\ARTICULOS\VPPA\GENOTIPADO\2007%20Boshoff%20et%20al.pdf) | AGTGCGTATACTTGTGCAGATACCAATGTAGACACTTGTGCAAGCATGTGTGCAGATACCAATGTAGACACCTGTGCAAGCATGTGTGCAGATACCAATGTAGATACCTGTGCAAGCACTTGTACAAGCACAGAATAC |
| 347 | Lt14/DP-Ignalina-9 | Lithuania | DP | 2014 | KM374571 | II | [This study (gallardo@inia.es)](http://asf-referencelab.info/asf/images/files/publicaciones/Gallardo-et-al-2009a.pdf) | ATGCAGCCCACTCACCACGCAGAGATAAGCTTTCAGGATAGAGATACAGCTCTTCCAGACGCATGTTCATCTATATCTGATATTAGCCCCGTTACGTATCCGATCACATTACCTATTATTAAAAACATTTCCGTAACTGCTCATGGTATCAATCTTATCGATAAATTTCCATCAAAGTTCTGCAGCTCTTACATACCCTTCCACTACGGAGGCAATGCGATTAAAACCCCCGATGATCCGGGTGCGATGATGATTACCTTTGCTTTGAAGCCACGGGAGGAATACCAACCCAGTGGTCATATTAACGTATCCAGAGCAAGAGAATTTTATATTAGTTGGGACACGGATTACGTGGGGTCTATCACTACGGCTGATCTTGTGGTATCGGCATCTGCTATTAACTT |  | [This study (gallardo@inia.es)](file:///C:\Users\Cgallardo\Dropbox\Carmina%20AGOSTO%202014\SECUENCIAS%20CRL\Desktop\Carmina%20MARZO%202014\ARTICULOS\VPPA\GENOTIPADO\2007%20Boshoff%20et%20al.pdf) | AGTGCGTATACTTGTGCAGATACCAATGTAGACACTTGTGCAAGCATGTGTGCAGATACCAATGTAGACACCTGTGCAAGCATGTGTGCAGATACCAATGTAGATACCTGTGCAAGCACTTGTACAAGCACAGAATAC |
| 348 | Ug10.Amuru | Uganda | DP | 2010 | KC990902 | IX | [Atuhaire DK et al 2013](file:///C:\Users\Cgallardo\Dropbox\Carmina%20AGOSTO%202014\SECUENCIAS%20CRL\Desktop\Carmina%20MARZO%202014\ARTICULOS\VPPA\GENOTIPADO\2003%20Bastos%20et%20al.pdf) | ATGCAGCCTACCCACCACGCAGAGGTAAGCTTTCAGGATAGAGATACAGCTCTTCCAGATGCATGTTCATCCATATCTGATATTACCCCCATTACTTATCCGATCACATTACCTATTATTAAAAACATTTCCGTTACTGCTCACGGTATCAATCTTATCGATAAATTTCCATCAAAGTTCTGCAGCTCTTACATACCCTTCCACTACGGAGGCAATTCGATTAAAACCCCCGACGATCCGGGCGCGATGATGATTACCTTTGCTTTGAAACCACGGGAGGAATACCAACCCAGCGGTCATATTAACGTATCCAGAGCAAGAGAATTTTATATTAGCTGGGACACAGATTATGTGGGGTCTATCACCACGGCTGATCTTGTGGTATCGGCATCCGCTATTAACTT | KC990868.1 | [Atuhaire DK et al 2013](http://asf-referencelab.info/asf/images/files/publicaciones/Nix_et_al_2006.pdf) | AGTGCGTATACCTGTGCAAGCACTTGTGCAAGCACTTGTGCAAGCACTTGTGCAAGCACTTGTGCAGACACCAATGTAGACACTTGTGCAAGCACTTGTGCAGACACTTGTGCAGACACCAATGTAGACACTTGTGCAAGCACTTGTGCAGATACTTGTGCAGACACTTGTGTAAGCACTTGTGTAAGCACTTGTGCAGATACTTGTGCAGACACCAATGTAGACACTTGTGCAAGCACTTGTGCAGACACCAATGTAGACACTTGTGTAAGCACTTGTGCAGACACCTGTGCAAGCACAGAATACCTTTACACATCATGCAAAAAACATTAAATGTAC |
| 349 | Lt14/DP-Utena | Lithuania | DP | 2014 |  | II | [This study (gallardo@inia.es)](file:///C:\Users\Cgallardo\Dropbox\Carmina%20AGOSTO%202014\SECUENCIAS%20CRL\Desktop\Carmina%20MARZO%202014\ARTICULOS\VPPA\GENOTIPADO\2003%20Bastos%20et%20al.pdf) | ATGCAGCCCACTCACCACGCAGAGATAAGCTTTCAGGATAGAGATACAGCTCTTCCAGACGCATGTTCATCTATATCTGATATTAGCCCCGTTACGTATCCGATCACATTACCTATTATTAAAAACATTTCCGTAACTGCTCATGGTATCAATCTTATCGATAAATTTCCATCAAAGTTCTGCAGCTCTTACATACCCTTCCACTACGGAGGCAATGCGATTAAAACCCCCGATGATCCGGGTGCGATGATGATTACCTTTGCTTTGAAGCCACGGGAGGAATACCAACCCAGTGGTCATATTAACGTATCCAGAGCAAGAGAATTTTATATTAGTTGGGACACGGATTACGTGGGGTCTATCACTACGGCTGATCTTGTGGTATCGGCATCTGCTATTAACTT |  | [This study (gallardo@inia.es)](file:///C:\Users\Cgallardo\Dropbox\Carmina%20AGOSTO%202014\SECUENCIAS%20CRL\Desktop\Carmina%20MARZO%202014\ARTICULOS\VPPA\GENOTIPADO\2004%20Bastos%20et%20al.pdf) | AGTGCGTATACTTGTGCAGATACCAATGTAGACACTTGTGCAAGCATGTGTGCAGATACCAATGTAGACACCTGTGCAAGCATGTGTGCAGATACCAATGTAGATACCTGTGCAAGCACTTGTACAAGCACAGAATAC |
| 350 | Pol14/WB-16421#11 | Poland | Wild pig | 2014 |  | II | [This study (gallardo@inia.es)](http://asf-referencelab.info/asf/images/files/publicaciones/Gallardo-et-al-2009a.pdf) | ATGCAGCCCACTCACCACGCAGAGATAAGCTTTCAGGATAGAGATACAGCTCTTCCAGACGCATGTTCATCTATATCTGATATTAGCCCCGTTACGTATCCGATCACATTACCTATTATTAAAAACATTTCCGTAACTGCTCATGGTATCAATCTTATCGATAAATTTCCATCAAAGTTCTGCAGCTCTTACATACCCTTCCACTACGGAGGCAATGCGATTAAAACCCCCGATGATCCGGGTGCGATGATGATTACCTTTGCTTTGAAGCCACGGGAGGAATACCAACCCAGTGGTCATATTAACGTATCCAGAGCAAGAGAATTTTATATTAGTTGGGACACGGATTACGTGGGGTCTATCACTACGGCTGATCTTGTGGTATCGGCATCTGCTATTAACTT |  | [This study (gallardo@inia.es)](file:///C:\Users\Cgallardo\Dropbox\Carmina%20AGOSTO%202014\SECUENCIAS%20CRL\Desktop\Carmina%20MARZO%202014\ARTICULOS\VPPA\GENOTIPADO\2004%20Bastos%20et%20al.pdf) | AGTGCGTATACTTGTGCAGATACCAATGTAGACACTTGTGCAAGCATGTGTGCAGATACCAATGTAGACACCTGTGCAAGCATGTGTGCAGATACCAATGTAGATACCTGTGCAAGCACTTGTACAAGCACAGAATAC |
| 351 | Pol14/WB-16606#12 | Poland | Wild pig | 2014 |  | II | [This study (gallardo@inia.es)](http://asf-referencelab.info/asf/images/files/publicaciones/Gallardo-et-al-2009a.pdf) | ATGCAGCCCACTCACCACGCAGAGATAAGCTTTCAGGATAGAGATACAGCTCTTCCAGACGCATGTTCATCTATATCTGATATTAGCCCCGTTACGTATCCGATCACATTACCTATTATTAAAAACATTTCCGTAACTGCTCATGGTATCAATCTTATCGATAAATTTCCATCAAAGTTCTGCAGCTCTTACATACCCTTCCACTACGGAGGCAATGCGATTAAAACCCCCGATGATCCGGGTGCGATGATGATTACCTTTGCTTTGAAGCCACGGGAGGAATACCAACCCAGTGGTCATATTAACGTATCCAGAGCAAGAGAATTTTATATTAGTTGGGACACGGATTACGTGGGGTCTATCACTACGGCTGATCTTGTGGTATCGGCATCTGCTATTAACTT |  | [This study (gallardo@inia.es)](file:///C:\Users\Cgallardo\Dropbox\Carmina%20AGOSTO%202014\SECUENCIAS%20CRL\Desktop\Carmina%20MARZO%202014\ARTICULOS\VPPA\GENOTIPADO\2004%20Bastos%20et%20al.pdf) | AGTGCGTATACTTGTGCAGATACCAATGTAGACACTTGTGCAAGCATGTGTGCAGATACCAATGTAGACACCTGTGCAAGCATGTGTGCAGATACCAATGTAGATACCTGTGCAAGCACTTGTACAAGCACAGAATAC |
| 352 | Pol14/WB-16607#12 | Poland | Wild pig | 2014 |  | II | [This study (gallardo@inia.es)](http://asf-referencelab.info/asf/images/files/publicaciones/Gallardo-et-al-2009a.pdf) | ATGCAGCCCACTCACCACGCAGAGATAAGCTTTCAGGATAGAGATACAGCTCTTCCAGACGCATGTTCATCTATATCTGATATTAGCCCCGTTACGTATCCGATCACATTACCTATTATTAAAAACATTTCCGTAACTGCTCATGGTATCAATCTTATCGATAAATTTCCATCAAAGTTCTGCAGCTCTTACATACCCTTCCACTACGGAGGCAATGCGATTAAAACCCCCGATGATCCGGGTGCGATGATGATTACCTTTGCTTTGAAGCCACGGGAGGAATACCAACCCAGTGGTCATATTAACGTATCCAGAGCAAGAGAATTTTATATTAGTTGGGACACGGATTACGTGGGGTCTATCACTACGGCTGATCTTGTGGTATCGGCATCTGCTATTAACTT |  | [This study (gallardo@inia.es)](file:///C:\Users\Cgallardo\Dropbox\Carmina%20AGOSTO%202014\SECUENCIAS%20CRL\Desktop\Carmina%20MARZO%202014\ARTICULOS\VPPA\GENOTIPADO\2004%20Bastos%20et%20al.pdf) | AGTGCGTATACTTGTGCAGATACCAATGTAGACACTTGTGCAAGCATGTGTGCAGATACCAATGTAGACACCTGTGCAAGCATGTGTGCAGATACCAATGTAGATACCTGTGCAAGCACTTGTACAAGCACAGAATAC |
| 353 | Pol14/WB-17513#12 | Poland | Wild pig | 2014 |  | II | [This study (gallardo@inia.es)](http://asf-referencelab.info/asf/images/files/publicaciones/Gallardo-et-al-2009a.pdf) | ATGCAGCCCACTCACCACGCAGAGATAAGCTTTCAGGATAGAGATACAGCTCTTCCAGACGCATGTTCATCTATATCTGATATTAGCCCCGTTACGTATCCGATCACATTACCTATTATTAAAAACATTTCCGTAACTGCTCATGGTATCAATCTTATCGATAAATTTCCATCAAAGTTCTGCAGCTCTTACATACCCTTCCACTACGGAGGCAATGCGATTAAAACCCCCGATGATCCGGGTGCGATGATGATTACCTTTGCTTTGAAGCCACGGGAGGAATACCAACCCAGTGGTCATATTAACGTATCCAGAGCAAGAGAATTTTATATTAGTTGGGACACGGATTACGTGGGGTCTATCACTACGGCTGATCTTGTGGTATCGGCATCTGCTATTAACTT |  | [This study (gallardo@inia.es)](file:///C:\Users\Cgallardo\Dropbox\Carmina%20AGOSTO%202014\SECUENCIAS%20CRL\Desktop\Carmina%20MARZO%202014\ARTICULOS\VPPA\GENOTIPADO\2004%20Bastos%20et%20al.pdf) | AGTGCGTATACTTGTGCAGATACCAATGTAGACACTTGTGCAAGCATGTGTGCAGATACCAATGTAGACACCTGTGCAAGCATGTGTGCAGATACCAATGTAGATACCTGTGCAAGCACTTGTACAAGCACAGAATAC |
| 354 | Lv14/DP/SKA-2 | Latvia | DP | 2014 |  | II | [This study (gallardo@inia.es)](file:///C:\Users\Cgallardo\Dropbox\Carmina%20AGOSTO%202014\SECUENCIAS%20CRL\Desktop\Carmina%20MARZO%202014\ARTICULOS\VPPA\GENOTIPADO\2003%20Bastos%20et%20al.pdf) | ATGCAGCCCACTCACCACGCAGAGATAAGCTTTCAGGATAGAGATACAGCTCTTCCAGACGCATGTTCATCTATATCTGATATTAGCCCCGTTACGTATCCGATCACATTACCTATTATTAAAAACATTTCCGTAACTGCTCATGGTATCAATCTTATCGATAAATTTCCATCAAAGTTCTGCAGCTCTTACATACCCTTCCACTACGGAGGCAATGCGATTAAAACCCCCGATGATCCGGGTGCGATGATGATTACCTTTGCTTTGAAGCCACGGGAGGAATACCAACCCAGTGGTCATATTAACGTATCCAGAGCAAGAGAATTTTATATTAGTTGGGACACGGATTACGTGGGGTCTATCACTACGGCTGATCTTGTGGTATCGGCATCTGCTATTAACTT |  | [This study (gallardo@inia.es)](file:///C:\Users\Cgallardo\Dropbox\Carmina%20AGOSTO%202014\SECUENCIAS%20CRL\Desktop\Carmina%20MARZO%202014\ARTICULOS\VPPA\GENOTIPADO\2004%20Bastos%20et%20al.pdf) | AGTGCGTATACTTGTGCAGATACCAATGTAGACACTTGTGCAAGCATGTGTGCAGATACCAATGTAGACACCTGTGCAAGCATGTGTGCAGATACCAATGTAGATACCTGTGCAAGCACTTGTACAAGCACAGAATAC |
| 355 | Pol14/DP-17192#2 | Poland | DP | 2014 |  | II | [This study (gallardo@inia.es)](http://asf-referencelab.info/asf/images/files/publicaciones/Gallardo-et-al-2009a.pdf) | ATGCAGCCCACTCACCACGCAGAGATAAGCTTTCAGGATAGAGATACAGCTCTTCCAGACGCATGTTCATCTATATCTGATATTAGCCCCGTTACGTATCCGATCACATTACCTATTATTAAAAACATTTCCGTAACTGCTCATGGTATCAATCTTATCGATAAATTTCCATCAAAGTTCTGCAGCTCTTACATACCCTTCCACTACGGAGGCAATGCGATTAAAACCCCCGATGATCCGGGTGCGATGATGATTACCTTTGCTTTGAAGCCACGGGAGGAATACCAACCCAGTGGTCATATTAACGTATCCAGAGCAAGAGAATTTTATATTAGTTGGGACACGGATTACGTGGGGTCTATCACTACGGCTGATCTTGTGGTATCGGCATCTGCTATTAACTT |  | [This study (gallardo@inia.es)](file:///C:\Users\Cgallardo\Dropbox\Carmina%20AGOSTO%202014\SECUENCIAS%20CRL\Desktop\Carmina%20MARZO%202014\ARTICULOS\VPPA\GENOTIPADO\2004%20Bastos%20et%20al.pdf) | AGTGCGTATACTTGTGCAGATACCAATGTAGACACTTGTGCAAGCATGTGTGCAGATACCAATGTAGACACCTGTGCAAGCATGTGTGCAGATACCAATGTAGATACCTGTGCAAGCACTTGTACAAGCACAGAATAC |
| 356 | Lt14/WB-Ignalina-1 | Lithuania | Wild pig | 2014 |  | II | [This study (gallardo@inia.es)](file:///C:\Users\Cgallardo\Dropbox\Carmina%20AGOSTO%202014\SECUENCIAS%20CRL\Desktop\Carmina%20MARZO%202014\ARTICULOS\VPPA\GENOTIPADO\2004%20Bastos%20et%20al.pdf) | ATGCAGCCCACTCACCACGCAGAGATAAGCTTTCAGGATAGAGATACAGCTCTTCCAGACGCATGTTCATCTATATCTGATATTAGCCCCGTTACGTATCCGATCACATTACCTATTATTAAAAACATTTCCGTAACTGCTCATGGTATCAATCTTATCGATAAATTTCCATCAAAGTTCTGCAGCTCTTACATACCCTTCCACTACGGAGGCAATGCGATTAAAACCCCCGATGATCCGGGTGCGATGATGATTACCTTTGCTTTGAAGCCACGGGAGGAATACCAACCCAGTGGTCATATTAACGTATCCAGAGCAAGAGAATTTTATATTAGTTGGGACACGGATTACGTGGGGTCTATCACTACGGCTGATCTTGTGGTATCGGCATCTGCTATTAACTT |  | [This study (gallardo@inia.es)](file:///C:\Users\Cgallardo\Dropbox\Carmina%20AGOSTO%202014\SECUENCIAS%20CRL\Desktop\Carmina%20MARZO%202014\ARTICULOS\VPPA\GENOTIPADO\2004%20Bastos%20et%20al.pdf) | AGTGCGTATACTTGTGCAGATACCAATGTAGACACTTGTGCAAGCATGTGTGCAGATACCAATGTAGACACCTGTGCAAGCATGTGTGCAGATACCAATGTAGATACCTGTGCAAGCACTTGTACAAGCACAGAATAC |
| 357 | Pol14/WB-17397#13 | Poland | Wild pig | 2014 |  | II | [This study (gallardo@inia.es)](file:///C:\Users\Cgallardo\Dropbox\Carmina%20AGOSTO%202014\SECUENCIAS%20CRL\Desktop\Carmina%20MARZO%202014\ARTICULOS\VPPA\GENOTIPADO\2004%20Bastos%20et%20al.pdf) | ATGCAGCCCACTCACCACGCAGAGATAAGCTTTCAGGATAGAGATACAGCTCTTCCAGACGCATGTTCATCTATATCTGATATTAGCCCCGTTACGTATCCGATCACATTACCTATTATTAAAAACATTTCCGTAACTGCTCATGGTATCAATCTTATCGATAAATTTCCATCAAAGTTCTGCAGCTCTTACATACCCTTCCACTACGGAGGCAATGCGATTAAAACCCCCGATGATCCGGGTGCGATGATGATTACCTTTGCTTTGAAGCCACGGGAGGAATACCAACCCAGTGGTCATATTAACGTATCCAGAGCAAGAGAATTTTATATTAGTTGGGACACGGATTACGTGGGGTCTATCACTACGGCTGATCTTGTGGTATCGGCATCTGCTATTAACTT |  | [This study (gallardo@inia.es)](file:///C:\Users\Cgallardo\Dropbox\Carmina%20AGOSTO%202014\SECUENCIAS%20CRL\Desktop\Carmina%20MARZO%202014\ARTICULOS\VPPA\GENOTIPADO\2004%20Bastos%20et%20al.pdf) | AGTGCGTATACTTGTGCAGATACCAATGTAGACACTTGTGCAAGCATGTGTGCAGATACCAATGTAGACACCTGTGCAAGCATGTGTGCAGATACCAATGTAGATACCTGTGCAAGCACTTGTACAAGCACAGAATAC |
| 358 | Lt14/DP-Ignalina-13 | Lithuania | DP | 2014 |  | II | [This study (gallardo@inia.es)](file:///C:\Users\Cgallardo\Dropbox\Carmina%20AGOSTO%202014\SECUENCIAS%20CRL\Desktop\Carmina%20MARZO%202014\ARTICULOS\VPPA\GENOTIPADO\2004%20Bastos%20et%20al.pdf) | ATGCAGCCCACTCACCACGCAGAGATAAGCTTTCAGGATAGAGATACAGCTCTTCCAGACGCATGTTCATCTATATCTGATATTAGCCCCGTTACGTATCCGATCACATTACCTATTATTAAAAACATTTCCGTAACTGCTCATGGTATCAATCTTATCGATAAATTTCCATCAAAGTTCTGCAGCTCTTACATACCCTTCCACTACGGAGGCAATGCGATTAAAACCCCCGATGATCCGGGTGCGATGATGATTACCTTTGCTTTGAAGCCACGGGAGGAATACCAACCCAGTGGTCATATTAACGTATCCAGAGCAAGAGAATTTTATATTAGTTGGGACACGGATTACGTGGGGTCTATCACTACGGCTGATCTTGTGGTATCGGCATCTGCTATTAACTT |  | [This study (gallardo@inia.es)](file:///C:\Users\Cgallardo\Dropbox\Carmina%20AGOSTO%202014\SECUENCIAS%20CRL\Desktop\Carmina%20MARZO%202014\ARTICULOS\VPPA\GENOTIPADO\2004%20Bastos%20et%20al.pdf) | AGTGCGTATACTTGTGCAGATACCAATGTAGACACTTGTGCAAGCATGTGTGCAGATACCAATGTAGACACCTGTGCAAGCATGTGTGCAGATACCAATGTAGATACCTGTGCAAGCACTTGTACAAGCACAGAATAC |
| 359 | Lt14/DP-Ignalina-14 | Lithuania | DP | 2014 |  | II | [This study (gallardo@inia.es)](http://asf-referencelab.info/asf/images/files/publicaciones/Gallardo-et-al-2009a.pdf) | ATGCAGCCCACTCACCACGCAGAGATAAGCTTTCAGGATAGAGATACAGCTCTTCCAGACGCATGTTCATCTATATCTGATATTAGCCCCGTTACGTATCCGATCACATTACCTATTATTAAAAACATTTCCGTAACTGCTCATGGTATCAATCTTATCGATAAATTTCCATCAAAGTTCTGCAGCTCTTACATACCCTTCCACTACGGAGGCAATGCGATTAAAACCCCCGATGATCCGGGTGCGATGATGATTACCTTTGCTTTGAAGCCACGGGAGGAATACCAACCCAGTGGTCATATTAACGTATCCAGAGCAAGAGAATTTTATATTAGTTGGGACACGGATTACGTGGGGTCTATCACTACGGCTGATCTTGTGGTATCGGCATCTGCTATTAACTT |  | [This study (gallardo@inia.es)](http://asf-referencelab.info/asf/images/files/publicaciones/Nix_et_al_2006.pdf) | AGTGCGTATACTTGTGCAGATACCAATGTAGACACTTGTGCAAGCATGTGTGCAGATACCAATGTAGACACCTGTGCAAGCATGTGTGCAGATACCAATGTAGATACCTGTGCAAGCACTTGTACAAGCACAGAATAC |
| 360 | Lt14/DP-Ignalina-15 | Lithuania | DP | 2014 |  | II | [This study (gallardo@inia.es)](http://asf-referencelab.info/asf/images/files/publicaciones/Gallardo-et-al-2009a.pdf) | ATGCAGCCCACTCACCACGCAGAGATAAGCTTTCAGGATAGAGATACAGCTCTTCCAGACGCATGTTCATCTATATCTGATATTAGCCCCGTTACGTATCCGATCACATTACCTATTATTAAAAACATTTCCGTAACTGCTCATGGTATCAATCTTATCGATAAATTTCCATCAAAGTTCTGCAGCTCTTACATACCCTTCCACTACGGAGGCAATGCGATTAAAACCCCCGATGATCCGGGTGCGATGATGATTACCTTTGCTTTGAAGCCACGGGAGGAATACCAACCCAGTGGTCATATTAACGTATCCAGAGCAAGAGAATTTTATATTAGTTGGGACACGGATTACGTGGGGTCTATCACTACGGCTGATCTTGTGGTATCGGCATCTGCTATTAACTT |  | [This study (gallardo@inia.es)](http://asf-referencelab.info/asf/images/files/publicaciones/Nix_et_al_2006.pdf) | AGTGCGTATACTTGTGCAGATACCAATGTAGACACTTGTGCAAGCATGTGTGCAGATACCAATGTAGACACCTGTGCAAGCATGTGTGCAGATACCAATGTAGATACCTGTGCAAGCACTTGTACAAGCACAGAATAC |
| 361 | Lv14/WB/LAU-1 | Latvia | Wild pig | 2014 |  | II | [This study (gallardo@inia.es)](http://asf-referencelab.info/asf/images/files/publicaciones/Gallardo-et-al-2009a.pdf) | ATGCAGCCCACTCACCACGCAGAGATAAGCTTTCAGGATAGAGATACAGCTCTTCCAGACGCATGTTCATCTATATCTGATATTAGCCCCGTTACGTATCCGATCACATTACCTATTATTAAAAACATTTCCGTAACTGCTCATGGTATCAATCTTATCGATAAATTTCCATCAAAGTTCTGCAGCTCTTACATACCCTTCCACTACGGAGGCAATGCGATTAAAACCCCCGATGATCCGGGTGCGATGATGATTACCTTTGCTTTGAAGCCACGGGAGGAATACCAACCCAGTGGTCATATTAACGTATCCAGAGCAAGAGAATTTTATATTAGTTGGGACACGGATTACGTGGGGTCTATCACTACGGCTGATCTTGTGGTATCGGCATCTGCTATTAACTT |  | [This study (gallardo@inia.es)](http://asf-referencelab.info/asf/images/files/publicaciones/Nix_et_al_2006.pdf) | AGTGCGTATACTTGTGCAGATACCAATGTAGACACTTGTGCAAGCATGTGTGCAGATACCAATGTAGACACCTGTGCAAGCATGTGTGCAGATACCAATGTAGATACCTGTGCAAGCACTTGTACAAGCACAGAATAC |
| 362 | Lt14/DP-Rokiškis-1 | Lithuania | DP | 2014 |  | II | [This study (gallardo@inia.es)](http://asf-referencelab.info/asf/images/files/publicaciones/Gallardo-et-al-2009a.pdf) | ATGCAGCCCACTCACCACGCAGAGATAAGCTTTCAGGATAGAGATACAGCTCTTCCAGACGCATGTTCATCTATATCTGATATTAGCCCCGTTACGTATCCGATCACATTACCTATTATTAAAAACATTTCCGTAACTGCTCATGGTATCAATCTTATCGATAAATTTCCATCAAAGTTCTGCAGCTCTTACATACCCTTCCACTACGGAGGCAATGCGATTAAAACCCCCGATGATCCGGGTGCGATGATGATTACCTTTGCTTTGAAGCCACGGGAGGAATACCAACCCAGTGGTCATATTAACGTATCCAGAGCAAGAGAATTTTATATTAGTTGGGACACGGATTACGTGGGGTCTATCACTACGGCTGATCTTGTGGTATCGGCATCTGCTATTAACTT |  | [This study (gallardo@inia.es)](http://asf-referencelab.info/asf/images/files/publicaciones/Nix_et_al_2006.pdf) | AGTGCGTATACTTGTGCAGATACCAATGTAGACACTTGTGCAAGCATGTGTGCAGATACCAATGTAGACACCTGTGCAAGCATGTGTGCAGATACCAATGTAGATACCTGTGCAAGCACTTGTACAAGCACAGAATAC |
| 363 | Pol14/WB-18861#14 | Poland | Wild pig | 2014 |  | II | [This study (gallardo@inia.es)](http://asf-referencelab.info/asf/images/files/publicaciones/Gallardo-et-al-2009a.pdf) | ATGCAGCCCACTCACCACGCAGAGATAAGCTTTCAGGATAGAGATACAGCTCTTCCAGACGCATGTTCATCTATATCTGATATTAGCCCCGTTACGTATCCGATCACATTACCTATTATTAAAAACATTTCCGTAACTGCTCATGGTATCAATCTTATCGATAAATTTCCATCAAAGTTCTGCAGCTCTTACATACCCTTCCACTACGGAGGCAATGCGATTAAAACCCCCGATGATCCGGGTGCGATGATGATTACCTTTGCTTTGAAGCCACGGGAGGAATACCAACCCAGTGGTCATATTAACGTATCCAGAGCAAGAGAATTTTATATTAGTTGGGACACGGATTACGTGGGGTCTATCACTACGGCTGATCTTGTGGTATCGGCATCTGCTATTAACTT |  | [This study (gallardo@inia.es)](http://asf-referencelab.info/asf/images/files/publicaciones/Nix_et_al_2006.pdf) | AGTGCGTATACTTGTGCAGATACCAATGTAGACACTTGTGCAAGCATGTGTGCAGATACCAATGTAGACACCTGTGCAAGCATGTGTGCAGATACCAATGTAGATACCTGTGCAAGCACTTGTACAAGCACAGAATAC |
| 364 | Lt14/DP-Ignalina-16 | Lithuania | DP | 2014 |  | II | [This study (gallardo@inia.es)](http://link.springer.com/article/10.1007%2Fs00705-005-0602-1) | ATGCAGCCCACTCACCACGCAGAGATAAGCTTTCAGGATAGAGATACAGCTCTTCCAGACGCATGTTCATCTATATCTGATATTAGCCCCGTTACGTATCCGATCACATTACCTATTATTAAAAACATTTCCGTAACTGCTCATGGTATCAATCTTATCGATAAATTTCCATCAAAGTTCTGCAGCTCTTACATACCCTTCCACTACGGAGGCAATGCGATTAAAACCCCCGATGATCCGGGTGCGATGATGATTACCTTTGCTTTGAAGCCACGGGAGGAATACCAACCCAGTGGTCATATTAACGTATCCAGAGCAAGAGAATTTTATATTAGTTGGGACACGGATTACGTGGGGTCTATCACTACGGCTGATCTTGTGGTATCGGCATCTGCTATTAACTT |  | [This study (gallardo@inia.es)](http://asf-referencelab.info/asf/images/files/publicaciones/Nix_et_al_2006.pdf) | AGTGCGTATACTTGTGCAGATACCAATGTAGACACTTGTGCAAGCATGTGTGCAGATACCAATGTAGACACCTGTGCAAGCATGTGTGCAGATACCAATGTAGATACCTGTGCAAGCACTTGTACAAGCACAGAATAC |
| 365 | Lt14/DP-Ignalina-17 | Lithuania | DP | 2014 |  | II | [This study (gallardo@inia.es)](http://link.springer.com/article/10.1007%2Fs00705-005-0602-1) | ATGCAGCCCACTCACCACGCAGAGATAAGCTTTCAGGATAGAGATACAGCTCTTCCAGACGCATGTTCATCTATATCTGATATTAGCCCCGTTACGTATCCGATCACATTACCTATTATTAAAAACATTTCCGTAACTGCTCATGGTATCAATCTTATCGATAAATTTCCATCAAAGTTCTGCAGCTCTTACATACCCTTCCACTACGGAGGCAATGCGATTAAAACCCCCGATGATCCGGGTGCGATGATGATTACCTTTGCTTTGAAGCCACGGGAGGAATACCAACCCAGTGGTCATATTAACGTATCCAGAGCAAGAGAATTTTATATTAGTTGGGACACGGATTACGTGGGGTCTATCACTACGGCTGATCTTGTGGTATCGGCATCTGCTATTAACTT |  | [This study (gallardo@inia.es)](http://asf-referencelab.info/asf/images/files/publicaciones/Nix_et_al_2006.pdf) | AGTGCGTATACTTGTGCAGATACCAATGTAGACACTTGTGCAAGCATGTGTGCAGATACCAATGTAGACACCTGTGCAAGCATGTGTGCAGATACCAATGTAGATACCTGTGCAAGCACTTGTACAAGCACAGAATAC |
| 366 | Lt14/DP-Ignalina-18 | Lithuania | DP | 2014 |  | II | [This study (gallardo@inia.es)](http://asf-referencelab.info/asf/images/files/publicaciones/Gallardo-et-al-2009a.pdf) | ATGCAGCCCACTCACCACGCAGAGATAAGCTTTCAGGATAGAGATACAGCTCTTCCAGACGCATGTTCATCTATATCTGATATTAGCCCCGTTACGTATCCGATCACATTACCTATTATTAAAAACATTTCCGTAACTGCTCATGGTATCAATCTTATCGATAAATTTCCATCAAAGTTCTGCAGCTCTTACATACCCTTCCACTACGGAGGCAATGCGATTAAAACCCCCGATGATCCGGGTGCGATGATGATTACCTTTGCTTTGAAGCCACGGGAGGAATACCAACCCAGTGGTCATATTAACGTATCCAGAGCAAGAGAATTTTATATTAGTTGGGACACGGATTACGTGGGGTCTATCACTACGGCTGATCTTGTGGTATCGGCATCTGCTATTAACTT |  | [This study (gallardo@inia.es)](http://asf-referencelab.info/asf/images/files/publicaciones/Nix_et_al_2006.pdf) | AGTGCGTATACTTGTGCAGATACCAATGTAGACACTTGTGCAAGCATGTGTGCAGATACCAATGTAGACACCTGTGCAAGCATGTGTGCAGATACCAATGTAGATACCTGTGCAAGCACTTGTACAAGCACAGAATAC |
| 367 | Lt14/DP-Ignalina-19 | Lithuania | DP | 2014 |  | II | [This study (gallardo@inia.es)](file:///C:\Users\Cgallardo\Dropbox\Carmina%20AGOSTO%202014\SECUENCIAS%20CRL\Desktop\Carmina%20MARZO%202014\ARTICULOS\VPPA\GENOTIPADO\2003%20Bastos%20et%20al.pdf) | ATGCAGCCCACTCACCACGCAGAGATAAGCTTTCAGGATAGAGATACAGCTCTTCCAGACGCATGTTCATCTATATCTGATATTAGCCCCGTTACGTATCCGATCACATTACCTATTATTAAAAACATTTCCGTAACTGCTCATGGTATCAATCTTATCGATAAATTTCCATCAAAGTTCTGCAGCTCTTACATACCCTTCCACTACGGAGGCAATGCGATTAAAACCCCCGATGATCCGGGTGCGATGATGATTACCTTTGCTTTGAAGCCACGGGAGGAATACCAACCCAGTGGTCATATTAACGTATCCAGAGCAAGAGAATTTTATATTAGTTGGGACACGGATTACGTGGGGTCTATCACTACGGCTGATCTTGTGGTATCGGCATCTGCTATTAACTT |  | [This study (gallardo@inia.es)](http://asf-referencelab.info/asf/images/files/publicaciones/Nix_et_al_2006.pdf) | AGTGCGTATACTTGTGCAGATACCAATGTAGACACTTGTGCAAGCATGTGTGCAGATACCAATGTAGACACCTGTGCAAGCATGTGTGCAGATACCAATGTAGATACCTGTGCAAGCACTTGTACAAGCACAGAATAC |
| 368 | Est14/WB-Valga-1 | Estonia | Wild pig | 2014 |  | II | [This study (gallardo@inia.es)](file:///C:\Users\Cgallardo\Dropbox\Carmina%20AGOSTO%202014\SECUENCIAS%20CRL\Desktop\Carmina%20MARZO%202014\ARTICULOS\VPPA\GENOTIPADO\2003%20Bastos%20et%20al.pdf) | ATGCAGCCCACTCACCACGCAGAGATAAGCTTTCAGGATAGAGATACAGCTCTTCCAGACGCATGTTCATCTATATCTGATATTAGCCCCGTTACGTATCCGATCACATTACCTATTATTAAAAACATTTCCGTAACTGCTCATGGTATCAATCTTATCGATAAATTTCCATCAAAGTTCTGCAGCTCTTACATACCCTTCCACTACGGAGGCAATGCGATTAAAACCCCCGATGATCCGGGTGCGATGATGATTACCTTTGCTTTGAAGCCACGGGAGGAATACCAACCCAGTGGTCATATTAACGTATCCAGAGCAAGAGAATTTTATATTAGTTGGGACACGGATTACGTGGGGTCTATCACTACGGCTGATCTTGTGGTATCGGCATCTGCTATTAACTT |  | [This study (gallardo@inia.es)](http://asf-referencelab.info/asf/images/files/publicaciones/Nix_et_al_2006.pdf) | AGTGCGTATACTTGTGCAGATACCAATGTAGACACTTGTGCAAGCATGTGTGCAGATACCAATGTAGACACCTGTGCAAGCATGTGTGCAGATACCAATGTAGATACCTGTGCAAGCACTTGTACAAGCACAGAATAC |
| 369 | Lv14/WB/VALKA 2 | Latvia | Wild pig | 2014 |  | II | [This study (gallardo@inia.es)](http://asf-referencelab.info/asf/images/files/publicaciones/Gallardo-et-al-2009a.pdf) | ATGCAGCCCACTCACCACGCAGAGATAAGCTTTCAGGATAGAGATACAGCTCTTCCAGACGCATGTTCATCTATATCTGATATTAGCCCCGTTACGTATCCGATCACATTACCTATTATTAAAAACATTTCCGTAACTGCTCATGGTATCAATCTTATCGATAAATTTCCATCAAAGTTCTGCAGCTCTTACATACCCTTCCACTACGGAGGCAATGCGATTAAAACCCCCGATGATCCGGGTGCGATGATGATTACCTTTGCTTTGAAGCCACGGGAGGAATACCAACCCAGTGGTCATATTAACGTATCCAGAGCAAGAGAATTTTATATTAGTTGGGACACGGATTACGTGGGGTCTATCACTACGGCTGATCTTGTGGTATCGGCATCTGCTATTAACTT |  | [This study (gallardo@inia.es)](http://asf-referencelab.info/asf/images/files/publicaciones/Nix_et_al_2006.pdf) | AGTGCGTATACTTGTGCAGATACCAATGTAGACACTTGTGCAAGCATGTGTGCAGATACCAATGTAGACACCTGTGCAAGCATGTGTGCAGATACCAATGTAGATACCTGTGCAAGCACTTGTACAAGCACAGAATAC |
| 370 | Lt14/WB-Ignalina-2 | Lithuania | Wild pig | 2014 |  | II | [This study (gallardo@inia.es)](http://asf-referencelab.info/asf/images/files/publicaciones/Gallardo-et-al-2009a.pdf) | ATGCAGCCCACTCACCACGCAGAGATAAGCTTTCAGGATAGAGATACAGCTCTTCCAGACGCATGTTCATCTATATCTGATATTAGCCCCGTTACGTATCCGATCACATTACCTATTATTAAAAACATTTCCGTAACTGCTCATGGTATCAATCTTATCGATAAATTTCCATCAAAGTTCTGCAGCTCTTACATACCCTTCCACTACGGAGGCAATGCGATTAAAACCCCCGATGATCCGGGTGCGATGATGATTACCTTTGCTTTGAAGCCACGGGAGGAATACCAACCCAGTGGTCATATTAACGTATCCAGAGCAAGAGAATTTTATATTAGTTGGGACACGGATTACGTGGGGTCTATCACTACGGCTGATCTTGTGGTATCGGCATCTGCTATTAACTT |  | [This study (gallardo@inia.es)](http://asf-referencelab.info/asf/images/files/publicaciones/Nix_et_al_2006.pdf) | AGTGCGTATACTTGTGCAGATACCAATGTAGACACTTGTGCAAGCATGTGTGCAGATACCAATGTAGACACCTGTGCAAGCATGTGTGCAGATACCAATGTAGATACCTGTGCAAGCACTTGTACAAGCACAGAATAC |
| 371 | Est14/WB-VIL-1 | Estonia | Wild pig | 2014 |  | II | [This study (gallardo@inia.es)](http://asf-referencelab.info/asf/images/files/publicaciones/Gallardo-et-al-2009a.pdf) | ATGCAGCCCACTCACCACGCAGAGATAAGCTTTCAGGATAGAGATACAGCTCTTCCAGACGCATGTTCATCTATATCTGATATTAGCCCCGTTACGTATCCGATCACATTACCTATTATTAAAAACATTTCCGTAACTGCTCATGGTATCAATCTTATCGATAAATTTCCATCAAAGTTCTGCAGCTCTTACATACCCTTCCACTACGGAGGCAATGCGATTAAAACCCCCGATGATCCGGGTGCGATGATGATTACCTTTGCTTTGAAGCCACGGGAGGAATACCAACCCAGTGGTCATATTAACGTATCCAGAGCAAGAGAATTTTATATTAGTTGGGACACGGATTACGTGGGGTCTATCACTACGGCTGATCTTGTGGTATCGGCATCTGCTATTAACTT |  | [This study (gallardo@inia.es)](http://asf-referencelab.info/asf/images/files/publicaciones/Nix_et_al_2006.pdf) | AGTGCGTATACTTGTGCAGATACCAATGTAGACACTTGTGCAAGCATGTGTGCAGATACCAATGTAGACACCTGTGCAAGCATGTGTGCAGATACCAATGTAGATACCTGTGCAAGCACTTGTACAAGCACAGAATAC |
| 372 | Est14/WB-Valga-2 | Estonia | Wild pig | 2014 |  | II | [This study (gallardo@inia.es)](http://link.springer.com/article/10.1007%2Fs00705-005-0602-1) | ATGCAGCCCACTCACCACGCAGAGATAAGCTTTCAGGATAGAGATACAGCTCTTCCAGACGCATGTTCATCTATATCTGATATTAGCCCCGTTACGTATCCGATCACATTACCTATTATTAAAAACATTTCCGTAACTGCTCATGGTATCAATCTTATCGATAAATTTCCATCAAAGTTCTGCAGCTCTTACATACCCTTCCACTACGGAGGCAATGCGATTAAAACCCCCGATGATCCGGGTGCGATGATGATTACCTTTGCTTTGAAGCCACGGGAGGAATACCAACCCAGTGGTCATATTAACGTATCCAGAGCAAGAGAATTTTATATTAGTTGGGACACGGATTACGTGGGGTCTATCACTACGGCTGATCTTGTGGTATCGGCATCTGCTATTAACTT |  | [This study (gallardo@inia.es)](http://asf-referencelab.info/asf/images/files/publicaciones/Nix_et_al_2006.pdf) | AGTGCGTATACTTGTGCAGATACCAATGTAGACACTTGTGCAAGCATGTGTGCAGATACCAATGTAGACACCTGTGCAAGCATGTGTGCAGATACCAATGTAGATACCTGTGCAAGCACTTGTACAAGCACAGAATAC |
| 373 | Est14/WB-VIL-2 | Estonia | Wild pig | 2014 |  | II | [This study (gallardo@inia.es)](http://link.springer.com/article/10.1007%2Fs00705-005-0602-1) | ATGCAGCCCACTCACCACGCAGAGATAAGCTTTCAGGATAGAGATACAGCTCTTCCAGACGCATGTTCATCTATATCTGATATTAGCCCCGTTACGTATCCGATCACATTACCTATTATTAAAAACATTTCCGTAACTGCTCATGGTATCAATCTTATCGATAAATTTCCATCAAAGTTCTGCAGCTCTTACATACCCTTCCACTACGGAGGCAATGCGATTAAAACCCCCGATGATCCGGGTGCGATGATGATTACCTTTGCTTTGAAGCCACGGGAGGAATACCAACCCAGTGGTCATATTAACGTATCCAGAGCAAGAGAATTTTATATTAGTTGGGACACGGATTACGTGGGGTCTATCACTACGGCTGATCTTGTGGTATCGGCATCTGCTATTAACTT |  | [This study (gallardo@inia.es)](http://asf-referencelab.info/asf/images/files/publicaciones/Nix_et_al_2006.pdf) | AGTGCGTATACTTGTGCAGATACCAATGTAGACACTTGTGCAAGCATGTGTGCAGATACCAATGTAGACACCTGTGCAAGCATGTGTGCAGATACCAATGTAGATACCTGTGCAAGCACTTGTACAAGCACAGAATAC |
| 374 | Est14/WB-VIL-3 | Estonia | Wild pig | 2014 |  | II | [This study (gallardo@inia.es)](http://link.springer.com/article/10.1007%2Fs00705-005-0602-1) | ATGCAGCCCACTCACCACGCAGAGATAAGCTTTCAGGATAGAGATACAGCTCTTCCAGACGCATGTTCATCTATATCTGATATTAGCCCCGTTACGTATCCGATCACATTACCTATTATTAAAAACATTTCCGTAACTGCTCATGGTATCAATCTTATCGATAAATTTCCATCAAAGTTCTGCAGCTCTTACATACCCTTCCACTACGGAGGCAATGCGATTAAAACCCCCGATGATCCGGGTGCGATGATGATTACCTTTGCTTTGAAGCCACGGGAGGAATACCAACCCAGTGGTCATATTAACGTATCCAGAGCAAGAGAATTTTATATTAGTTGGGACACGGATTACGTGGGGTCTATCACTACGGCTGATCTTGTGGTATCGGCATCTGCTATTAACTT |  | [This study (gallardo@inia.es)](http://asf-referencelab.info/asf/images/files/publicaciones/Nix_et_al_2006.pdf) | AGTGCGTATACTTGTGCAGATACCAATGTAGACACTTGTGCAAGCATGTGTGCAGATACCAATGTAGACACCTGTGCAAGCATGTGTGCAGATACCAATGTAGATACCTGTGCAAGCACTTGTACAAGCACAGAATAC |
| 375 | Est14/WB-VIL-4 | Estonia | Wild pig | 2014 |  | II | [This study (gallardo@inia.es)](http://link.springer.com/article/10.1007%2Fs00705-005-0602-1) | ATGCAGCCCACTCACCACGCAGAGATAAGCTTTCAGGATAGAGATACAGCTCTTCCAGACGCATGTTCATCTATATCTGATATTAGCCCCGTTACGTATCCGATCACATTACCTATTATTAAAAACATTTCCGTAACTGCTCATGGTATCAATCTTATCGATAAATTTCCATCAAAGTTCTGCAGCTCTTACATACCCTTCCACTACGGAGGCAATGCGATTAAAACCCCCGATGATCCGGGTGCGATGATGATTACCTTTGCTTTGAAGCCACGGGAGGAATACCAACCCAGTGGTCATATTAACGTATCCAGAGCAAGAGAATTTTATATTAGTTGGGACACGGATTACGTGGGGTCTATCACTACGGCTGATCTTGTGGTATCGGCATCTGCTATTAACTT |  | [This study (gallardo@inia.es)](http://asf-referencelab.info/asf/images/files/publicaciones/Nix_et_al_2006.pdf) | AGTGCGTATACTTGTGCAGATACCAATGTAGACACTTGTGCAAGCATGTGTGCAGATACCAATGTAGACACCTGTGCAAGCATGTGTGCAGATACCAATGTAGATACCTGTGCAAGCACTTGTACAAGCACAGAATAC |
| 376 | Pol14/WB-21516#15 | Poland | Wild pig | 2014 |  | II | [This study (gallardo@inia.es)](http://asf-referencelab.info/asf/images/files/publicaciones/Gallardo-et-al-2009a.pdf) | ATGCAGCCCACTCACCACGCAGAGATAAGCTTTCAGGATAGAGATACAGCTCTTCCAGACGCATGTTCATCTATATCTGATATTAGCCCCGTTACGTATCCGATCACATTACCTATTATTAAAAACATTTCCGTAACTGCTCATGGTATCAATCTTATCGATAAATTTCCATCAAAGTTCTGCAGCTCTTACATACCCTTCCACTACGGAGGCAATGCGATTAAAACCCCCGATGATCCGGGTGCGATGATGATTACCTTTGCTTTGAAGCCACGGGAGGAATACCAACCCAGTGGTCATATTAACGTATCCAGAGCAAGAGAATTTTATATTAGTTGGGACACGGATTACGTGGGGTCTATCACTACGGCTGATCTTGTGGTATCGGCATCTGCTATTAACTT |  | [This study (gallardo@inia.es)](file:///C:\Users\Cgallardo\Dropbox\Carmina%20AGOSTO%202014\SECUENCIAS%20CRL\Desktop\Carmina%20MARZO%202014\ARTICULOS\VPPA\GENOTIPADO\2007%20Boshoff%20et%20al.pdf) | AGTGCGTATACTTGTGCAGATACCAATGTAGACACTTGTGCAAGCATGTGTGCAGATACCAATGTAGACACCTGTGCAAGCATGTGTGCAGATACCAATGTAGATACCTGTGCAAGCACTTGTACAAGCACAGAATAC |
| 377 | Est14/WB-IDA-1 | Estonia | Wild pig | 2014 |  | II | [This study (gallardo@inia.es)](file:///C:\Users\Cgallardo\Dropbox\Carmina%20AGOSTO%202014\SECUENCIAS%20CRL\Desktop\Carmina%20MARZO%202014\ARTICULOS\VPPA\GENOTIPADO\2007%20Boshoff%20et%20al.pdf) | ATGCAGCCCACTCACCACGCAGAGATAAGCTTTCAGGATAGAGATACAGCTCTTCCAGACGCATGTTCATCTATATCTGATATTAGCCCCGTTACGTATCCGATCACATTACCTATTATTAAAAACATTTCCGTAACTGCTCATGGTATCAATCTTATCGATAAATTTCCATCAAAGTTCTGCAGCTCTTACATACCCTTCCACTACGGAGGCAATGCGATTAAAACCCCCGATGATCCGGGTGCGATGATGATTACCTTTGCTTTGAAGCCACGGGAGGAATACCAACCCAGTGGTCATATTAACGTATCCAGAGCAAGAGAATTTTATATTAGTTGGGACACGGATTACGTGGGGTCTATCACTACGGCTGATCTTGTGGTATCGGCATCTGCTATTAACTT |  | [This study (gallardo@inia.es)](file:///C:\Users\Cgallardo\Dropbox\Carmina%20AGOSTO%202014\SECUENCIAS%20CRL\Desktop\Carmina%20MARZO%202014\ARTICULOS\VPPA\GENOTIPADO\2007%20Boshoff%20et%20al.pdf) | AGTGCGTATACTTGTGCAGATACCAATGTAGACACTTGTGCAAGCATGTGTGCAGATACCAATGTAGACACCTGTGCAAGCATGTGTGCAGATACCAATGTAGATACCTGTGCAAGCACTTGTACAAGCACAGAATAC |
| 378 | Pol14/WB-23704#16 | Poland | Wild pig | 2014 |  | II | [This study (gallardo@inia.es)](http://asf-referencelab.info/asf/images/files/publicaciones/Gallardo-et-al-2009a.pdf) | ATGCAGCCCACTCACCACGCAGAGATAAGCTTTCAGGATAGAGATACAGCTCTTCCAGACGCATGTTCATCTATATCTGATATTAGCCCCGTTACGTATCCGATCACATTACCTATTATTAAAAACATTTCCGTAACTGCTCATGGTATCAATCTTATCGATAAATTTCCATCAAAGTTCTGCAGCTCTTACATACCCTTCCACTACGGAGGCAATGCGATTAAAACCCCCGATGATCCGGGTGCGATGATGATTACCTTTGCTTTGAAGCCACGGGAGGAATACCAACCCAGTGGTCATATTAACGTATCCAGAGCAAGAGAATTTTATATTAGTTGGGACACGGATTACGTGGGGTCTATCACTACGGCTGATCTTGTGGTATCGGCATCTGCTATTAACTT |  | [This study (gallardo@inia.es)](file:///C:\Users\Cgallardo\Dropbox\Carmina%20AGOSTO%202014\SECUENCIAS%20CRL\Desktop\Carmina%20MARZO%202014\ARTICULOS\VPPA\GENOTIPADO\2007%20Boshoff%20et%20al.pdf) | AGTGCGTATACTTGTGCAGATACCAATGTAGACACTTGTGCAAGCATGTGTGCAGATACCAATGTAGACACCTGTGCAAGCATGTGTGCAGATACCAATGTAGATACCTGTGCAAGCACTTGTACAAGCACAGAATAC |
| 379 | Lt14/WB-Vilnius-1 | Lithuania | Wild pig | 2014 |  | II | [This study (gallardo@inia.es)](file:///C:\Users\Cgallardo\Dropbox\Carmina%20AGOSTO%202014\SECUENCIAS%20CRL\Desktop\Carmina%20MARZO%202014\ARTICULOS\VPPA\GENOTIPADO\2007%20Boshoff%20et%20al.pdf) | ATGCAGCCCACTCACCACGCAGAGATAAGCTTTCAGGATAGAGATACAGCTCTTCCAGACGCATGTTCATCTATATCTGATATTAGCCCCGTTACGTATCCGATCACATTACCTATTATTAAAAACATTTCCGTAACTGCTCATGGTATCAATCTTATCGATAAATTTCCATCAAAGTTCTGCAGCTCTTACATACCCTTCCACTACGGAGGCAATGCGATTAAAACCCCCGATGATCCGGGTGCGATGATGATTACCTTTGCTTTGAAGCCACGGGAGGAATACCAACCCAGTGGTCATATTAACGTATCCAGAGCAAGAGAATTTTATATTAGTTGGGACACGGATTACGTGGGGTCTATCACTACGGCTGATCTTGTGGTATCGGCATCTGCTATTAACTT |  | [This study (gallardo@inia.es)](file:///C:\Users\Cgallardo\Dropbox\Carmina%20AGOSTO%202014\SECUENCIAS%20CRL\Desktop\Carmina%20MARZO%202014\ARTICULOS\VPPA\GENOTIPADO\2007%20Boshoff%20et%20al.pdf) | AGTGCGTATACTTGTGCAGATACCAATGTAGACACTTGTGCAAGCATGTGTGCAGATACCAATGTAGACACCTGTGCAAGCATGTGTGCAGATACCAATGTAGATACCTGTGCAAGCACTTGTACAAGCACAGAATAC |
| 380 | Lt14/WB-Vilnius-2 | Lithuania | Wild pig | 2014 |  | II | [This study (gallardo@inia.es)](file:///C:\Users\Cgallardo\Dropbox\Carmina%20AGOSTO%202014\SECUENCIAS%20CRL\Desktop\Carmina%20MARZO%202014\ARTICULOS\VPPA\GENOTIPADO\2007%20Boshoff%20et%20al.pdf) | ATGCAGCCCACTCACCACGCAGAGATAAGCTTTCAGGATAGAGATACAGCTCTTCCAGACGCATGTTCATCTATATCTGATATTAGCCCCGTTACGTATCCGATCACATTACCTATTATTAAAAACATTTCCGTAACTGCTCATGGTATCAATCTTATCGATAAATTTCCATCAAAGTTCTGCAGCTCTTACATACCCTTCCACTACGGAGGCAATGCGATTAAAACCCCCGATGATCCGGGTGCGATGATGATTACCTTTGCTTTGAAGCCACGGGAGGAATACCAACCCAGTGGTCATATTAACGTATCCAGAGCAAGAGAATTTTATATTAGTTGGGACACGGATTACGTGGGGTCTATCACTACGGCTGATCTTGTGGTATCGGCATCTGCTATTAACTT |  | [This study (gallardo@inia.es)](file:///C:\Users\Cgallardo\Dropbox\Carmina%20AGOSTO%202014\SECUENCIAS%20CRL\Desktop\Carmina%20MARZO%202014\ARTICULOS\VPPA\GENOTIPADO\2007%20Boshoff%20et%20al.pdf) | AGTGCGTATACTTGTGCAGATACCAATGTAGACACTTGTGCAAGCATGTGTGCAGATACCAATGTAGACACCTGTGCAAGCATGTGTGCAGATACCAATGTAGATACCTGTGCAAGCACTTGTACAAGCACAGAATAC |
| 381 | Lt14/WB-Alytus-1 | Lithuania | Wild pig | 2014 |  | II | [This study (gallardo@inia.es)](file:///C:\Users\Cgallardo\Dropbox\Carmina%20AGOSTO%202014\SECUENCIAS%20CRL\Desktop\Carmina%20MARZO%202014\ARTICULOS\VPPA\GENOTIPADO\2007%20Boshoff%20et%20al.pdf) | ATGCAGCCCACTCACCACGCAGAGATAAGCTTTCAGGATAGAGATACAGCTCTTCCAGACGCATGTTCATCTATATCTGATATTAGCCCCGTTACGTATCCGATCACATTACCTATTATTAAAAACATTTCCGTAACTGCTCATGGTATCAATCTTATCGATAAATTTCCATCAAAGTTCTGCAGCTCTTACATACCCTTCCACTACGGAGGCAATGCGATTAAAACCCCCGATGATCCGGGTGCGATGATGATTACCTTTGCTTTGAAGCCACGGGAGGAATACCAACCCAGTGGTCATATTAACGTATCCAGAGCAAGAGAATTTTATATTAGTTGGGACACGGATTACGTGGGGTCTATCACTACGGCTGATCTTGTGGTATCGGCATCTGCTATTAACTT |  | [This study (gallardo@inia.es)](file:///C:\Users\Cgallardo\Dropbox\Carmina%20AGOSTO%202014\SECUENCIAS%20CRL\Desktop\Carmina%20MARZO%202014\ARTICULOS\VPPA\GENOTIPADO\2007%20Boshoff%20et%20al.pdf) | AGTGCGTATACTTGTGCAGATACCAATGTAGACACTTGTGCAAGCATGTGTGCAGATACCAATGTAGACACCTGTGCAAGCATGTGTGCAGATACCAATGTAGATACCTGTGCAAGCACTTGTACAAGCACAGAATAC |
| 382 | Pol14/WB-23947-2#17 | Poland | Wild pig | 2014 |  | II | [This study (gallardo@inia.es)](http://asf-referencelab.info/asf/images/files/publicaciones/Gallardo-et-al-2009a.pdf) | ATGCAGCCCACTCACCACGCAGAGATAAGCTTTCAGGATAGAGATACAGCTCTTCCAGACGCATGTTCATCTATATCTGATATTAGCCCCGTTACGTATCCGATCACATTACCTATTATTAAAAACATTTCCGTAACTGCTCATGGTATCAATCTTATCGATAAATTTCCATCAAAGTTCTGCAGCTCTTACATACCCTTCCACTACGGAGGCAATGCGATTAAAACCCCCGATGATCCGGGTGCGATGATGATTACCTTTGCTTTGAAGCCACGGGAGGAATACCAACCCAGTGGTCATATTAACGTATCCAGAGCAAGAGAATTTTATATTAGTTGGGACACGGATTACGTGGGGTCTATCACTACGGCTGATCTTGTGGTATCGGCATCTGCTATTAACTT |  | [This study (gallardo@inia.es)](file:///C:\Users\Cgallardo\Dropbox\Carmina%20AGOSTO%202014\SECUENCIAS%20CRL\Desktop\Carmina%20MARZO%202014\ARTICULOS\VPPA\GENOTIPADO\2007%20Boshoff%20et%20al.pdf) | AGTGCGTATACTTGTGCAGATACCAATGTAGACACTTGTGCAAGCATGTGTGCAGATACCAATGTAGACACCTGTGCAAGCATGTGTGCAGATACCAATGTAGATACCTGTGCAAGCACTTGTACAAGCACAGAATAC |
| 383 | Pol14/WB-23942-1#18 | Poland | Wild pig | 2014 |  | II | [This study (gallardo@inia.es)](http://asf-referencelab.info/asf/images/files/publicaciones/Gallardo-et-al-2009a.pdf) | ATGCAGCCCACTCACCACGCAGAGATAAGCTTTCAGGATAGAGATACAGCTCTTCCAGACGCATGTTCATCTATATCTGATATTAGCCCCGTTACGTATCCGATCACATTACCTATTATTAAAAACATTTCCGTAACTGCTCATGGTATCAATCTTATCGATAAATTTCCATCAAAGTTCTGCAGCTCTTACATACCCTTCCACTACGGAGGCAATGCGATTAAAACCCCCGATGATCCGGGTGCGATGATGATTACCTTTGCTTTGAAGCCACGGGAGGAATACCAACCCAGTGGTCATATTAACGTATCCAGAGCAAGAGAATTTTATATTAGTTGGGACACGGATTACGTGGGGTCTATCACTACGGCTGATCTTGTGGTATCGGCATCTGCTATTAACTT |  | [This study (gallardo@inia.es)](file:///C:\Users\Cgallardo\Dropbox\Carmina%20AGOSTO%202014\SECUENCIAS%20CRL\Desktop\Carmina%20MARZO%202014\ARTICULOS\VPPA\GENOTIPADO\2007%20Boshoff%20et%20al.pdf) | AGTGCGTATACTTGTGCAGATACCAATGTAGACACTTGTGCAAGCATGTGTGCAGATACCAATGTAGACACCTGTGCAAGCATGTGTGCAGATACCAATGTAGATACCTGTGCAAGCACTTGTACAAGCACAGAATAC |
| 384 | Pol14/WB-23942-3#18 | Poland | Wild pig | 2014 |  | II | [This study (gallardo@inia.es)](http://asf-referencelab.info/asf/images/files/publicaciones/Gallardo-et-al-2009a.pdf) | ATGCAGCCCACTCACCACGCAGAGATAAGCTTTCAGGATAGAGATACAGCTCTTCCAGACGCATGTTCATCTATATCTGATATTAGCCCCGTTACGTATCCGATCACATTACCTATTATTAAAAACATTTCCGTAACTGCTCATGGTATCAATCTTATCGATAAATTTCCATCAAAGTTCTGCAGCTCTTACATACCCTTCCACTACGGAGGCAATGCGATTAAAACCCCCGATGATCCGGGTGCGATGATGATTACCTTTGCTTTGAAGCCACGGGAGGAATACCAACCCAGTGGTCATATTAACGTATCCAGAGCAAGAGAATTTTATATTAGTTGGGACACGGATTACGTGGGGTCTATCACTACGGCTGATCTTGTGGTATCGGCATCTGCTATTAACTT |  | [This study (gallardo@inia.es)](http://asf-referencelab.info/asf/images/files/publicaciones/Nix_et_al_2006.pdf) | AGTGCGTATACTTGTGCAGATACCAATGTAGACACTTGTGCAAGCATGTGTGCAGATACCAATGTAGACACCTGTGCAAGCATGTGTGCAGATACCAATGTAGATACCTGTGCAAGCACTTGTACAAGCACAGAATAC |
| 385 | Pol14/WB-23942-4#18 | Poland | Wild pig | 2014 |  | II | [This study (gallardo@inia.es)](http://asf-referencelab.info/asf/images/files/publicaciones/Gallardo-et-al-2009a.pdf) | ATGCAGCCCACTCACCACGCAGAGATAAGCTTTCAGGATAGAGATACAGCTCTTCCAGACGCATGTTCATCTATATCTGATATTAGCCCCGTTACGTATCCGATCACATTACCTATTATTAAAAACATTTCCGTAACTGCTCATGGTATCAATCTTATCGATAAATTTCCATCAAAGTTCTGCAGCTCTTACATACCCTTCCACTACGGAGGCAATGCGATTAAAACCCCCGATGATCCGGGTGCGATGATGATTACCTTTGCTTTGAAGCCACGGGAGGAATACCAACCCAGTGGTCATATTAACGTATCCAGAGCAAGAGAATTTTATATTAGTTGGGACACGGATTACGTGGGGTCTATCACTACGGCTGATCTTGTGGTATCGGCATCTGCTATTAACTT |  | [This study (gallardo@inia.es)](http://asf-referencelab.info/asf/images/files/publicaciones/Nix_et_al_2006.pdf) | AGTGCGTATACTTGTGCAGATACCAATGTAGACACTTGTGCAAGCATGTGTGCAGATACCAATGTAGACACCTGTGCAAGCATGTGTGCAGATACCAATGTAGATACCTGTGCAAGCACTTGTACAAGCACAGAATAC |
| 386 | Pol14/WB-23942-6#18 | Poland | Wild pig | 2014 |  | II | [This study (gallardo@inia.es)](http://asf-referencelab.info/asf/images/files/publicaciones/Gallardo-et-al-2009a.pdf) | ATGCAGCCCACTCACCACGCAGAGATAAGCTTTCAGGATAGAGATACAGCTCTTCCAGACGCATGTTCATCTATATCTGATATTAGCCCCGTTACGTATCCGATCACATTACCTATTATTAAAAACATTTCCGTAACTGCTCATGGTATCAATCTTATCGATAAATTTCCATCAAAGTTCTGCAGCTCTTACATACCCTTCCACTACGGAGGCAATGCGATTAAAACCCCCGATGATCCGGGTGCGATGATGATTACCTTTGCTTTGAAGCCACGGGAGGAATACCAACCCAGTGGTCATATTAACGTATCCAGAGCAAGAGAATTTTATATTAGTTGGGACACGGATTACGTGGGGTCTATCACTACGGCTGATCTTGTGGTATCGGCATCTGCTATTAACTT |  | [This study (gallardo@inia.es)](http://asf-referencelab.info/asf/images/files/publicaciones/Nix_et_al_2006.pdf) | AGTGCGTATACTTGTGCAGATACCAATGTAGACACTTGTGCAAGCATGTGTGCAGATACCAATGTAGACACCTGTGCAAGCATGTGTGCAGATACCAATGTAGATACCTGTGCAAGCACTTGTACAAGCACAGAATAC |
| 387 | Pol14/WB-23943#18 | Poland | Wild pig | 2014 |  | II | [This study (gallardo@inia.es)](http://asf-referencelab.info/asf/images/files/publicaciones/Gallardo-et-al-2009a.pdf) | ATGCAGCCCACTCACCACGCAGAGATAAGCTTTCAGGATAGAGATACAGCTCTTCCAGACGCATGTTCATCTATATCTGATATTAGCCCCGTTACGTATCCGATCACATTACCTATTATTAAAAACATTTCCGTAACTGCTCATGGTATCAATCTTATCGATAAATTTCCATCAAAGTTCTGCAGCTCTTACATACCCTTCCACTACGGAGGCAATGCGATTAAAACCCCCGATGATCCGGGTGCGATGATGATTACCTTTGCTTTGAAGCCACGGGAGGAATACCAACCCAGTGGTCATATTAACGTATCCAGAGCAAGAGAATTTTATATTAGTTGGGACACGGATTACGTGGGGTCTATCACTACGGCTGATCTTGTGGTATCGGCATCTGCTATTAACTT |  | [This study (gallardo@inia.es)](http://asf-referencelab.info/asf/images/files/publicaciones/Nix_et_al_2006.pdf) | AGTGCGTATACTTGTGCAGATACCAATGTAGACACTTGTGCAAGCATGTGTGCAGATACCAATGTAGACACCTGTGCAAGCATGTGTGCAGATACCAATGTAGATACCTGTGCAAGCACTTGTACAAGCACAGAATAC |
| 388 | Pol14/WB-24305-1#18 | Poland | Wild pig | 2014 |  | II | [This study (gallardo@inia.es)](http://asf-referencelab.info/asf/images/files/publicaciones/Gallardo-et-al-2009a.pdf) | ATGCAGCCCACTCACCACGCAGAGATAAGCTTTCAGGATAGAGATACAGCTCTTCCAGACGCATGTTCATCTATATCTGATATTAGCCCCGTTACGTATCCGATCACATTACCTATTATTAAAAACATTTCCGTAACTGCTCATGGTATCAATCTTATCGATAAATTTCCATCAAAGTTCTGCAGCTCTTACATACCCTTCCACTACGGAGGCAATGCGATTAAAACCCCCGATGATCCGGGTGCGATGATGATTACCTTTGCTTTGAAGCCACGGGAGGAATACCAACCCAGTGGTCATATTAACGTATCCAGAGCAAGAGAATTTTATATTAGTTGGGACACGGATTACGTGGGGTCTATCACTACGGCTGATCTTGTGGTATCGGCATCTGCTATTAACTT |  | [This study (gallardo@inia.es)](http://asf-referencelab.info/asf/images/files/publicaciones/Nix_et_al_2006.pdf) | AGTGCGTATACTTGTGCAGATACCAATGTAGACACTTGTGCAAGCATGTGTGCAGATACCAATGTAGACACCTGTGCAAGCATGTGTGCAGATACCAATGTAGATACCTGTGCAAGCACTTGTACAAGCACAGAATAC |
| 389 | Pol14/WB-24305-2#18 | Poland | Wild pig | 2014 |  | II | [This study (gallardo@inia.es)](http://asf-referencelab.info/asf/images/files/publicaciones/Gallardo-et-al-2009a.pdf) | ATGCAGCCCACTCACCACGCAGAGATAAGCTTTCAGGATAGAGATACAGCTCTTCCAGACGCATGTTCATCTATATCTGATATTAGCCCCGTTACGTATCCGATCACATTACCTATTATTAAAAACATTTCCGTAACTGCTCATGGTATCAATCTTATCGATAAATTTCCATCAAAGTTCTGCAGCTCTTACATACCCTTCCACTACGGAGGCAATGCGATTAAAACCCCCGATGATCCGGGTGCGATGATGATTACCTTTGCTTTGAAGCCACGGGAGGAATACCAACCCAGTGGTCATATTAACGTATCCAGAGCAAGAGAATTTTATATTAGTTGGGACACGGATTACGTGGGGTCTATCACTACGGCTGATCTTGTGGTATCGGCATCTGCTATTAACTT |  | [This study (gallardo@inia.es)](http://asf-referencelab.info/asf/images/files/publicaciones/Nix_et_al_2006.pdf) | AGTGCGTATACTTGTGCAGATACCAATGTAGACACTTGTGCAAGCATGTGTGCAGATACCAATGTAGACACCTGTGCAAGCATGTGTGCAGATACCAATGTAGATACCTGTGCAAGCACTTGTACAAGCACAGAATAC |
| 390 | Pol14/WB-24612#17 | Poland | Wild pig | 2014 |  | II | [This study (gallardo@inia.es)](http://asf-referencelab.info/asf/images/files/publicaciones/Gallardo-et-al-2009a.pdf) | ATGCAGCCCACTCACCACGCAGAGATAAGCTTTCAGGATAGAGATACAGCTCTTCCAGACGCATGTTCATCTATATCTGATATTAGCCCCGTTACGTATCCGATCACATTACCTATTATTAAAAACATTTCCGTAACTGCTCATGGTATCAATCTTATCGATAAATTTCCATCAAAGTTCTGCAGCTCTTACATACCCTTCCACTACGGAGGCAATGCGATTAAAACCCCCGATGATCCGGGTGCGATGATGATTACCTTTGCTTTGAAGCCACGGGAGGAATACCAACCCAGTGGTCATATTAACGTATCCAGAGCAAGAGAATTTTATATTAGTTGGGACACGGATTACGTGGGGTCTATCACTACGGCTGATCTTGTGGTATCGGCATCTGCTATTAACTT |  | [This study (gallardo@inia.es)](http://asf-referencelab.info/asf/images/files/publicaciones/Nix_et_al_2006.pdf) | AGTGCGTATACTTGTGCAGATACCAATGTAGACACTTGTGCAAGCATGTGTGCAGATACCAATGTAGACACCTGTGCAAGCATGTGTGCAGATACCAATGTAGATACCTGTGCAAGCACTTGTACAAGCACAGAATAC |
| 391 | Lt14/WB-Alytus-2 | Lithuania | Wild pig | 2014 |  | II | [This study (gallardo@inia.es)](http://asf-referencelab.info/asf/images/files/publicaciones/Gallardo-et-al-2009a.pdf) | ATGCAGCCCACTCACCACGCAGAGATAAGCTTTCAGGATAGAGATACAGCTCTTCCAGACGCATGTTCATCTATATCTGATATTAGCCCCGTTACGTATCCGATCACATTACCTATTATTAAAAACATTTCCGTAACTGCTCATGGTATCAATCTTATCGATAAATTTCCATCAAAGTTCTGCAGCTCTTACATACCCTTCCACTACGGAGGCAATGCGATTAAAACCCCCGATGATCCGGGTGCGATGATGATTACCTTTGCTTTGAAGCCACGGGAGGAATACCAACCCAGTGGTCATATTAACGTATCCAGAGCAAGAGAATTTTATATTAGTTGGGACACGGATTACGTGGGGTCTATCACTACGGCTGATCTTGTGGTATCGGCATCTGCTATTAACTT |  | [This study (gallardo@inia.es)](http://asf-referencelab.info/asf/images/files/publicaciones/Nix_et_al_2006.pdf) | AGTGCGTATACTTGTGCAGATACCAATGTAGACACTTGTGCAAGCATGTGTGCAGATACCAATGTAGACACCTGTGCAAGCATGTGTGCAGATACCAATGTAGATACCTGTGCAAGCACTTGTACAAGCACAGAATAC |
| 392 | Lt14/WB-Alytus-3 | Lithuania | Wild pig | 2014 |  | II | [This study (gallardo@inia.es)](http://asf-referencelab.info/asf/images/files/publicaciones/Gallardo-et-al-2009a.pdf) | ATGCAGCCCACTCACCACGCAGAGATAAGCTTTCAGGATAGAGATACAGCTCTTCCAGACGCATGTTCATCTATATCTGATATTAGCCCCGTTACGTATCCGATCACATTACCTATTATTAAAAACATTTCCGTAACTGCTCATGGTATCAATCTTATCGATAAATTTCCATCAAAGTTCTGCAGCTCTTACATACCCTTCCACTACGGAGGCAATGCGATTAAAACCCCCGATGATCCGGGTGCGATGATGATTACCTTTGCTTTGAAGCCACGGGAGGAATACCAACCCAGTGGTCATATTAACGTATCCAGAGCAAGAGAATTTTATATTAGTTGGGACACGGATTACGTGGGGTCTATCACTACGGCTGATCTTGTGGTATCGGCATCTGCTATTAACTT |  | [This study (gallardo@inia.es)](http://asf-referencelab.info/asf/images/files/publicaciones/Nix_et_al_2006.pdf) | AGTGCGTATACTTGTGCAGATACCAATGTAGACACTTGTGCAAGCATGTGTGCAGATACCAATGTAGACACCTGTGCAAGCATGTGTGCAGATACCAATGTAGATACCTGTGCAAGCACTTGTACAAGCACAGAATAC |
| 393 | Lt14/WB-Alytus-4 | Lithuania | Wild pig | 2014 |  | II | [This study (gallardo@inia.es)](http://asf-referencelab.info/asf/images/files/publicaciones/Gallardo-et-al-2009a.pdf) | ATGCAGCCCACTCACCACGCAGAGATAAGCTTTCAGGATAGAGATACAGCTCTTCCAGACGCATGTTCATCTATATCTGATATTAGCCCCGTTACGTATCCGATCACATTACCTATTATTAAAAACATTTCCGTAACTGCTCATGGTATCAATCTTATCGATAAATTTCCATCAAAGTTCTGCAGCTCTTACATACCCTTCCACTACGGAGGCAATGCGATTAAAACCCCCGATGATCCGGGTGCGATGATGATTACCTTTGCTTTGAAGCCACGGGAGGAATACCAACCCAGTGGTCATATTAACGTATCCAGAGCAAGAGAATTTTATATTAGTTGGGACACGGATTACGTGGGGTCTATCACTACGGCTGATCTTGTGGTATCGGCATCTGCTATTAACTT |  | [This study (gallardo@inia.es)](http://asf-referencelab.info/asf/images/files/publicaciones/Nix_et_al_2006.pdf) | AGTGCGTATACTTGTGCAGATACCAATGTAGACACTTGTGCAAGCATGTGTGCAGATACCAATGTAGACACCTGTGCAAGCATGTGTGCAGATACCAATGTAGATACCTGTGCAAGCACTTGTACAAGCACAGAATAC |
| 394 | Lt14/WB-Alytus-5 | Lithuania | Wild pig | 2014 |  | II | [This study (gallardo@inia.es)](http://asf-referencelab.info/asf/images/files/publicaciones/Nix_et_al_2006.pdf) | ATGCAGCCCACTCACCACGCAGAGATAAGCTTTCAGGATAGAGATACAGCTCTTCCAGACGCATGTTCATCTATATCTGATATTAGCCCCGTTACGTATCCGATCACATTACCTATTATTAAAAACATTTCCGTAACTGCTCATGGTATCAATCTTATCGATAAATTTCCATCAAAGTTCTGCAGCTCTTACATACCCTTCCACTACGGAGGCAATGCGATTAAAACCCCCGATGATCCGGGTGCGATGATGATTACCTTTGCTTTGAAGCCACGGGAGGAATACCAACCCAGTGGTCATATTAACGTATCCAGAGCAAGAGAATTTTATATTAGTTGGGACACGGATTACGTGGGGTCTATCACTACGGCTGATCTTGTGGTATCGGCATCTGCTATTAACTT |  | [This study (gallardo@inia.es)](http://asf-referencelab.info/asf/images/files/publicaciones/Nix_et_al_2006.pdf) | AGTGCGTATACTTGTGCAGATACCAATGTAGACACTTGTGCAAGCATGTGTGCAGATACCAATGTAGACACCTGTGCAAGCATGTGTGCAGATACCAATGTAGATACCTGTGCAAGCACTTGTACAAGCACAGAATAC |
| 395 | Lt14/WB-Alytus-6 | Lithuania | Wild pig | 2014 |  | II | [This study (gallardo@inia.es)](http://asf-referencelab.info/asf/images/files/publicaciones/Nix_et_al_2006.pdf) | ATGCAGCCCACTCACCACGCAGAGATAAGCTTTCAGGATAGAGATACAGCTCTTCCAGACGCATGTTCATCTATATCTGATATTAGCCCCGTTACGTATCCGATCACATTACCTATTATTAAAAACATTTCCGTAACTGCTCATGGTATCAATCTTATCGATAAATTTCCATCAAAGTTCTGCAGCTCTTACATACCCTTCCACTACGGAGGCAATGCGATTAAAACCCCCGATGATCCGGGTGCGATGATGATTACCTTTGCTTTGAAGCCACGGGAGGAATACCAACCCAGTGGTCATATTAACGTATCCAGAGCAAGAGAATTTTATATTAGTTGGGACACGGATTACGTGGGGTCTATCACTACGGCTGATCTTGTGGTATCGGCATCTGCTATTAACTT |  | [This study (gallardo@inia.es)](http://asf-referencelab.info/asf/images/files/publicaciones/Nix_et_al_2006.pdf) | AGTGCGTATACTTGTGCAGATACCAATGTAGACACTTGTGCAAGCATGTGTGCAGATACCAATGTAGACACCTGTGCAAGCATGTGTGCAGATACCAATGTAGATACCTGTGCAAGCACTTGTACAAGCACAGAATAC |
| 396 | Lt14/WB-Alytus-7 | Lithuania | Wild pig | 2014 |  | II | [This study (gallardo@inia.es)](http://asf-referencelab.info/asf/images/files/publicaciones/Nix_et_al_2006.pdf) | ATGCAGCCCACTCACCACGCAGAGATAAGCTTTCAGGATAGAGATACAGCTCTTCCAGACGCATGTTCATCTATATCTGATATTAGCCCCGTTACGTATCCGATCACATTACCTATTATTAAAAACATTTCCGTAACTGCTCATGGTATCAATCTTATCGATAAATTTCCATCAAAGTTCTGCAGCTCTTACATACCCTTCCACTACGGAGGCAATGCGATTAAAACCCCCGATGATCCGGGTGCGATGATGATTACCTTTGCTTTGAAGCCACGGGAGGAATACCAACCCAGTGGTCATATTAACGTATCCAGAGCAAGAGAATTTTATATTAGTTGGGACACGGATTACGTGGGGTCTATCACTACGGCTGATCTTGTGGTATCGGCATCTGCTATTAACTT |  | [This study (gallardo@inia.es)](http://asf-referencelab.info/asf/images/files/publicaciones/Nix_et_al_2006.pdf) | AGTGCGTATACTTGTGCAGATACCAATGTAGACACTTGTGCAAGCATGTGTGCAGATACCAATGTAGACACCTGTGCAAGCATGTGTGCAGATACCAATGTAGATACCTGTGCAAGCACTTGTACAAGCACAGAATAC |
| 397 | Lt14/WB-Alytus-8 | Lithuania | Wild pig | 2014 |  | II | [This study (gallardo@inia.es)](http://asf-referencelab.info/asf/images/files/publicaciones/Nix_et_al_2006.pdf) | ATGCAGCCCACTCACCACGCAGAGATAAGCTTTCAGGATAGAGATACAGCTCTTCCAGACGCATGTTCATCTATATCTGATATTAGCCCCGTTACGTATCCGATCACATTACCTATTATTAAAAACATTTCCGTAACTGCTCATGGTATCAATCTTATCGATAAATTTCCATCAAAGTTCTGCAGCTCTTACATACCCTTCCACTACGGAGGCAATGCGATTAAAACCCCCGATGATCCGGGTGCGATGATGATTACCTTTGCTTTGAAGCCACGGGAGGAATACCAACCCAGTGGTCATATTAACGTATCCAGAGCAAGAGAATTTTATATTAGTTGGGACACGGATTACGTGGGGTCTATCACTACGGCTGATCTTGTGGTATCGGCATCTGCTATTAACTT |  | [This study (gallardo@inia.es)](http://asf-referencelab.info/asf/images/files/publicaciones/Nix_et_al_2006.pdf) | AGTGCGTATACTTGTGCAGATACCAATGTAGACACTTGTGCAAGCATGTGTGCAGATACCAATGTAGACACCTGTGCAAGCATGTGTGCAGATACCAATGTAGATACCTGTGCAAGCACTTGTACAAGCACAGAATAC |
| 398 | Lt14/WB-Alytus-9 | Lithuania | Wild pig | 2014 |  | II | [This study (gallardo@inia.es)](http://asf-referencelab.info/asf/images/files/publicaciones/Nix_et_al_2006.pdf) | ATGCAGCCCACTCACCACGCAGAGATAAGCTTTCAGGATAGAGATACAGCTCTTCCAGACGCATGTTCATCTATATCTGATATTAGCCCCGTTACGTATCCGATCACATTACCTATTATTAAAAACATTTCCGTAACTGCTCATGGTATCAATCTTATCGATAAATTTCCATCAAAGTTCTGCAGCTCTTACATACCCTTCCACTACGGAGGCAATGCGATTAAAACCCCCGATGATCCGGGTGCGATGATGATTACCTTTGCTTTGAAGCCACGGGAGGAATACCAACCCAGTGGTCATATTAACGTATCCAGAGCAAGAGAATTTTATATTAGTTGGGACACGGATTACGTGGGGTCTATCACTACGGCTGATCTTGTGGTATCGGCATCTGCTATTAACTT |  | [This study (gallardo@inia.es)](http://asf-referencelab.info/asf/images/files/publicaciones/Nix_et_al_2006.pdf) | AGTGCGTATACTTGTGCAGATACCAATGTAGACACTTGTGCAAGCATGTGTGCAGATACCAATGTAGACACCTGTGCAAGCATGTGTGCAGATACCAATGTAGATACCTGTGCAAGCACTTGTACAAGCACAGAATAC |
| 399 | Pol14/WB-25744#19 | Poland | Wild pig | 2014 |  | II | [This study (gallardo@inia.es)](http://asf-referencelab.info/asf/images/files/publicaciones/Gallardo-et-al-2009a.pdf) | ATGCAGCCCACTCACCACGCAGAGATAAGCTTTCAGGATAGAGATACAGCTCTTCCAGACGCATGTTCATCTATATCTGATATTAGCCCCGTTACGTATCCGATCACATTACCTATTATTAAAAACATTTCCGTAACTGCTCATGGTATCAATCTTATCGATAAATTTCCATCAAAGTTCTGCAGCTCTTACATACCCTTCCACTACGGAGGCAATGCGATTAAAACCCCCGATGATCCGGGTGCGATGATGATTACCTTTGCTTTGAAGCCACGGGAGGAATACCAACCCAGTGGTCATATTAACGTATCCAGAGCAAGAGAATTTTATATTAGTTGGGACACGGATTACGTGGGGTCTATCACTACGGCTGATCTTGTGGTATCGGCATCTGCTATTAACTT |  | [This study (gallardo@inia.es)](http://asf-referencelab.info/asf/images/files/publicaciones/Nix_et_al_2006.pdf) | AGTGCGTATACTTGTGCAGATACCAATGTAGACACTTGTGCAAGCATGTGTGCAGATACCAATGTAGACACCTGTGCAAGCATGTGTGCAGATACCAATGTAGATACCTGTGCAAGCACTTGTACAAGCACAGAATAC |
| 400 | Est14/WB-VORU-1 | Estonia | Wild pig | 2014 |  | II | [This study (gallardo@inia.es)](http://asf-referencelab.info/asf/images/files/publicaciones/Nix_et_al_2006.pdf) | ATGCAGCCCACTCACCACGCAGAGATAAGCTTTCAGGATAGAGATACAGCTCTTCCAGACGCATGTTCATCTATATCTGATATTAGCCCCGTTACGTATCCGATCACATTACCTATTATTAAAAACATTTCCGTAACTGCTCATGGTATCAATCTTATCGATAAATTTCCATCAAAGTTCTGCAGCTCTTACATACCCTTCCACTACGGAGGCAATGCGATTAAAACCCCCGATGATCCGGGTGCGATGATGATTACCTTTGCTTTGAAGCCACGGGAGGAATACCAACCCAGTGGTCATATTAACGTATCCAGAGCAAGAGAATTTTATATTAGTTGGGACACGGATTACGTGGGGTCTATCACTACGGCTGATCTTGTGGTATCGGCATCTGCTATTAACTT |  | [This study (gallardo@inia.es)](http://asf-referencelab.info/asf/images/files/publicaciones/Nix_et_al_2006.pdf) | AGTGCGTATACTTGTGCAGATACCAATGTAGACACTTGTGCAAGCATGTGTGCAGATACCAATGTAGACACCTGTGCAAGCATGTGTGCAGATACCAATGTAGATACCTGTGCAAGCACTTGTACAAGCACAGAATAC |
| 401 | Est14/WB-VORU-2 | Estonia | Wild pig | 2014 |  | II | [This study (gallardo@inia.es)](http://asf-referencelab.info/asf/images/files/publicaciones/Nix_et_al_2006.pdf) | ATGCAGCCCACTCACCACGCAGAGATAAGCTTTCAGGATAGAGATACAGCTCTTCCAGACGCATGTTCATCTATATCTGATATTAGCCCCGTTACGTATCCGATCACATTACCTATTATTAAAAACATTTCCGTAACTGCTCATGGTATCAATCTTATCGATAAATTTCCATCAAAGTTCTGCAGCTCTTACATACCCTTCCACTACGGAGGCAATGCGATTAAAACCCCCGATGATCCGGGTGCGATGATGATTACCTTTGCTTTGAAGCCACGGGAGGAATACCAACCCAGTGGTCATATTAACGTATCCAGAGCAAGAGAATTTTATATTAGTTGGGACACGGATTACGTGGGGTCTATCACTACGGCTGATCTTGTGGTATCGGCATCTGCTATTAACTT |  | [This study (gallardo@inia.es)](http://asf-referencelab.info/asf/images/files/publicaciones/Nix_et_al_2006.pdf) | AGTGCGTATACTTGTGCAGATACCAATGTAGACACTTGTGCAAGCATGTGTGCAGATACCAATGTAGACACCTGTGCAAGCATGTGTGCAGATACCAATGTAGATACCTGTGCAAGCACTTGTACAAGCACAGAATAC |
| 402 | Est14/WB-VORU-3 | Estonia | Wild pig | 2014 |  | II | [This study (gallardo@inia.es)](http://asf-referencelab.info/asf/images/files/publicaciones/Gallardo-et-al-2009a.pdf) | ATGCAGCCCACTCACCACGCAGAGATAAGCTTTCAGGATAGAGATACAGCTCTTCCAGACGCATGTTCATCTATATCTGATATTAGCCCCGTTACGTATCCGATCACATTACCTATTATTAAAAACATTTCCGTAACTGCTCATGGTATCAATCTTATCGATAAATTTCCATCAAAGTTCTGCAGCTCTTACATACCCTTCCACTACGGAGGCAATGCGATTAAAACCCCCGATGATCCGGGTGCGATGATGATTACCTTTGCTTTGAAGCCACGGGAGGAATACCAACCCAGTGGTCATATTAACGTATCCAGAGCAAGAGAATTTTATATTAGTTGGGACACGGATTACGTGGGGTCTATCACTACGGCTGATCTTGTGGTATCGGCATCTGCTATTAACTT |  | [This study (gallardo@inia.es)](http://asf-referencelab.info/asf/images/files/publicaciones/Nix_et_al_2006.pdf) | AGTGCGTATACTTGTGCAGATACCAATGTAGACACTTGTGCAAGCATGTGTGCAGATACCAATGTAGACACCTGTGCAAGCATGTGTGCAGATACCAATGTAGATACCTGTGCAAGCACTTGTACAAGCACAGAATAC |
| 403 | Est14/WB-VORU-4 | Estonia | Wild pig | 2014 |  | II | [This study (gallardo@inia.es)](http://asf-referencelab.info/asf/images/files/publicaciones/Gallardo-et-al-2009a.pdf) | ATGCAGCCCACTCACCACGCAGAGATAAGCTTTCAGGATAGAGATACAGCTCTTCCAGACGCATGTTCATCTATATCTGATATTAGCCCCGTTACGTATCCGATCACATTACCTATTATTAAAAACATTTCCGTAACTGCTCATGGTATCAATCTTATCGATAAATTTCCATCAAAGTTCTGCAGCTCTTACATACCCTTCCACTACGGAGGCAATGCGATTAAAACCCCCGATGATCCGGGTGCGATGATGATTACCTTTGCTTTGAAGCCACGGGAGGAATACCAACCCAGTGGTCATATTAACGTATCCAGAGCAAGAGAATTTTATATTAGTTGGGACACGGATTACGTGGGGTCTATCACTACGGCTGATCTTGTGGTATCGGCATCTGCTATTAACTT |  | [This study (gallardo@inia.es)](http://asf-referencelab.info/asf/images/files/publicaciones/Nix_et_al_2006.pdf) | AGTGCGTATACTTGTGCAGATACCAATGTAGACACTTGTGCAAGCATGTGTGCAGATACCAATGTAGACACCTGTGCAAGCATGTGTGCAGATACCAATGTAGATACCTGTGCAAGCACTTGTACAAGCACAGAATAC |
| 404 | Lt14/WB-Vilnius-3 | Lithuania | Wild pig | 2014 |  | II | [This study (gallardo@inia.es)](http://asf-referencelab.info/asf/images/files/publicaciones/Gallardo-et-al-2009a.pdf) | ATGCAGCCCACTCACCACGCAGAGATAAGCTTTCAGGATAGAGATACAGCTCTTCCAGACGCATGTTCATCTATATCTGATATTAGCCCCGTTACGTATCCGATCACATTACCTATTATTAAAAACATTTCCGTAACTGCTCATGGTATCAATCTTATCGATAAATTTCCATCAAAGTTCTGCAGCTCTTACATACCCTTCCACTACGGAGGCAATGCGATTAAAACCCCCGATGATCCGGGTGCGATGATGATTACCTTTGCTTTGAAGCCACGGGAGGAATACCAACCCAGTGGTCATATTAACGTATCCAGAGCAAGAGAATTTTATATTAGTTGGGACACGGATTACGTGGGGTCTATCACTACGGCTGATCTTGTGGTATCGGCATCTGCTATTAACTT |  | [This study (gallardo@inia.es)](http://asf-referencelab.info/asf/images/files/publicaciones/Nix_et_al_2006.pdf) | AGTGCGTATACTTGTGCAGATACCAATGTAGACACTTGTGCAAGCATGTGTGCAGATACCAATGTAGACACCTGTGCAAGCATGTGTGCAGATACCAATGTAGATACCTGTGCAAGCACTTGTACAAGCACAGAATAC |
| 405 | Lt14/WB-Vilnius-4 | Lithuania | Wild pig | 2014 |  | II | [This study (gallardo@inia.es)](http://asf-referencelab.info/asf/images/files/publicaciones/Gallardo-et-al-2009a.pdf) | ATGCAGCCCACTCACCACGCAGAGATAAGCTTTCAGGATAGAGATACAGCTCTTCCAGACGCATGTTCATCTATATCTGATATTAGCCCCGTTACGTATCCGATCACATTACCTATTATTAAAAACATTTCCGTAACTGCTCATGGTATCAATCTTATCGATAAATTTCCATCAAAGTTCTGCAGCTCTTACATACCCTTCCACTACGGAGGCAATGCGATTAAAACCCCCGATGATCCGGGTGCGATGATGATTACCTTTGCTTTGAAGCCACGGGAGGAATACCAACCCAGTGGTCATATTAACGTATCCAGAGCAAGAGAATTTTATATTAGTTGGGACACGGATTACGTGGGGTCTATCACTACGGCTGATCTTGTGGTATCGGCATCTGCTATTAACTT |  | [This study (gallardo@inia.es)](http://asf-referencelab.info/asf/images/files/publicaciones/Nix_et_al_2006.pdf) | AGTGCGTATACTTGTGCAGATACCAATGTAGACACTTGTGCAAGCATGTGTGCAGATACCAATGTAGACACCTGTGCAAGCATGTGTGCAGATACCAATGTAGATACCTGTGCAAGCACTTGTACAAGCACAGAATAC |
| 406 | Lt14/WB-Vilnius-5 | Lithuania | Wild pig | 2014 |  | II | [This study (gallardo@inia.es)](http://asf-referencelab.info/asf/images/files/publicaciones/Gallardo-et-al-2009a.pdf) | ATGCAGCCCACTCACCACGCAGAGATAAGCTTTCAGGATAGAGATACAGCTCTTCCAGACGCATGTTCATCTATATCTGATATTAGCCCCGTTACGTATCCGATCACATTACCTATTATTAAAAACATTTCCGTAACTGCTCATGGTATCAATCTTATCGATAAATTTCCATCAAAGTTCTGCAGCTCTTACATACCCTTCCACTACGGAGGCAATGCGATTAAAACCCCCGATGATCCGGGTGCGATGATGATTACCTTTGCTTTGAAGCCACGGGAGGAATACCAACCCAGTGGTCATATTAACGTATCCAGAGCAAGAGAATTTTATATTAGTTGGGACACGGATTACGTGGGGTCTATCACTACGGCTGATCTTGTGGTATCGGCATCTGCTATTAACTT |  | [This study (gallardo@inia.es)](http://asf-referencelab.info/asf/images/files/publicaciones/Nix_et_al_2006.pdf) | AGTGCGTATACTTGTGCAGATACCAATGTAGACACTTGTGCAAGCATGTGTGCAGATACCAATGTAGACACCTGTGCAAGCATGTGTGCAGATACCAATGTAGATACCTGTGCAAGCACTTGTACAAGCACAGAATAC |
| 407 | Lt14/WB-Vilnius-6 | Lithuania | Wild pig | 2014 |  | II | [This study (gallardo@inia.es)](http://asf-referencelab.info/asf/images/files/publicaciones/Gallardo-et-al-2009a.pdf) | ATGCAGCCCACTCACCACGCAGAGATAAGCTTTCAGGATAGAGATACAGCTCTTCCAGACGCATGTTCATCTATATCTGATATTAGCCCCGTTACGTATCCGATCACATTACCTATTATTAAAAACATTTCCGTAACTGCTCATGGTATCAATCTTATCGATAAATTTCCATCAAAGTTCTGCAGCTCTTACATACCCTTCCACTACGGAGGCAATGCGATTAAAACCCCCGATGATCCGGGTGCGATGATGATTACCTTTGCTTTGAAGCCACGGGAGGAATACCAACCCAGTGGTCATATTAACGTATCCAGAGCAAGAGAATTTTATATTAGTTGGGACACGGATTACGTGGGGTCTATCACTACGGCTGATCTTGTGGTATCGGCATCTGCTATTAACTT |  | [This study (gallardo@inia.es)](http://asf-referencelab.info/asf/images/files/publicaciones/Nix_et_al_2006.pdf) | AGTGCGTATACTTGTGCAGATACCAATGTAGACACTTGTGCAAGCATGTGTGCAGATACCAATGTAGACACCTGTGCAAGCATGTGTGCAGATACCAATGTAGATACCTGTGCAAGCACTTGTACAAGCACAGAATAC |
| 408 | Lt14/WB-Vilnius-7 | Lithuania | Wild pig | 2014 |  | II | [This study (gallardo@inia.es)](http://asf-referencelab.info/asf/images/files/publicaciones/Gallardo-et-al-2009a.pdf) | ATGCAGCCCACTCACCACGCAGAGATAAGCTTTCAGGATAGAGATACAGCTCTTCCAGACGCATGTTCATCTATATCTGATATTAGCCCCGTTACGTATCCGATCACATTACCTATTATTAAAAACATTTCCGTAACTGCTCATGGTATCAATCTTATCGATAAATTTCCATCAAAGTTCTGCAGCTCTTACATACCCTTCCACTACGGAGGCAATGCGATTAAAACCCCCGATGATCCGGGTGCGATGATGATTACCTTTGCTTTGAAGCCACGGGAGGAATACCAACCCAGTGGTCATATTAACGTATCCAGAGCAAGAGAATTTTATATTAGTTGGGACACGGATTACGTGGGGTCTATCACTACGGCTGATCTTGTGGTATCGGCATCTGCTATTAACTT |  | [This study (gallardo@inia.es)](http://asf-referencelab.info/asf/images/files/publicaciones/Nix_et_al_2006.pdf) | AGTGCGTATACTTGTGCAGATACCAATGTAGACACTTGTGCAAGCATGTGTGCAGATACCAATGTAGACACCTGTGCAAGCATGTGTGCAGATACCAATGTAGATACCTGTGCAAGCACTTGTACAAGCACAGAATAC |
| 409 | Lt14/WB-Rokiškis-1 | Lithuania | Wild pig | 2014 |  | II | [This study (gallardo@inia.es)](http://asf-referencelab.info/asf/images/files/publicaciones/Gallardo-et-al-2009a.pdf) | ATGCAGCCCACTCACCACGCAGAGATAAGCTTTCAGGATAGAGATACAGCTCTTCCAGACGCATGTTCATCTATATCTGATATTAGCCCCGTTACGTATCCGATCACATTACCTATTATTAAAAACATTTCCGTAACTGCTCATGGTATCAATCTTATCGATAAATTTCCATCAAAGTTCTGCAGCTCTTACATACCCTTCCACTACGGAGGCAATGCGATTAAAACCCCCGATGATCCGGGTGCGATGATGATTACCTTTGCTTTGAAGCCACGGGAGGAATACCAACCCAGTGGTCATATTAACGTATCCAGAGCAAGAGAATTTTATATTAGTTGGGACACGGATTACGTGGGGTCTATCACTACGGCTGATCTTGTGGTATCGGCATCTGCTATTAACTT |  | [This study (gallardo@inia.es)](http://asf-referencelab.info/asf/images/files/publicaciones/Nix_et_al_2006.pdf) | AGTGCGTATACTTGTGCAGATACCAATGTAGACACTTGTGCAAGCATGTGTGCAGATACCAATGTAGACACCTGTGCAAGCATGTGTGCAGATACCAATGTAGATACCTGTGCAAGCACTTGTACAAGCACAGAATAC |
| 410 | Lt14/WB-Vilnius-8 | Lithuania | Wild pig | 2014 |  | II | [This study (gallardo@inia.es)](http://asf-referencelab.info/asf/images/files/publicaciones/Gallardo-et-al-2009a.pdf) | ATGCAGCCCACTCACCACGCAGAGATAAGCTTTCAGGATAGAGATACAGCTCTTCCAGACGCATGTTCATCTATATCTGATATTAGCCCCGTTACGTATCCGATCACATTACCTATTATTAAAAACATTTCCGTAACTGCTCATGGTATCAATCTTATCGATAAATTTCCATCAAAGTTCTGCAGCTCTTACATACCCTTCCACTACGGAGGCAATGCGATTAAAACCCCCGATGATCCGGGTGCGATGATGATTACCTTTGCTTTGAAGCCACGGGAGGAATACCAACCCAGTGGTCATATTAACGTATCCAGAGCAAGAGAATTTTATATTAGTTGGGACACGGATTACGTGGGGTCTATCACTACGGCTGATCTTGTGGTATCGGCATCTGCTATTAACTT |  | [This study (gallardo@inia.es)](http://asf-referencelab.info/asf/images/files/publicaciones/Nix_et_al_2006.pdf) | AGTGCGTATACTTGTGCAGATACCAATGTAGACACTTGTGCAAGCATGTGTGCAGATACCAATGTAGACACCTGTGCAAGCATGTGTGCAGATACCAATGTAGATACCTGTGCAAGCACTTGTACAAGCACAGAATAC |
| 411 | Lt14/WB-Alytus-10 | Lithuania | Wild pig | 2014 |  | II | [This study (gallardo@inia.es)](http://asf-referencelab.info/asf/images/files/publicaciones/Gallardo-et-al-2009a.pdf) | ATGCAGCCCACTCACCACGCAGAGATAAGCTTTCAGGATAGAGATACAGCTCTTCCAGACGCATGTTCATCTATATCTGATATTAGCCCCGTTACGTATCCGATCACATTACCTATTATTAAAAACATTTCCGTAACTGCTCATGGTATCAATCTTATCGATAAATTTCCATCAAAGTTCTGCAGCTCTTACATACCCTTCCACTACGGAGGCAATGCGATTAAAACCCCCGATGATCCGGGTGCGATGATGATTACCTTTGCTTTGAAGCCACGGGAGGAATACCAACCCAGTGGTCATATTAACGTATCCAGAGCAAGAGAATTTTATATTAGTTGGGACACGGATTACGTGGGGTCTATCACTACGGCTGATCTTGTGGTATCGGCATCTGCTATTAACTT |  | [This study (gallardo@inia.es)](http://asf-referencelab.info/asf/images/files/publicaciones/Nix_et_al_2006.pdf) | AGTGCGTATACTTGTGCAGATACCAATGTAGACACTTGTGCAAGCATGTGTGCAGATACCAATGTAGACACCTGTGCAAGCATGTGTGCAGATACCAATGTAGATACCTGTGCAAGCACTTGTACAAGCACAGAATAC |
| 412 | Lt14/WB-Alytus-11 | Lithuania | Wild pig | 2014 |  | II | [This study (gallardo@inia.es)](http://asf-referencelab.info/asf/images/files/publicaciones/Gallardo-et-al-2009a.pdf) | ATGCAGCCCACTCACCACGCAGAGATAAGCTTTCAGGATAGAGATACAGCTCTTCCAGACGCATGTTCATCTATATCTGATATTAGCCCCGTTACGTATCCGATCACATTACCTATTATTAAAAACATTTCCGTAACTGCTCATGGTATCAATCTTATCGATAAATTTCCATCAAAGTTCTGCAGCTCTTACATACCCTTCCACTACGGAGGCAATGCGATTAAAACCCCCGATGATCCGGGTGCGATGATGATTACCTTTGCTTTGAAGCCACGGGAGGAATACCAACCCAGTGGTCATATTAACGTATCCAGAGCAAGAGAATTTTATATTAGTTGGGACACGGATTACGTGGGGTCTATCACTACGGCTGATCTTGTGGTATCGGCATCTGCTATTAACTT |  | [This study (gallardo@inia.es)](http://asf-referencelab.info/asf/images/files/publicaciones/Nix_et_al_2006.pdf) | AGTGCGTATACTTGTGCAGATACCAATGTAGACACTTGTGCAAGCATGTGTGCAGATACCAATGTAGACACCTGTGCAAGCATGTGTGCAGATACCAATGTAGATACCTGTGCAAGCACTTGTACAAGCACAGAATAC |
| 413 | Lt14/WB-Alytus-12 | Lithuania | Wild pig | 2014 |  | II | [This study (gallardo@inia.es)](http://asf-referencelab.info/asf/images/files/publicaciones/Gallardo-et-al-2009a.pdf) | ATGCAGCCCACTCACCACGCAGAGATAAGCTTTCAGGATAGAGATACAGCTCTTCCAGACGCATGTTCATCTATATCTGATATTAGCCCCGTTACGTATCCGATCACATTACCTATTATTAAAAACATTTCCGTAACTGCTCATGGTATCAATCTTATCGATAAATTTCCATCAAAGTTCTGCAGCTCTTACATACCCTTCCACTACGGAGGCAATGCGATTAAAACCCCCGATGATCCGGGTGCGATGATGATTACCTTTGCTTTGAAGCCACGGGAGGAATACCAACCCAGTGGTCATATTAACGTATCCAGAGCAAGAGAATTTTATATTAGTTGGGACACGGATTACGTGGGGTCTATCACTACGGCTGATCTTGTGGTATCGGCATCTGCTATTAACTT |  | [This study (gallardo@inia.es)](http://asf-referencelab.info/asf/images/files/publicaciones/Nix_et_al_2006.pdf) | AGTGCGTATACTTGTGCAGATACCAATGTAGACACTTGTGCAAGCATGTGTGCAGATACCAATGTAGACACCTGTGCAAGCATGTGTGCAGATACCAATGTAGATACCTGTGCAAGCACTTGTACAAGCACAGAATAC |
| 414 | Lt14/WB-Ignalina-3 | Lithuania | Wild pig | 2014 |  | II | [This study (gallardo@inia.es)](http://asf-referencelab.info/asf/images/files/publicaciones/Gallardo-et-al-2009a.pdf) | ATGCAGCCCACTCACCACGCAGAGATAAGCTTTCAGGATAGAGATACAGCTCTTCCAGACGCATGTTCATCTATATCTGATATTAGCCCCGTTACGTATCCGATCACATTACCTATTATTAAAAACATTTCCGTAACTGCTCATGGTATCAATCTTATCGATAAATTTCCATCAAAGTTCTGCAGCTCTTACATACCCTTCCACTACGGAGGCAATGCGATTAAAACCCCCGATGATCCGGGTGCGATGATGATTACCTTTGCTTTGAAGCCACGGGAGGAATACCAACCCAGTGGTCATATTAACGTATCCAGAGCAAGAGAATTTTATATTAGTTGGGACACGGATTACGTGGGGTCTATCACTACGGCTGATCTTGTGGTATCGGCATCTGCTATTAACTT |  | [This study (gallardo@inia.es)](http://asf-referencelab.info/asf/images/files/publicaciones/Nix_et_al_2006.pdf) | AGTGCGTATACTTGTGCAGATACCAATGTAGACACTTGTGCAAGCATGTGTGCAGATACCAATGTAGACACCTGTGCAAGCATGTGTGCAGATACCAATGTAGATACCTGTGCAAGCACTTGTACAAGCACAGAATAC |
| 415 | Lt14/WB-Alytus-13 | Lithuania | Wild pig | 2014 |  | II | [This study (gallardo@inia.es)](http://asf-referencelab.info/asf/images/files/publicaciones/Gallardo-et-al-2009a.pdf) | ATGCAGCCCACTCACCACGCAGAGATAAGCTTTCAGGATAGAGATACAGCTCTTCCAGACGCATGTTCATCTATATCTGATATTAGCCCCGTTACGTATCCGATCACATTACCTATTATTAAAAACATTTCCGTAACTGCTCATGGTATCAATCTTATCGATAAATTTCCATCAAAGTTCTGCAGCTCTTACATACCCTTCCACTACGGAGGCAATGCGATTAAAACCCCCGATGATCCGGGTGCGATGATGATTACCTTTGCTTTGAAGCCACGGGAGGAATACCAACCCAGTGGTCATATTAACGTATCCAGAGCAAGAGAATTTTATATTAGTTGGGACACGGATTACGTGGGGTCTATCACTACGGCTGATCTTGTGGTATCGGCATCTGCTATTAACTT |  | [This study (gallardo@inia.es)](http://asf-referencelab.info/asf/images/files/publicaciones/Nix_et_al_2006.pdf) | AGTGCGTATACTTGTGCAGATACCAATGTAGACACTTGTGCAAGCATGTGTGCAGATACCAATGTAGACACCTGTGCAAGCATGTGTGCAGATACCAATGTAGATACCTGTGCAAGCACTTGTACAAGCACAGAATAC |
| 416 | Pol14/WB-28374#20 | Poland | Wild pig | 2014 |  | II | [This study (gallardo@inia.es)](http://asf-referencelab.info/asf/images/files/publicaciones/Gallardo-et-al-2009a.pdf) | ATGCAGCCCACTCACCACGCAGAGATAAGCTTTCAGGATAGAGATACAGCTCTTCCAGACGCATGTTCATCTATATCTGATATTAGCCCCGTTACGTATCCGATCACATTACCTATTATTAAAAACATTTCCGTAACTGCTCATGGTATCAATCTTATCGATAAATTTCCATCAAAGTTCTGCAGCTCTTACATACCCTTCCACTACGGAGGCAATGCGATTAAAACCCCCGATGATCCGGGTGCGATGATGATTACCTTTGCTTTGAAGCCACGGGAGGAATACCAACCCAGTGGTCATATTAACGTATCCAGAGCAAGAGAATTTTATATTAGTTGGGACACGGATTACGTGGGGTCTATCACTACGGCTGATCTTGTGGTATCGGCATCTGCTATTAACTT |  | [This study (gallardo@inia.es)](http://asf-referencelab.info/asf/images/files/publicaciones/Nix_et_al_2006.pdf) | AGTGCGTATACTTGTGCAGATACCAATGTAGACACTTGTGCAAGCATGTGTGCAGATACCAATGTAGACACCTGTGCAAGCATGTGTGCAGATACCAATGTAGATACCTGTGCAAGCACTTGTACAAGCACAGAATAC |
| 417 | Lt14/WB-Rokiškis-2 | Lithuania | Wild pig | 2014 |  | II | [This study (gallardo@inia.es)](http://asf-referencelab.info/asf/images/files/publicaciones/Gallardo-et-al-2009a.pdf) | ATGCAGCCCACTCACCACGCAGAGATAAGCTTTCAGGATAGAGATACAGCTCTTCCAGACGCATGTTCATCTATATCTGATATTAGCCCCGTTACGTATCCGATCACATTACCTATTATTAAAAACATTTCCGTAACTGCTCATGGTATCAATCTTATCGATAAATTTCCATCAAAGTTCTGCAGCTCTTACATACCCTTCCACTACGGAGGCAATGCGATTAAAACCCCCGATGATCCGGGTGCGATGATGATTACCTTTGCTTTGAAGCCACGGGAGGAATACCAACCCAGTGGTCATATTAACGTATCCAGAGCAAGAGAATTTTATATTAGTTGGGACACGGATTACGTGGGGTCTATCACTACGGCTGATCTTGTGGTATCGGCATCTGCTATTAACTT |  | [This study (gallardo@inia.es)](http://asf-referencelab.info/asf/images/files/publicaciones/Nix_et_al_2006.pdf) | AGTGCGTATACTTGTGCAGATACCAATGTAGACACTTGTGCAAGCATGTGTGCAGATACCAATGTAGACACCTGTGCAAGCATGTGTGCAGATACCAATGTAGATACCTGTGCAAGCACTTGTACAAGCACAGAATAC |
| 418 | Lt14/WB-Rokiškis-3 | Lithuania | Wild pig | 2014 |  | II | [This study (gallardo@inia.es)](http://asf-referencelab.info/asf/images/files/publicaciones/Gallardo-et-al-2009a.pdf) | ATGCAGCCCACTCACCACGCAGAGATAAGCTTTCAGGATAGAGATACAGCTCTTCCAGACGCATGTTCATCTATATCTGATATTAGCCCCGTTACGTATCCGATCACATTACCTATTATTAAAAACATTTCCGTAACTGCTCATGGTATCAATCTTATCGATAAATTTCCATCAAAGTTCTGCAGCTCTTACATACCCTTCCACTACGGAGGCAATGCGATTAAAACCCCCGATGATCCGGGTGCGATGATGATTACCTTTGCTTTGAAGCCACGGGAGGAATACCAACCCAGTGGTCATATTAACGTATCCAGAGCAAGAGAATTTTATATTAGTTGGGACACGGATTACGTGGGGTCTATCACTACGGCTGATCTTGTGGTATCGGCATCTGCTATTAACTT |  | [This study (gallardo@inia.es)](http://asf-referencelab.info/asf/images/files/publicaciones/Nix_et_al_2006.pdf) | AGTGCGTATACTTGTGCAGATACCAATGTAGACACTTGTGCAAGCATGTGTGCAGATACCAATGTAGACACCTGTGCAAGCATGTGTGCAGATACCAATGTAGATACCTGTGCAAGCACTTGTACAAGCACAGAATAC |
| 419 | Lt14/WB-Rokiškis-4 | Lithuania | Wild pig | 2014 |  | II | [This study (gallardo@inia.es)](http://asf-referencelab.info/asf/images/files/publicaciones/Gallardo-et-al-2009a.pdf) | ATGCAGCCCACTCACCACGCAGAGATAAGCTTTCAGGATAGAGATACAGCTCTTCCAGACGCATGTTCATCTATATCTGATATTAGCCCCGTTACGTATCCGATCACATTACCTATTATTAAAAACATTTCCGTAACTGCTCATGGTATCAATCTTATCGATAAATTTCCATCAAAGTTCTGCAGCTCTTACATACCCTTCCACTACGGAGGCAATGCGATTAAAACCCCCGATGATCCGGGTGCGATGATGATTACCTTTGCTTTGAAGCCACGGGAGGAATACCAACCCAGTGGTCATATTAACGTATCCAGAGCAAGAGAATTTTATATTAGTTGGGACACGGATTACGTGGGGTCTATCACTACGGCTGATCTTGTGGTATCGGCATCTGCTATTAACTT |  | [This study (gallardo@inia.es)](http://asf-referencelab.info/asf/images/files/publicaciones/Nix_et_al_2006.pdf) | AGTGCGTATACTTGTGCAGATACCAATGTAGACACTTGTGCAAGCATGTGTGCAGATACCAATGTAGACACCTGTGCAAGCATGTGTGCAGATACCAATGTAGATACCTGTGCAAGCACTTGTACAAGCACAGAATAC |
| 420 | Lt14/WB-Rokiškis-5 | Lithuania | Wild pig | 2014 |  | II | [This study (gallardo@inia.es)](http://asf-referencelab.info/asf/images/files/publicaciones/Gallardo-et-al-2009a.pdf) | ATGCAGCCCACTCACCACGCAGAGATAAGCTTTCAGGATAGAGATACAGCTCTTCCAGACGCATGTTCATCTATATCTGATATTAGCCCCGTTACGTATCCGATCACATTACCTATTATTAAAAACATTTCCGTAACTGCTCATGGTATCAATCTTATCGATAAATTTCCATCAAAGTTCTGCAGCTCTTACATACCCTTCCACTACGGAGGCAATGCGATTAAAACCCCCGATGATCCGGGTGCGATGATGATTACCTTTGCTTTGAAGCCACGGGAGGAATACCAACCCAGTGGTCATATTAACGTATCCAGAGCAAGAGAATTTTATATTAGTTGGGACACGGATTACGTGGGGTCTATCACTACGGCTGATCTTGTGGTATCGGCATCTGCTATTAACTT |  | [This study (gallardo@inia.es)](http://asf-referencelab.info/asf/images/files/publicaciones/Nix_et_al_2006.pdf) | AGTGCGTATACTTGTGCAGATACCAATGTAGACACTTGTGCAAGCATGTGTGCAGATACCAATGTAGACACCTGTGCAAGCATGTGTGCAGATACCAATGTAGATACCTGTGCAAGCACTTGTACAAGCACAGAATAC |
| 421 | Lt14/WB-Rokiškis-6 | Lithuania | Wild pig | 2014 |  | II | [This study (gallardo@inia.es)](http://asf-referencelab.info/asf/images/files/publicaciones/Gallardo-et-al-2009a.pdf) | ATGCAGCCCACTCACCACGCAGAGATAAGCTTTCAGGATAGAGATACAGCTCTTCCAGACGCATGTTCATCTATATCTGATATTAGCCCCGTTACGTATCCGATCACATTACCTATTATTAAAAACATTTCCGTAACTGCTCATGGTATCAATCTTATCGATAAATTTCCATCAAAGTTCTGCAGCTCTTACATACCCTTCCACTACGGAGGCAATGCGATTAAAACCCCCGATGATCCGGGTGCGATGATGATTACCTTTGCTTTGAAGCCACGGGAGGAATACCAACCCAGTGGTCATATTAACGTATCCAGAGCAAGAGAATTTTATATTAGTTGGGACACGGATTACGTGGGGTCTATCACTACGGCTGATCTTGTGGTATCGGCATCTGCTATTAACTT |  | [This study (gallardo@inia.es)](http://asf-referencelab.info/asf/images/files/publicaciones/Nix_et_al_2006.pdf) | AGTGCGTATACTTGTGCAGATACCAATGTAGACACTTGTGCAAGCATGTGTGCAGATACCAATGTAGACACCTGTGCAAGCATGTGTGCAGATACCAATGTAGATACCTGTGCAAGCACTTGTACAAGCACAGAATAC |
| 422 | Lt14/WB-Vilnius-10 | Lithuania | Wild pig | 2014 |  | II | [This study (gallardo@inia.es)](http://asf-referencelab.info/asf/images/files/publicaciones/Gallardo-et-al-2009a.pdf) | ATGCAGCCCACTCACCACGCAGAGATAAGCTTTCAGGATAGAGATACAGCTCTTCCAGACGCATGTTCATCTATATCTGATATTAGCCCCGTTACGTATCCGATCACATTACCTATTATTAAAAACATTTCCGTAACTGCTCATGGTATCAATCTTATCGATAAATTTCCATCAAAGTTCTGCAGCTCTTACATACCCTTCCACTACGGAGGCAATGCGATTAAAACCCCCGATGATCCGGGTGCGATGATGATTACCTTTGCTTTGAAGCCACGGGAGGAATACCAACCCAGTGGTCATATTAACGTATCCAGAGCAAGAGAATTTTATATTAGTTGGGACACGGATTACGTGGGGTCTATCACTACGGCTGATCTTGTGGTATCGGCATCTGCTATTAACTT |  | [This study (gallardo@inia.es)](http://asf-referencelab.info/asf/images/files/publicaciones/Nix_et_al_2006.pdf) | AGTGCGTATACTTGTGCAGATACCAATGTAGACACTTGTGCAAGCATGTGTGCAGATACCAATGTAGACACCTGTGCAAGCATGTGTGCAGATACCAATGTAGATACCTGTGCAAGCACTTGTACAAGCACAGAATAC |
| 423 | Lt14/WB-Vilnius-9 | Lithuania | Wild pig | 2014 |  | II | [This study (gallardo@inia.es)](http://asf-referencelab.info/asf/images/files/publicaciones/Gallardo-et-al-2009a.pdf) | ATGCAGCCCACTCACCACGCAGAGATAAGCTTTCAGGATAGAGATACAGCTCTTCCAGACGCATGTTCATCTATATCTGATATTAGCCCCGTTACGTATCCGATCACATTACCTATTATTAAAAACATTTCCGTAACTGCTCATGGTATCAATCTTATCGATAAATTTCCATCAAAGTTCTGCAGCTCTTACATACCCTTCCACTACGGAGGCAATGCGATTAAAACCCCCGATGATCCGGGTGCGATGATGATTACCTTTGCTTTGAAGCCACGGGAGGAATACCAACCCAGTGGTCATATTAACGTATCCAGAGCAAGAGAATTTTATATTAGTTGGGACACGGATTACGTGGGGTCTATCACTACGGCTGATCTTGTGGTATCGGCATCTGCTATTAACTT |  | [This study (gallardo@inia.es)](http://asf-referencelab.info/asf/images/files/publicaciones/Nix_et_al_2006.pdf) | AGTGCGTATACTTGTGCAGATACCAATGTAGACACTTGTGCAAGCATGTGTGCAGATACCAATGTAGACACCTGTGCAAGCATGTGTGCAGATACCAATGTAGATACCTGTGCAAGCACTTGTACAAGCACAGAATAC |
| 424 | Lt14/WB-Rokiškis-7 | Lithuania | Wild pig | 2014 |  | II | [This study (gallardo@inia.es)](http://asf-referencelab.info/asf/images/files/publicaciones/Gallardo-et-al-2009a.pdf) | ATGCAGCCCACTCACCACGCAGAGATAAGCTTTCAGGATAGAGATACAGCTCTTCCAGACGCATGTTCATCTATATCTGATATTAGCCCCGTTACGTATCCGATCACATTACCTATTATTAAAAACATTTCCGTAACTGCTCATGGTATCAATCTTATCGATAAATTTCCATCAAAGTTCTGCAGCTCTTACATACCCTTCCACTACGGAGGCAATGCGATTAAAACCCCCGATGATCCGGGTGCGATGATGATTACCTTTGCTTTGAAGCCACGGGAGGAATACCAACCCAGTGGTCATATTAACGTATCCAGAGCAAGAGAATTTTATATTAGTTGGGACACGGATTACGTGGGGTCTATCACTACGGCTGATCTTGTGGTATCGGCATCTGCTATTAACTT |  | [This study (gallardo@inia.es)](http://asf-referencelab.info/asf/images/files/publicaciones/Nix_et_al_2006.pdf) | AGTGCGTATACTTGTGCAGATACCAATGTAGACACTTGTGCAAGCATGTGTGCAGATACCAATGTAGACACCTGTGCAAGCATGTGTGCAGATACCAATGTAGATACCTGTGCAAGCACTTGTACAAGCACAGAATAC |
| 425 | Lt14/WB-Rokiškis-8 | Lithuania | Wild pig | 2014 |  | II | [This study (gallardo@inia.es)](http://asf-referencelab.info/asf/images/files/publicaciones/Gallardo-et-al-2009a.pdf) | ATGCAGCCCACTCACCACGCAGAGATAAGCTTTCAGGATAGAGATACAGCTCTTCCAGACGCATGTTCATCTATATCTGATATTAGCCCCGTTACGTATCCGATCACATTACCTATTATTAAAAACATTTCCGTAACTGCTCATGGTATCAATCTTATCGATAAATTTCCATCAAAGTTCTGCAGCTCTTACATACCCTTCCACTACGGAGGCAATGCGATTAAAACCCCCGATGATCCGGGTGCGATGATGATTACCTTTGCTTTGAAGCCACGGGAGGAATACCAACCCAGTGGTCATATTAACGTATCCAGAGCAAGAGAATTTTATATTAGTTGGGACACGGATTACGTGGGGTCTATCACTACGGCTGATCTTGTGGTATCGGCATCTGCTATTAACTT |  | [This study (gallardo@inia.es)](http://asf-referencelab.info/asf/images/files/publicaciones/Nix_et_al_2006.pdf) | AGTGCGTATACTTGTGCAGATACCAATGTAGACACTTGTGCAAGCATGTGTGCAGATACCAATGTAGACACCTGTGCAAGCATGTGTGCAGATACCAATGTAGATACCTGTGCAAGCACTTGTACAAGCACAGAATAC |
| 426 | Pol14/WB-29321#21 | Poland | Wild pig | 2014 |  | II | [This study (gallardo@inia.es)](http://asf-referencelab.info/asf/images/files/publicaciones/Gallardo-et-al-2009a.pdf) | ATGCAGCCCACTCACCACGCAGAGATAAGCTTTCAGGATAGAGATACAGCTCTTCCAGACGCATGTTCATCTATATCTGATATTAGCCCCGTTACGTATCCGATCACATTACCTATTATTAAAAACATTTCCGTAACTGCTCATGGTATCAATCTTATCGATAAATTTCCATCAAAGTTCTGCAGCTCTTACATACCCTTCCACTACGGAGGCAATGCGATTAAAACCCCCGATGATCCGGGTGCGATGATGATTACCTTTGCTTTGAAGCCACGGGAGGAATACCAACCCAGTGGTCATATTAACGTATCCAGAGCAAGAGAATTTTATATTAGTTGGGACACGGATTACGTGGGGTCTATCACTACGGCTGATCTTGTGGTATCGGCATCTGCTATTAACTT |  | [This study (gallardo@inia.es)](http://asf-referencelab.info/asf/images/files/publicaciones/Nix_et_al_2006.pdf) | AGTGCGTATACTTGTGCAGATACCAATGTAGACACTTGTGCAAGCATGTGTGCAGATACCAATGTAGACACCTGTGCAAGCATGTGTGCAGATACCAATGTAGATACCTGTGCAAGCACTTGTACAAGCACAGAATAC |
| 427 | Pol14/WB-30011#22 | Poland | Wild pig | 2014 |  | II | [This study (gallardo@inia.es)](http://asf-referencelab.info/asf/images/files/publicaciones/Gallardo-et-al-2009a.pdf) | ATGCAGCCCACTCACCACGCAGAGATAAGCTTTCAGGATAGAGATACAGCTCTTCCAGACGCATGTTCATCTATATCTGATATTAGCCCCGTTACGTATCCGATCACATTACCTATTATTAAAAACATTTCCGTAACTGCTCATGGTATCAATCTTATCGATAAATTTCCATCAAAGTTCTGCAGCTCTTACATACCCTTCCACTACGGAGGCAATGCGATTAAAACCCCCGATGATCCGGGTGCGATGATGATTACCTTTGCTTTGAAGCCACGGGAGGAATACCAACCCAGTGGTCATATTAACGTATCCAGAGCAAGAGAATTTTATATTAGTTGGGACACGGATTACGTGGGGTCTATCACTACGGCTGATCTTGTGGTATCGGCATCTGCTATTAACTT |  | [This study (gallardo@inia.es)](http://asf-referencelab.info/asf/images/files/publicaciones/Nix_et_al_2006.pdf) | AGTGCGTATACTTGTGCAGATACCAATGTAGACACTTGTGCAAGCATGTGTGCAGATACCAATGTAGACACCTGTGCAAGCATGTGTGCAGATACCAATGTAGATACCTGTGCAAGCACTTGTACAAGCACAGAATAC |
| 428 | Lt14/WB-Ignalina-4 | Lithuania | Wild pig | 2014 |  | II | [This study (gallardo@inia.es)](http://asf-referencelab.info/asf/images/files/publicaciones/Gallardo-et-al-2009a.pdf) | ATGCAGCCCACTCACCACGCAGAGATAAGCTTTCAGGATAGAGATACAGCTCTTCCAGACGCATGTTCATCTATATCTGATATTAGCCCCGTTACGTATCCGATCACATTACCTATTATTAAAAACATTTCCGTAACTGCTCATGGTATCAATCTTATCGATAAATTTCCATCAAAGTTCTGCAGCTCTTACATACCCTTCCACTACGGAGGCAATGCGATTAAAACCCCCGATGATCCGGGTGCGATGATGATTACCTTTGCTTTGAAGCCACGGGAGGAATACCAACCCAGTGGTCATATTAACGTATCCAGAGCAAGAGAATTTTATATTAGTTGGGACACGGATTACGTGGGGTCTATCACTACGGCTGATCTTGTGGTATCGGCATCTGCTATTAACTT |  | [This study (gallardo@inia.es)](http://asf-referencelab.info/asf/images/files/publicaciones/Nix_et_al_2006.pdf) | AGTGCGTATACTTGTGCAGATACCAATGTAGACACTTGTGCAAGCATGTGTGCAGATACCAATGTAGACACCTGTGCAAGCATGTGTGCAGATACCAATGTAGATACCTGTGCAAGCACTTGTACAAGCACAGAATAC |
| 429 | Pol14/WB-30258#23 | Poland | Wild pig | 2014 |  | II | [This study (gallardo@inia.es)](http://asf-referencelab.info/asf/images/files/publicaciones/Gallardo-et-al-2009a.pdf) | ATGCAGCCCACTCACCACGCAGAGATAAGCTTTCAGGATAGAGATACAGCTCTTCCAGACGCATGTTCATCTATATCTGATATTAGCCCCGTTACGTATCCGATCACATTACCTATTATTAAAAACATTTCCGTAACTGCTCATGGTATCAATCTTATCGATAAATTTCCATCAAAGTTCTGCAGCTCTTACATACCCTTCCACTACGGAGGCAATGCGATTAAAACCCCCGATGATCCGGGTGCGATGATGATTACCTTTGCTTTGAAGCCACGGGAGGAATACCAACCCAGTGGTCATATTAACGTATCCAGAGCAAGAGAATTTTATATTAGTTGGGACACGGATTACGTGGGGTCTATCACTACGGCTGATCTTGTGGTATCGGCATCTGCTATTAACTT |  | [This study (gallardo@inia.es)](http://asf-referencelab.info/asf/images/files/publicaciones/Nix_et_al_2006.pdf) | AGTGCGTATACTTGTGCAGATACCAATGTAGACACTTGTGCAAGCATGTGTGCAGATACCAATGTAGACACCTGTGCAAGCATGTGTGCAGATACCAATGTAGATACCTGTGCAAGCACTTGTACAAGCACAGAATAC |
| 430 | Pol14/WB-30420#24 | Poland | Wild pig | 2014 |  | II | [This study (gallardo@inia.es)](http://asf-referencelab.info/asf/images/files/publicaciones/Gallardo-et-al-2009a.pdf) | ATGCAGCCCACTCACCACGCAGAGATAAGCTTTCAGGATAGAGATACAGCTCTTCCAGACGCATGTTCATCTATATCTGATATTAGCCCCGTTACGTATCCGATCACATTACCTATTATTAAAAACATTTCCGTAACTGCTCATGGTATCAATCTTATCGATAAATTTCCATCAAAGTTCTGCAGCTCTTACATACCCTTCCACTACGGAGGCAATGCGATTAAAACCCCCGATGATCCGGGTGCGATGATGATTACCTTTGCTTTGAAGCCACGGGAGGAATACCAACCCAGTGGTCATATTAACGTATCCAGAGCAAGAGAATTTTATATTAGTTGGGACACGGATTACGTGGGGTCTATCACTACGGCTGATCTTGTGGTATCGGCATCTGCTATTAACTT |  | [This study (gallardo@inia.es)](http://asf-referencelab.info/asf/images/files/publicaciones/Nix_et_al_2006.pdf) | AGTGCGTATACTTGTGCAGATACCAATGTAGACACTTGTGCAAGCATGTGTGCAGATACCAATGTAGACACCTGTGCAAGCATGTGTGCAGATACCAATGTAGATACCTGTGCAAGCACTTGTACAAGCACAGAATAC |
[truncated: 165,235 more chars]
