# Supplementary material for: Phylodynamics and evolutionary epidemiology of African swine fever p72-CVR genes in Eurasia and Africa
Source: PLoS One. 2018 Feb 28;13(2):e0192565. doi: 10.1371/journal.pone.0192565 (PMC5831051; doi:10.1371/journal.pone.0192565)
Supplement: S2 Table — (DOCX) [file pone.0192565.s002.docx]

### **S2 Table.** Summary profile of ASF vp72-CVR gene sequences isolated in Eurasia and Africa between 1960 and 2015 per country (N = 665).

| **Region/Country** | **Number of Sequences** |
| --- | --- |
| *Eurasia* | |
| Armenia | 1 |
| Azerbaijan | 2 |
| Belarus | 1 |
| Estonia | 84 |
| France | 1 |
| Georgia | 2 |
| Italy | 52 |
| Latvia | 29 |
| Lithuania | 150 |
| Poland | 104 |
| Portugal | 16 |
| Russia | 15 |
| Spain | 14 |
| Ukraine | 1 |
| **Total** | **472** |
| *West and Central Africa* | |
| Angola | 1 |
| Benin | 3 |
| Burkina Faso | 20 |
| Cameroon | 3 |
| Cape Verde | 2 |
| Democratic republic of Congo | 1 |
| Ivory Coast | 6 |
| Ghana | 7 |
| Nigeria | 18 |
| Republic of Congo | 2 |
| Togo | 3 |
| **Total** | **66** |
| *East Africa* | |
| Burundi | 3 |
| Kenya | 64 |
| Madagascar | 1 |
| Malawi | 6 |
| Mozambique | 13 |
| Tanzania | 2 |
| Uganda | 36 |
| Zambia | 2 |
| **Total** | **127** |
