# Supplementary material for: Phylodynamics and evolutionary epidemiology of African swine fever p72-CVR genes in Eurasia and Africa
Source: PLoS One. 2018 Feb 28;13(2):e0192565. doi: 10.1371/journal.pone.0192565 (PMC5831051; doi:10.1371/journal.pone.0192565)
Supplement: S3 Table — (DOCX) [file pone.0192565.s003.docx]

### S3 Table. Summary profile of ASF vp72-CVR gene sequences isolated in Eurasia and Africa between 1960 and 2015 (N = 665) per geographical region and host species. Number of sequences used in the phylogenetic molecular clock analyses (n = 96) are enclosed between brackets.

|  | Host species | | | | |
| --- | --- | --- | --- | --- | --- |
| Region/Continent | *Unknown* | *Wild suid* | *Domestic pig* | *Tick* | **Total** |
| Eurasia | 1(0) | 304 (5) | 163 (27) | 4 (1) | **472 (33)** |
| East Africa | - | 5 (3) | 100 (22) | 22 (8) | **127 (33)** |
| West Africa | - | - | 66 (30) | - | **66 (30)** |
| **Total** | **1(0)** | **309 (8)** | **329 (79)** | **26 (9)** | **665 (96)** |
