# Supplementary material for: Phylodynamics and evolutionary epidemiology of African swine fever p72-CVR genes in Eurasia and Africa
Source: PLoS One. 2018 Feb 28;13(2):e0192565. doi: 10.1371/journal.pone.0192565 (PMC5831051; doi:10.1371/journal.pone.0192565)
Supplement: S4 Table — BFs based on SS marginal likelihood estimates are on the upper off-diagonal of this table, while BFs based on PS marginal likelihood estimates are on the lower off-diagonal of this table. Best fitting model is boldfaced. (DOCX) [file pone.0192565.s004.docx]

### S4 Table. Bayes factors (BFs) comparisons of ASF vp72-CVR genes relaxed-clock models using stepping-stone (SS) and path-sampling (PS) methods. BFs based on SS marginal likelihood estimates are on the upper off-diagonal of this table, while BFs based on PS marginal likelihood estimates are on the lower off-diagonal of this table. Best fitting model is boldfaced.

| Relaxed-clock |  |  |  |  | Bayes Factor |  |  |  |  |  |
| --- | --- | --- | --- | --- | --- | --- | --- | --- | --- | --- |
| Model | UCED+CP | UCED+EG | UCED+EGx | UCED+LG | UCED+GMRF | UCLN+CP | UCLN+EG | UCLN+EGx | UCLN+LG | UCLN+GMRF |
| UCED^a^+CP^b^ | –– | -208.59 | -182.97 | 509.75 | 193.6125 | 3.62 | 0.08 | -3.89 | 487.2 | 67.53 |
| **UCED+EG^c^** | **15.9** | **––** | **25.62** | **718.34** | **402.2025** | **212.21** | **208.67** | **204.7** | **695.79** | **276.12** |
| UCED+EGx^d^ | 17.6 | 1.7 | –– | 692.72 | 376.5825 | 186.59 | 183.05 | 179.08 | 670.17 | 250.5 |
| UCED+LG^e^ | -284.2 | -300.1 | -301.8 | –– | -316.1375 | -506.13 | -509.67 | -513.64 | -22.55 | -442.22 |
| UCED+BSg^f*^ | -215.33 | -231.23 | -232.93 | 68.87 | –– | -189.9925 | -193.5325 | -197.5025 | 293.5875 | -126.0825 |
| UCLN^g^+CP | -0.7 | -16.6 | -18.3 | 283.5 | 214.63 | –– | -3.54 | -7.51 | 483.58 | 63.91 |
| UCLN+EG | 10.5 | -5.4 | -7.1 | 294.7 | 225.83 | 11.2 | –– | -3.97 | 487.12 | 67.45 |
| UCLN+EGx | 5.5 | -12.1 | -12.1 | 289.7 | 220.83 | 6.2 | -5 | –– | 491.09 | 71.42 |
| UCLN+LG | -388.55 | -104.35 | -406.15 | -104.35 | -173.22 | -387.85 | -399.05 | -394.05 | –– | -419.67 |
| UCLN+*BSg | -20.5 | 194.83 | -38.1 | 263.7 | 194.83 | -19.8 | -31 | -26 | 368.05 | –– |

^a^Uncorrelated relaxed clock with exponential distribution

^b^Constant population size coalescent model

^c^Expansion population size coalescent model

^d^Exponential population size coalescent model

^e^Logistic population size coalescent model

^f^Bayesian Skygrid coalescent model

^g^Uncorrelated relaxed clock with log-normal distribution

*non-parameteric demographic model
