# Supplementary material for: Phylodynamics and evolutionary epidemiology of African swine fever p72-CVR genes in Eurasia and Africa
Source: PLoS One. 2018 Feb 28;13(2):e0192565. doi: 10.1371/journal.pone.0192565 (PMC5831051; doi:10.1371/journal.pone.0192565)
Supplement: S1 Fig — Support given at nodes based on through bootstrap search using 10 runs, with 100 ML replicates in each run, implemented in RAxML version 8. Scale bar indicate substitution rate per site for all sequence data (N = 665). (PDF) [file pone.0192565.s005.pdf]

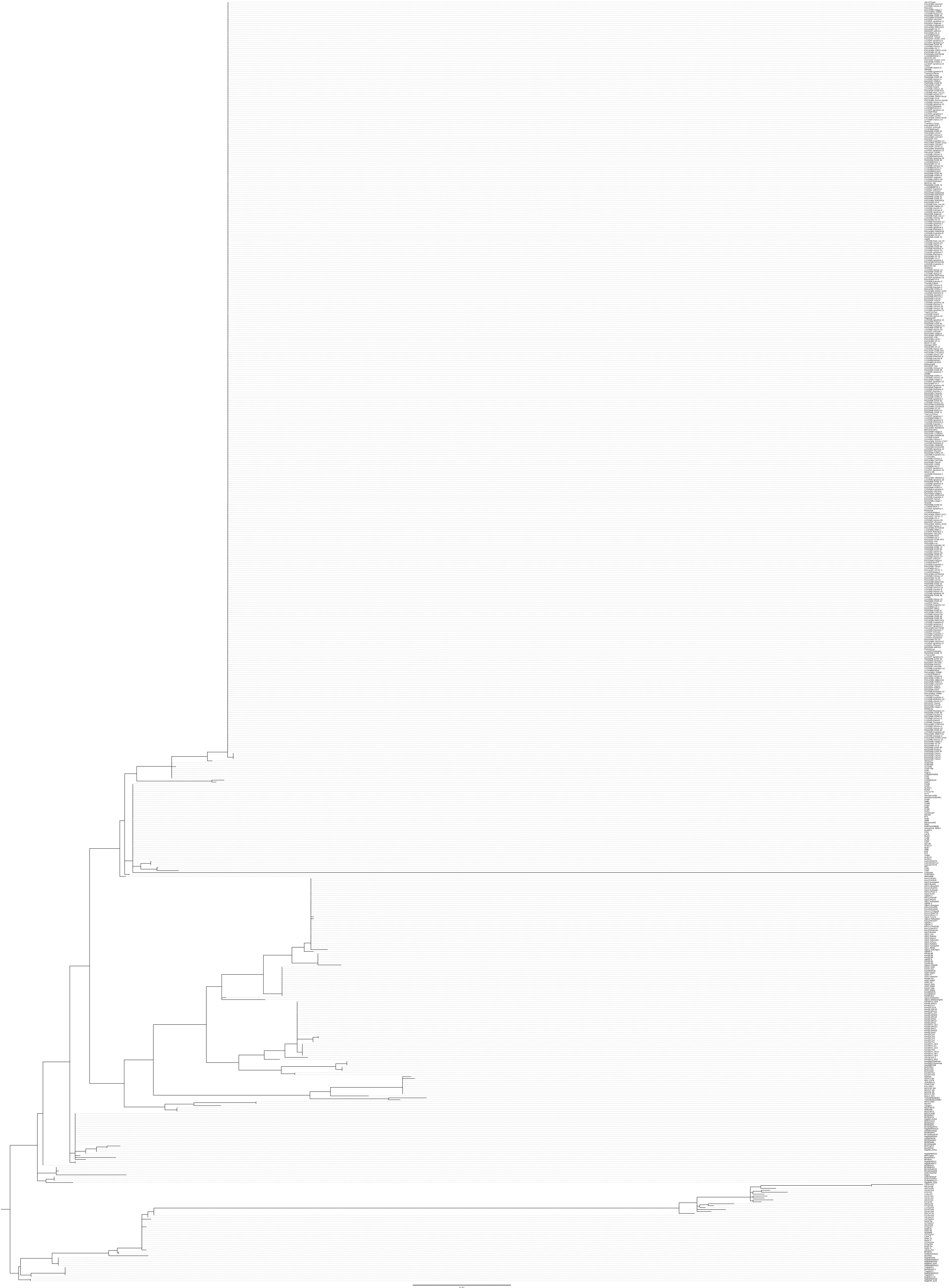

0.07
